# Comprehensive machine learning models for predicting therapeutic targets in type 2 diabetes utilizing molecular and biochemical features in rats

**DOI:** 10.3389/fendo.2024.1384984

**Published:** 2024-05-24

**Authors:** Marwa Matboli, Hiba S. Al-Amodi, Abdelrahman Khaled, Radwa Khaled, Marian M. S. Roushdy, Marwa Ali, Gouda Ibrahim Diab, Mahmoud Fawzy Elnagar, Rasha A. Elmansy, Hagir H. TAhmed, Enshrah M. E. Ahmed, Doaa M. A. Elzoghby, Hala F. M.Kamel, Mohamed F. Farag, Hind A. ELsawi, Laila M. Farid, Mariam B. Abouelkhair, Eman K. Habib, Heba Fikry, Lobna A. Saleh, Ibrahim H. Aboughaleb

**Affiliations:** ^1^ Medical Biochemistry and Molecular Biology Department, Faculty of Medicine, Ain Shams University, Cairo, Egypt; ^2^ Biochemistry Department, Faculty of Medicine, Umm Al-Qura University, Makkah, Saudi Arabia; ^3^ Bioinformatics Group, Center of Informatics Sciences (CIS), School of Information Technology and Computer Sciences, Nile University, Giza, Egypt; ^4^ Biotechnology/Biomolecular Chemistry Department, Faculty of Science, Cairo University, Cairo, Egypt; ^5^ Medicinal Biochemistry and Molecular Biology Department, Modern University for Technology and Information, Cairo, Egypt; ^6^ Biomedical Engineering Department, Egyptian Armed Forces, Cairo, Egypt; ^7^ Zoology Department, Faculty of Science, Ain Shams University, Cairo, Egypt; ^8^ Anatomy Unit, Department of Basic Medical Sciences, College of Medicine and Medical Sciences, Qassim University, Buraydah, Saudi Arabia; ^9^ Department of Anatomy and Cell Biology, Faculty of Medicine, Ain Shams University, Cairo, Egypt; ^10^ Anatomy Unit, Department of Basic Medical Sciences, College of Medicine and Medical Sciences, AlNeelain University, Khartoum, Sudan; ^11^ Pathology Unit, Department of Basic Medical Sciences, College of Medicine and Medical Sciences, Gassim University, Buraydah, Saudi Arabia; ^12^ Clinical Pathology, Faculty of Medicine, Ain Shams University, Cairo, Egypt; ^13^ Medical Physiology Department, Armed Forces College of Medicine, Cairo, Egypt; ^14^ Department of Internal Medicine, Badr University in Cairo, Badr, Egypt; ^15^ Pathology Department, Faculty of Medicine, Ain Shams University, Cairo, Egypt; ^16^ Department of Anatomy and Cell Biology, Faculty of Medicine, Galala University, Attaka, Suez Governorate, Egypt; ^17^ Department of Histology, Faculty of Medicine, Ain Shams University, Cairo, Egypt; ^18^ Department of Clinical Pharmacology, Faculty of Medicine, Ain Shams University, Cairo, Egypt

**Keywords:** type 2 diabetes, therapeutic targets, machine learning, drug response, rats

## Abstract

**Introduction:**

With the increasing prevalence of type 2 diabetes mellitus (T2DM), there is an urgent need to discover effective therapeutic targets for this complex condition. Coding and non-coding RNAs, with traditional biochemical parameters, have shown promise as viable targets for therapy. Machine learning (ML) techniques have emerged as powerful tools for predicting drug responses.

**Method:**

In this study, we developed an ML-based model to identify the most influential features for drug response in the treatment of type 2 diabetes using three medicinal plant-based drugs (Rosavin, Caffeic acid, and Isorhamnetin), and a probiotics drug (Z-biotic), at different doses. A hundred rats were randomly assigned to ten groups, including a normal group, a streptozotocin-induced diabetic group, and eight treated groups. Serum samples were collected for biochemical analysis, while liver tissues (L) and adipose tissues (A) underwent histopathological examination and molecular biomarker extraction using quantitative PCR. Utilizing five machine learning algorithms, we integrated 32 molecular features and 12 biochemical features to select the most predictive targets for each model and the combined model.

**Results and discussion:**

Our results indicated that high doses of the selected drugs effectively mitigated liver inflammation, reduced insulin resistance, and improved lipid profiles and renal function biomarkers. The machine learning model identified 13 molecular features, 10 biochemical features, and 20 combined features with an accuracy of 80% and AUC (0.894, 0.93, and 0.896), respectively. This study presents an ML model that accurately identifies effective therapeutic targets implicated in the molecular pathways associated with T2DM pathogenesis.

## 1 Introduction

Globally, the burden of diabetes mellitus (DM) is projected to rise to 1.3 billion people by 2050, making it one of the most widely spread diseases worldwide (1). Type 2 diabetes mellitus (T2DM) is the prevalent form of DM, it is hallmarked by hyperglycemia, insulin resistance, and ultimate decrease in β-cells insulin secretion (2). When it develops further worsening comorbidities emerge including micro- and macrovascular disease, leading to kidney dysfunction, diabetic retinopathy, blindness, heart disease, stroke, and lower limb amputations (3).

Diabetes mellitus is a highly heterogeneous entity (4). To enhance our comprehension of the underlying biological mechanisms and identify individuals at risk, it is crucial to investigate the genetic contributions to diabetes. Such knowledge can ultimately lead to the development of more precise and effective therapeutic approaches. As T2DM progresses, it often necessitates the simultaneous administration of multiple medications that target different pathophysiologic pathways (5). This combined treatment approach aims to regulate blood glucose levels and mitigate the progression of complications.

However, emerging evidence suggests that inflammatory pathways play a pivotal role as common mediators in the natural course of diabetes when influenced by risk factors (6). Interestingly, a previous study discovered that HFD-induced mice increased mitochondrial DNA (mtDNA) release into the cytosol of adipocytes, activating the cGAS-STING pathway and inflammatory response, resulting in chronic inflammation in adipose tissue and insulin resistance (7, 8). Moreover, autophagy plays a crucial role in T2DM pathogenesis as it protects cells from the damaging effects of oxidative stress and endoplasmic reticulum stress, which is essential for the survival and proper functioning of β-cell and insulin sensitivity, however, when the autophagic system in β-cells fails, it can exacerbate β-cell dysfunction, particularly in the presence of insulin resistance, potentially leading to hyperglycemia (9). A growing body of evidence suggests that enhanced autophagy, triggered by insulin resistance, may act as a safeguarding mechanism against the deterioration and increased apoptosis of pancreatic β-cells (10). This underscores the potential significance of autophagy modulation in the pursuit of therapeutic strategies aimed at preserving β-cell function in T2DM and ultimately managing hyperglycemia.

The growing availability of high-throughput technologies in large populations, such as genomics and transcriptomics with linked medical record data supports the development of new computational approaches for drug targeting using molecular biomarkers in addition to the traditional biomarkers. Noncoding RNAs (ncRNAs) including microRNAs (miRNAs) and long noncoding RNAs (LncRNAs), exhibit diverse functions in post-transcriptional gene regulation, epigenetic gene silencing, modulation of insulin secretion, and endoplasmic reticulum stress (11, 12). These ncRNAs have been intricately linked to the development of T2DM (13, 14) For instance, mir-375, a highly expressed miRNA in islet cells, plays a crucial role in insulin secretion and β-cell functioning. Dysregulation of mir-375 has been linked to impaired insulin secretion and β-cell dysfunction (15). On the other hand, lncRNAs have been implicated in the pathogenesis of insulin resistance and the maintenance of glucose homeostasis by regulating inflammatory and lipogenic processes (16, 17).

Despite the presence of chemical anti-diabetic agents, possible adverse effects and limited efficiency could occur. As a result, recent endeavors have explored other treatment options for the rising T2DM prevalence (18). Medicinal plants are of paramount importance in maintaining body health with no side effects compared with synthetic drugs (19). Likewise, several clinical studies have been conducted to examine the impact of probiotics on improving glycemic index, lipid profile, glucose metabolism, and insulin sensitivity (20). These investigations were fuelled by the observation that the gut microbiota of diabetic patients tends to be altered (21). Such changes in the gut microbiota could lead to metabolic endotoxemia, which occurs through the release of lipopolysaccharides and could trigger inflammation and insulin resistance (22).

Several researches has confirmed that inflammation is closely linked to the pathogenesis of T2DM and its complications. Many anti-diabetic drugs are usually prescribed to diabetic patients, to decrease the progression of T2DM through modulation of inflammation. However, those anti-diabetic drugs are often not successful as a result of side effects; such as sulphonylureas & biguanides may cause acute severe hypoglycemia and lactic acidosis. Therefore, researchers are searching for efficient natural therapeutic targets with less or no side effects. Natural products’ derived bioactive molecules have been proven to improve insulin resistance and associated complications through suppression of inflammatory signaling pathways (23). Moreover, the utilization of probiotics as dietary supplements gains popularity because gut microbiota dysbiosis significantly contributes to T2DM (24). Zbiotics as newly implemented enginerred probiotics as a new complementary therapeutic strategy that alleviate oxidative stress and beneficial effect on reducing blood glucose levels, HOMA-IR, and HbA1c.

This study investigates the potency of medicinal plant-based drugs and probiotics in modulating T2DM in streptozotocin-high-fat diet-induced induced rats. Isorhamnetin is a flavonoid found in sea buckthorn, medicinal plants, and ginkgo fruits (25). It possesses various pharmacological effects, including anti-inflammatory, anti-tumor, antioxidant, antibacterial, and antiviral properties (26, 27). It has been found to promote glucose uptake, maintain glucose homeostasis, and improve dyslipidemia in mice with T2DM (28). It can also reduce the expression of inflammatory cytokines and enhance the health of the gut microbiota in T2DM mice (29). *Rhodiola rosea L*. is abundant in flavonoids, glycosides, coumarins, and organic acid compounds (30). Rosavin, the R. rosea bioactive compound, possesses protective properties against inflammation, reduces blood glucose levels, exhibits antiviral and antitumor effects, and promotes blood circulation activation (31, 32). The caffeic acid extract derived from *Artemisia dracunculus L*. has been proven to enhance insulin receptor signaling. Semisynthetic compounds derived from caffeic acid induce DNA damage and apoptosis in tumor cells by activating autophagy (33). Furthermore, it plays a protective role in preventing renal damage (34, 35). ZBiotics is an engineered probiotic that involves the use of a genetically modified strain of *B. subtilis* by incorporating an acetaldehyde dehydrogenase gene. The modified strain converts acetaldehyde derived from ethanol into acetic acid,thereby reducing the potential harm caused by alcohol consumption (36). Animal toxicity studies have indicated ZBiotics’ high level of safety (37). However,its potential as a therapeutic drug for diabetes has not been investigated yet.

In order to examine the clinical trials’ data related to caffeic acid and related compounds in diabetic patients, we searched the largest clinical trial database at ‘https://clinicaltrials.gov’. No search results were obtained with the keywords ‘saffeic acid and diabetes mellitus’. Since propolis found in beehive is a major source of caffeic acid derivatives, therefore we searched with keywords ‘propolis and diabetes mellitus’ on the database. Three studies were found in which propolis was administered orally or applied topically to diabetic patients(ClinicalTrials.gov Identifier: NCT03416127 phase 2 in patients with type 2 DM for 12 weeks, NCT02794506 Phase: 4. in Type 2 DM, periodontitis, and NCT03649243 in diabetic foot ulcer). Similarly, no results found for rosavin in T2DM except one recent clinical trial in diabetic kidney disease(NCT06176599). More than 20 clinical trials were found correlating Rhodiola Rosea the origin of rosavin in many mental disorders, metabolic diseases, coronary diseases. Additionally, one clinical trial(NCT00961909) assessing the efficacy of Artemisia dracunculus plant rich in isorhamantin in T2DM. Moreover, no results found for Zbiotics in T2DM (**Supplementary Table S1**).

To minimize the consequences of diabetes and improve patient care, researchers have explored various fields, such as machine learning (ML) and artificial intelligence (AI), by applying ML techniques in the field of biology, researchers have significantly improved the precision of prediction models (38). Numerous studies have looked into using ML techniques to predict the occurrence of diabetes (39, 40).

Deberneh et al. created an ML model that can predict the occurrence of type 2 diabetes (T2D). The models categorize input data instances into three conditions: normal (non-diabetic), prediabetes, or diabetes. To construct their prediction model, they identified key features using a data-driven technique that includes an analysis of variance (ANOVA) test and recursive feature elimination methods. Also, they compared the performance of various machine learning models, such as LR, support vector machine (SVM), RF, and XGBoost algorithms (41). Wei S et al. built an ML model for diabetes detection. The study assessed two crucial data processing techniques: Principal Component Analysis (PCA) and Linear Discriminant Analysis (LDA) across various machine learning algorithms. The highest accuracy achieved among the five algorithms tested (Neural Network, Support Vector Machine, Decision Tree, LR, and Naïve Bayes) was 77.86% using 10-fold cross-validation (42).

Elsherbini AM et al. employed machine learning techniques to identify significant genes associated with diabetes and assess their potential as biomarkers for early detection. The analysis highlighted the *HLA-DQB1* gene as a promising biomarker to detect diabetes through ML algorithms with decent accuracy (43). Xu et al. introduced a computational model utilizing stochastic gradient boosting. They incorporated six features, encompassing molecular structures, structural similarities, ATC code similarities, protein–protein interaction, chemical-chemical interaction, and disease pathways (44). Moreover, Costello et al. and Jang et al. performed extensive comparative analyses of machine learning methods for drug response prediction in cancer cell lines, recommending using elastic net or ridge regression with input features from all genomic profiling platforms (45, 46).

In this study, we aimed to utilize standard statistical methods and machine learning techniques to leverage the gene expression patterns and their epigenetic regulators in the livers and adipose tissues, along with conventional biochemical parameters to identify predictive features that could be used to assess the response to medicinal plant-based drugs and probiotics in an animal model of T2DM.

## 2 Materials and methods

### 2.1 Chemicals

Rosavin was obtained from Aktin Chemicals, Inc (Cat.No. APC-380, China), While isorhamnetin 3-O-acylglucosides, Z-Biotics, Caffeic acid, and sodium citrate buffer were purchased from Sigma Chemical Co, St. Louis, Mo, USA.

### 2.2 Experimental design

**Figure 1:**
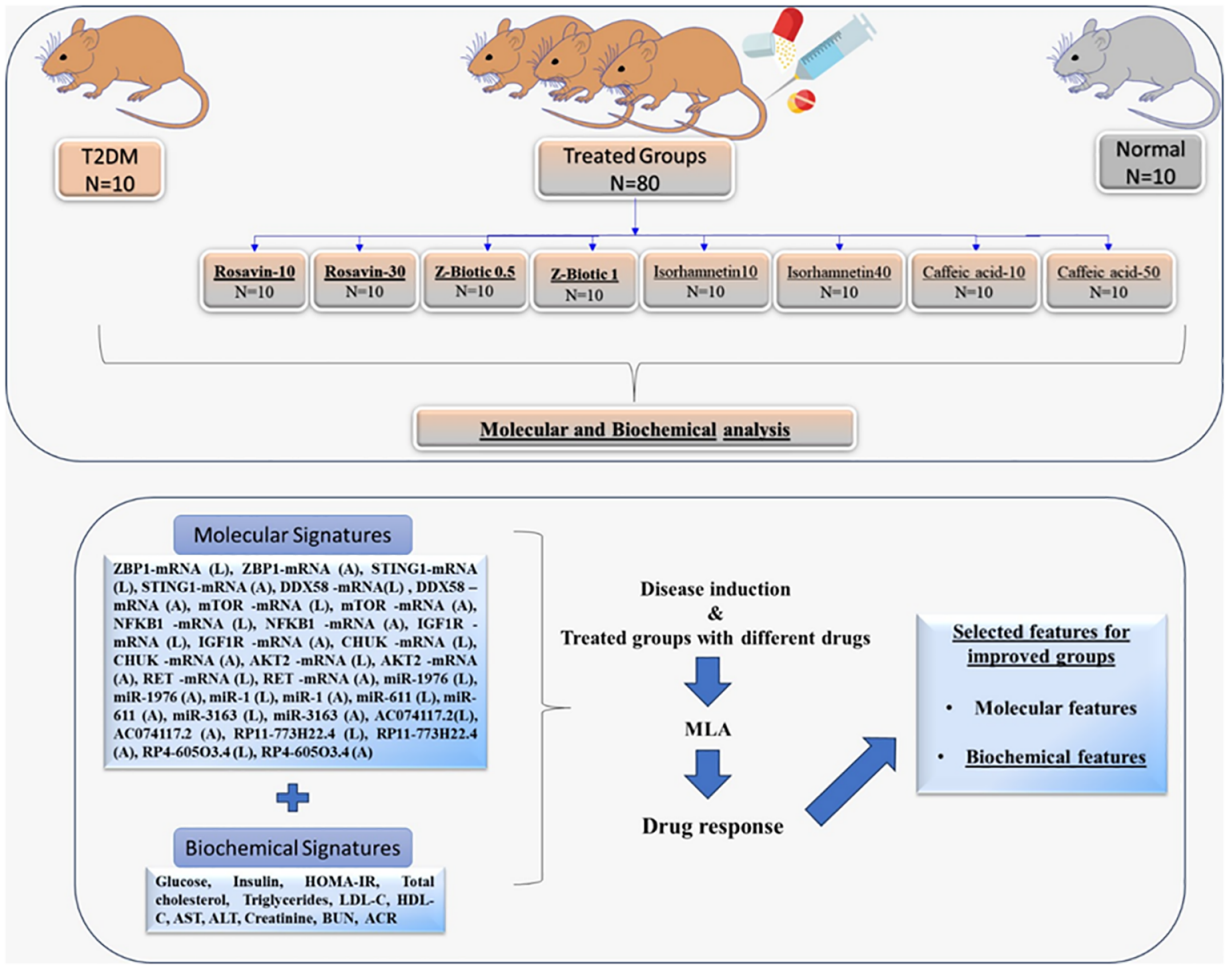
Workflow describing the animal groups subjected to molecular and biochemical analysis for ML-model building identify signatures associated with drug response.T2DM, Type 2 Diabetes Mellitus; (L) extracted from the Liver tissues; (A) extracted from the Adipose tissues; HOMA-IR, Homeostatic Model Assessment of Insulin Resistance; LDL-C, low-density lipoprotein cholesterol; HDL-C, high-density lipoprotein cholesterol; AST, aspartate transaminase; ALT, alanine transaminase; BUN, Blood Urea Nitrogen; ACR, urine albumin to creatinine ratio; MLA, Machine learning Algorithm.

A hundred male Wistar rats (150–170 g) were purchased from the Holding Company for Biological Products and Vaccines based in Giza, Egypt. Rats were randomized into 10 groups (N=10 per group) and kept one week for acclimation with free access to normal rat chow and water under well-controlled condition (20 ± 2°C), 12 h light/dark cycle. The animal procedures followed the guidelines outlined in the National Institutes of Health guide for the care and use of Laboratory Animals (8th edition, 2011). Ethical approval for the experiments was obtained from the Institutional Animal Ethics Committee of Ain Shams University, Faculty of Medicine NO. FWA000017585. The Diabetic rat model was induced by feeding rats with a high-fat diet (HFD) that consists of 58% fat, 17% carbohydrate, and 25% protein and libitum for a total of 12 weeks. After the initial 4 weeks, the rats were administered two low-dose intraperitoneal (i.p.) injections of streptozotocin (STZ; 30 mg/kg) dissolved in citrate buffer (pH 4.5), with a one-week interval between injections. The Normal control rats were administered citrate buffer only. After one week following the last STZ injection, blood samples were collected from the tail vein, and blood glucose levels were measured using a glucometer (Accu-check, Roche Diagnostics, Risch-Rotkreuz, Switzerland). The onset of diabetes was confirmed when the non-fasting blood glucose levels were equal to or higher than 200 mg/dl. Throughout the study, the rats were allowed to continue consuming their respective diets until its completion. Then, T2DM-induced rats were randomly divided into 9 groups, 10 rats each, including; (I) the T2DM control group (N=10): that received intraperitoneal STZ, fed HFD, and 0.9% saline orally, (II) Rosavin-10, and 30 groups: the T2DM induced rats received 10 mg/kg-30 mg/kg of rosavin, respectively dissolved in 0.9% saline for 4 weeks, (III) Z-Biotic 0.5, and Z-Biotic 1 groups: T2DM induced rats received 0.5 mg/kg- 1 mg/kg Z-Biotic, respectively dissolved in DMSO for 3 weeks. (IV) Isorhamnetin-10, and Isorhamnetin-40 groups: the T2DM induced rats received 10 mg/kg-40 mg/kg Isorhamnetin dissolved in DMSO for 3 weeks, (V) Caffeic acid groups: the T2DM induced rats received 10 mg/kg-50 mg/kg caffeic acid, respectively dissolved in cold water. Rats received the medicinal plant-based drugs and the probiotics orally by gastric gavage. (VI) The Normal group (N=10): rats received sodium citrate buffer 1 ml/kg intraperitoneally, the same amount injected in the weight-matched rat in the other groups. At the end of the experiment, blood samples were obtained from the retro-orbital veins of the animals under ether anesthesia. Subsequently, the animals were euthanized through cervical dislocation. Both the right and left gastrocnemius muscles, along with adipose tissue, were collected from all the animal groups. One gastrocnemius muscle and a portion of adipose tissue from each animal were frozen at -80°C for biochemical analysis. The other gastrocnemius muscle and a portion of adipose tissue were fixed in 10% formalin for histopathological evaluation (**Figure 1**).

### 2.3 Biochemical analysis

Prior to euthanization, blood samples were obtained from the optical vein of the rats and then subjected to centrifugation at 2000g for 10 minutes at 4°C. The resulting serum was collected for further analysis. Commercial ELISA kits from RayBiotech, USA, were utilized to measure the levels of cholesterol, triglycerides, high-density lipoprotein (HDL), low-density lipoprotein (LDL), alanine transaminase (ALT), aspartate transaminase (AST), total cholesterol (TC), serum creatinine, Blood Urea Nitrogen (BUN), fasting blood glucose, postprandial blood glucose, and fasting blood insulin following the manufacturer’s instructions. The calculation of the Homeostatic Model Assessment of Insulin Resistance (HOMA-IR) was performed using the equation: fasting insulin (μU/L) multiplied by fasting glucose (nmol/L)/22.5. Urine samples were collected for one day using individual metabolic cages, which took place on the day before the completion of the treatment. The level of albumin in the urine was evaluated using commercially available colorimetric kits from RayBiotech, USA, following the instructions provided by the supplier.

### 2.4 Histopathological analysis

Liver and adipose tissue specimens underwent dissection and fixation in neutral buffered formalin for 72 hours. Subsequently, the samples underwent a series of processing steps involving ethyl alcohol, Xylene clearance, and embedding in paraplast tissue-embedding media. Employing a rotatory microtome, tissue sections with a thickness of 5μm were acquired and affixed onto glass slides. The staining procedure utilized Hematoxylin and Eosin (H and E), following standard protocols outlined by Culling, C.F.A. 2013. Skilled histologists, operating in a blinded fashion, scrutinized these tissue sections.

### 2.5 Morphometric analysis

For the analysis of white fat cells and the determination of average cell diameter, a minimum of 6 non-overlapping fields were randomly selected and scanned. Following the approach outlined by Batts and Ludwig (47), individual biopsy specimens were assessed for the grade of inflammation, rated on a scale from 0 to 4 [0: no activity; 1: minimal; 2: mild; 3: moderate; and 4: severe]. All data were acquired through the Leica Application module linked to Leica Microsystems GmbH (Germany).

### 2.6 Bioinformatics analysis

- In this study, we conducted a comprehensive analysis of genes that are differentially expressed relevant to diabetes. To identify these genes, we performed a search in the Gene Expression Omnibus (GEO) database http://www.ncbi.nlm.nih.gov/geo, accessed on Jan 2024. In this study, we conducted a comprehensive analysis of genes that are differentially expressed relevant to diabetes. To identify these genes, we performed a search in the Gene Expression Omnibus (GEO) database http://www.ncbi.nlm.nih.gov/geo, accessed on Jan 2024. We utilized keywords related to diabetes mellitus type 2 such as ‘type 2 diabetes,’ ‘diabetic nephropathy,’ ‘tissue,’ ‘pancreas’. After obtaining a pool of potential datasets, we proceeded to screen them based on specific inclusion criteria. The first criterion was related to the tissue type, where we focused on tissues relevant to T2DM. The second inclusion criterion was the availability of normal tissues used as controls in the dataset. The inclusion of normal tissues ensures a suitable baseline for comparative analysis with tissues from T2DM patients. Furthermore, we also considered the sample size in the dataset. To ensure statistical robustness and reduce potential bias, we set a minimum threshold of ten or more samples in each dataset.

Specifically, we selected two datasets, GSE20966 and GSE142025, and used the “GEO2R/Limma R” package to screen for highly significant differentially expressed genes (DEGs). We considered genes with a p-value less than 0.05 and a log twofold change (LogFC) value of ≥ 1 or ≤ -1, using the Benjamini and Hochberg method for false discovery rate adjustment. In order to identify the biological pathways of DE-mRNAs, a thorough enrichment analysis was conducted using Enrichr (http://amp.pharm.mssm.edu/Enrichr, Jan 2022) with the Kyoto Encyclopedia of Genes and Genomes (KEGG) selected as the analysis tool. The analysis revealed The DE-mRNAs were related to Pancreatic secretion, ECM-receptor interaction, HNF3B pathway,RANKL regulation of apoptosis and immune response(**Supplementary Figure S2**). then filtered based on their association with, specific pathways that are of interest in diabetes research, such as insulin resistance, autophagy, cGAS/STING, and NOD-like receptor pathways(**Supplementary Figure S2**). To identify genes associated with these pathways, we utilized the GeneCards database https://www.genecards.org/, accessed on Jan 2024. Accordingly, we selected nine genes: Z-DNA Binding Protein 1 (*ZBP1*), Stimulator Of Interferon Response CGAMP Interactor 1 (*STING1*), RIG-I, Retinoic Acid-Inducible Gene 1 Protein (*DDX58*), mammalian target of rapamycin (*mTOR*), Nuclear Factor Kappa B Subunit 1 (*NFKB1*), insulin-like growth factor-1 (*IGF-1*), Component Of Inhibitor Of Nuclear Factor Kappa B Kinase Complex (*CHUK*), RAC-beta serine/threonine-protein kinase (*AKT2*), and Ret Proto-Oncogene (*RET*). To explore the interactions between the differentially expressed genes, we utilized the String database https://string-db.org/, accessed on Jan 2024 to construct a protein-protein interaction (PPI) network. Furthermore, to identify miRNAs that target the selected DEGs, we used the mirwalk database http://mirwalk.umm.uni-heidelberg.de/, accessed on Jan 2024. Finally, to predict the interaction of miRNAs-LncRNAs, we used RNA22 https://cm.jefferson.edu/rna22/, accessed on Jan 2024, mirwalk database http://mirwalk.umm.uni-heidelberg.de/, accessed on Jan 2024 and DIANA Tools https://diana.e-ce.uth.gr/lncbasev3/interactions, accessed on Jan 2024. Thus, the following LncRNAs (AC074117.2, RP11–773H22.4, and RP4–605O3.4) and miRNAs (miR-1976, miR-1, miR-611, and miR-3163) were chosen (**Supplementary Table S2**, **S3**, **Supplementary Figures S3**-**S8**).

### 2.7 Total RNA extraction and quantitative real-time PCR

Total RNA was isolated from the Liver (L) and Adipose tissue (A) samples using the miRNeasy kit (Qiagen, USA; Cat no. 74104). Next, the RNA quality, integrity, and concentration were measured using the DeNovix DS-11 microvolume spectrophotometer (Wilmington, USA) and stored at -80°C. The obtained RNA samples were reverse transcribed into cDNA by the two-step RT-PCR using the miScript II RT kit (Qiagen, USA; Cat no. 218161). Then the qRT-PCR was performed using Applied Biosystems Tm 7500 system (Foster City, California, United States). Regarding the expression of mRNAs, QuantiTect SYBR Green PCR Kit (Qiagen, Helman Germany; Cat no. 204143) was used. The relative expression of the miRNAs was obtained using the miScript SYBR Green PCR Kit (Qiagen, Helman Germany; Cat no. 218073). The relative expression of the lncRNAs was performed using RT^2^ SYBR Green ROX qPCR Master mix (Qiagen, Helman Germany; Cat no: 330500). Glyceraldehyde-3-phosphate dehydrogenase (*GAPDH*) and U6 were regarded as internal controls for the mRNAs, LncRNAs, and miRNAs, respectively. The Livak method RQ = 2^-ΔΔCt^ method was adopted to analyze the relative expression. For each sample, 2 replicates were set. Ct values of more than 35 were deemed negative. Melting curve analysis validated the amplicons’ specificities for SYBR Green-based PCR amplification. In this study, proper standardization procedures were used to detect any experimental error produced at any stage of the RNA extraction and processing according to MIQE recommendations. The PCR procedure was as follows: an initial activation phase at 95°C for 15 minutes was followed by 40 cycles of PCR at 94°C for 15 s, 55°C for 30 seconds, and 72°C for 30 s.

### 2.8 Statistical analysis

The Statistical analysis was performed with SPSS 26. Software. Data are presented as mean ± SD and significant differences were compared using a 1-way analysis of variance followed by *post hoc* Tukey’s test. The Shapiro-Wilk test confirmed that the data in this study followed a normal distribution. Categorical data were expressed as percentages and compared using the chi-square test.

## 3 Machine learning model

### 3.1 Data

**Figure 2:**
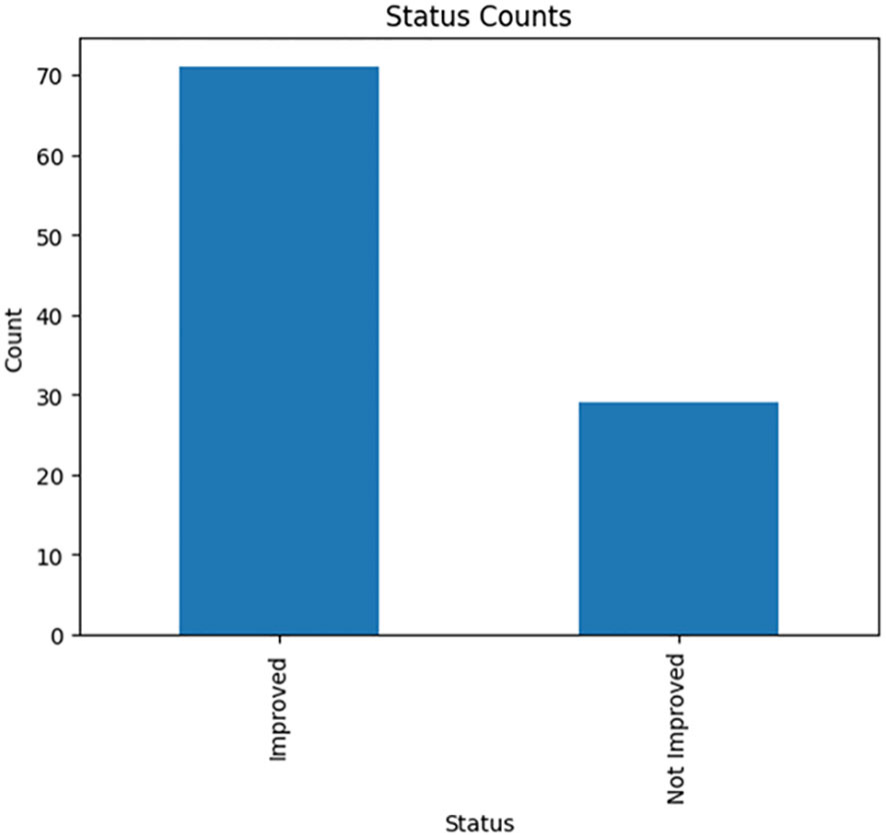
Show the number of improved (48) and not improved (29) samples.

One of the main objectives of this study is to create a predictive model using machine learning algorithms to thoroughly identify a promising set of features that strongly indicate the drug’s response to T2DM. The dataset in this study is a mouse model data with 100 samples distributed among normal, treated, and diseased groups (**Table 1**) with molecular features expressed in adipose tissues (A) and liver tissues (L) besides the biochemical features (**Table 2**). To determine treatment response, two key features were focused on: inflammation grade and fat cell diameter. Samples exhibiting a fat cell diameter exceeding 70 and an inflammation grade of 3 or higher were categorized as “not improved” (binary 0), while those falling below these thresholds were labeled as “improved” (binary 1). The analysis showed the relative ratio is 1:2.44 for the improved and not improved samples as shown in **Figure 2**.

**Table 1:** The number of samples per normal, disease model, and treatment groups.

Condition	Number of samples
Normal (Healthy)	10
T2DM	10
Rosavin-10	10
Rosavin-30	10
Z-Biotic 0.5	10
Z-Biotic 1	10
Isorhamnetin-10	10
Isorhamnetin-40	10
caffeic acid-10	10
caffeic acid-50	10

**Table 2:** Molecular and biochemical features used in ML models.

Molecular (32 features)	Biochemical (12 features)
1. ZBP1-mRNA (L) 2. ZBP1-mRNA (A) 3. STING1-mRNA (L) 4. STING1-mRNA (A) 5. DDX58 -mRNA (L) 6. DDX58 -mRNA (A) 7. mTOR -mRNA (L) 8. mTOR -mRNA (A) 9. NFKB1 -mRNA (L) 10. NFKB1 -mRNA (A) 11. IGF1R -mRNA (L) 12. IGF1R -mRNA (A) 13. CHUK -mRNA (L) 14. CHUK -mRNA (A) 15. AKT2 -mRNA (L) 16. AKT2 -mRNA (A) 17. RET -mRNA (L) 18. RET -mRNA (A) 19. miR-1976 (L) 20. miR-1976 (A) 21. miR-1 (L) 22. miR-1 (A) 23. miR-611 (L) 24. miR-611 (A) 25. miR-3163 (L) 26. miR-3163 (A) 27. AC074117.2 (L) 28. AC074117.2 (A) 29. RP11–773H22.4 (L) 30. RP11–773H22.4 (A) 31. RP4–605O3.4 (L) 32. RP4–605O3.4 (A)	1. Glucose 2. Insulin 3. HOMA-IR 4. Total cholesterol 5. Triglycerides 6. LDL-C 7. HDL-C 8. AST 9. ALT 10. Creatinine 11. BUN 12. ACR

(A), means expressed in the adipose tissues; (L), means expressed in liver tissues.

The dataset was divided into 70 samples for training and 30 samples for testing. Also, Multi-classifiers approaches were implemented using 5 classifiers including K-Nearest Neighbors (KNN), Light Gradient Boosting Machine (LGBM), Random Forest (RF), Logistic Regression (LR), and Ada Boost Classifier.

### 3.2 Dataset preprocessing

In the development of machine learning models, data preprocessing stands as a critical step. Herein, thorough measures were taken to ensure the data’s quality by addressing several factors and they were null feature removal, noise reduction, and outlier elimination. Additionally, the column index was reset to accurately assign each column its respective index. Moreover, the creation of a ‘status’ column in binary format (1,0) was undertaken to reflect treatment response whether it was improved or not improved (49).

### 3.3 Feature selection

The feature selection technique was utilized, this method effectively reduces the complexity and size of the data, enhancing the learning process. Additionally, by selecting only the relevant features, the model becomes faster and more precise, thereby enhancing its predictive capabilities through noise reduction.

#### 3.3.1 Selecting top features with recursive feature elimination cross validation

An initial trial was carried out to determine the selected machine learning features for predicting T2DM drug response. The Recursive Feature Elimination Cross-Validation (RFECV) technique was employed for this purpose. RFECV functions by iteratively eliminating features and evaluating prediction accuracy. The most optimal reduced set of features, which either matches or surpasses the original accuracy of the complete feature set, is then identified as the optimal set of predictive features. Various combinations of features yield different accuracy levels (50). Furthermore, relevant model hyperparameters for the RF model (e.g., maximum tree depth, minimum samples split, and estimators) were fine-tuned to achieve the best-performing model. Optimization involves experimenting with different parameter sets to determine the most effective configuration for our dataset. The top selected features for molecular, biochemical, and both combined were reported.

### 3.4 Cross validation

In k-fold cross-validation, the training set is partitioned into k distinct and equally-sized subsets. The classifier is trained on each subset using the union of all other subsets. Consequently, the average error rate across all subsets provides an estimate of the classifier’s error rate. Each data point is included in the test set precisely once and appears in the training set k-1 times. Increasing k helps reduce the variance of the resulting estimate. We utilized the k-fold cross-validation method, employing k=3 in our analysis (51).Of note, we utilized Stratified K-Fold cross-validation with a number of folds set to 3 to validate the performance of our machine learning model. This technique was chosen to ensure that each fold preserves the proportion of samples for each class, thereby mitigating potential biases and enhancing the reliability of our results. During the validation process, we partitioned the dataset into three subsets, with each subset containing a representative distribution of the target variable classes. We then iteratively trained the model on two-thirds of the data (training set) and evaluated its performance on the remaining one-third (validation set), ensuring that the model was tested on unseen data in each fold. By adopting Stratified K-Fold cross-validation, we aimed to demonstrate the independence and rigor of our model validation, thereby enhancing the reproducibility and accuracy of our results.

### 3.5 Models predictions

A multi-class approach using five machine learning algorithms (KNN, RF, LGBM, LR, and Ada Boost) was employed on three separate models as follows: one using only Molecular features, one using only Biochemical features, and one combining both feature groups as shown in **Table 3**. The top selected features obtained from RFECV were used in each model. This comprehensive strategy aimed to identify a potential set of highly predictive features for drug response. Also, the top-performing classifier applied on the training set for each feature group was selected and then applied to the testing set to evaluate their performance on an unseen dataset.

**Table 3:** The three predictive models were applied to the five classifiers.

Model	Data Type
1	Molecular
2	Biochemical
3	Molecular + Biochemical

### 3.6 Machine learning evaluation

The three model’s performance were assessed using the test dataset. The evaluation was based on key performance metrics, such as the area under the curve (AUC). To further analyze the model’s efficacy, a 2 × 2 confusion matrix was constructed using the test dataset, allowing for the calculation of true positive, false positive, true negative, and false negative values.

### 3.7 Visual analysis for molecular and biochemical features

a graphical implementation has been used to analyze the relationship between each molecular and biochemical features of treated samples against the T2DM group as well, as to the normal group to capture insights for drug response.

### 3.8 Python packages

This study’s data were processed using Python 3.7 as the programming language. We used many Python-based packages and modules as well to ease the processing pipeline. The ‘pandas’ package (version 1.3.5) and ‘NumPy’ (version 1.20.3) are utilized for data manipulation and analysis. ‘Seaborn’ (version 0.13. 2) enhances data visualization capabilities. ‘Matplotlib.pyplot’ (version 3.5.0) is another data visualization package, offering a versatile toolkit for creating static, interactive, and animated plots. The scikit-learn (version 1.0.2) is extensively used for machine learning tasks. ‘statistics’ provides functions for mathematical statistics.

## 4 Results

### 4.1 Effect on diabetic parameters, serum biochemicals, and liver inflammation

**Table 4** presents the observed changes in different parameters in the T2DM group compared to the normal group, along with the effects of medicinal plant-based drugs and probiotics drug administration. In the T2DM group, there was a significant increase in serum glucose, insulin, and HOMAIR levels. However, these elevated levels were modulated after the administration of our selected drugs, particularly at higher doses, suggesting that the drugs have the potential to suppress hyperinsulinemia and improve insulin resistance. Furthermore, renal function biomarkers including serum creatinine, BUN, and albumin-to-creatinine ratio (ACR) showed a significant increase in the T2DM group. On the other hand, the administration of different doses of drugs led to a modulation of these biomarkers. The lipid profile analysis revealed a significant increase in total cholesterol, triglycerides, and LDL levels, along with reduced HDL levels in the T2DM rat group. While the treated groups exhibited improvements in the lipid profile levels. Moreover, hepatic damage biomarkers such as AST and ALT were elevated in the T2DM group but decreased in the treated groups. Similarly, liver inflammation grades exhibited an increase in the T2DM group but decreased in the treated groups, particularly at higher doses. The fat cells’ diameter increased significantly in the T2DM group as well as the treated groups except for the Rosavin-30 treated group which showed decreasing in the fat cells’ diameter.

**Table 4:** Serum biochemical parameters, liver inflammation in the normal, T2DM, and treated rats.

	Normal	T2DM	Rosavin-10	Rosavin-30	Z-Biotic 0.5	Z-biotic 1	Isorhamnetin-10	Isorhamnetin-40	caffeic acid-10	caffeic acid-50	P-value	F
**Glucose(mmol/L)**	5.8 ± .36	29.07 ± 2.4^a^	12.51 ± 0.76^ab^	6.87 ± .467^b^	11.754.8 ± 12.91^ab^	5.63 ± .38^b^	12.73 ± 4.54^ab^	7.08 ± 0.54^b^	15 ± .9^ab^	7.63 ± 1^ab^	1.62E-73	519.032
**Insulin(μU/ml)**	4.64 ± 0.91	16.29 ± 1.14^a^	13.78 ± 0.92^ab^	5.76 ± 0.97^b^	12.96 ± 0.87^ab^	4.72 ± 0.8^b^	14.18 ± 0.92^ab^	5.36 ± 0.97^b^	14.06 ± 0.94^ab^	5.87 ± 0.99^b^	2.76E-60	258.422
**HOMAIR**	1.2 ± 0.24	21.06 ± 2.18^a^	7.69 ± 0.85^ab^	1.77 ± 0.36^b^	6.79 ± 0.75^ab^	1.19 ± 0.24^b^	8.02 ± 0.57^ab^	1.68 ± 0.28^b^	9.4 ± 1.03^ab^	2 ± 0.41^b^	3.64E-72	483.638
**Total cholesterol(mmol/L)**	1.86 ± 0.14	10.44 ± 1.25^a^	6.07 ± 0.59^ab^	3.08 ± 0.58^ab^	5.67 ± 0.55^ab^	2.2 ± 0.42^b^	9.1 ± 0.89^ab^	2.16 ± 0.41^b^	7.89 ± 0.77^ab^	3.7 ± 0.7^ab^	1.12E-55	201.870
**Triglycerides(mmol/L)**	1.73 ± 0.22	17.11 ± 0.95^a^	8.49 ± 0.64^ab^	4.03 ± 0.34^ab^	9.34 ± 0.71^ab^	4.43 ± 0.37^ab^	7.72 ± 0.58^ab^	1.55 ± 0.13^b^	5.94 ± 0.45^ab^	2.82 ± 0.24^ab^	7.47E-82	800.876
**LDLC(mmol/L)**	0.51 ± 0.04	6.83 ± 1.03^a^	3.19 ± 0.46^ab^	1.38 ± 0.33^ab^	2.66 ± 0.38^ab^	1.15 ± 0.28^b^	4.46 ± 0.64^ab^	1.93 ± 0.46^ab^	5.28 ± 0.76^ab^	2.15 ± 0.52^ab^	8.54E-48	131.283
**HDLC(mmol/L)**	1.68 ± 0.24	0.73 ± 0.18^a^	0.91 ± 0.06^a^	1.38 ± 0.23^ab^	1.14 ± 0.07^ab^	1.72 ± 0.28^b^	0.8 ± 0.05^a^	1.21 ± 0.2^ab^	1 ± 0.06^ab^	1.52 ± 0.25^b^	1.2943E-26	37.205
**AST(IU/L)**	19.3 ± 3.13	152 ± 11.32^a^	83.5 ± 8.24^ab^	30.4 ± 3.24^ab^	68.5 ± 8.24^ab^	25.6 ± 3.06^b^	96.3 ± 4.97^ab^	40.2 ± 4.43^ab^	98.5 ± 8.24^ab^	42.4 ± 3.24^ab^	8.18E-70	427.572
**ALT(IU/L)**	11.5 ± 2.17	152.4 ± 19.77^a^	79.5 ± 6.1^ab^	18.1 ± 3.54^b^	56.8 ± 4.29^ab^	12.9 ± 2.69^b^	72.2 ± 5.49^b^	16.5 ± 3.17^b^	95.4 ± 7.37^ab^	21.7 ± 4.19^b^	2.88E-67	374.018
**Creatinine((mmol/L))**	5924 ± 44.2	345.7 ± 39.79^a^	186.5 ± 12.38^ab^	68.79 ± 5.31^b^	150.3 ± 9.73^ab^	55.7 ± 4.42^b^	237.8 ± 17.6^ab^	96.83 ± 7.07^ab^	231.62 ± 15.03^ab^	8.88 ± 0.07^ab^	3.63E-68	392.145
**BUN(mmol/L)**	57.09 ± 2.04	169.65 ± 17.99^a^	91.3 ± 7.89^ab^	61.09 ± 2.46^b^	73.63 ± 6.3^ab^	49.27 ± 1.988^b^	127.83 ± 10.99^ab^	85.53 ± 3.4^ab^	113.22 ± 9.79^ab^	75.75 ± 0.19 ^ab^	7.76E-56	203.614
**Urine ACR(mg/mmol)**	1.9 ± 0.12	9.7 ± 1.04^ab^	5.8 ± .4^ab^	2.33 ± 0.33^ab^	4.69 ± 0.33^ab^	1.91 ± 0.27^b^	8.14 ± 0.63^ab^	3.25 ± 0.47^ab^	7.21 ± 0.49^a^	2.88 ± 0.41^ab^	3.6853E-65	334.688
**Fat cells diameter**	36.3 ± 3.5	82.2 ± 3.61^a^	71.3 ± 2.95^ab^	40.6 ± 2.41^b^	68.6 ± 2.95^ab^	46.7 ± 4.45 ^ab^	68.9 ± 2.42 ^ab^	45.2 ± 4.29 ^ab^	68.5 ± 3.6 ^ab^	42.89 ± 3.07 ^ab^	3.6426E-58	230.744
**Liver inflammation**											9.82E-25	
**0**	10(100%)	0	0	0	0	3(30%)	0	0	0	4(40%)		
**1**	0	0	0	8(80%)	0	7(70%)	0	4(40%)	0	6(60%)		
**2**	0	0	6(60%)	2(20%)	6(60%)	0	2(20%)	6(60%)	7(70%)	0		
**3**	0	5(50%)	4(40%)	0	4(40%)	0	8(80%)	0	3(30%)	0		
**4**	0	5(50%)	0	0	0	0	0	0	0	0		

Data represented as mean ± SD or N(%), the statistical significance between groups was calculated using the ANOVA-Tukey post hoc test for numerical data and the Chi-square test for the categorical data where ‘a’ represents statistical significance when compared to the normal group, and ‘b’ represents statistical significance when compared to the T2DM group.

### 4.2 Histopathological results

**Figure 3:**
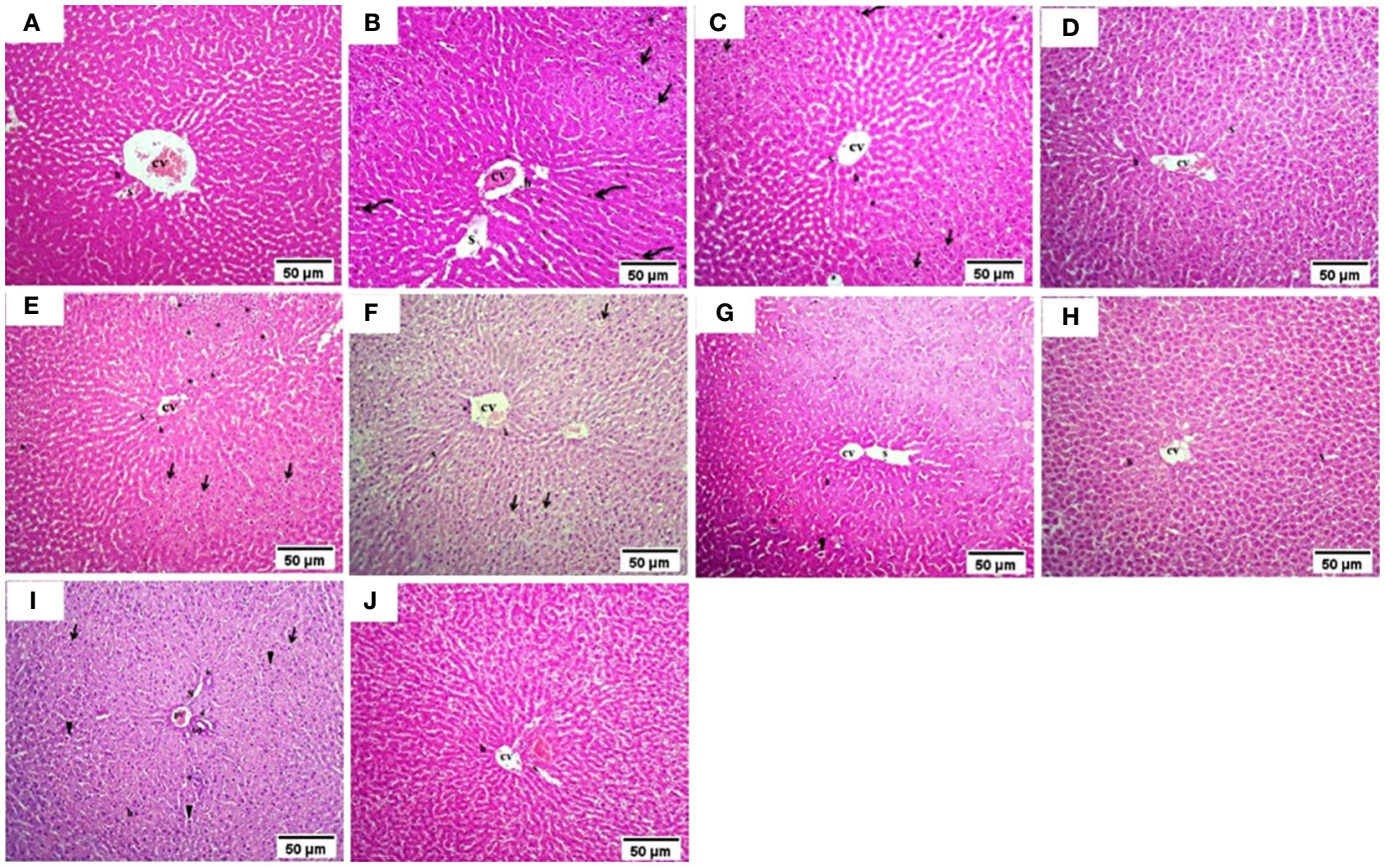
Histopathology examination of liver tissue in experimental animals. **(A)** the Normal group, **(B)** the T2DM group, **(C)** Rosavin-10 group, **(D)** Rosavin-30, **(E)** Z-Biotic 0.5, **(F)** Z-Biotic 1, **(G)** Isorhamnetin-10, **(H)** Isorhamnetin-40, **(I)** Caffeic acid-10, **(J)** Caffeic acid-50. Microvesicular steatosis (Arrow), Inflammatory cells infiltrate (asterisks), Necrotic cells (Arrowhead), Pyknotic hepatocytes (Curved arrow), Sinusoids (S), Central vein (CV), Portal vein (PV), Bile duct (BD). Hepatocytes (h) (10X magnifications).

**Figure 4:**
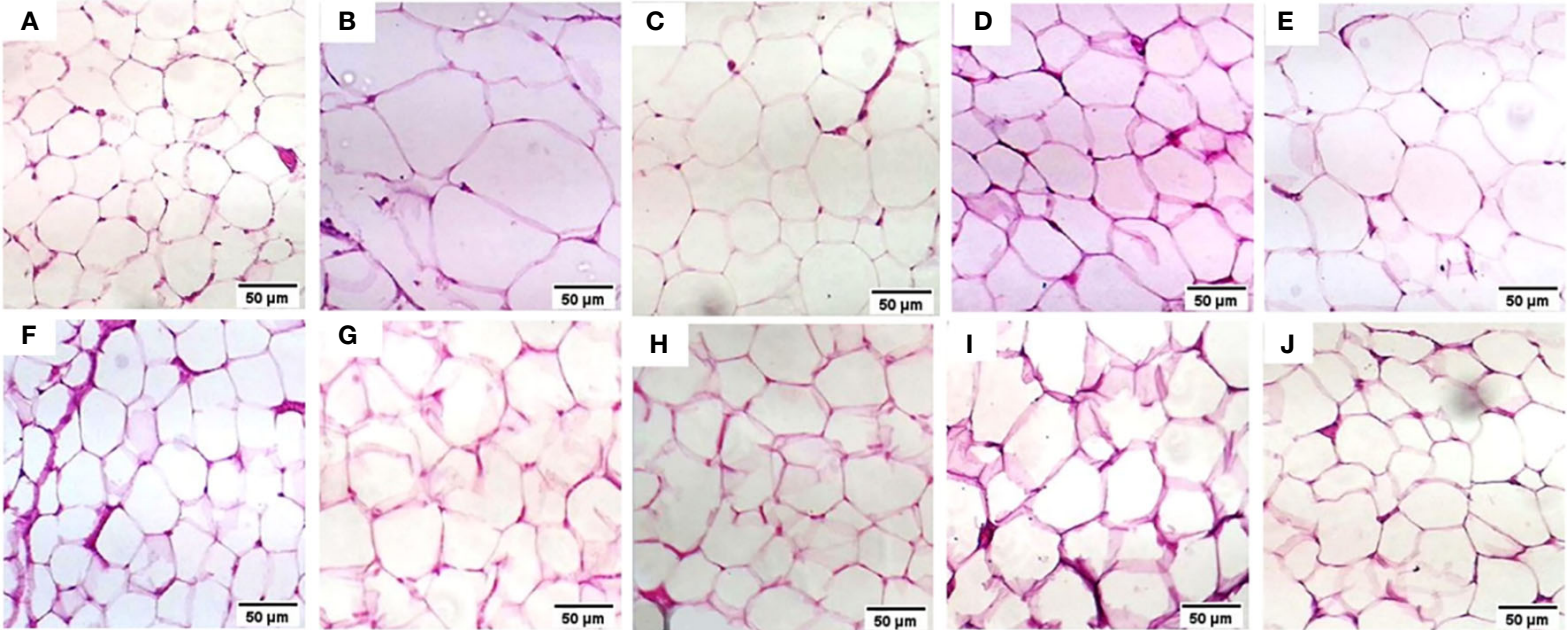
Histopathology examination of adipose tissue in the experimental animals. **(A)** the Normal group, **(B)** the T2DM group, **(C)** the Rosavin-10 group, **(D)** Rosavin-30, **(E)** Z-Biotic 0.5, **(F)** Z-Biotic 1, **(G)** Isorhamnetin-10, **(H)** Isorhamnetin-40, **(I)** Caffeic acid-10, **(J)** Caffeic acid-50. (40X magnifications).

The normal group demonstrated a normal architecture. The hepatic lobule is composed of hepatocytes (h) that are arranged into branching cords with sinusoids (S) in between. Hepatocytes appeared as polygonal cells with granular eosinophilic cytoplasm and round nuclei. Central vein (CV) is located at the center of the lobule whereas the T2DM group showed an altered architecture with marked hepatocellular microvesicular steatosis (Arrow) all over hepatic lobule accompanied, widespread of necrotic cells (Arrowhead), inflammatory cells infiltrate (asterisks) and pyknotic cells (Curved arrow). The Rosavin-10 treated group showed altered architecture with mild hepatocellular microvesicular steatosis (Arrow), in addition to the presence of pyknotic cells (Curved arrow), necrotic cells (Arrowhead), and inflammatory cells (asterisks) whereas the Rosavin-30 treated group showed a normal histological feature of the hepatic lobule with wide sinusoids (S). The Z-Biotic 0.5 treated group showed an altered architecture of the liver with moderate hepatocellular microvesicular steatosis (Arrow), and the presence of inflammatory cell infiltrates (asterisks) whereas the Z-Biotic 1 treated group showed an altered architecture of the liver with mild hepatocellular microvesicular steatosis (Arrow) with minimum mononuclear inflammatory cells infiltrates. The Isorhamnetin-10 treated group demonstrated necrotic changes (Arrowhead) in the hepatocytes, several hepatocytes showed pale cytoplasm with vacuolated nuclei. Inflammatory cells (asterisks) are scattered in the hepatic lobule whereas the Isorhamnetin-40 treated group demonstrated a slightly normal histological feature. The Caffeic acid-10 treated group showed an altered architecture with hepatocellular microvesicular steatosis (Arrow), scattered necrotic cells (Arrowhead), and inflammatory cell infiltrates (asterisks) whereas the Caffeic acid-50 treated group demonstrated a slightly normal histological feature (**Figure 3**). Analysis of adipose tissue revealed a significant increase in fat cell diameter in T2DM animals. However, treatment of the diabetic animals with rosavin, Z-biotic, isorhamnetin, or Caffeic acid exhibited notable decreases in epididymal adipocyte cell size compared to the normal group. Interestingly, the most pronounced ameliorative effect was observed in the high-dose-treated groups (**Figure 4**).

### 4.3 Effect on molecular targets mRNAs-miRNAs-LncRNAs

The results presented in **Table 5** demonstrate significant changes in various biomarkers among the different experimental groups expressed in liver tissues (L) and adipose tissues (A). In comparison to the normal group, the T2DM group exhibited markedly elevated levels of *ZBP1, STING1, DDX58, mTOR, NFKB1, IGF1R, CHUK, RET*, hsa-miR-1976, hsa-miR-611, and lnc-RP11–773H22.4 (p < 0.05) while *AKT2*, hsa-miR-1, hsa-miR-3163, lnc-AC074117.2, and lnc-RP4–605O3.4 were decreased. However, after drug administration, particularly at higher doses, this disturbance was significantly modulated.

**Table 5:** The differential expression of mRNAs-miRNAs-LncRNAs in liver tissues (L) and adipose tissues (A) among different animal groups, including a normal group, T2DM group, and various treatment groups with Rosavin, Z-Biotic, Isorhamnetin, and caffeic acid.

	Normal	T2DM	Rosavin-10	Rosavin-30	Z-Biotic 0.5	Z-Biotic 1	Isorhamnetin-10	Isorhamnetin-40	caffeic acid-10	caffeic acid-50	P-value	F
*ZBP1* (L)	1.84 ± 0.31	170.13 ± 10.61^a^	92.33 ± 3.96^ab^	25.2 ± 3.42 ^ab^	71.03 ± 3.04 ^ab^	14.82 ± 2.01 ^ab^	46.17 ± 1.98 ^ab^	10.08 ± 1.37 ^ab^	61.56 ± 2.64 ^ab^	14 ± 1.9 ^ab^	8.39E-95	1563.68
*ZBP1* (A)	1.43 ± 0.2	20.97 ± 1.98^a^	4.71 ± 0.64^ab^	1.39 ± 0.24^b^	9.42 ± 1.28^ab^	2.64 ± 0.45^b^	4.06 ± 0.55^ab^	1.20 ± 0.21^b^	7.07 ± 0.96^ab^	2.09 ± 0.36^b^	5.5293E-72	479.054
*STING1*(L)	1.44 ± 0.2	7.7 ± 0.82^a^	2.04 ± 0.16^ab^	0.34 ± 0.03^ab^	4.09 ± 0.33^ab^	0.64 ± 0.06^ab^	1.76 ± 0.14^b^	0.29 ± 0.03^ab^	3.07 ± 0.24^ab^	0.5 ± 0.05^ab^	2.74E-75	569.3
*STING1*(A)	1 ± 0.05	14.64 ± 1.72^a^	5.59 ± 0.54^ab^	1.96 ± 0.11^b^	8.38 ± 0.8^ab^	2.55 ± 0.14^ab^	3.29 ± 0.31^ab^	1.16 ± 0.06^b^	9.5 ± 0.91^ab^	3.34 ± 0.18^ab^	1.4297E-68	400.573
*DDX58* (L)	1.03 ± 0.09	50.49 ± 5.48^a^	13.29 ± 2.16^ab^	0.95 ± 0.17^b^	19.94 ± 3.25^ab^	1.23 ± 0.23^b^	7.82 ± 1.27^ab^	0.56 ± 0.1^b^	22.6 ± 3.68^ab^	1.61 ± 0.3^b^	2.52E-69	416.732
*DDX58* (A)	1.17 ± 0.22	120.16 ± 11.65^a^	55.77 ± 6.87^ab^	3.6 ± 0.6^b^	83.65 ± 10.3^ab^	4.67 ± 0.78^b^	32.8 ± 4.04^ab^	2.12 ± 0.35^b^	94.81 ± 11.68^ab^	6.11 ± 1.02^b^	3.9696E-71	458.061
*mTOR* (L)	1.68 ± 0.28	82.8 ± 6.05^a^	29.72 ± 4.22^ab^	5.21 ± 0.55^b^	14.86 ± 2.11^ab^	2.41 ± 0.25^b^	35.67 ± 5.06^ab^	6.78 ± 0.71^b^	24.77 ± 3.52^ab^	4 ± 0.42^b^	1.84E-77	637.509
*mTOR* (A)	0.95 ± 0.09	4.46 ± 0.65^a^	1.21 ± 0.14^b^	0.1 ± 0.02^ab^	2.42 ± 0.28^ab^	0.2 ± 0.03^ab^	1.04 ± 0.12^b^	0.09 ± 0.01^ab^	1.81 ± 0.21^ab^	0.16 ± 0.03^ab^	2.3057E-64	320.894
*NFKB1* (L)	1.05 ± 0.12	50.92 ± 5.5^a^	22.3 ± 2.38^ab^	8.44 ± 1.75^ab^	12.63 ± 1.93^ab^	2.83 ± 1.93^b^	8.45 ± 0.52^ab^	0.65 ± 0.07^b^	17.5 ± 1.59^ab^	2.27 ± 0.21^b^	8.36E-73	500.059
*NFKB1* (A)	1.04 ± 0.1	41.56 ± 6.33^a^	13.7 ± 1.71^ab^	4.96 ± 1.03^ab^	9.03 ± 0.84^ab^	2.91 ± 0.27^b^	23.78 ± 2.54^ab^	2.92 ± 0.61^b^	10.96 ± 1.37^ab^	1.03 ± 0.12^b^	1.8857E-63	305.761
*IGF1R* (L)	1.2 ± 0.21	62.26 ± 5.46^a^	35.68 ± 3.81^ab^	11.72 ± 1.66^ab^	11.73 ± 3.82^ab^	5.2 ± 0.84^ab^	12.54 ± 1.46^ab^	2.79 ± 0.45^b^	3.83 ± 0.24^b^	0.38 ± 0.04^b^	4.61E-76	592.74
*IGF1R* (A)	1.05 ± 0.08	75.72 ± 8.2^a^	20.27 ± 2.7^ab^	1.79 ± 0.29^b^	30.4 ± 4.05^ab^	2.32 ± 0.38^b^	11.92 ± 1.59^ab^	1.05 ± 0.17^b^	34.45 ± 4.59^ab^	3.03 ± 0.5^b^	1.565E-72	492.988
*CHUK* (L)	1.17 ± 0.17	38.05 ± 4.99^ab^	14.36 ± 1.72^ab^	3.25 ± 0.68^b^	8.62 ± 1.02^ab^	2.62 ± 0.31^b^	2.63 ± 0.25^b^	0.48 ± 0.06^b^	4.21 ± 0.51^ab^	0.21 ± 0.02^b^	1.04E-70	448.166
*CHUK* (A)	0.99 ± 0.07	82.2 ± 9.38^a^	27.23 ± 1.72^ab^	6.18 ± 0.74^b^	10.11 ± 1.29^ab^	4.65 ± 1.11^b^	54.56 ± 6.55^ab^	9.62 ± 1.15^ab^	23.03 ± 4.8^ab^	0.75 ± 0.08^b^	1.6302E-70	443.572
*AKT2*(L)	1.04 ± 0.11	0.08 ± 0.01	4.37 ± 0.64^b^	15.16 ± 3.44^ab^	3.25 ± 0.42	7.37 ± 1.18^a^	3.59 ± 0.41	9.5 ± 1.52^ab^	15.77 ± 2.97^ab^	67.11 ± 7.45^ab^	2.16E-72	489.394
*AKT2*(A)	1.57 ± 0.36	0.21 ± 0.02	11.49 ± 1.78^ab^	40.75 ± 2.99^ab^	10.9 ± 1.67^ab^	19.98 ± 2.13^ab^	4.54 ± 0.43	48.82 ± 5.86^ab^	23.89 ± 2.41^ab^	147.88 ± 10.15^ab^	1.2722E-89	1197.044
*RET* (L)	1 ± 0.09	59.31 ± 3.97 ^a^	38.72 ± 1.82 ^ab^	8.9 ± 1.18 ^ab^	15.39 ± 2.41 ^ab^	3.08 ± 0.48^b^	28.07 ± 2.24 ^ab^	6.87 ± 1.08 ^ab^	3.98 ± 0.62 ^ab^	0.8 ± 0.12 ^b^	6.81E-89	1152.837
*RET* (A)	1.07 ± 0.1	71.19 ± 5.46^a^	52.71 ± 2.87^ab^	11.62 ± 1.13^ab^	12.82 ± 2.01^ab^	2.56 ± 0.4^b^	36.81 ± 2.66^ab^	5.73 ± 0.9^ab^	3.32 ± 0.52^b^	0.24 ± 0.04^b^	7.5164E-90	1211.250
hsa-miR-1976(L)	1.21 ± 0.16	106.28 ± 10.14^a^	62.55 ± 7.11^ab^	12.81 ± 2.01^ab^	40.58 ± 3.25^ab^	9.08 ± 0.99^ab^	8.24 ± 1.03^ab^	3.95 ± 0.37^b^	88.12 ± 7.18^ab^	20.51 ± 1.97^ab^	2.27E-77	634.5
hsa-miR-1976(A)	0.88 ± 0.08	13.85 ± 2.06^a^	2.77 ± 0.31^ab^	0.7 ± 0.14^b^	1.34 ± 0.15^b^	0.3 ± 0.06^b^	5.52 ± 1.15^ab^	1.13 ± 0.12^b^	5.72 ± 0.63^ab^	1.57 ± 0.19^b^	8.2892E-62	280.236
hsa-miR-1(L)	1.06 ± 0.11	0.06 ± 0.01	2.94 ± 0.43^b^	10.19 ± 2.31^ab^	2.19 ± 0.28	4.95 ± 0.79^ab^	2.42 ± 0.27	6.39 ± 1.02^ab^	10.6 ± 1.99^ab^	45.13 ± 5.01^ab^	2.55E-72	487.556
hsa-miR-1(A)	1.61 ± 0.38	0.16 ± 0.02	7.73 ± 1.2^ab^	27.4 ± 2.01^ab^	7.33 ± 1.13^ab^	13.44 ± 1.43^ab^	3.05 ± 0.29	32.83 ± 3.94^ab^	16.07 ± 1.62^ab^	99.45 ± 6.82^ab^	1.5192E-89	1192.289
hsa-miR-611(L)	1.14 ± 0.16	47.56 ± 6.23^a^	32.34 ± 3.71^ab^	11.58 ± 1.53^ab^	20 ± 3.13^ab^	4 ± 0.63^b^	36.5 ± 2.91^ab^	8.94 ± 1.4^ab^	5.17 ± 0.81^ab^	1.03 ± 0.16^b^	1.66E-66	359.336
hsa-miR-611(A)	0.92 ± 0.13	98.7 ± 8.79^a^	68.52 ± 3.73^ab^	15.11 ± 1.47^ab^	16.67 ± 2.61^ab^	3.33 ± 0.52^b^	47.85 ± 3.46^ab^	7.45 ± 1.17^ab^	4.31 ± 0.67^b^	0.32 ± 0.05^b^	2.346E-86	1011.174
hsa-miR-3163(L)	1.39 ± 0.22	0.15 ± 0.02	10.13 ± 1.16^ab^	75.2 ± 6.89^ab^	2.86 ± 0.33	9.15 ± 1.59^ab^	6.13 ± 0.7^b^	55.57 ± 8.21^ab^	1.91 ± 0.4	12.44 ± 1.44^ab^	8.24E-75	555.277
hsa-miR-3163(A)	1.23 ± 0.19	0.09 ± 0.01	0.68 ± 0.07	2.21 ± 0.49	7.26 ± 0.94^b^	18.58 ± 2.36^ab^	12.04 ± 1.38^ab^	139.5 ± 13.89^ab^	3.37 ± 0.7	49.84 ± 5.22^ab^	2.127E-82	823.840
lnc-AC074117.2(L)	1.63 ± 0.36	0.18 ± 0.03	0.93 ± 0.12	4.08 ± 0.76^b^	11.6 ± 1.6^ab^	47.35 ± 3.8^ab^	1.9 ± 0.22	9.82 ± 1.56^ab^	8.09 ± 0.86^ab^	29.36 ± 2.78^ab^	3.28E-82	815.841
lnc-AC074117.2(A)	1.44 ± 0.27	0.14 ± 0.03	1.89 ± 0.3	6.28 ± 1^ab^	30.6 ± 1.87^ab^	99.56 ± 5.14^ab^	2.47 ± 0.23	26.8 ± 3.22^ab^	4.43 ± 0.6^b^	15.81 ± 1.86^ab^	6.056E-100	2037.489
lnc-RP11–773H22.4(L)	1.01 ± 0.09	49.84 ± 4^a^	18.12 ± 2.5^ab^	3.34 ± 0.65^b^	20.34 ± 3.31^ab^	3.67 ± 0.61^b^	13.56 ± 2.21^ab^	1.25 ± 0.23^b^	7.98 ± 1.3^ab^	0.57 ± 0.1^b^	3.57E-75	565.887
lnc-RP11–773H22.4(A)	1.26 ± 0.15	91.85 ± 6.94^a^	40.81 ± 2.5^ab^	11.13 ± 0.88^ab^	35.22 ± 4.23^ab^	1.01 ± 0.19^b^	61.41 ± 0.63^ab^	2.26 ± 0.38^b^	59.67 ± 7.35^ab^	5.1 ± 0.85^b^	4.5705E-73	506.957
lnc-RP4–605O3.4(L)	0.99 ± 0.1	0.09 ± 0.01	2.18 ± 0.32^b^	7.58 ± 1.72^ab^	1.63 ± 0.21	3.68 ± 0.59^a^	1.8 ± 0.2	4.75 ± 0.76^ab^	7.88 ± 1.48^ab^	33.56 ± 3.73^ab^	2.94E-72	485.973
lnc-RP4–605O3.4(A)	1.5 ± 0.34	0.23 ± 0.02	5.75 ± 0.89^ab^	20.37 ± 1.5^ab^	5.45 ± 0.84^ab^	9.99 ± 1.06^ab^	2.27 ± 0.21	24.41 ± 2.93^ab^	11.95 ± 1.21^ab^	73.94 ± 5.07^ab^	1.7806E-89	1188.052

Data represented as mean ± SD, the statistical significance between groups was calculated using ANOVA-Tukey post hoc test where ‘a’ represents statistical significance when compared to the normal group, and ‘b’ represents statistical significance when compared to the T2DM group.

### 4.5 ML Analysis

#### 4.5.1 Feature selection using RFECV-based Random Forest for T2DM drug response prediction

**Figure 5:**
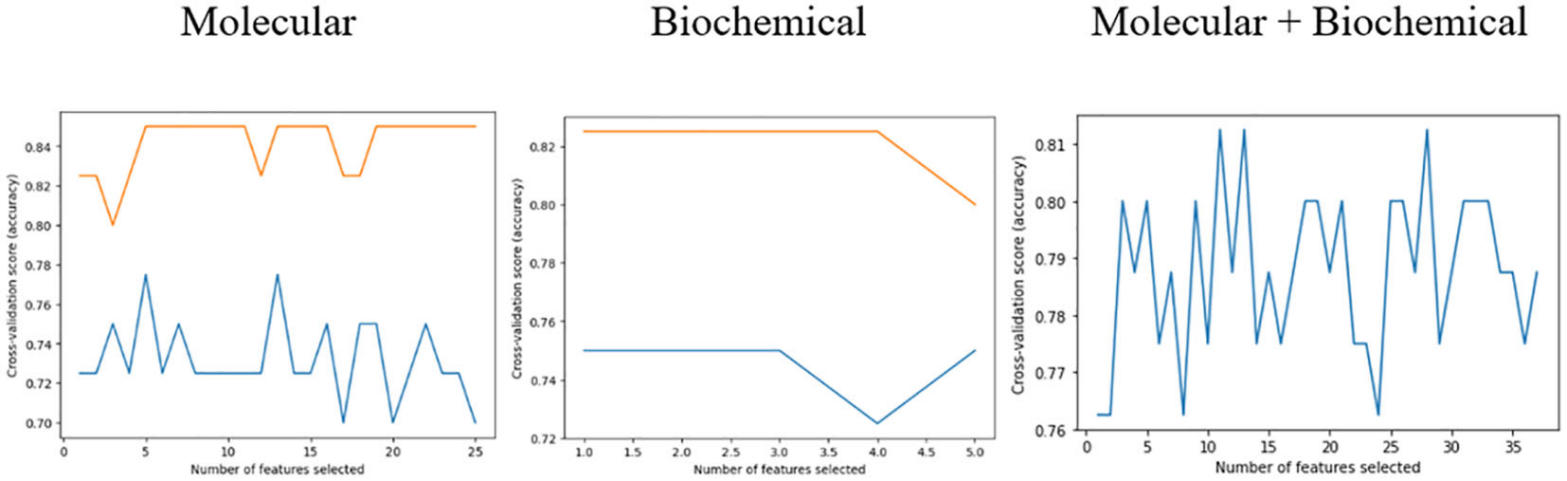
Number and accuracy scores for each feature set and both of them combined.

The feature selection procedure employing RFECV yielded 13 features out of 32 for the molecular model, 10 out of 12 for the biochemical model, and 20 out of 44 for the combined model. These selections, as shown in **Table 6**, were made while maintaining the same prediction accuracy level as shown in **Figure 5**. Molecular accuracy achieved 80% while the biochemical and both of them combined achieved 85%.

**Table 6:** Show the top selected features by RFECV for each model.

Model	Included Features
	Feature
**Molecular** Included: 13 Excluded: 19 Total: 32	ZBP1-mRNA (A) STING1-mRNA (L) DDX58 -mRNA (L) mTOR -mRNA (L) NFKB1 -mRNA (A) CHUK -mRNA (A) RET -mRNA (L) RET -mRNA (A) miR-1976 (A) miR-611 (L) miR-611 (A) RP11–773H22.4 (L) RP11–773H22.4 (A)
**Biochemical** Included: 10 Excluded: 2 Total: 12	Glucose Insulin HOMA-IR Total cholesterol Triglycerides AST ALT Creatinine BUN ACR
**Combined** Included: 20 Excluded: 24 Total: 44	STING1-mRNA (L) mTOR -mRNA (L) NFKB1 -mRNA (A) CHUK -mRNA (A) RET -mRNA (L) RET -mRNA (A) miR-1976 (A) miR-611 (L) miR-611 (A) RP11–773H22.4 (L) RP11–773H22.4 (A) Insulin Total cholesterol Triglycerides Glucose AST ALT Creatinine BUN ACR

#### 4.5.2 Model prediction results

Initial results applied on the training set showed the top-performing classifiers for each feature group. **Table 7** summarizes the accuracy of the adopted classifiers for each feature group. Notably, the KNN was the top classifier for the molecular model and the combined model while LGBM Classifier performed the best for the biochemical model.

**Table 7:** The evaluation metric for the top-performing classifier on the training set.

Model (Molecular)	Accuracy	AUC	Recall	Precision	F1-Score
**KNN**	0.8714	0.9177	0.9387	0.8974	0.9137
**Random Forest**	0.8424	0.9051	0.9007	0.8833	0.8918
**Ada Boost**	0.8152	0.8370	0.8811	0.8648	0.8727
**LGBM**	0.8140	0.8739	0.8603	0.8867	0.8719
**Logistic Regression**	0.8001	0.8801	0.8419	0.8750	0.8580
Model (Biochemical)	Accuracy	AUC	Recall	Precision	F1-Score
**LGBM**	0.8714	0.9397	0.9203	0.9029	0.9108
**Ada Boost**	0.8575	0.8950	0.8603	0.9386	0.8969
**Random Forest**	0.8297	0.9075	0.8799	0.8819	0.8803
**KNN**	0.8285	0.9449	0.8419	0.9265	0.8707
**Logistic Regression**	0.8001	0.9047	0.8419	0.8760	0.8563
Model (Molecular+Biochemical)	Accuracy	AUC	Recall	Precision	F1-Score
**KNN**	0.8714	0.9000	0.9600	0.8848	0.9159
**Random Forest**	0.8571	0.9100	0.9200	0.8981	0.9029
**Ada Boost**	0.8571	0.8900	0.9200	0.8933	0.9014
**LGBM**	0.8286	0.9100	0.8800	0.8933	0.8812
**Logistic Regression**	0.7857	0.8500	0.8400	0.8700	0.8455

Then, the selected classifiers were applied to the testing set for the T2DM drug response prediction and to evaluate their predictive performance on unseen data. Using this strategy, we ensured that only the most effective classifiers were applied for prediction for each feature group, thereby enhancing the reliability and robustness of our predictive models. **Table 8** summarizes the evaluation metric for the testing set. Notably, all classifiers in the three models achieved the same prediction power (0.8).

**Table 8:** The evaluation metric for the best classifiers on the testing set for each feature group.

Model (Molecular)	Accuracy	AUC	Recall	Precision	F1-Score
**KNN Classifier**	0.8	0.8942	0.8095	0.8947	0.85
Model (Biochemical)	Accuracy	AUC	Recall	Precision	F1-Score
**LGBM**	0.8	0.93	0.9048	0.8261	0.8633
Model (Molecular+Biochemical)	Accuracy	AUC	Recall	Precision	F1-Score
**KNN Classifier**	0.8	0.8968	0.8571	0.8571	0.8571

#### 4.5.3 Performance evaluation of machine learning models for drug response prediction

**Figure 6:**
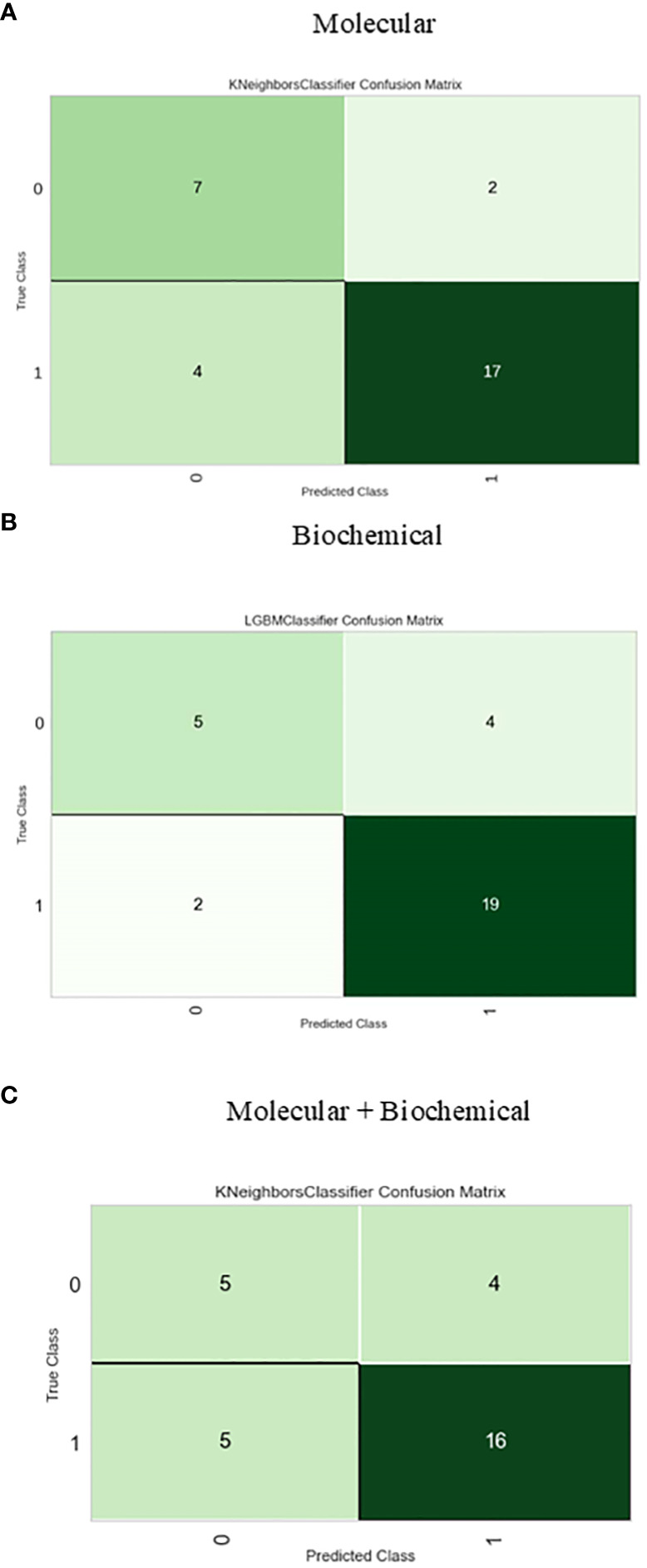
Confusion matrix for the best-performing classifiers for each feature group. **(A)** Molecular, **(B)** Biochemical, **(C)** Molecular+Biochemical.

**Figure 7:**
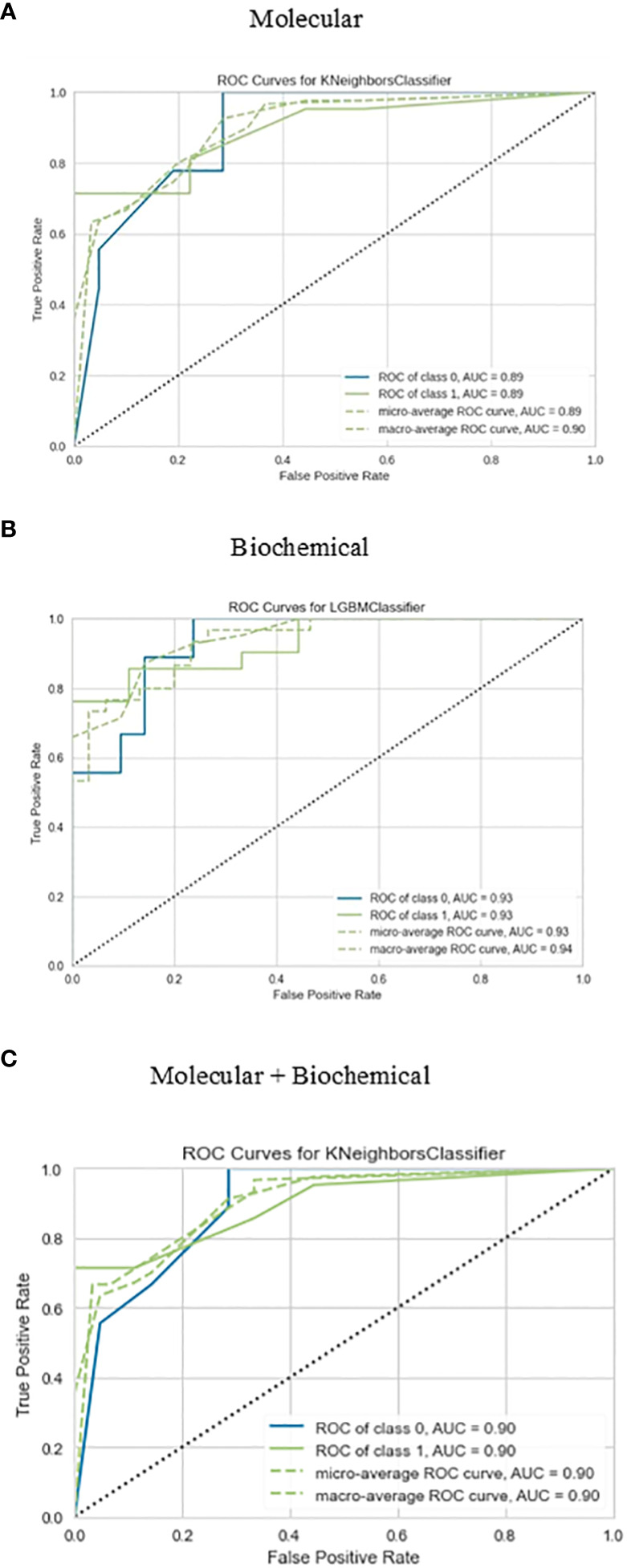
Roc curve for the best-performing classifiers for each feature group for the prediction of drug response. **(A)** Molecular, **(B)** Biochemical, **(C)** Molecular+Biochemical.

The confusion matrix presented in **Figure 6** displays the correctness of the prediction of whether the sample is improved (1), or not improved (0) on the test set, for the molecular, biochemical, and combined model. Roc curve displays how accurate the prediction models are (**Figure 7**).

#### 4.5.4 Visual analysis results for molecular and biochemical features

The implemented graphical approach displayed the differences in feature values by showing measures such as mean values and standard deviations between the different treated groups (drugs), against the normal and the T2DM groups (**Supplementary Figures S6**, **S7**).

## 5 Discussion

Diabetes mellitus is a metabolic disorder that impacts various organs and is primarily characterized by a deficiency in insulin production or response. This deficiency leads to impaired glucose tolerance and high blood glucose levels, accompanied by disruptions in the metabolism of fats, carbohydrates, and proteins (52).

Various factors, including modified dietary patterns, metabolic stress, and genetic predisposition, have the potential to activate the innate immune system. This activation leads to insulin resistance, T2DM, and related complications like dyslipidemia, diabetic nephropathy, retinopathy, and atherosclerosis. Among the drugs tested in our study, rosavin has garnered attention due to its antioxidant and anti-inflammatory properties, which help mitigate oxidative stress and chronic inflammation often observed in diabetes (53). Rosavin has been shown to lower total cholesterol, TGs, and LDL levels while increasing HDL, thereby exerting lipid-lowering effects. Insulin resistance precedes the abnormal elevation of blood glucose levels, which is the primary clinical indicator of T2DM. In the prediabetic stage, the body responds to insulin resistance by increasing insulin production to meet the normal insulin requirements. This leads to a state of chronic hyperinsulinemia and, as a result of sustained high blood sugar levels, the failure of pancreatic beta cells. Eventually, this progression leads to the development of T2DM. Prolonged and exaggerated metabolic stress can lead to detrimental inflammatory reactions, resulting in insulin resistance and inflammatory diseases. This chronic inflammatory state eventually gives rise to long-term complications associated with diabetes, including microvascular complications such as diabetic liver disease, diabetic nephropathy, neuropathy, and macrovascular complications like cardiovascular and cerebrovascular diseases. The cGAS-STING pathway plays a crucial role in diabetic complications and has been increasingly reported in diabetic nephropathy and diabetic angiopathy, which is linked to mitochondrial dysfunction caused by lipotoxicity. Evidence suggests that the cGAS-STING pathway is over-activated in diabetes and its complications. This heightened activation of cGAS-STING may serve as a protective mechanism, considering that diabetic patients are more susceptible to infections. Notably, knocking out STING has been shown to reduce insulin resistance induced by a high-fat diet in peripheral tissues and improve overall glucose intolerance. However, it is important to note that STING deficiency also impairs the ability of beta cells to secrete insulin in response to glucose stimulation. Autophagy is crucial in T2DM, serving to sustain cellular energy levels during fasting while eliminating damaged components like organelles, lipids, and misfolded proteins. Moreover, it contributes to pancreatic beta cell function and insulin resistance. Recent findings highlight the role of autophagy in T2DM pathophysiology, particularly in maintaining pancreatic beta cell function. Furthermore, increased autophagy serves as a protective mechanism against oxidative stress in insulin-targeted tissues like the liver, adipose tissue, and skeletal muscle. NOD1 and NOD2 are implicated in the development of diabetes, likely through their interaction with the gut microbiota. Antibiotic-induced changes in the gut microbiota are crucial for enhancing insulin sensitivity. Nucleotide-binding oligomerization domain (NOD)-like receptors, such as NOD1 and NOD2, both recruit receptor-interacting protein kinase 2 (RIPK2), but they exert opposite effects on blood glucose regulation. While NOD1 links signals from bacterial cell walls to metabolic inflammation and insulin resistance, NOD2 can foster immune tolerance, improve insulin sensitivity, and enhance blood glucose control in obesity. Similarly, NLR family pyrin domain-containing (NLRP) inflammasomes can elicit different metabolic outcomes. NLRP1 may protect against obesity and metabolic inflammation, possibly due to its preference for regulating IL-18, whereas NLRP3 tends to promote IL-1β-mediated metabolic inflammation and insulin resistance. Additionally, Rosavin may protect against diabetic complications such as neuropathy and nephropathy by reducing nerve damage, kidney injury, and hepatic damage as indicated by decreased levels of creatinine, BUN, ALT, and AST (30). Specifically, rosavin has demonstrated protective anti-inflammatory effects in various models, including bleomycin-induced pulmonary fibrosis. Rosavin exerts its effects by downregulating the expression of pro-inflammatory molecules such as NF-κB, p65, TGF-β1, and α-SMA while upregulating the expression of nuclear erythroid 2-related factor 2 (*Nrf2*), a transcription factor involved in antioxidant defense (54) Our findings supported the hypoglycemic (lower serum glucose, insulin, and HOMA-IR) and hypolipidemic (lower serum TG, TC, LDL, and higher HDL levels) effects of rosavin treatment (55). Mao. reported that Rhodiola rosea L. root extracts improved oral glucose tolerance, decreased serum TG, and LDL levels, and increased HDL levels in KKAy mice, a T2D model (56). Liu et al. proved that rosavin has been shown to attenuate LPS-induced activation of the TLR-4/NF-κB signaling pathway in RAW264.7 cells and inhibit the release of inflammatory factors in A549 cells. In a dose-dependent manner, rosavin ameliorated histopathological alterations, reduced the levels of inflammatory factors, and inhibited the TLR-4/NF-κB/MAPK signaling pathway and apoptosis activation. It also significantly reduced the number of inflammatory cells in bronchoalveolar lavage fluid and the expression of NF-κB p65 protein in the lung tissue of a mouse model. Moreover, it reduced the expression of hydroxyproline and malondialdehyde while enhancing the activities of superoxide dismutase and glutathione peroxidase in lung tissue (57).

Zbiotics, a newly engineered probiotic, has not been fully investigated for its impact on the pathogenesis of DM. Therefore, we conducted a study to assess its effectiveness on biochemical and molecular markers associated with DM. Both low and high doses of zbiotics demonstrated hypoglycemic and hypolipidemic effects, along with hepatoprotective and renoprotective effects evidenced by reductions in AST, ALT, creatinine, and BUN levels, particularly noticeable with the high dose. Histopathological analysis revealed mild hepatocellular microvesicular steatosis with minimal inflammatory cell infiltration. Our findings indicate that zbiotics with engineered acetaldehyde dehydrogenase can eloquently explain the results of the present study regarding how ZBiotics^®^ reversed the toll of diabetes on the studied histopathological, biochemical and molecular levels. The concept was based on two major mechanisms that could elicit inflammation that leads over time to diabetic settings and evolves diabetic complications; firstly, the oxidative-stress-induced inflammation, and secondly the disturbance in diabetic gut microbiota and barriers that could lead to the activation of subsequently inflammation.

Isorhamnetin has emerged as a promising therapeutic agent for T2DM by improving gut health and insulin resistance (29). Previous studies have demonstrated its efficacy in lowering fasting blood glucose levels, improving renal function, and ameliorating dyslipidemia in T2DM rats by upregulating autophagy in renal tissues (26). In animal models, Isorhamnetin supplementation has been found to reduce reactive oxygen species levels, inhibit atherosclerotic plaque formation, and mitigate myocardial hypertrophy and fibrosis ((58) (Mechanistically, Isorhamnetin acts through various pathways, including inhibition of the PI3K/AKT signaling pathway, activation of the AMPK/mTOR pathways, and modulation of insulin secretion via phosphorylation of insulin receptor substrate-2 (*IRS-2*), phosphatidylinositol 3-kinase (*PI3K*), Akt, and activated pancreatic and duodenal homeobox-1 (*PDX-1*) (59, 60). Moreover, Yang et al. proved that isorhamnetin exhibited hepatoprotective effects by reducing liver fibrosis through inhibition of HSC activation and ECM formation, and by downregulating the TGF-β1/Smad3 and TGF-β1/p38 MAPK pathways (61). In our previous study, we demonstrated that Isorhamnetin acted as an effective therapy for DM by modulating the insulin resistance signaling pathway and autophagy-related RNA network (28). Building upon our previous findings, our current study expands on the beneficial effects of isorhamnetin. We observed that isorhamnetin improved insulin resistance parameters, reduced elevated glucose levels, and alleviated inflammation infiltration in liver sections. Furthermore, isorhamnetin treatment led to the recovery of elevated levels of ALT and AST, indicating its hepatoprotective effects against chronic injury. isorhamnetin efficiently improves altered lipid metabolism by decreasing TGs, LDL, and TC, while increasing HDL. These lipid-lowering effects contribute to the hepatoprotective role of isorhamnetin, which can be attributed to its anti-inflammatory properties. Additionally, Lu et al. demonstrate that Isorhamnetin affects the P38/PPAR-α pathway, which in turn regulates the expression of apoptosis and autophagy-related proteins (62). This finding is consistent with our study, where Isorhamnetin modulated autophagy-related genes such as *STING1, IGF-1*, and *AKT2*.

Caffeic acid demonstrates anti-diabetic effects through multiple mechanisms, including the enhancement of antioxidant enzymes, inhibition of NF-κB signaling pathways, and activation of the NrF2 transcription factor (63). In a study by Xu et al., it was shown that caffeic acid administration at doses of 25 and 35 mg/kg significantly reduced plasma glucose, TG, TC, and LDL levels, while notably increasing HDL, insulin, and antioxidant levels in streptozotocin-induced diabetic Wistar rats after five weeks of treatment (64). The findings from Xu’s study align with our research, where we investigated the effects of caffeic acid treatment at doses of 10 and 50 mg/kg and we observed significant improvements in plasma glucose, lipid profiles, and insulin levels in diabetic rats treated with caffeic acid. Additionally, our results showed hepatoprotective and renoprotective effects of caffeic acid, along with a reduction in inflammatory cell infiltration in the liver and a decrease in epididymal adipocyte cell size, particularly with the higher dose. Furthermore, our previous research indicated that caffeic acid may activate the mitogen-activated protein kinases (MAPK) signaling pathway through the regulation of miR-636, leading to the induction of autophagy and attenuation of diabetic nephropathy (65). Consistent with this, our recent findings demonstrate that caffeic acid treatment significantly downregulates the expression of autophagy-related genes such as *IGF1R, NFKB1*, and *STING1* in adipose and liver tissues, further supporting its beneficial effects in diabetes management. Bhattacharya et al. investigated the effects of caffeic acid on glucose sensitivity, glucose-stimulated insulin secretion (GSIS), and gene expression in INS-1E cells under normoglycemic conditions (NC) and glucotoxic conditions (GC). They found that caffeic acid significantly increased the expression of Insulin-1 (*Ins-1*), *Ins-2*, pancreatic and duodenal homeobox 1 (*Pdx-1*), *Akt-1, Akt-2*, insulin receptor substrate-1 (*Irs1*), *Bcl2*, heat shock protein 90 and 70 (*Hsp90* and *Hsp70*) during NC. Additionally, caffeic acid downregulated acetyl coenzyme A carboxylase 1 (*ACC1*) without affecting Glucokinase (*Gck*) and Glucose transporter-2 (*Glut-2*) expressions in INS-1E cells. However, under GC conditions, caffeic acid did not change the expression of *GLUT-2, Gck, Ins2, Beta2, Pdx1, Akt2, Irs1, Bcl2, and Hsp90*. Instead, it upregulated *Ins1, Akt2*, and *Hsp70*, while downregulating *Beta2*, Caspase 3 (*Casp3*), and *Bax*. caffeic acid also significantly increased glucose sensitivity and GSIS in INS-1E cells and thereby caffeic acid may enhance the survival and function of β-cells during glucotoxic conditions by modulating the expression of these genes (66).

Many human and animal studies investigated prolonged drug exposure on both safety and efficacy outcomes. Lekomtseva et al. investigated the effects of Rhodiola rosea extract on prolonged or chronic fatigue symptoms, 100 subjects were administered 2 × 200 mg of the extract daily over 8 weeks of an open-label clinical trial. Results showed the greatest improvement after just 1 week of treatment, with continued reduction in fatigue symptoms throughout the study, reaching statistically significant improvement by week 8. Importantly, safety assessments indicated favorable outcomes, with most adverse events being mild and unrelated to the study drug (67). Ochoa-Morales et al. conducted a 12-week double-blind, randomized placebo-controlled trial that assessed the efficacy of propolis compared to placebo in controlling glycemic levels in 36 patients with T2DM. Administered twice daily before breakfast and dinner, propolis (300 mg) significantly reduced fasting plasma glucose (FPG) and 2-hour post-load glucose (2-h PG) levels compared to placebo (48). In a 6-month masked, randomized clinical trial conducted by El‐Sharkawy et al. individuals with chronic periodontitis (CP) and T2DM undergoing scaling and root planning (SRP) were given either a placebo or a daily regimen of 400 mg oral propolis. Results showed that the propolis group exhibited significant reductions in hemoglobin A1c (HbA1c) levels by 0.82% and 0.96% units at 3 and 6 months, respectively, along with decreases in fasting plasma glucose (FPG) and serum N€-(carboxymethyl) lysine (CML) levels. Additionally, both groups showed improvements in periodontal parameters after therapy, but the propolis group demonstrated significantly greater reductions in probing depth and gains in clinical attachment level compared to the control group (68). In a study investigating the effects of caffeic acid on diabetic cardiomyopathy, it was found that caffeic acid, along with ellagic acid, demonstrated protective effects in diabetic mice. Various parameters, including lipid profile, coagulability, oxidative stress, and inflammation, were assessed. After 12 weeks, the treated animals showed beneficial effects, including decreased triglyceride levels, increased plasma insulin levels, decreased plasma glucose levels, anti-coagulatory effects, antioxidative effects, and anti-inflammatory properties in the cardiac tissue (69). Moreover, Rodríguez-Rodríguez et al. investigated the metabolic effects of an extract from Opuntia ficus-indica (OFI) for 12 weeks, known for its high isorhamnetin glycoside content, in a mouse model of diet-induced obesity and isolated pancreatic islets. Mice fed a high-fat (HF) diet supplemented with OFI extract exhibited reduced body weight gain and lower levels of circulating total cholesterol, LDL cholesterol, and HDL cholesterol compared to those on the HF diet alone. Furthermore, HF-OFI diet-fed mice showed lower glucose and insulin concentrations but slightly higher insulin levels compared to control mice. These metabolic enhancements were associated with decreased adipocyte size, enhanced hepatic phosphorylation of IRS1 tyr-608 and S6 K thr-389, and reduced hepatic lipid content (70). Jamali-Raeufy et al. studied the effect of Isorhamnetin on diabetic male rats for 12 weeks. Isorhamnetin, administered intraperitoneally at a dose of 10 mg/kg body weight once daily, elicited significant effects on various parameters. Notably, Isorhamnetin treatment led to a marked reduction in pain severity and blood glucose levels, while also promoting a significant increase in body weight compared to the control group. Moreover, Isorhamnetin demonstrated inhibitory effects on astroglial activation, acetyl-cholinesterase activity, oxidative stress markers, apoptosis, and inflammatory markers within diabetic rats (58).

In this study, we incorporated molecular biomarkers (mRNAs-miRNAs-LncRNAs) expressed in livers and adipose tissues of animal models representing normal, T2DM, and treated groups alongside conventional biochemical parameters. Our objective was to utilize these predictive targets to select the most potent candidates for treating T2DM using medicinal plant-based drugs such as Rosavin, isorhamnetin, and Caffeic acid, as well as probiotics like Z-biotic. To achieve this goal, we developed a machine-learning model using 5 different algorithms (KNN, RF, LR, LGBM, and Ada Boost) to recognize the significant features associated with T2DM to achieve a reliable and effective improvement prediction.

As expected, the diabetic rat models exhibited the highest levels of serum insulin resistance index (HOMA-IR), insulin, fasting blood glucose, as well as biomarkers indicating renal function impairment, liver damage, and lipid profiles. Conversely, the normal group, as well as the groups treated with medicinal plants and probiotics, displayed the lowest levels of these markers, more obvious in the highest drug doses, with an opposite relationship observed for HDL (**Table 4**). As a heterogeneous condition, T2DM is typified by impaired insulin secretion (known as the beta cell secretory defect) and insulin resistance, leading to elevated blood glucose levels. Various disturbances in biochemical parameters are associated with T2DM, including dyslipidemia, which involves elevated fasting and postprandial TG, decreased levels of HDL-C, increased levels of LDL-C, and a prevalence of small, dense LDL-C particles (71). Liver enzymes play a crucial role in regulating metabolism, particularly in maintaining normal blood glucose levels during fasting and after meals. Insulin resistance in the liver leads to increased glycogenolysis and lipolysis, resulting in elevated hepatic glucose production and abnormal triglyceride and fatty acid synthesis. These abnormalities, including elevated levels of AST, ALT, and ALP, are indicative of liver dysfunction and can precede the onset of fasting hyperglycemia. The elevation of these enzymes may be attributed to the direct hepatotoxic effects of excess fatty acids, which can disrupt cell membranes and impair mitochondrial function (72). Additionally, oxidative stress, peroxisomal beta-oxidation, and inflammation mediated by pro-inflammatory cytokines like TNF-α contribute to hepatic injury. Furthermore, the increased activity of ALT, a gluconeogenic enzyme suppressed by insulin, suggests a disruption in insulin signaling rather than solely hepatocyte injury (73, 74). Prolonged high blood glucose levels lead to increased oxidative stress, inflammation, and dysfunction of microvascular endothelial cells, which are common underlying mechanisms in both diabetic nephropathy (DN) and diabetic retinopathy (DR). These conditions often coexist, with elevated blood urea nitrogen (BUN) levels indicating their presence. BUN levels are also linked to the body’s catabolic activity and may reflect reduced blood flow and increased oxidative stress. Additionally, microvascular hypoperfusion and oxidative stress contribute to the development of DR, potentially explaining the association between elevated BUN levels and DR (75). Serum creatinine, primarily metabolized by skeletal muscle, correlates with total skeletal muscle mass. Low serum creatinine levels are considered a risk factor for T2DM and dysglycemia, as they reflect reduced skeletal muscle mass. Skeletal muscle plays a crucial role in glucose uptake, with reduced uptake contributing to insulin resistance and the development of T2DM long before hyperglycemia becomes apparent. The decrease in skeletal muscle mass is thus associated with increased insulin resistance and the risk of developing T2DM (76, 77).

We next determined the histopathological alterations in the studied groups, the livers of the T2DM-induced rats showed significant changes, including fat accumulation, cell death, inflammation, and abnormal cell morphology, in addition to an increase in the adipocyte cell size. Treatment with Rosavin at a higher dose restored the liver structure to normal, while Z-Biotic and isorhamnetin showed moderate improvements. caffeic acid had some positive effects but to a lesser extent. The administration of rosavin, particularly at a high dosage, significantly decreased the severity of liver inflammation grades. This outcome aligns with our previous findings in a rat model of non-alcoholic steatohepatitis (NASH), where rosavin treatment improved liver functions, and lipid profile, and mitigated hepatic inflammation, fibrosis, and cell death (78). Overall, the drugs Rosavin, Z-Biotic, isorhamnetin, and Caffeic acid exhibited varying effects on the destructive liver architecture induced by diabetes, with higher doses generally demonstrating a more restorative effect and this was also obvious in decreasing the adipocyte cell diameter and liver inflammation grades.

Inflammation and autoimmunity play significant roles in the development of diabetes, and hence, targeting the inflammatory response has shown therapeutic benefits (79, 80). The NOD signaling pathway is involved in the inflammation triggered by the cGAS-STING pathway (81, 82). Perturbations in the gut microbiota in diabetic patients can, in turn, activate the cGAS-STING-NOD pathway, leading to inflammation (83). Paradoxically, we found that the probiotics Z-Biotic 1mg significantly modulated most of the inflammatory-enriched mRNAs (*DDX58*, *NFKB1*, *CHUK*, *RET*) that were implicated in the STING/NOD/IR pathways and made them decrease significantly to be close to the normal group ratios, interestingly, the high dose of caffeic acid behaved similarly.

The cytoplasmic DNA sensor known as cyclic GMP-AMP synthase (cGAS) directly binds to DNA, including mitochondrial DNA (mtDNA) and abnormal bacterial or viral DNA, leading to its activation. Upon activation, cGAS triggers the synthesis of a secondary messenger called 2′,3′-cGAMP, which then binds to and activates the stimulator of the interferon gene (STING). STING, a transmembrane protein primarily found in the endoplasmic reticulum (ER), undergoes translocation to the Golgi and recruits the downstream TANK-binding kinase 1 (TBK1) to form a complex. This complex, in turn, phosphorylates and activates interferon regulatory factor 3 (IRF3) and nuclear factor kappa B (NF-κB), initiating a cascade of signals that activate various innate immune-related genes, including type I interferon (IFN) (84). Recent studies have implicated the cGAS-STING pathway in the development of diabetic cardiomyopathy, a condition characterized by aseptic inflammatory activation. Notably, increased mtDNA in the cytosol and the activation of the cGAS-STING signaling pathway, along with its downstream targets such as IRF3, NF-κB, IL-18, and IL-1β, have been observed in the context of diabetic cardiomyopathy (85). A previous study discovered that activating *STING* resulted in an induction of the *NLRP3* inflammasome and pyroptosis, which culminated in an inflammatory response observed in diabetic mice (86). Qiao et al. found that *STING* plays a unique role in regulating insulin action in peripheral metabolic tissues and insulin secretion from β-cells (87).DDX58 participates in innate immune responses through the NOD-like receptor signaling pathway, detecting cytoplasmic DNA and triggering the production of interferon 1 and inflammatory cytokines (88, 89). Moreover, the cGAS-STING1 and DDX58-MAVS pathways are connected to the innate immune response (90).In a study conducted by Frietze et al. using a NASH model, it was shown that activation of DDX58 leads to the cleavage of LC3, a marker linked to autophagosome formation, DDX58 interacts with the autophagy receptor protein SQSTM1 to degrade itself selectively after viral stimulation. Activation of DDX58 enhances autophagic responses, aiding in the removal of harmful lipid inclusion bodies associated with inflammation and cell death. Excessive fatty acids hinder DDX58 activity, reducing crucial autophagic responses and worsening lipotoxicity. The study revealed that DDX58 directly influences SQSTM1 gene expression, protein accumulation, and targeted autophagic degradation. Furthermore, sustained overexpression of DDX58 markedly reduces inflammation mediated by JAK-STAT signaling (91).Studies have demonstrated that ZBP1, a cytosolic nucleic acid sensor, plays a pivotal role in coordinating innate immune responses by triggering both NF-κB and interferon regulatory factors (IRFs) signaling pathways (92). Additionally, ZBP1 activation leads to the expression of inflammatory cytokines and induces various forms of inflammatory cell death, including necroptosis, pyroptosis, apoptosis, and PANoptosis, in response to different host-derived nucleic acids (93, 94). Moreover, *ZBP1* acts as a regulator of IFN-1-mediated disease progression, sensing mitochondrial DNA instability through the cGAS-STING pathway and sustaining IFN-1 signaling, which is involved in heart failure and cardiac cell remodeling (95, 96).

Stimulation of NOD and TNF receptors in intestinal epithelial cells activates *CHUK*, which stabilizes *ATG16L1*, preventing endoplasmic reticulum stress during inflammation (97). It also inhibits Kappa Beta kinases that suppress *NFKB1* (98). High glucose promotes proliferation and invasiveness in pancreatic cancer cells by upregulating *RET*, a proto-oncogene encoding a receptor tyrosine kinase (99). *ZBP1* acts as a regulator of IFN-1-mediated disease progression, sensing mitochondrial DNA instability through the cGAS-STING pathway and sustaining IFN-1 signaling, which is involved in heart failure and cardiac cell remodeling (95, 96) A previous study discovered that activating *STING* resulted in an induction of the *NLRP3* inflammasome and pyroptosis, which culminated in an inflammatory response observed in diabetic mice (86). Qiao et al. found that *STING* plays a unique role in regulating insulin action in peripheral metabolic tissues and insulin secretion from β-cells (87).

Intense evidence supports the involvement of miRNAs in the intricate regulation of glucose homeostasis, making them potential contributors to the development of diabetes (100). These small molecules exert their influence by impacting various aspects of insulin levels, including insulin production, and exocytosis (101) Additionally, lncRNAs can act as miRNA sponges by binding to miRNAs, thus preventing them from interacting with their mRNA targets (102). This regulatory mechanism plays a crucial role in diabetes pathogenesis, influencing beta-cell function, apoptosis, insulin secretion, glucose metabolism, and insulin resistance (103, 104).

We previously reported that miRNAs (has-miR-1976 and has-miR-611) acted as sponges on lncRNAs (AC074117.2 and RP4–605O3.4), decreasing the expression of mRNA (*CHUK* mRNA). Notably, these findings demonstrated the potential of has-miR(-1976 and 611) miRNAs as distinguishing factors between insulin-resistant and insulin-sensitive patients, and their involvement in modulating the STING/NOD/IR pathways (105). These results were consistent with our observations in animal models. Previous studies have highlighted the role of miR-1976 as a tumor suppressor in non-small cell lung cancer (106). *In vitro* experiments demonstrated that the suppression of miR-1976 resulted in a significant acceleration of wound healing and cell migration. Furthermore, it stimulated cell proliferation, decreased cell apoptosis, and increased the populations of CD44+/CD24− cells (107).

Our results suggested that RP11–773H22.4 could bind to miR-1, and miR-3163 and serve as a competing endogenous RNA (ceRNA) to upregulate *m-TOR*, *IGF1-R*, and downregulate *AKT2* expression, thus contributing to increased autophagy and the progression of T2DM, consistent with our previous results (28). Previous studies have indicated that insulin-like growth factor-1 (*IGF-1*) hinders the process of autophagy by inhibiting *AKT*. This inhibition is believed to be mediated by *AKT*’s activation of rapamycin complex 1 (*mTORC1*), a known inhibitor of autophagy, there for Inhibition of *IGF-1R* signaling cascade reduces autophagy, impacting autophagosome precursor formation and suggests that targeting the IGF-1R receptor or its downstream pathway may have implications for therapeutic purposes (108). Moreover, *Akt2* can inhibit the expression of the cGAS-STING pathway and suppress the inflammatory response in diabetes (109). Autophagy is crucial for maintaining cellular balance during stressful conditions, excessive or uncontrolled autophagy can trigger autophagy-dependent apoptosis, cardiac injury, and impaired function, primarily through autophagic cell death (110–112). MiR-1, which has been studied extensively, is often suppressed in various biological samples from patients with T2DM (113). Overexpression of miR-1 has been shown to inhibit cardiac fibrosis and apoptosis by altering the expression levels of *Bcl-2*, *TGFb1*, and *Bax* (114). Additionally, *mTOR* plays a significant role in cardioprotection, diabetes, cellular metabolism, apoptosis, autophagy, and mitochondrial biogenesis (115–117) Studies have demonstrated that inhibiting *mTOR* with rapamycin (*RAPA*) can reduce cardiomyocyte apoptosis following myocardial infarction (MI) stress (118). Moreover, in retinoblastoma cancer stem cells, miR-3163 influences cell proliferation, apoptosis, and drug resistance (119). Additionally, miR-3163 has been shown to enhance the sensitivity of hepatocellular carcinoma (HCC) cells to sorafenib by suppressing the cleavage of Notch protein (120).

The present study involved; i, Data preprocessing and feature selection: The dataset was carefully preprocessed to ensure data integrity and consistency with the study objectives. Null values ​​and duplicate entries were carefully handled to maintain the quality of the dataset, which was essential for subsequent analysis. Targeted variables created following data correction methods ‘standardization’ are needed to predict response to type 2 diabetes mellitus (T2DM) treatment Based on ‘fat cell diameter’ and ‘. Inflammation (liver)’ features used to divide the sample into “unimproved” and “improved” categories. Categorical features of the dataset were adjusted to facilitate their incorporation into the modeling pipeline. Robust coding techniques such as one-hot coding were used to ensure compatibility with machine learning algorithms. Feature selection was performed using cross-validation (RFECV) method and recursive feature elimination, which is the cornerstone of predictive modeling. This approach systematically identified factors that contribute to understanding T2DM drug response patterns. Notably, the selected molecules include ZBP1 (A), STING1(L), DDX58 (L), mTOR (L), NFKB1 (A), CHUK (A), RET (L), RET (A), miR-1. (A), miR-611 (L), miR-611 (A), LncRNA-RP11–773H22.4 (L), and LncRNA-RP11–773H22.4 (A). Furthermore, biological parameters such as glucose, insulin, HOMA-IR, total cholesterol, triglycerides, AST, ALT, creatinine, BUN, and ACR were identified as important contributors to the prediction of T2DM treatment response. ii, Training and evaluation model: The trained machine learning models, including K-Nearest Neighbors (KNN) and Light Gradient Boosting Machine (LGBM), were subjected to rigorous evaluation to assess their predictive prowess. Leveraging cross-validation techniques, the models demonstrated commendable performance, achieving an impressive accuracy of 80%. These findings underscore the robustness and efficacy of the selected features in discerning intricate patterns inherent in T2DM treatment response. Moreover, the systematic approach adopted in this study holds promise for enhancing therapeutic strategies and optimizing patient outcomes in clinical settings.

In this study, a feature selection approach based on the RFECV technique was adopted to select the top molecular and biochemical features that had the highest prediction accuracy for T2DM drug response in the dataset. The selected molecular features were *ZBP1* (A), *STING1* (L), *DDX58* (L), *mTOR* (L), *NFKB1* (A), *CHUK* (A), *RET* (L), *RET* (A), miR-1976 (A), miR-611 (L), miR-611 (A), LncRNA-RP11–773H22.4 (L), and LncRNA-RP11–773H22.4 (A). While the biochemical features were Glucose, Insulin, HOMA-IR, Total cholesterol, Triglycerides, AST, ALT, Creatinine, BUN, and ACR. The utilized molecular and biochemical models using these features combined or separately maintained decent prediction performance with top classifiers KNN and LGBM at an accuracy of 80%.

While our study provides valuable insights into the potential therapeutic efficacy of medicinal plant-based drugs and probiotics in treating T2DM, there are several limitations that should be acknowledged. Our study focused on a limited number of medicinal plant-based drugs and a single probiotic, which may not fully represent the spectrum of potential therapeutic interventions for T2DM. Future research incorporating a broader range of pharmacological agents and therapeutic modalities could provide a more comprehensive understanding of effective treatment strategies for this complex condition. Further larger studies addressing the possibility of multiple dose and long-term treatment regimens and their impact on the evaluation of drug safety and efficacy should be explored. Also, Further future validation through independent datasets is strongly needed to ensure the findings’ applicability in human T2DM treatment. Moreover, while our study assessed various molecular and biochemical features associated with T2DM pathogenesis, it is important to recognize that these chosen parameters may not capture the full complexity of the disease process. Additional factors such as genetic predisposition, environmental influences, and lifestyle factors may also contribute to individual variability in drug response and treatment outcomes. Further research is needed to address the limitations and translate these findings into clinically meaningful interventions for patients with T2DM. Researchers stressed the pivotal role of all members of brain, kidneys, pancreatic cells, alpha cells, and the gastrointestinal tract in the development of glucose intolerance besides the four cell types(liver, muscle and adipose tissues) (121). Thus further larger validation in other tissues is strongly recommended to ensure the generalization of the results.

## 6 Conclusion

We developed a prediction system for identification of potential therapeutic targets using machine learning algorithms with feature selection using mRNAs-miRNAs-LncRNAs implicated in autophagy and STING/NOD/IR pathways that directly correlated with T2DM pathogenesis in addition to biochemical features. Our results demonstrated that the KNN algorithm outperformed other classifiers in both the molecular and combined models, for the biochemical model, the LGBM Classifier exhibited the highest performance with AUC values of 0.9177, 0.9, and 0.9397, respectively. Notably, our machine learning approach successfully identified 20 significant features out of the total 44 features in the combined model with an accuracy of 80%.

## Data availability statement

The original contributions presented in the study are included in the article/**Supplementary Material**. Further inquiries can be directed to the corresponding author.

## Ethics statement

The animal study was approved by Research Ethics Committee of the Faculty of Medicine at Ain Shams University under federal-wide assurance NO. FWA000017585. The study was conducted in accordance with the local legislation and institutional requirements.

## Author contributions

MS: Writing – review & editing, Validation, Methodology, Investigation, Funding acquisition, Conceptualization. HA-A: Writing – review & editing, Investigation. AK: Writing – original draft, Visualization, Data curation. RK: Writing – original draft, Investigation, Formal analysis. MR: Writing – review & editing, Software. MA: Writing – review & editing, Investigation. GD: Writing – original draft, Methodology. ME: Writing – original draft, Visualization, Methodology. RE: Writing – review & editing, Supervision. HA: Writing – review & editing, Investigation. EA: Writing – original draft, Data curation. DE: Writing – review & editing, Investigation. HK: Writing – review & editing, Investigation. MF: Writing – original draft, Writing – review & editing, Methodology. HE: Writing – review & editing, Investigation. LF: Writing – review & editing, Validation. MBA: Writing – review & editing, Validation. EH: Writing – review & editing, Visualization, Resources. HF: Writing – review & editing, Validation. LS: Writing – review & editing, Validation. IA: Writing – review & editing, Writing – original draft, Methodology, Data curation.
