# Supplementary material for: Comprehensive machine learning models for predicting therapeutic targets in type 2 diabetes utilizing molecular and biochemical features in rats
Source: Front Endocrinol (Lausanne). 2024 May 24;15:1384984. doi: 10.3389/fendo.2024.1384984 (PMC11157016; doi:10.3389/fendo.2024.1384984)

Table S1; List of clinical trials conducted on propolis , Rhodiola Rosea and Artemisia dracunculus in diabetes mellitus and related diseases

| Drugs | ClinicalTrials.gov |
| --- | --- |
| Caffeic acid  (propolis as plant source) | ClinicalTrials.gov Identifier: NCT03416127  Phase: 2   - Propolis administration modified the glycemic control in patients with type 2 DM. - Propolis 300 mg twice a day for 12 wk |
|  | ClinicalTrials.gov Identifier: NCT02794506  Phase: 4  Status: Completed   - Improvement in HbA1c, FPG, serum CML, and changes in periodontal parameters - Propolis 400 mg for 6 mo, after performing scaling and root planning |
|  | ClinicalTrials.govIdentifier: NCT03649243  Phase: Not applicable   - Propolis possesses anti-inflammatory and antioxidant effects and its topical application is well tolerated, improving the healing of human diabetic foot ulcer - Propolis spray at the site of injury |
| Isorhamantin  (Artemisia dracunculus as plant source) | - ClinicalTrials.gov Identifier: NCT00961909, Phase 1 - A Study of RO5095932 in Patients With Type 2 Diabetes Mellitus - This randomized, double-blind, placebo-controlled study will assess the efficacy, safety, tolerability, pharmacokinetics and pharmacodynamics of RO5095932 in patients with type 2 diabetes mellitus. Patients will be randomized to receive either RO5095932 subcutaneously once weekly or placebo for 4 weeks (part 1) or 6 weeks (part 2), in addition to their current stable doses of metformin. |
| Rosavin  (Rhodiola Rosea as plant source) | ClinicalTrials.gov Identifier ;NCT06176599  Clinical Study on the Treatment of Diabetic Kidney Disease With Shenxiao Yuning Decoction With Syndrome of Qi and Yin Deficiency and Blood Stasis |
|  | ClinicalTrials.gov Identifier ;NCT01278992, **phase 2**  Rhodiola Rosea for Mental and Physical Fatigue |
|  | ClinicalTrials.gov Identifier ; NCT03633890, Phase 4  DaZhu Rhodiola Rosea Capsule for Coronary Artery Disease With Angina Pectoris |
|  | ClinicalTrials.gov Identifier ;NCT04218916, phase 3  Rhodiola Rosea for Coronary Microvascular Disease |
|  | ClinicalTrials.gov Identifier ; NCT04623164, phase 4  Complex Phytoadaptogenes in Chronic Periodontitis Treatment |
|  | ClinicalTrials.gov Identifier ; NCT02737033, phase 4 Effects of 800mg of Rhodiola Rosea in Attention in Adults With Attention-Deficit/Hyperactivity Disorder |
|  | ClinicalTrials.gov Identifier ; NCT03461380, phase 2Effect of Menopause Relief EP-40 in Women With Menopausal Symptoms  - This study compares the efficacy of a fixed combination of black cohosh (EP-40) and Rhodiola rosea (EPR-7) with low (6.5 mg) and high doses (500 mg) of a standardized black cohosh extract only in adult woman with menopausal complaints. |

**Table S3. Details of the GSE142025 and GSE20966 datasets that were retrieved from the GEO database.**

| Accession number | Platform | Organism | Experimental design |
| --- | --- | --- | --- |
| GSE20966 | GPL1352 [U133_X3P] Affymetrix Human X3P Array | Homo sapiens | The gene expression profile of beta-cells obtained from cadaver pancreases of 10 control and 10 type 2 diabetic subjects was evaluated. Beta-cells were acquired from pancreatic tissue sections using the laser capture microdissection technique. RNA was extracted, amplified, biotinylated, and hybridized to GeneChip Human X3P Array (Affymetrix). Array data were normalized, and analysis was performed using the DNA-Chip Analyzer software. |
| GSE142025 | GPL20301 Illumina HiSeq 4000 (Homo sapiens) | Homo sapiens | A total of 28 patients with biopsy-proven DN hospitalized from January 2015 to December 2016 in Shanghai Jiao Tong University Affiliated Sixth People’s Hospital were enrolled in the study. Nine control human kidney samples were obtained from the unaffected portion of tumor nephrectomies. RNA-seq was performed on 28 DN and 9 control samples. |

| **Figure S1. The volcano plot shows the highly significant DEGs of the GSE20966 and GSE142025 datasets between the normal control cells and the DM patients using the GEO2R/Limma analysis, the x-axis represents the log2Fc, and the y-axis represents the -log10 of an adjusted p-value. The up-regulated genes in the dataset are represented in red color and the down-regulated genes are represented in blue color.**   \| 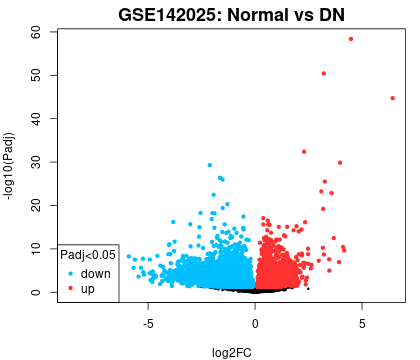 \| 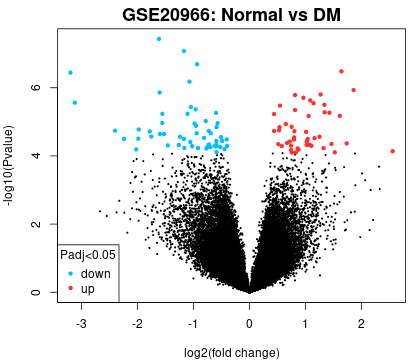 \| \| --- \| --- \|   *Abbreviations*: DN; Diabetic Nephropathy, DM; Diabetes Mellitus. |  |
| --- | --- | --- | --- |

**Figure S2 Pathway enrichment analysis of DEmRNA using using Enrichr (http://amp.pharm.mssm.edu/Enrichr, Jan 2022)**

**
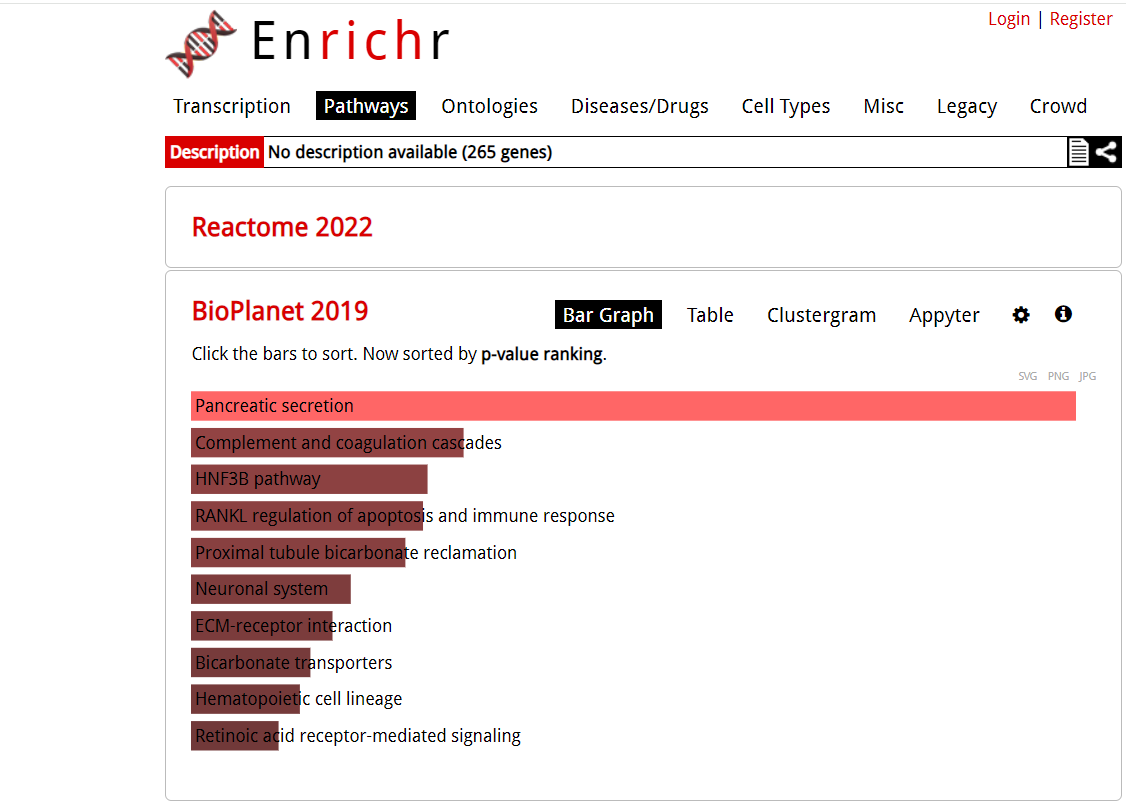
**

**Figure S3. Retrieval of candidate mRNAs related to diabetes pathogenesis pathways using Genecards database (**[**https://www.genecards.org/**](https://www.genecards.org/)**, assessed on Jan 2024)**


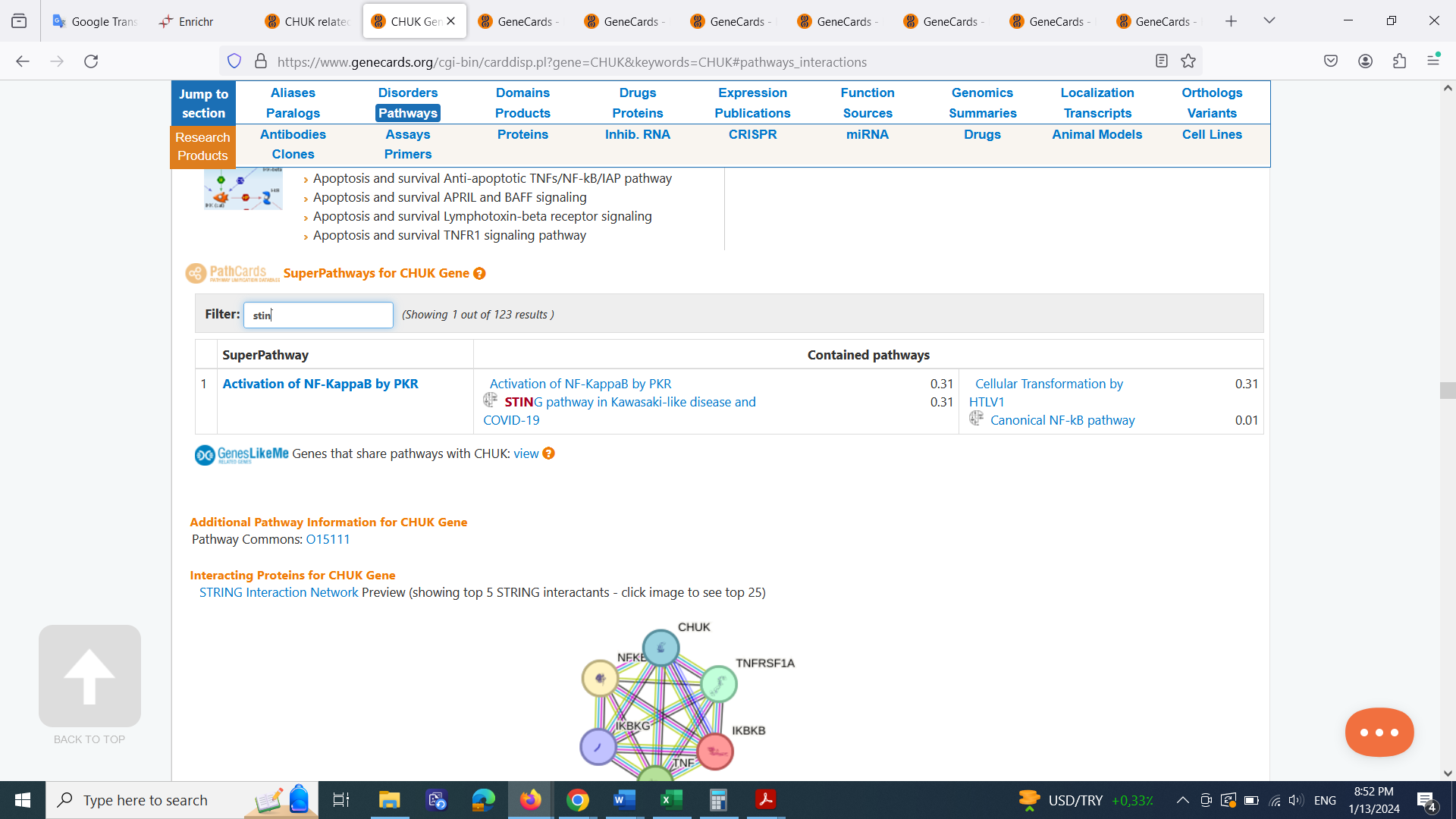


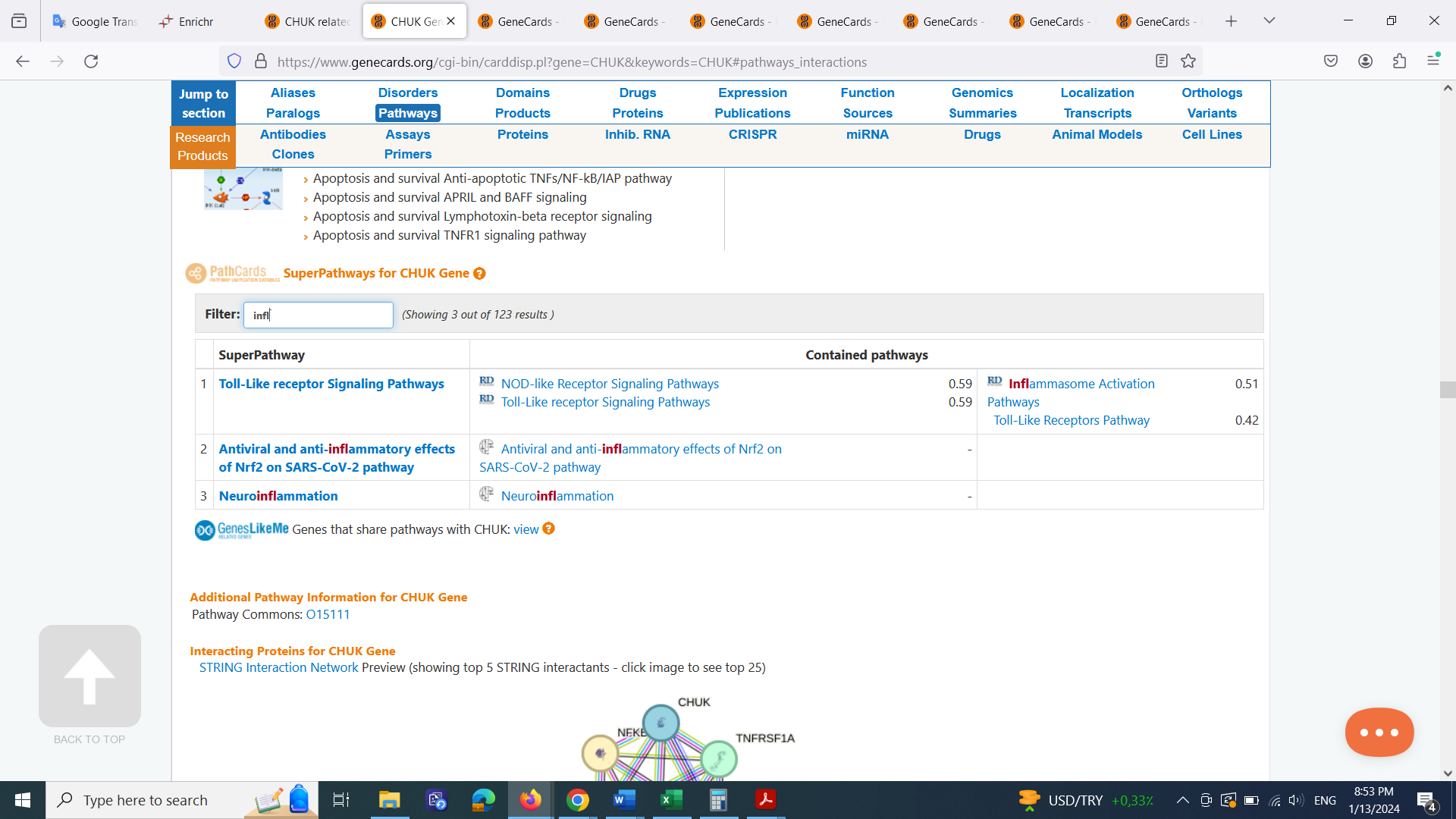


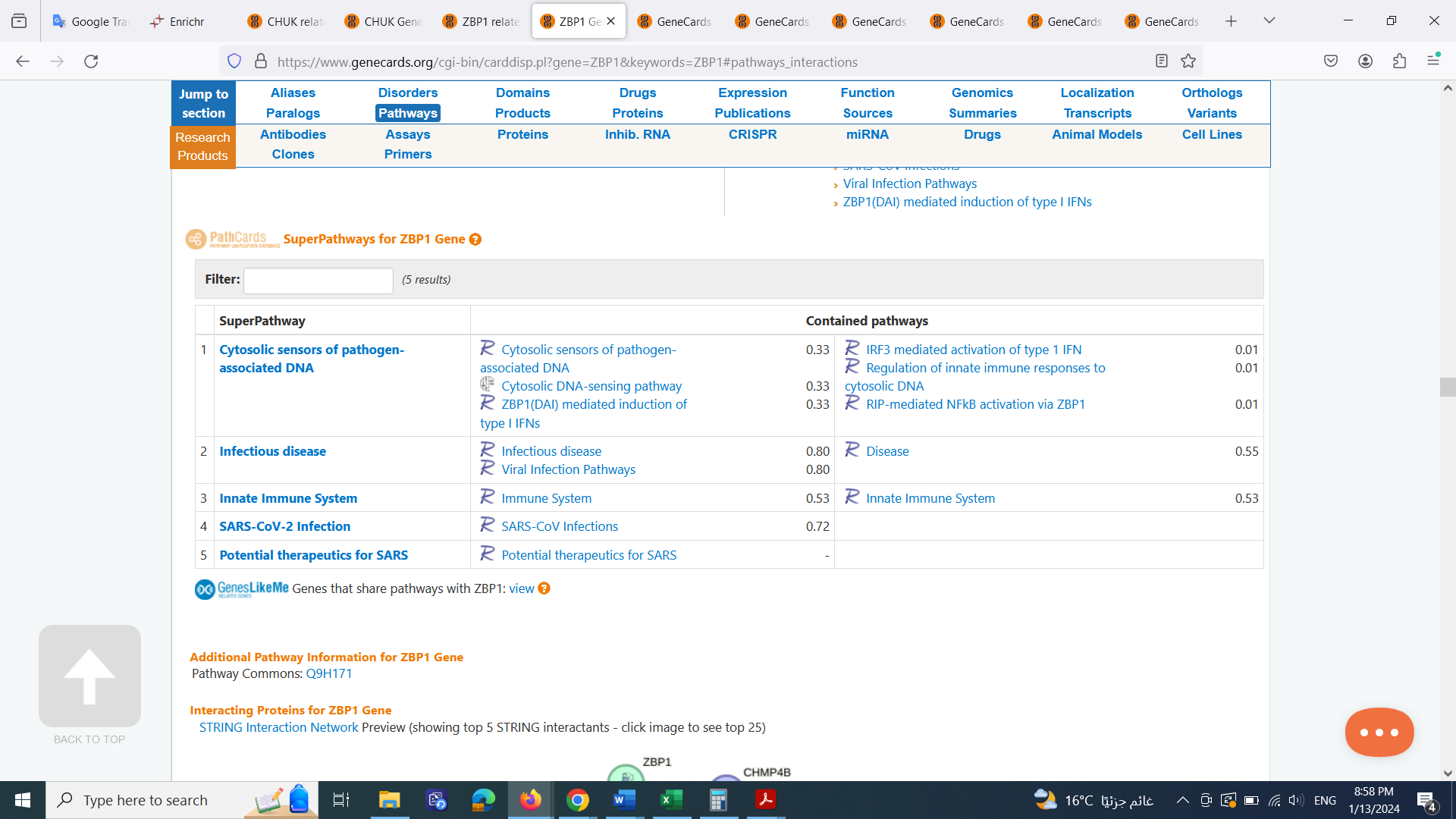


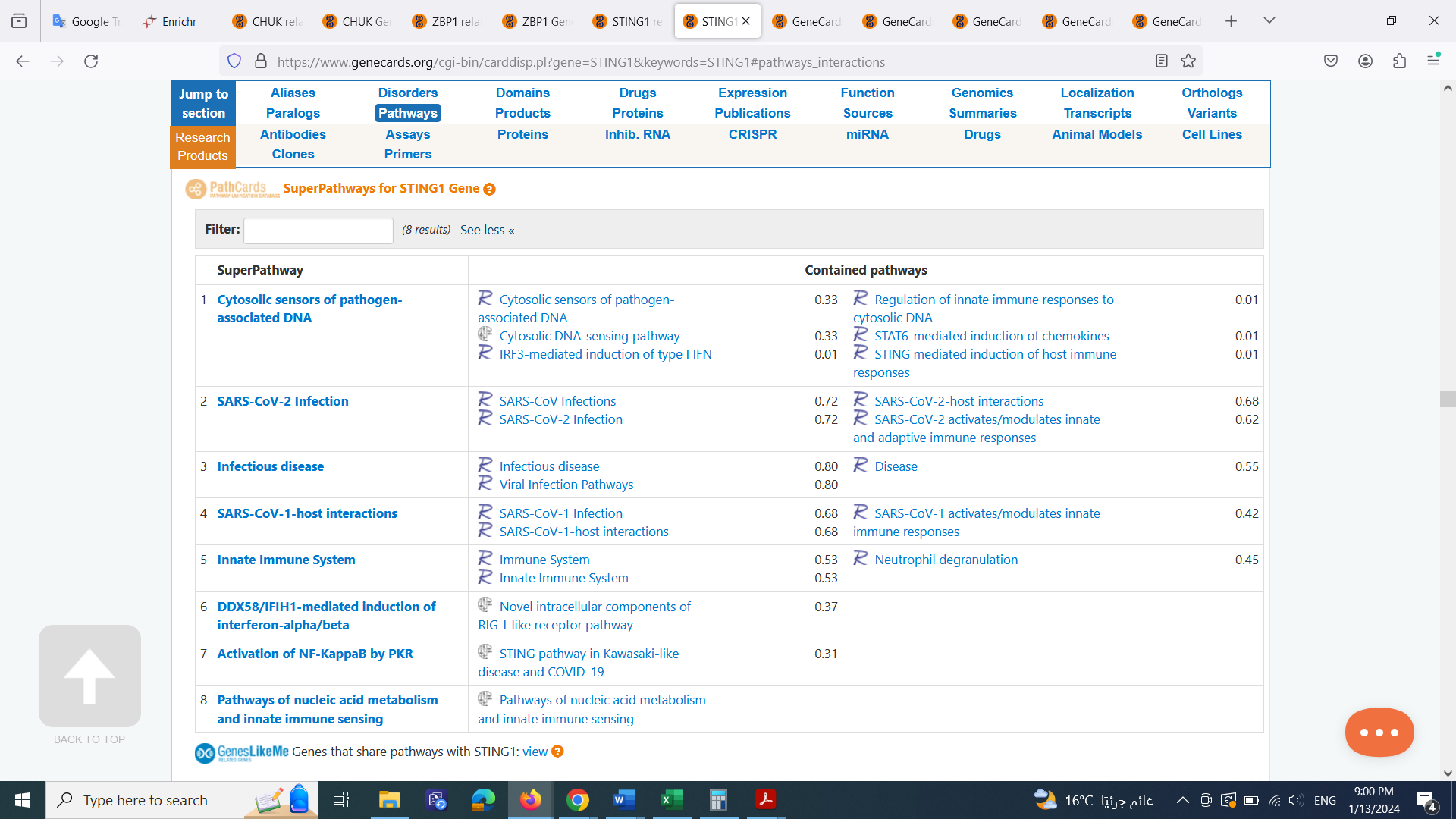


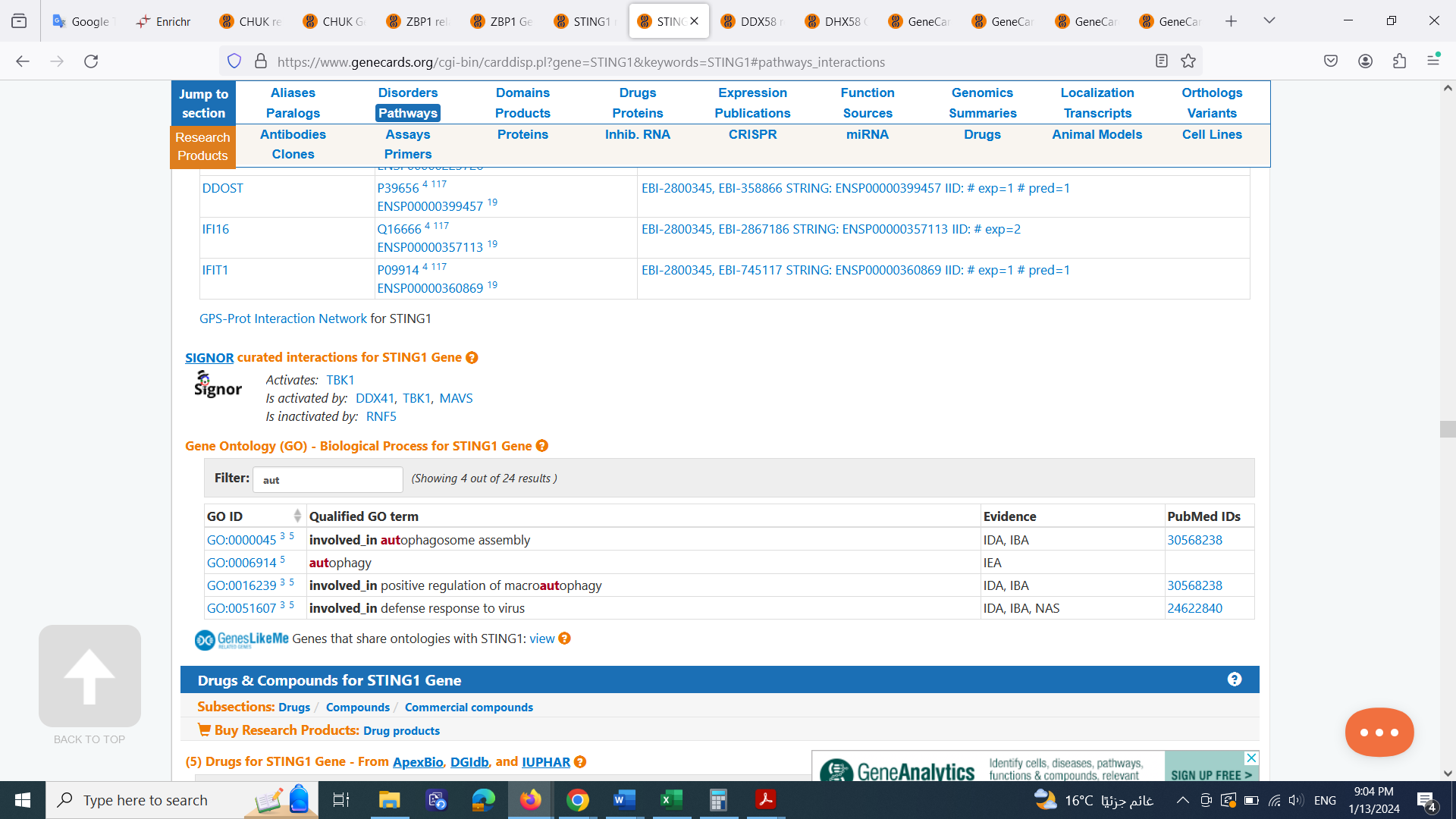


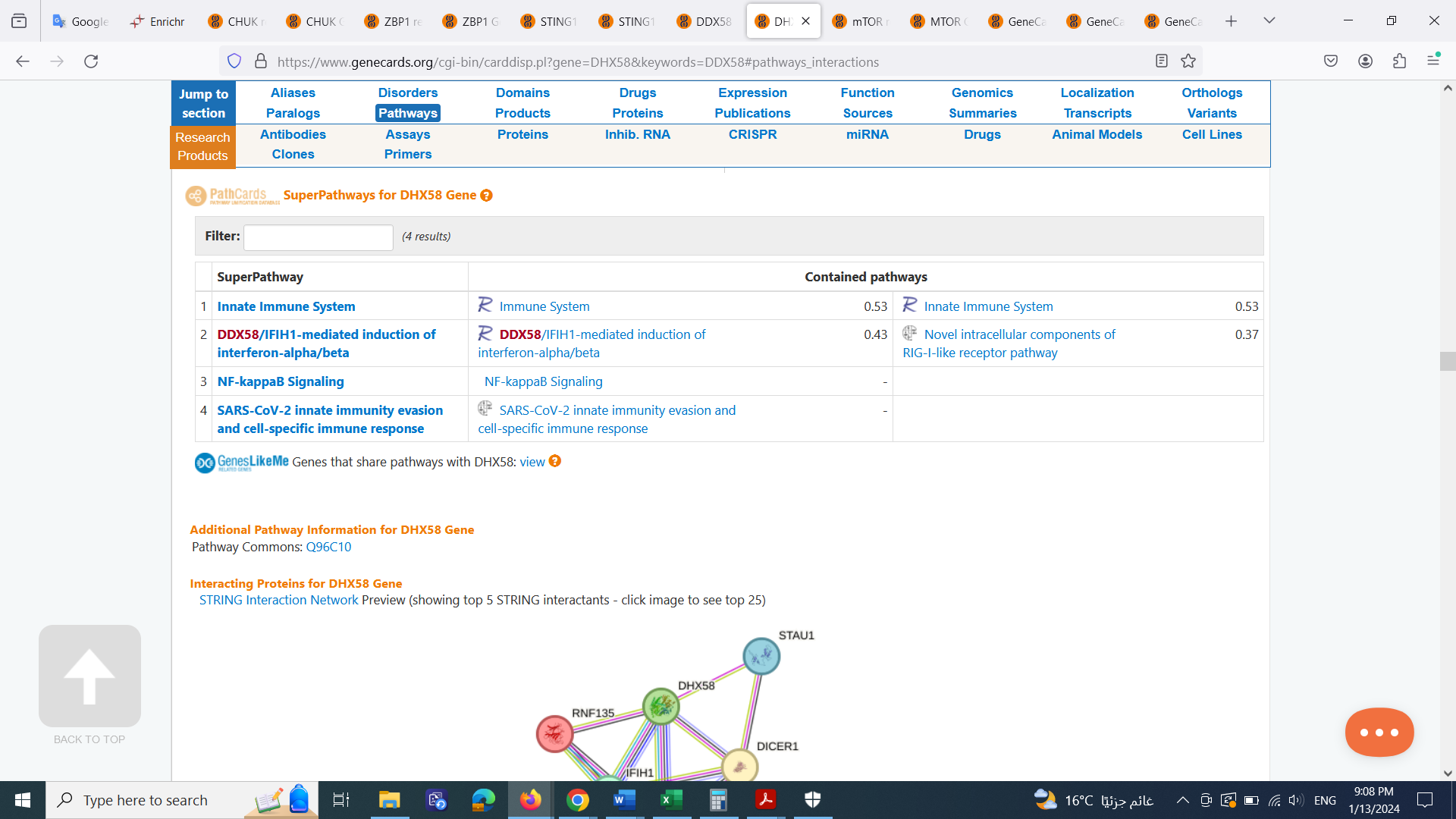


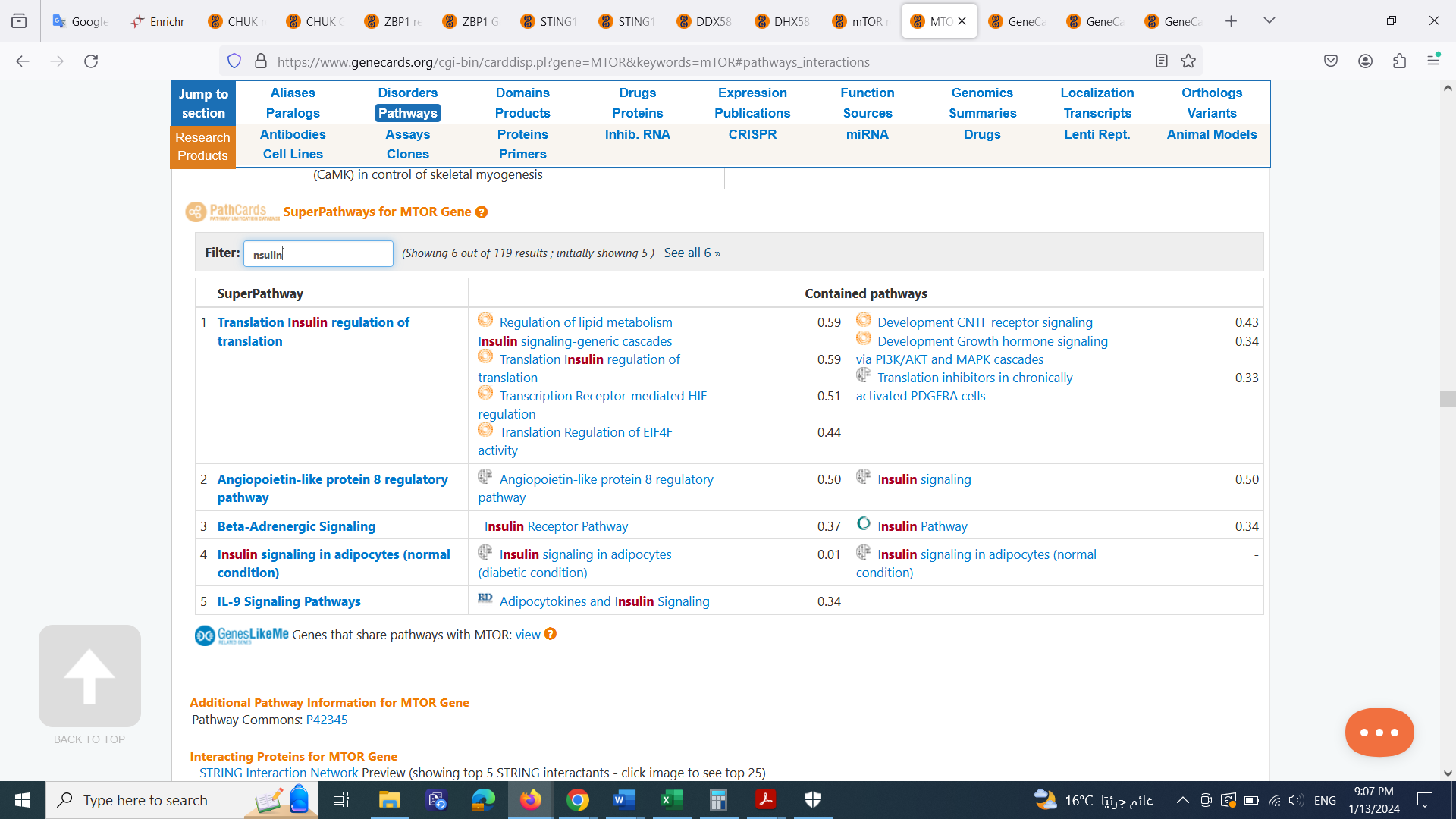


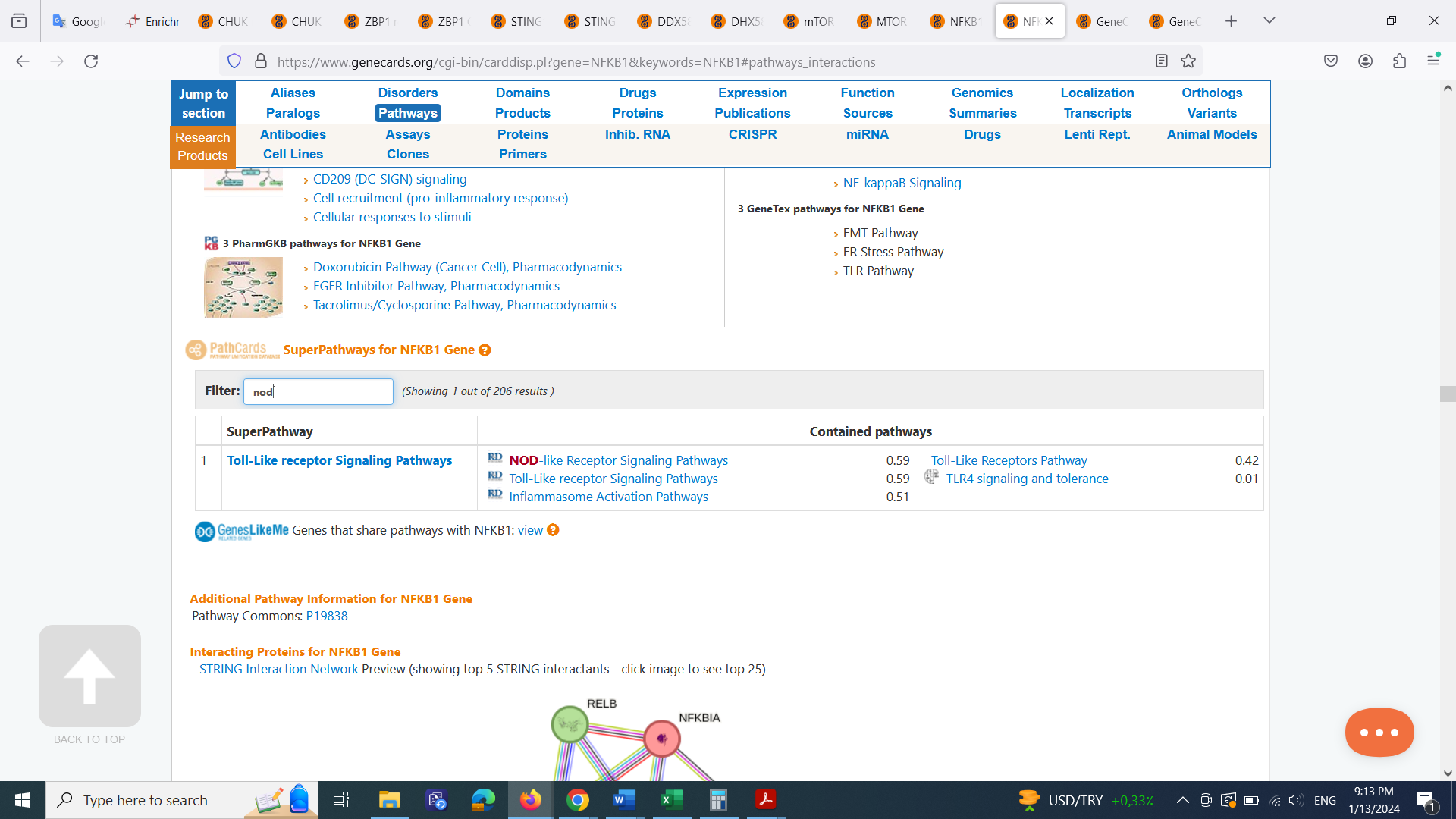


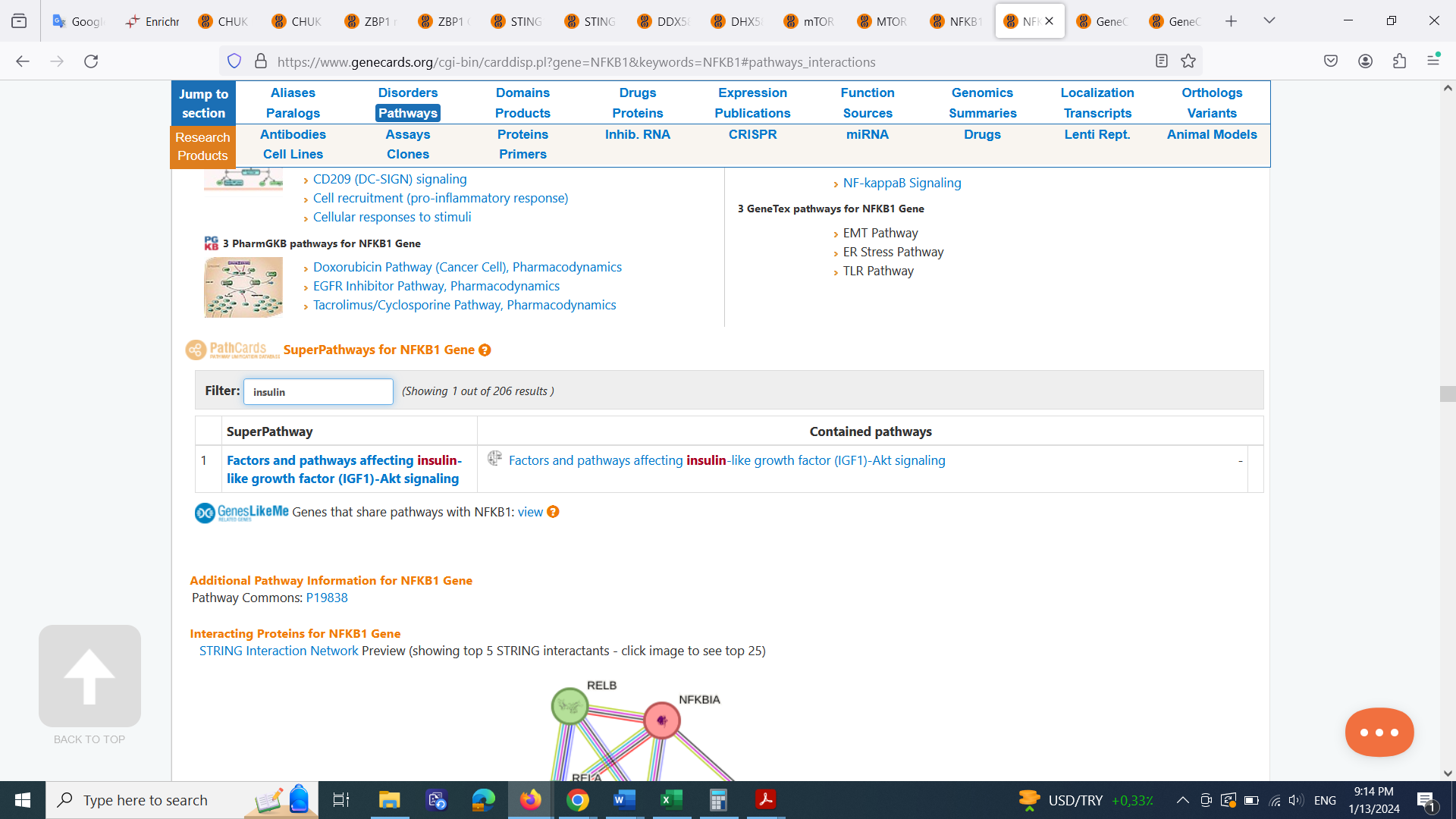


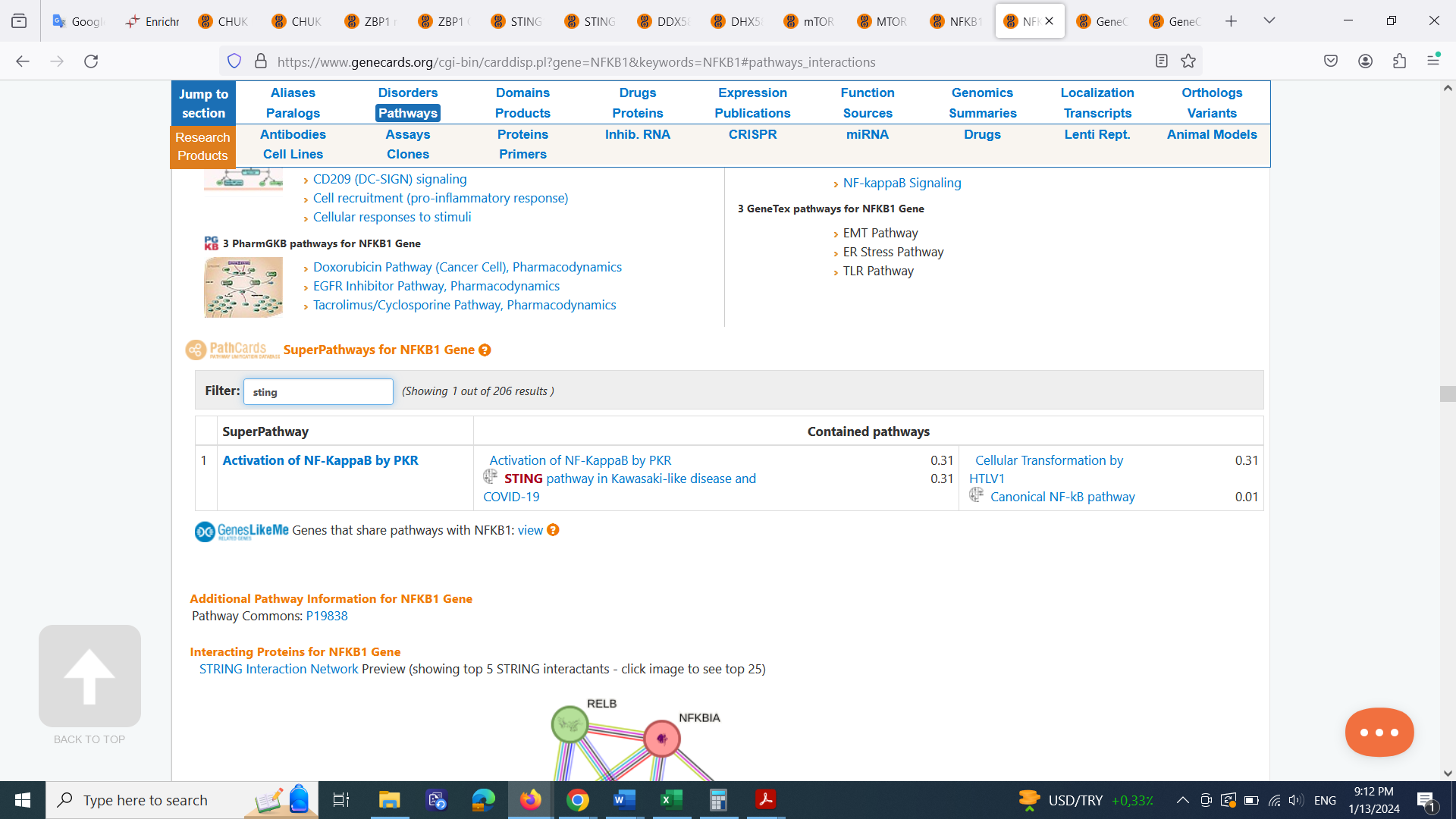


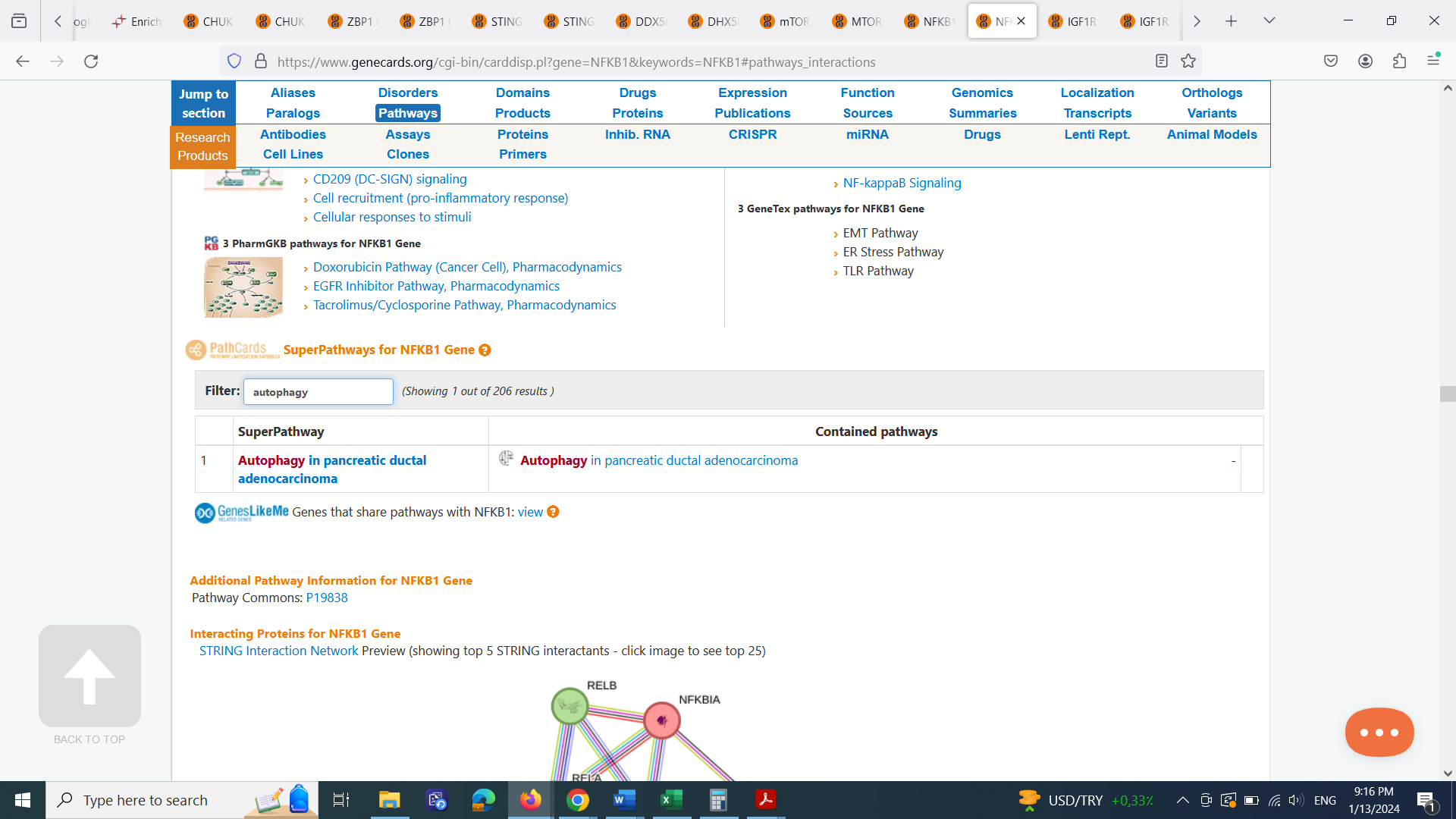


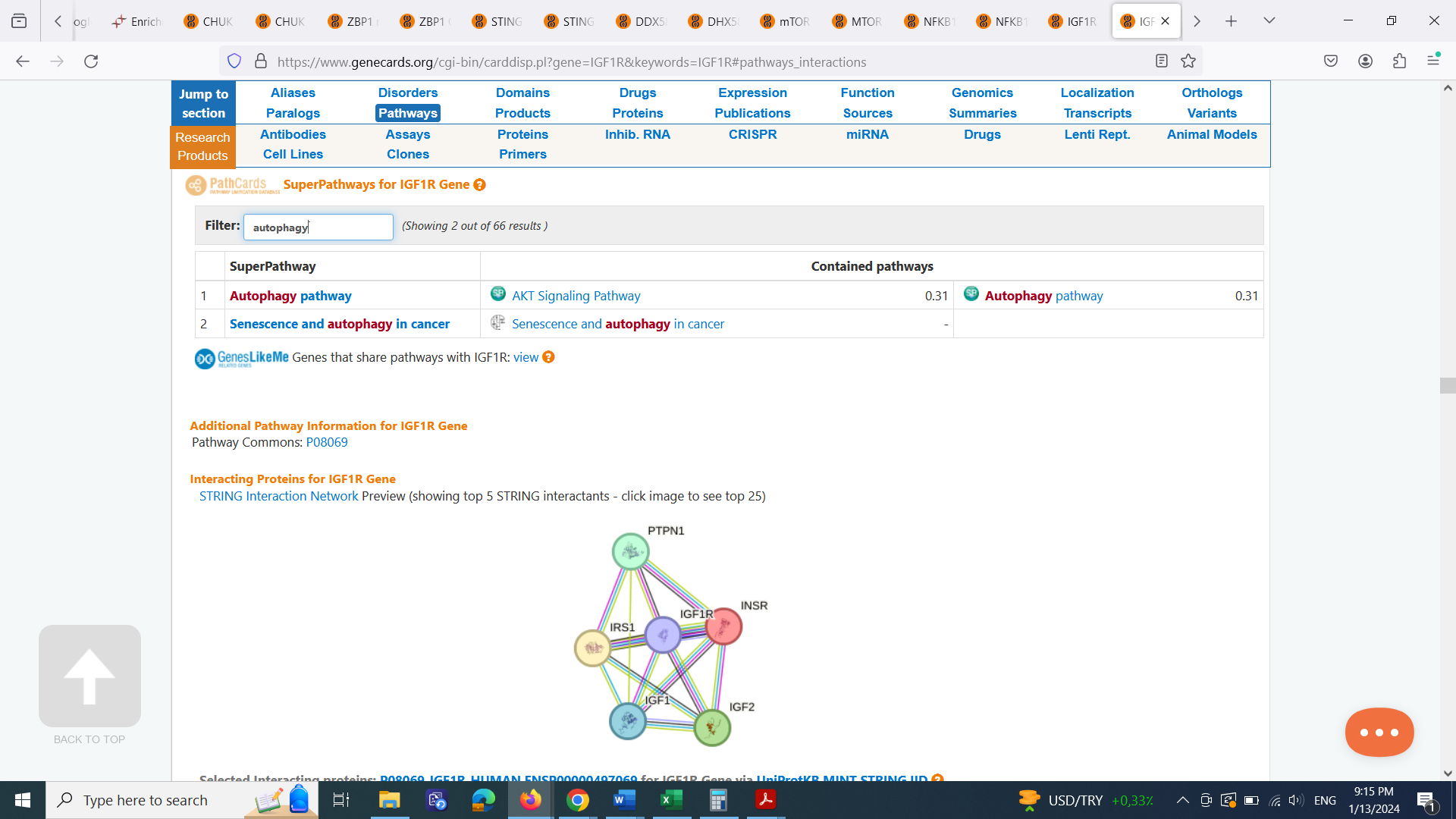


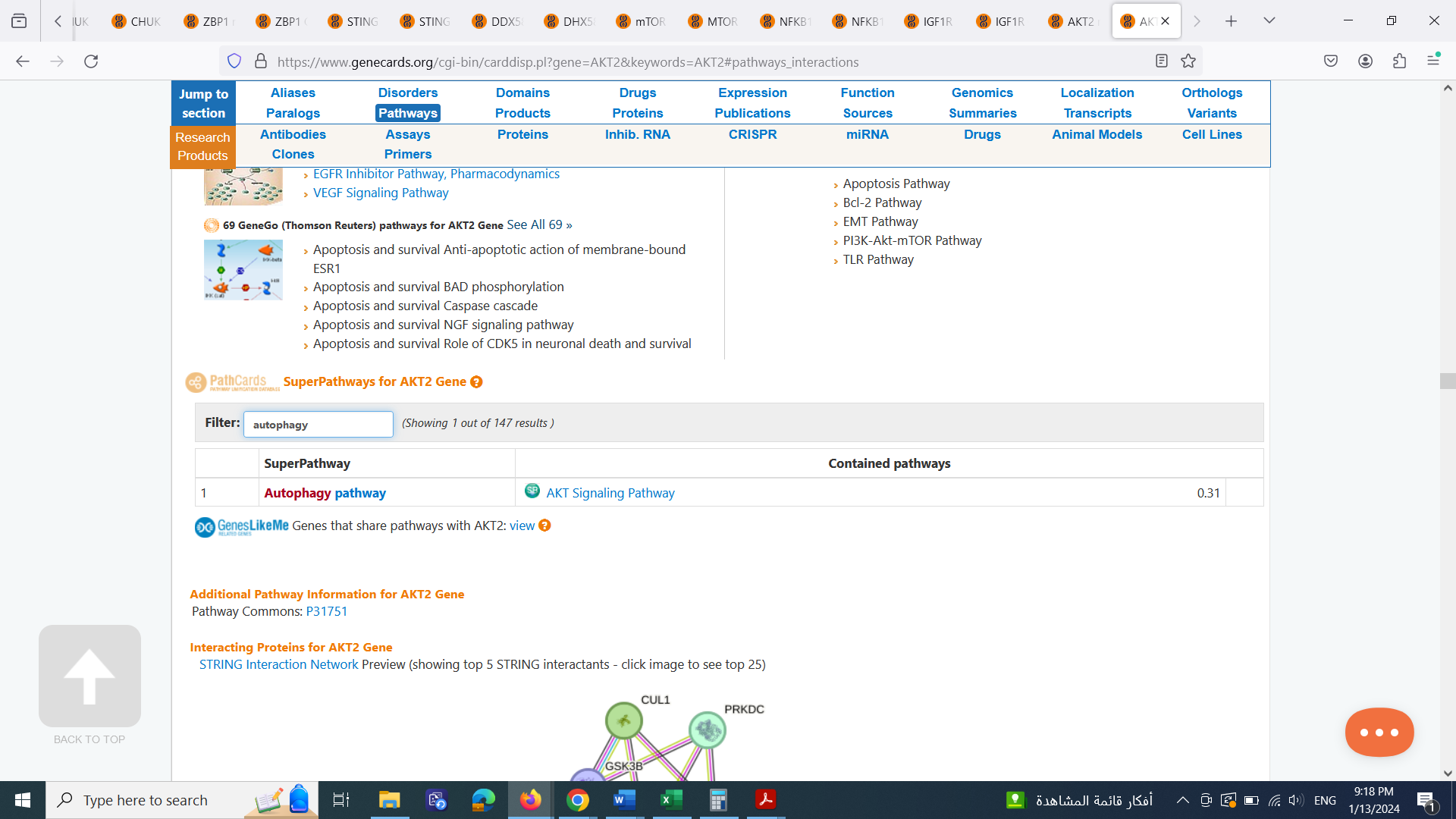


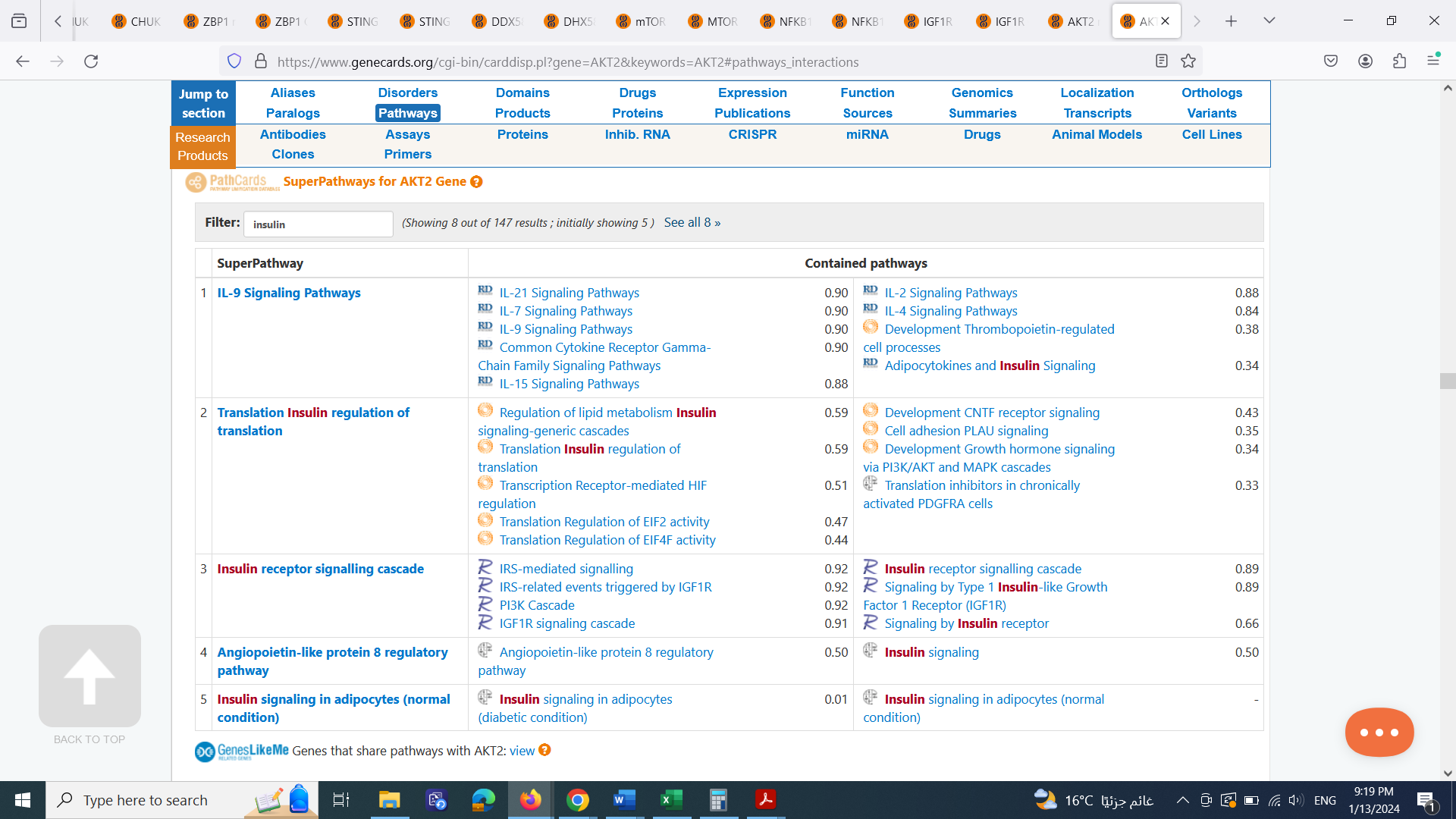


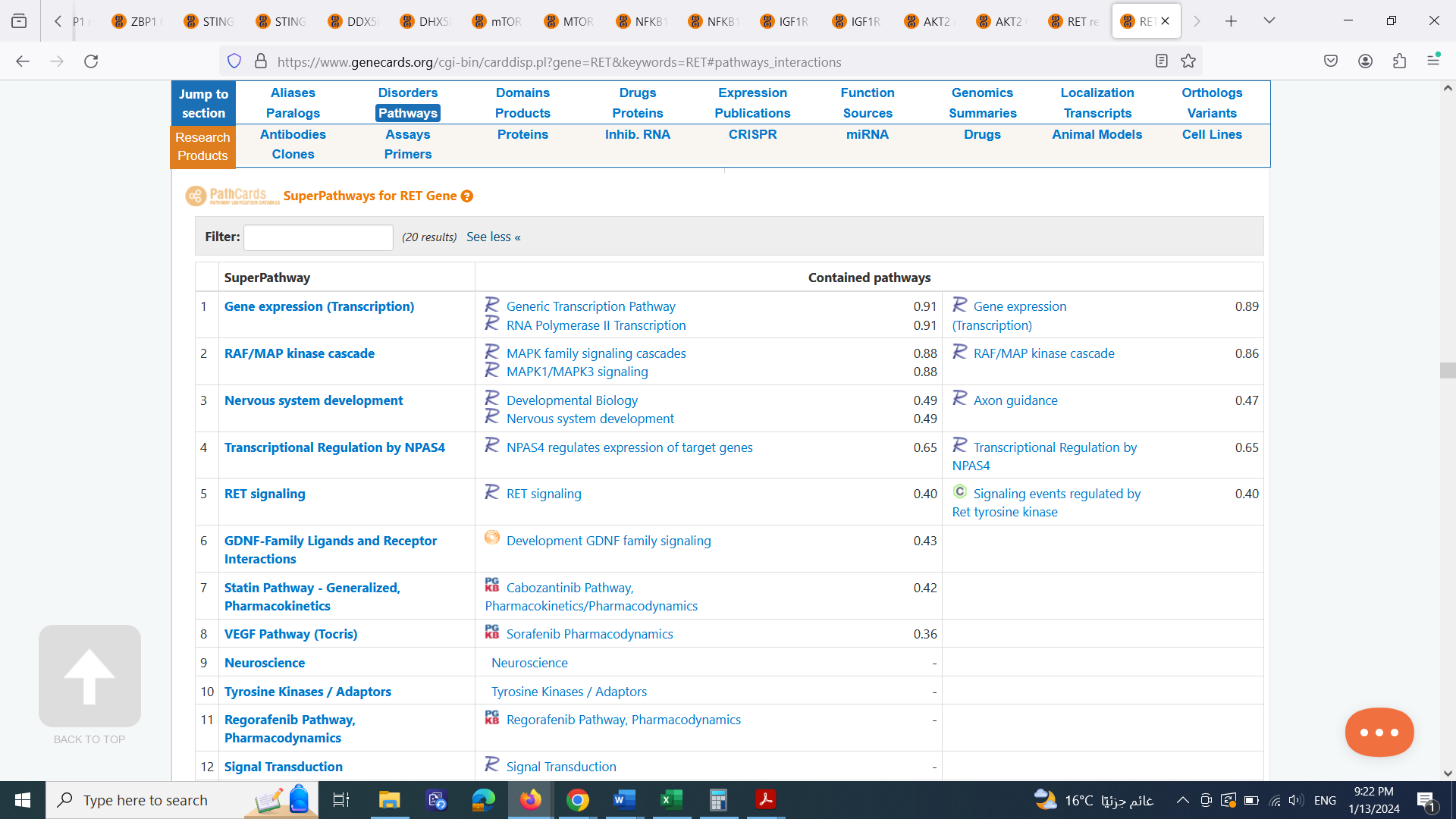


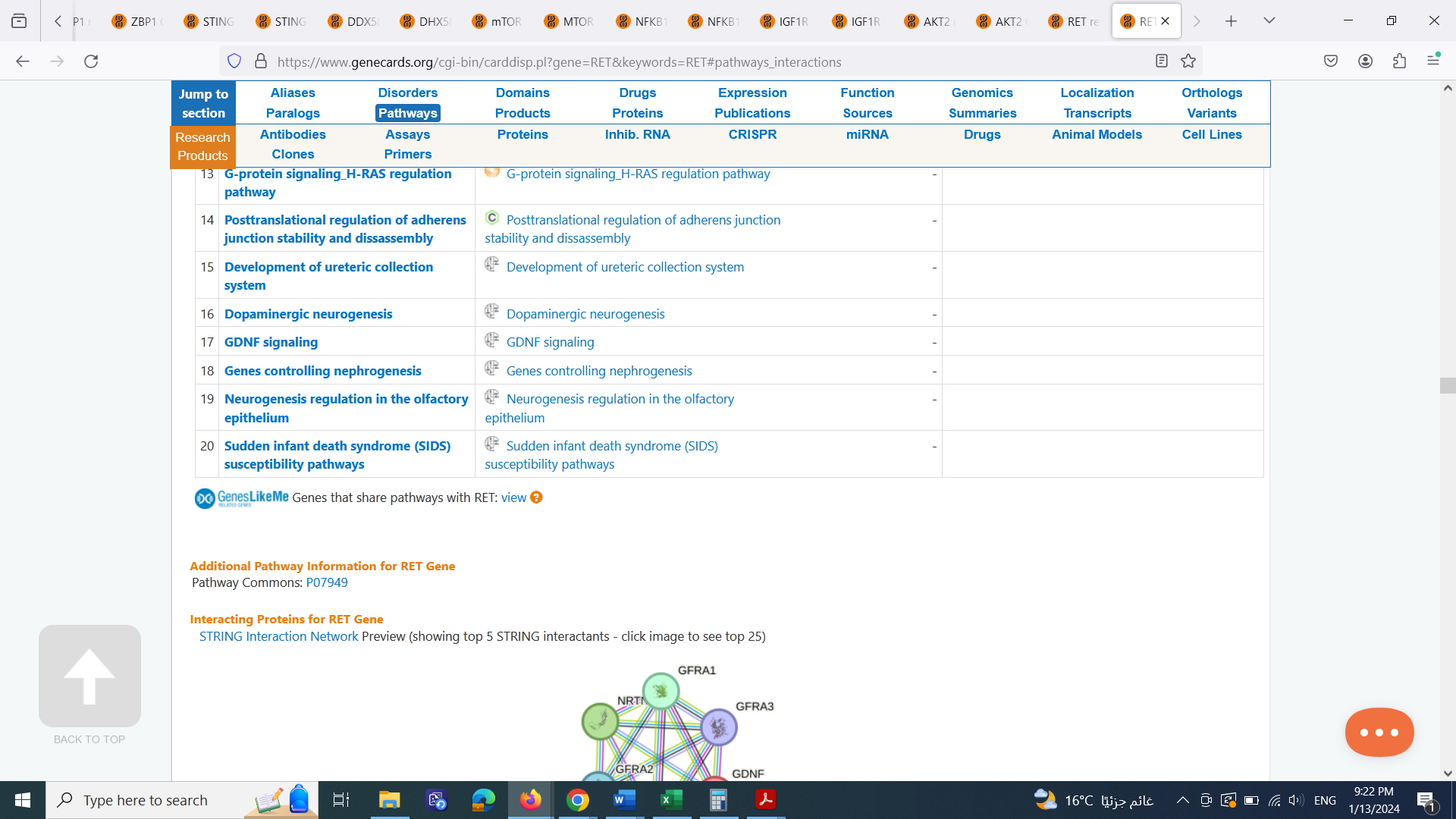


**Figure S4. The PPI interaction of the selected mRNAs using the STRING database (**[**https://string-db.org/cgi/input?sessionId=b5d8ry13LUR4&input_page_active_form=multiple_identifiers**](https://string-db.org/cgi/input?sessionId=b5d8ry13LUR4&input_page_active_form=multiple_identifiers)**, assessed on Jan 2024).**


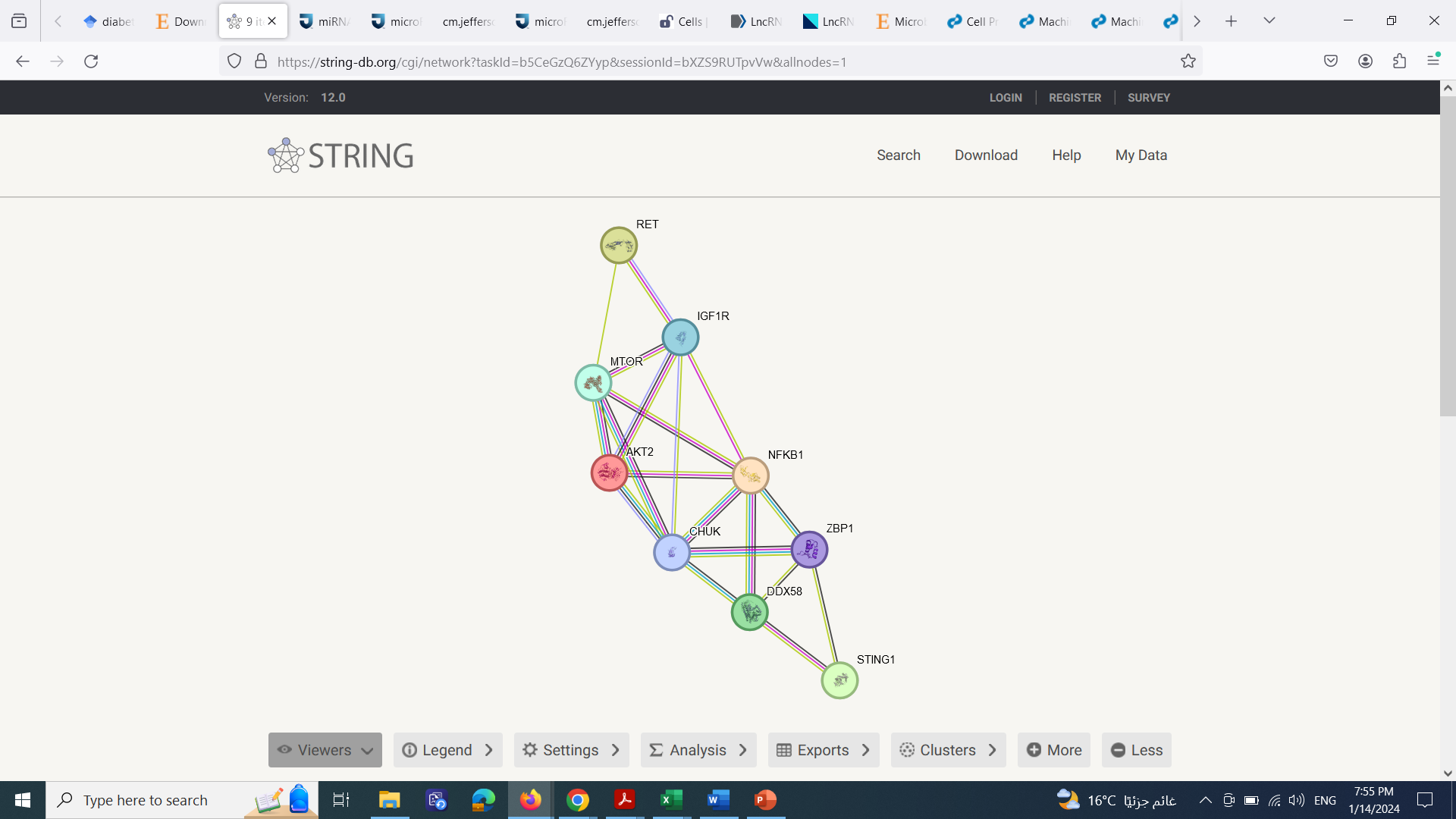


**Figure S5.: Represents the interaction between the selected mRNAs and the microRNAs with their mRNA targets using miRTarBase (**[**https://mirtarbase.cuhk.edu.cn/~miRTarBase/miRTarBase_2022/php/index.php**](https://mirtarbase.cuhk.edu.cn/~miRTarBase/miRTarBase_2022/php/index.php)**, assessed on Jan 2024 ) and** mirwalk (<http://mirwalk.umm.uni-heidelberg.de/>, **assessed on Jan 2024**)


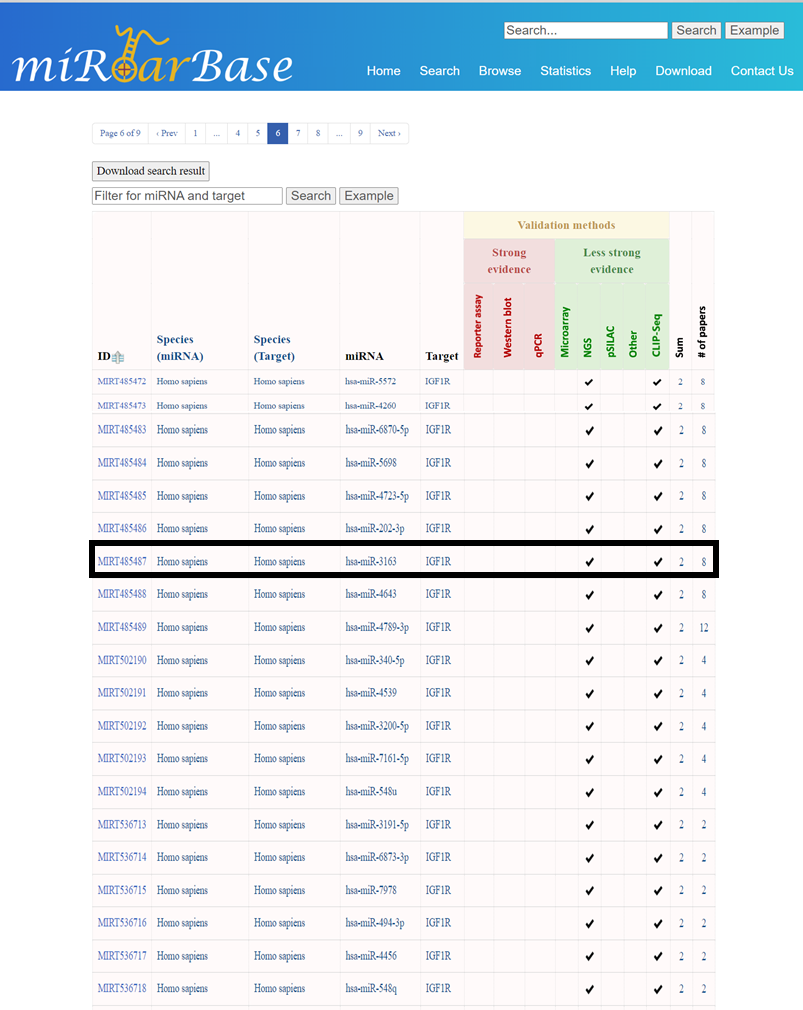


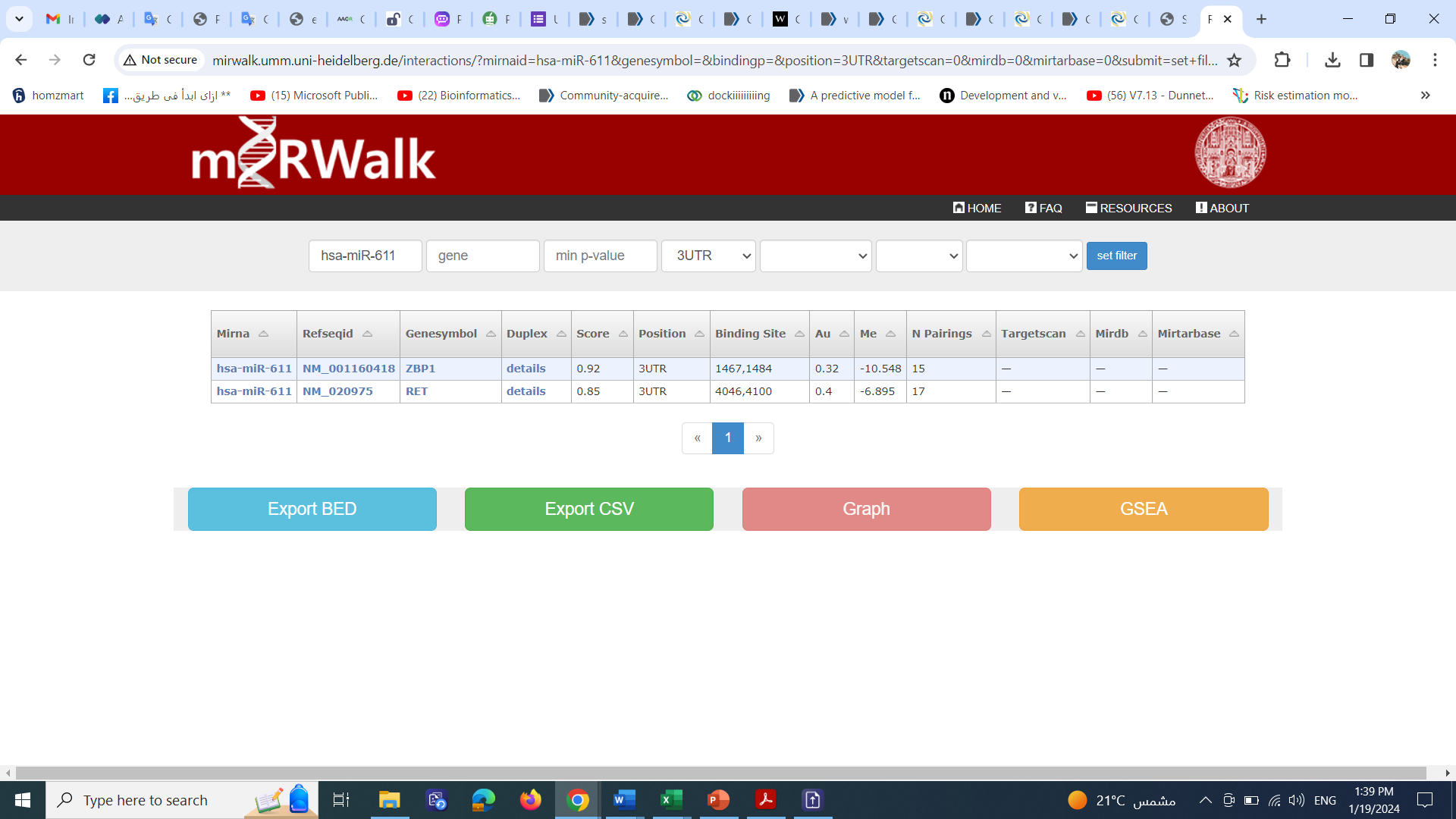


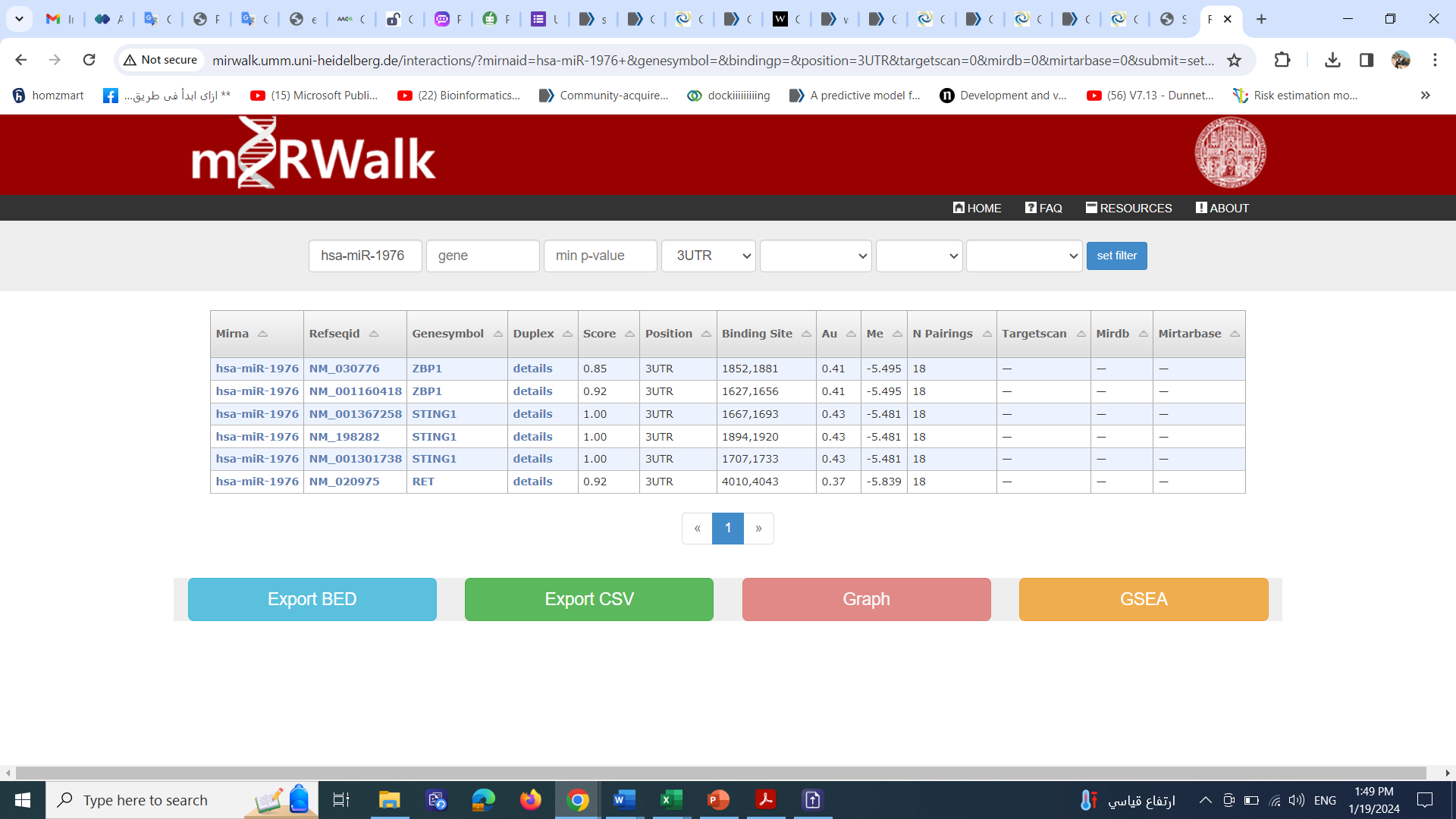


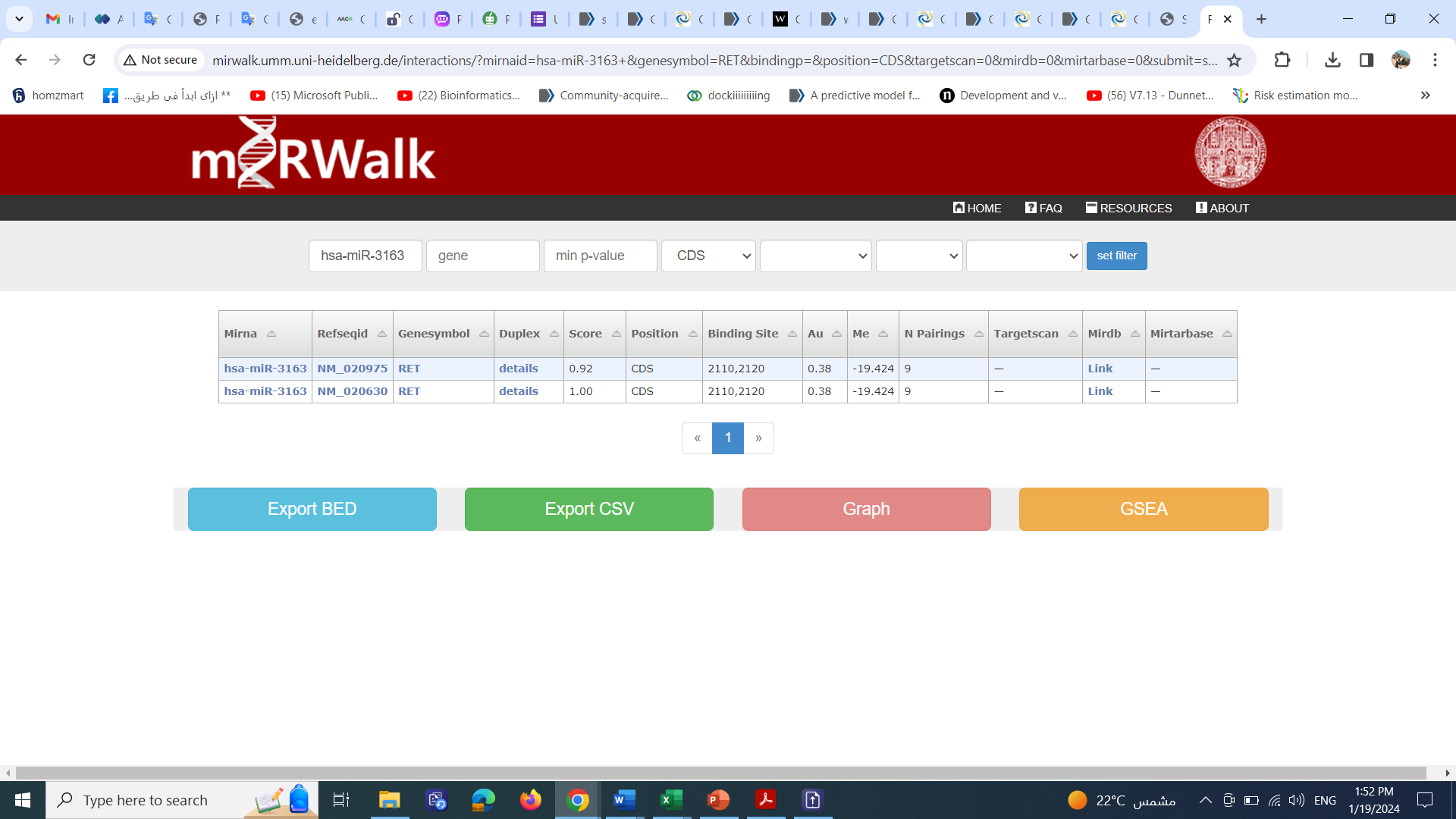


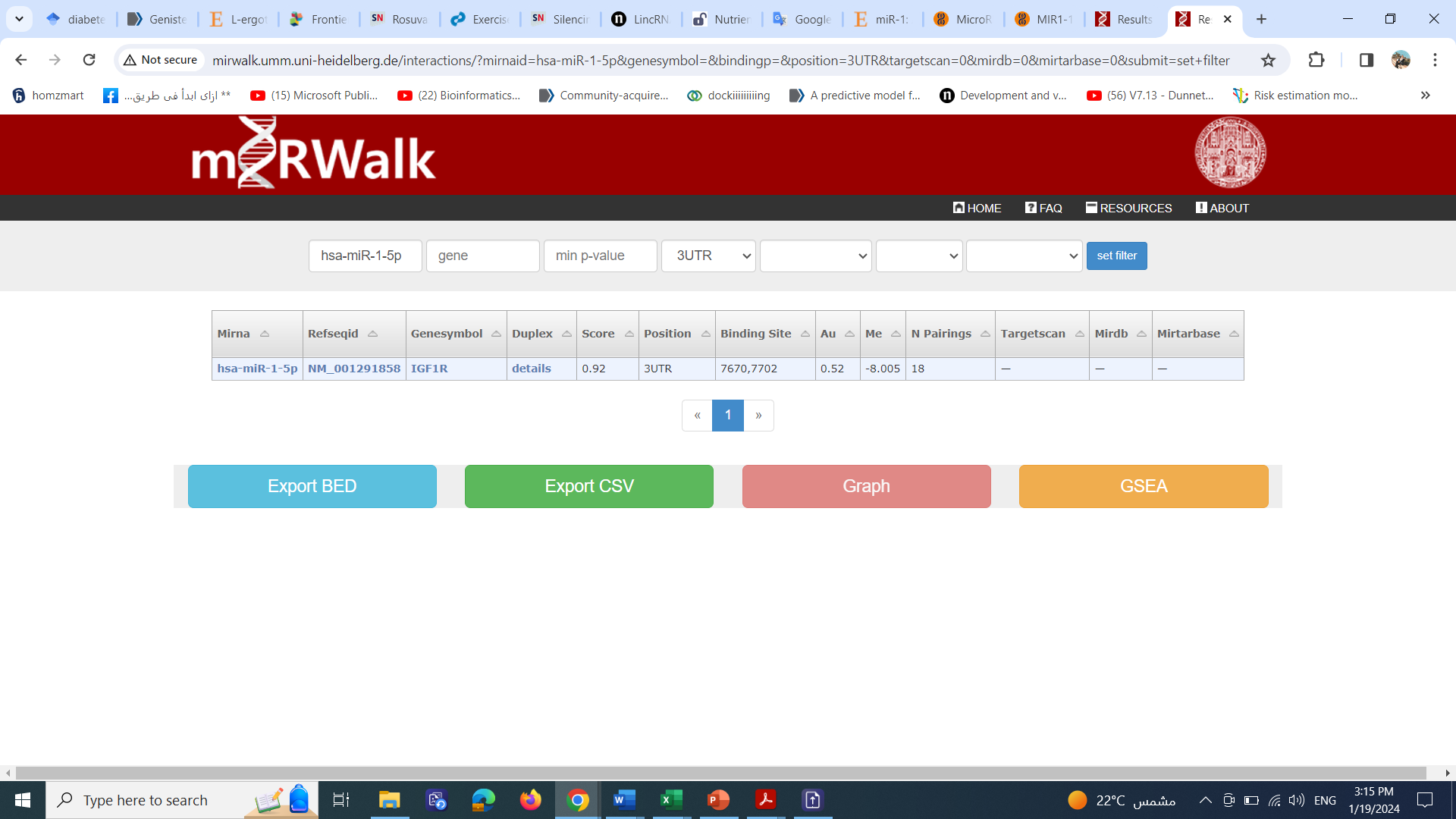


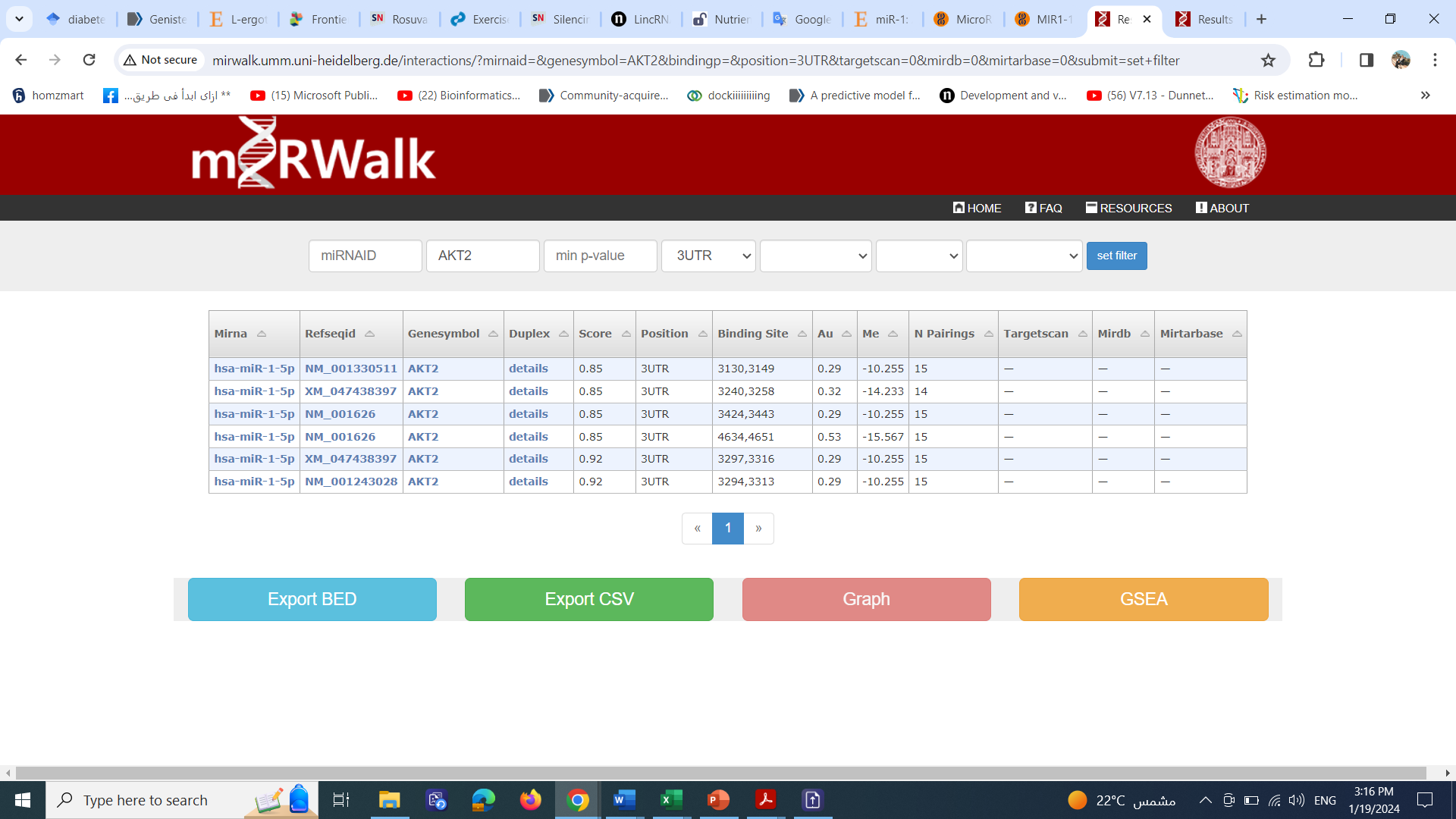


**Figure S6.: Represents the interaction of miRNAs-LncRNAs, using RNA22 (**[**https://cm.jefferson.edu/rna22/**](https://cm.jefferson.edu/rna22/)**, accessed on Jan 2024) and DIANA Tools (**[**https://diana.e-ce.uth.gr/lncbasev3/interactions**](https://diana.e-ce.uth.gr/lncbasev3/interactions)**, accessed on Jan 2024), and mirwalk database (**[**http://mirwalk.umm.uni-heidelberg.de/**](http://mirwalk.umm.uni-heidelberg.de/)**, accessed on Jan 2024)
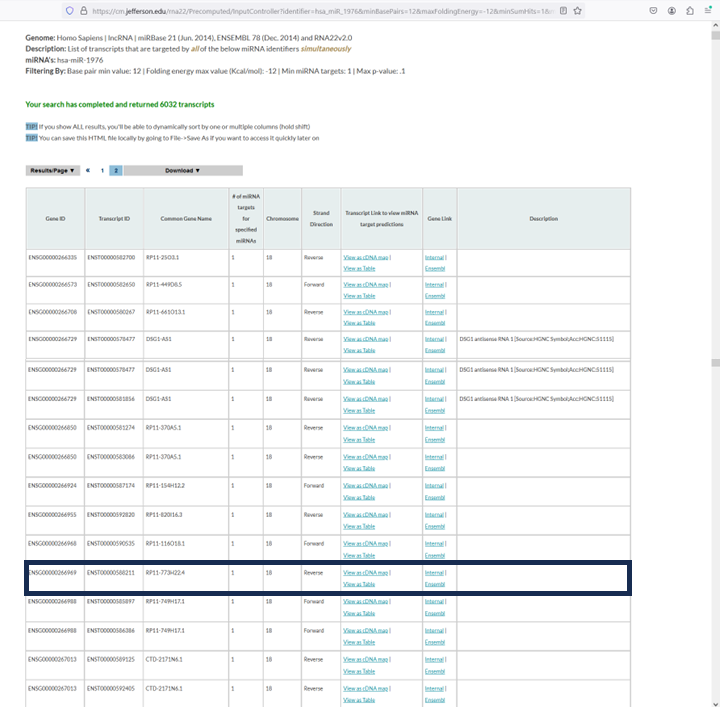
**


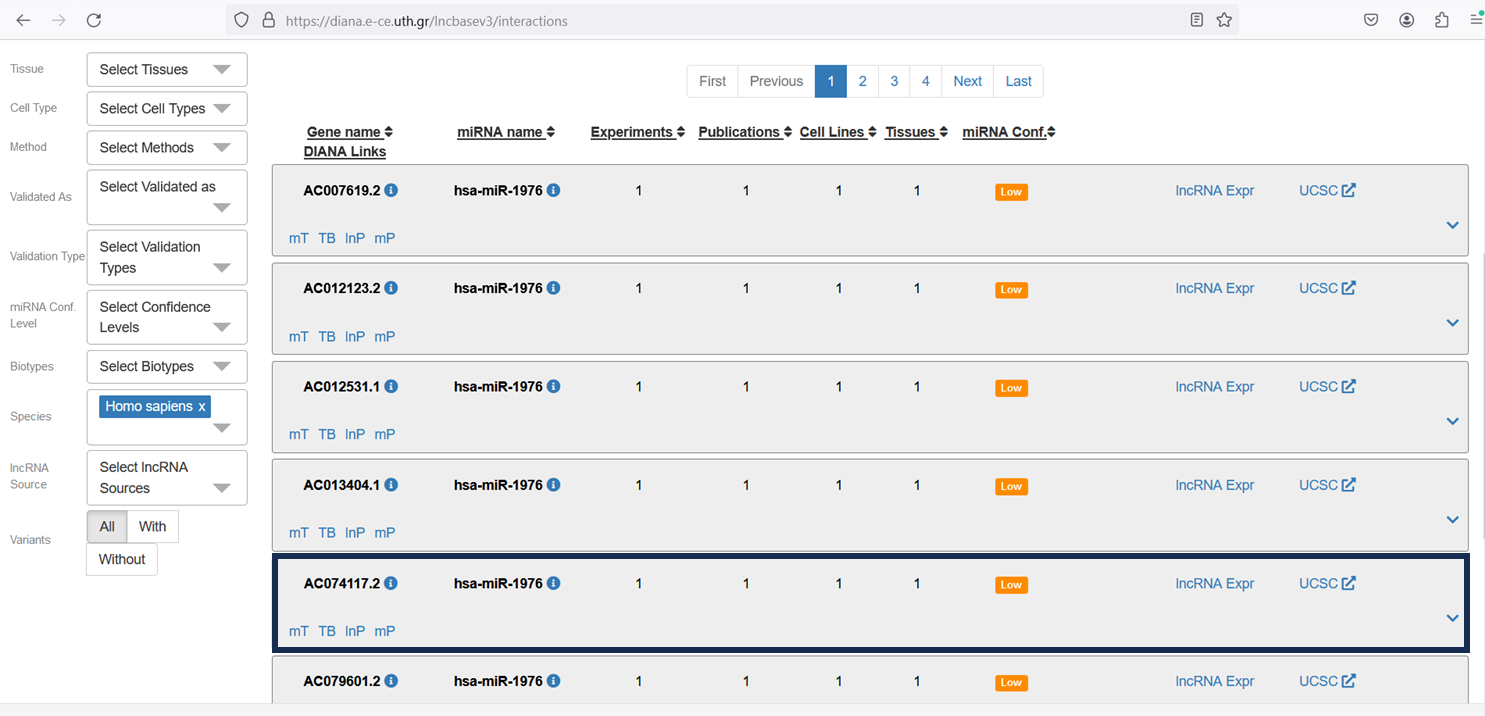


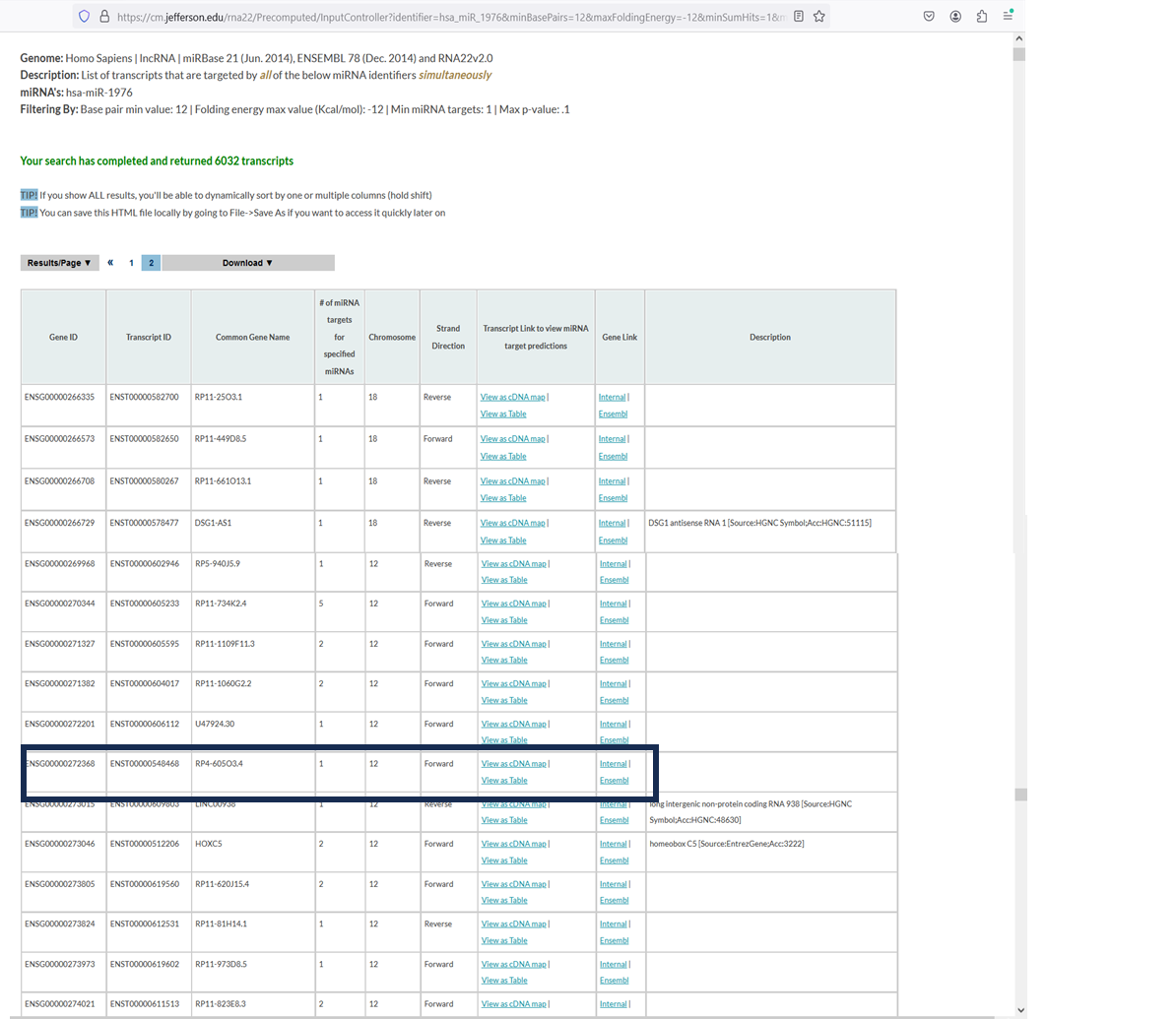


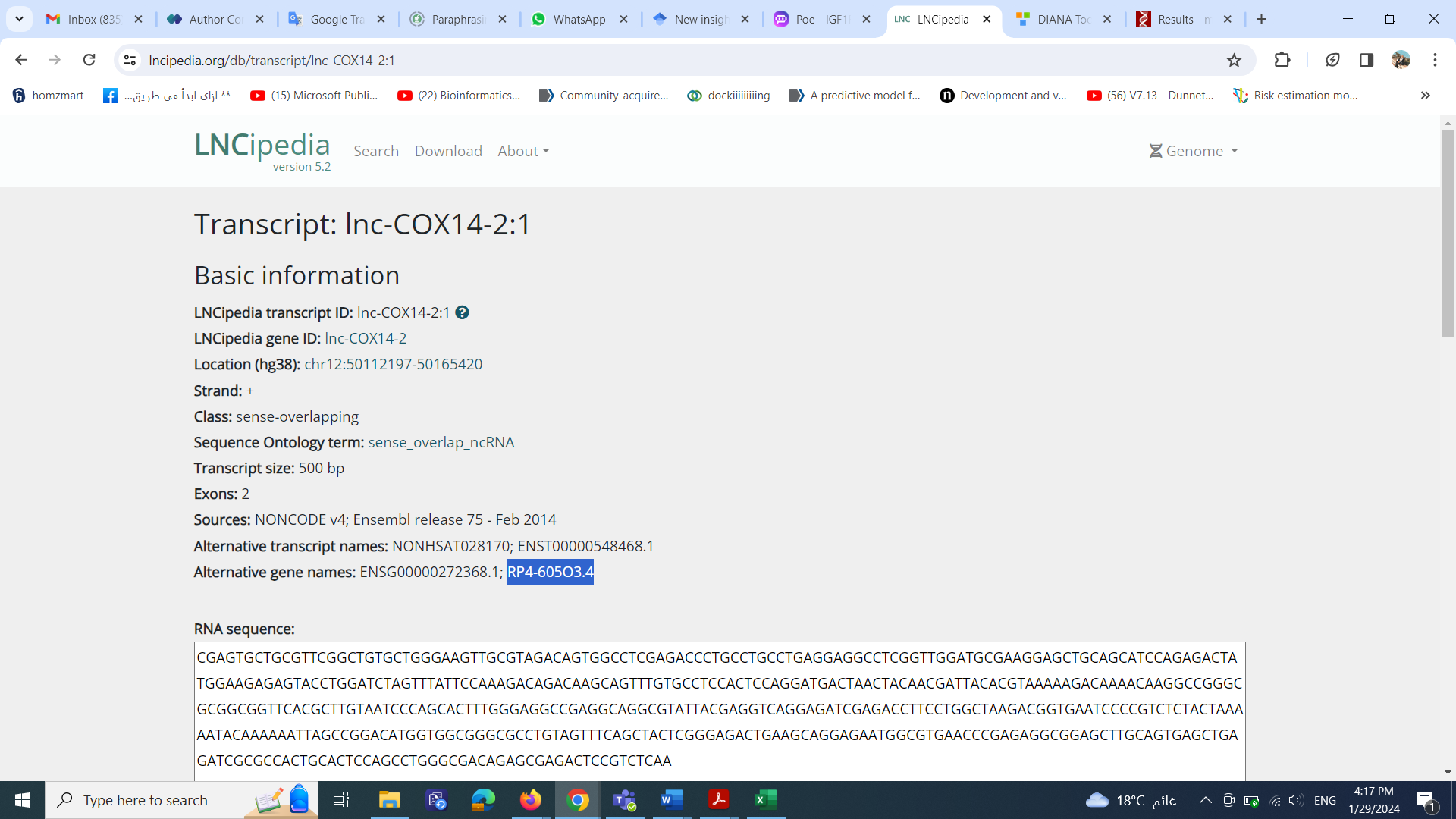


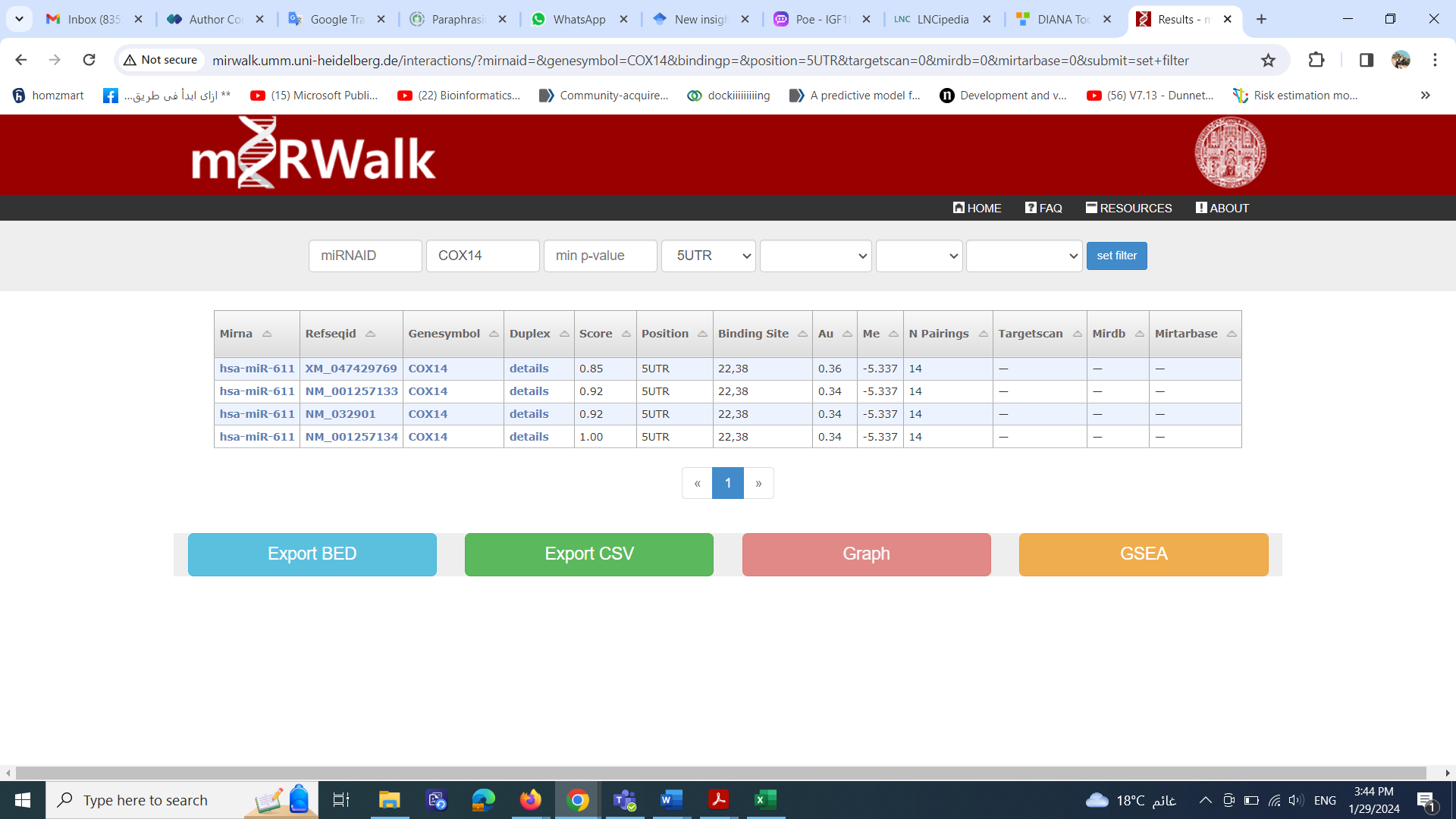


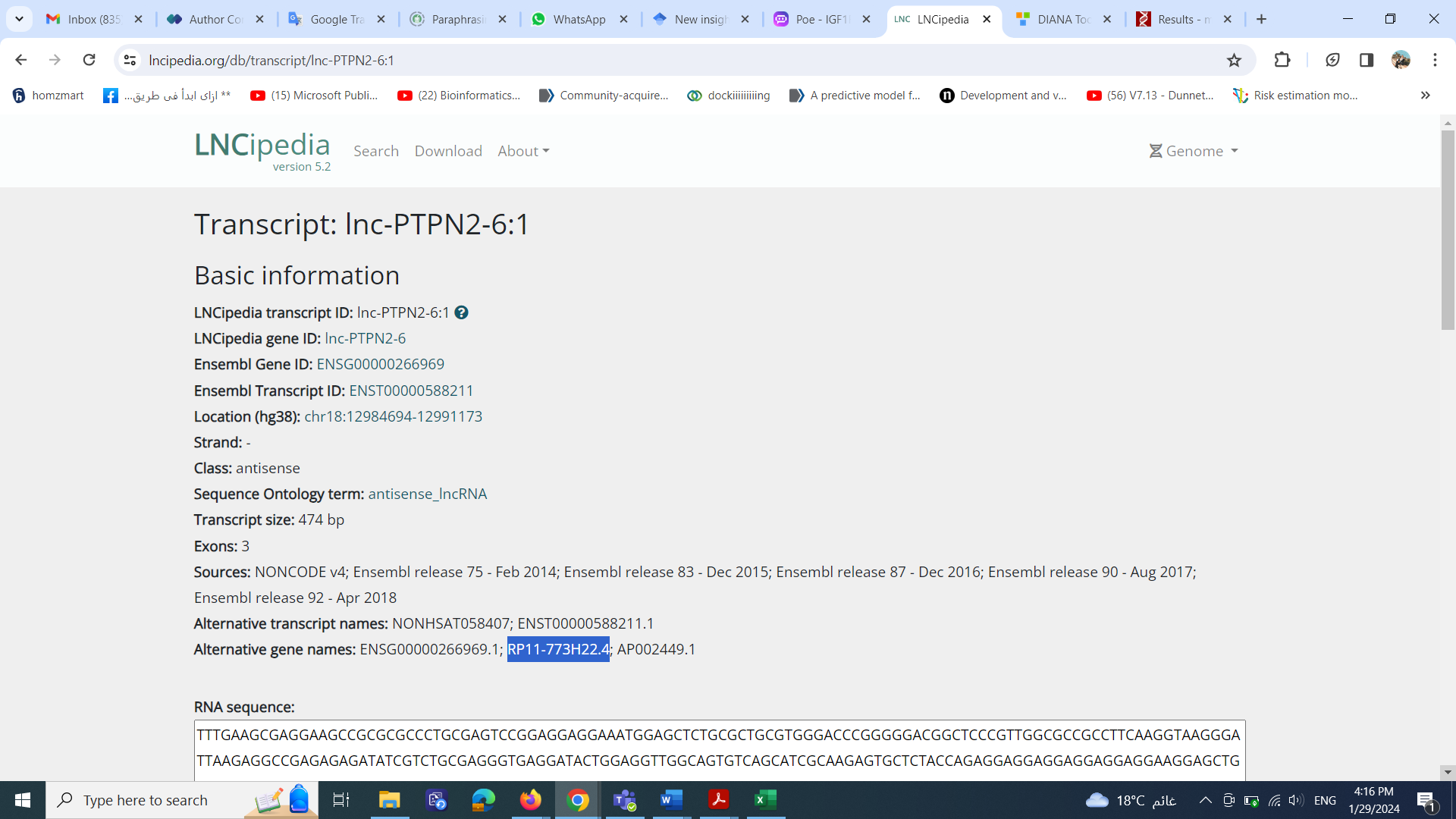


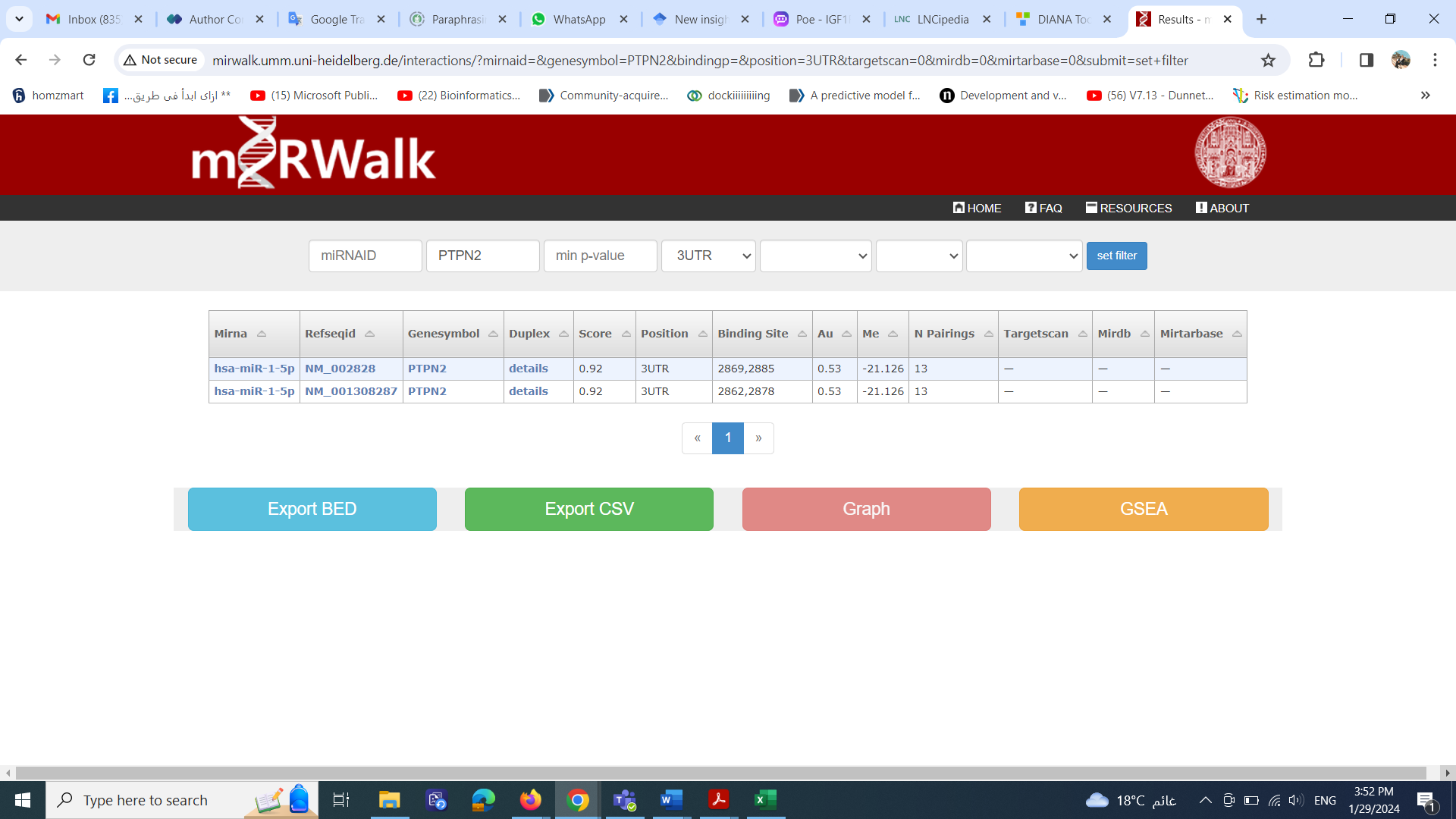

Supplement: Supplementary file 1 [file DataSheet_1.docx]
