# Supplementary figures and images for: Comprehensive machine learning models for predicting therapeutic targets in type 2 diabetes utilizing molecular and biochemical features in rats
Source: Front Endocrinol (Lausanne). 2024 May 24;15:1384984. doi: 10.3389/fendo.2024.1384984 (PMC11157016; doi:10.3389/fendo.2024.1384984)

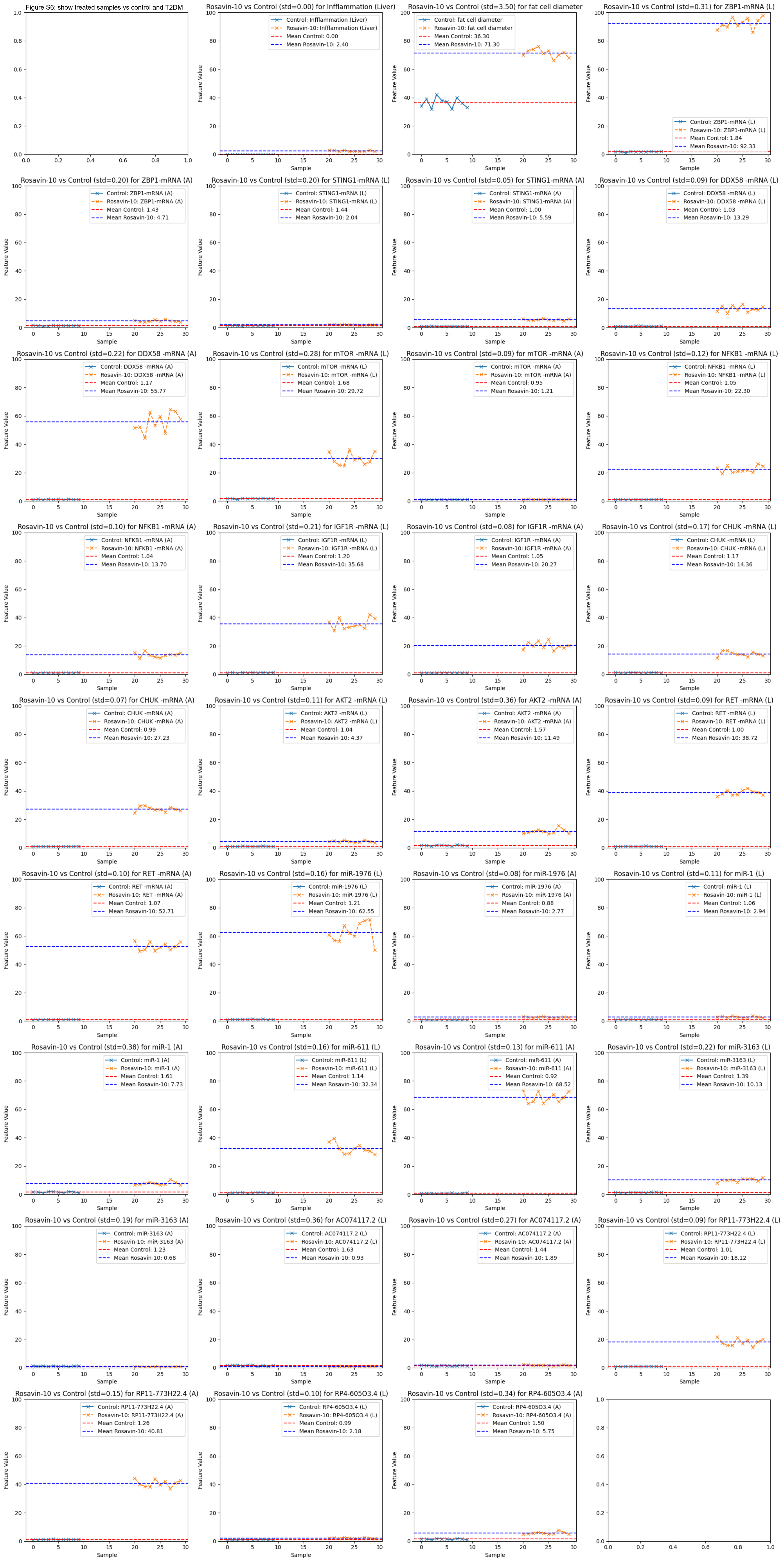

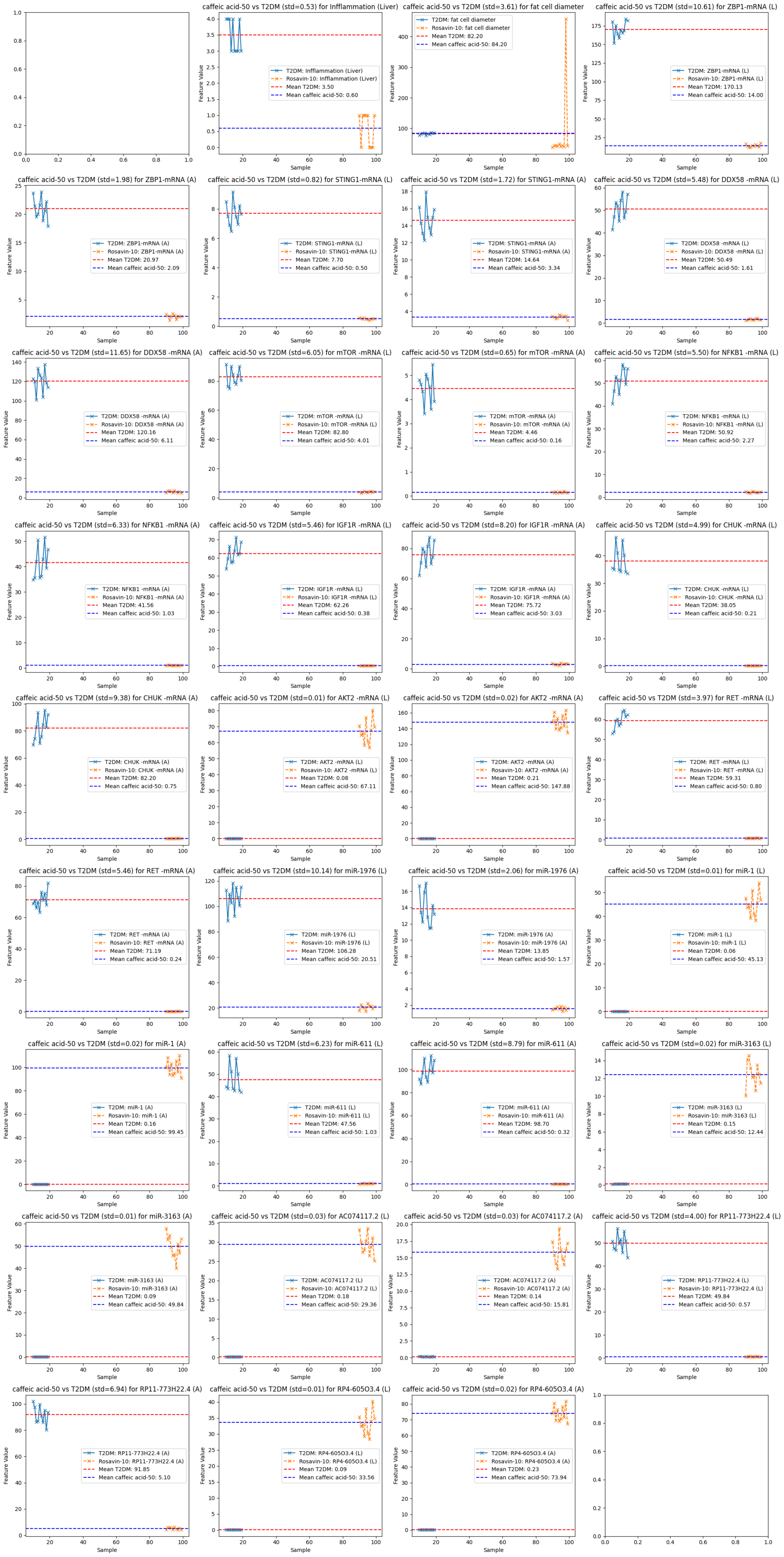

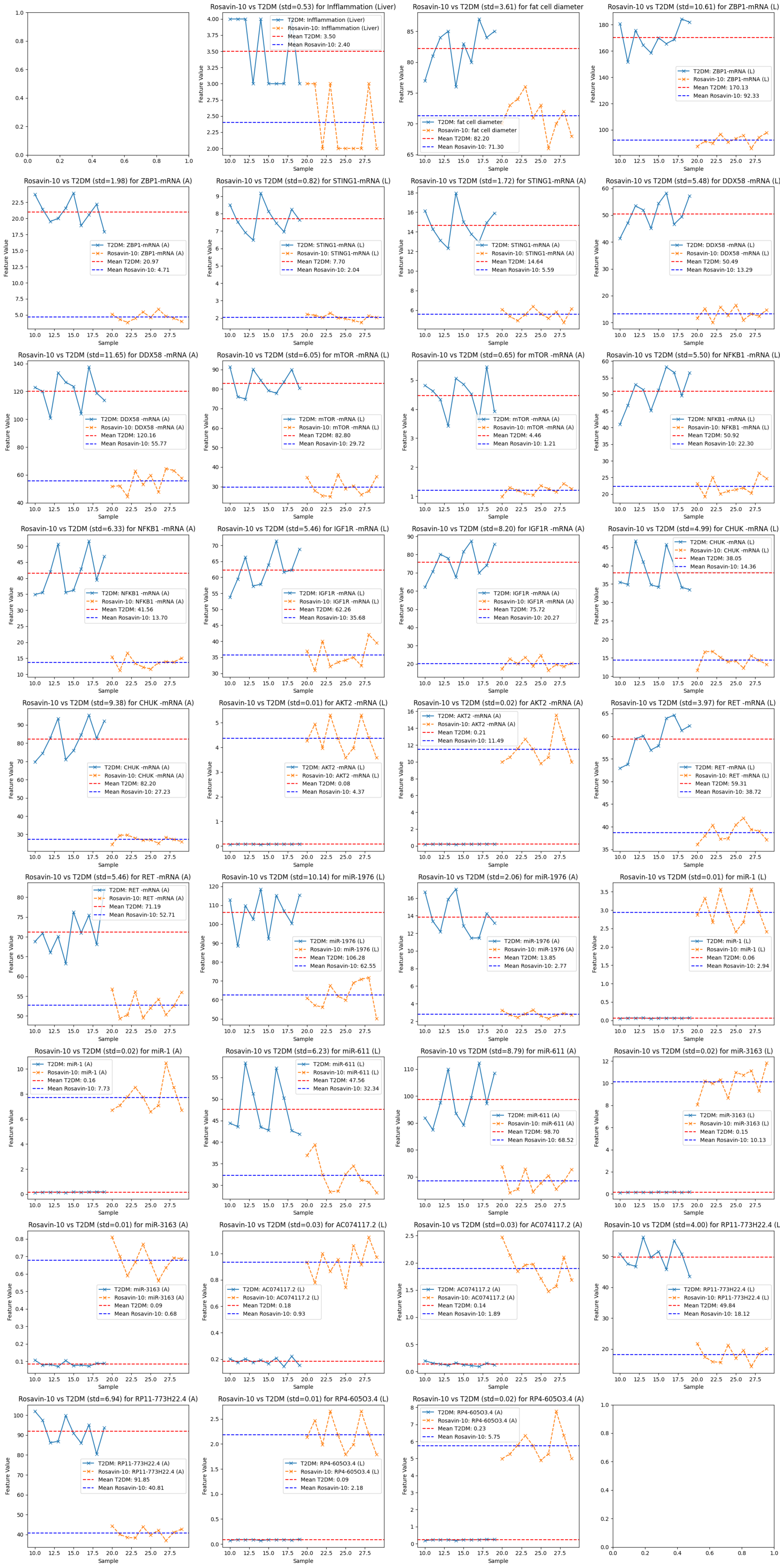

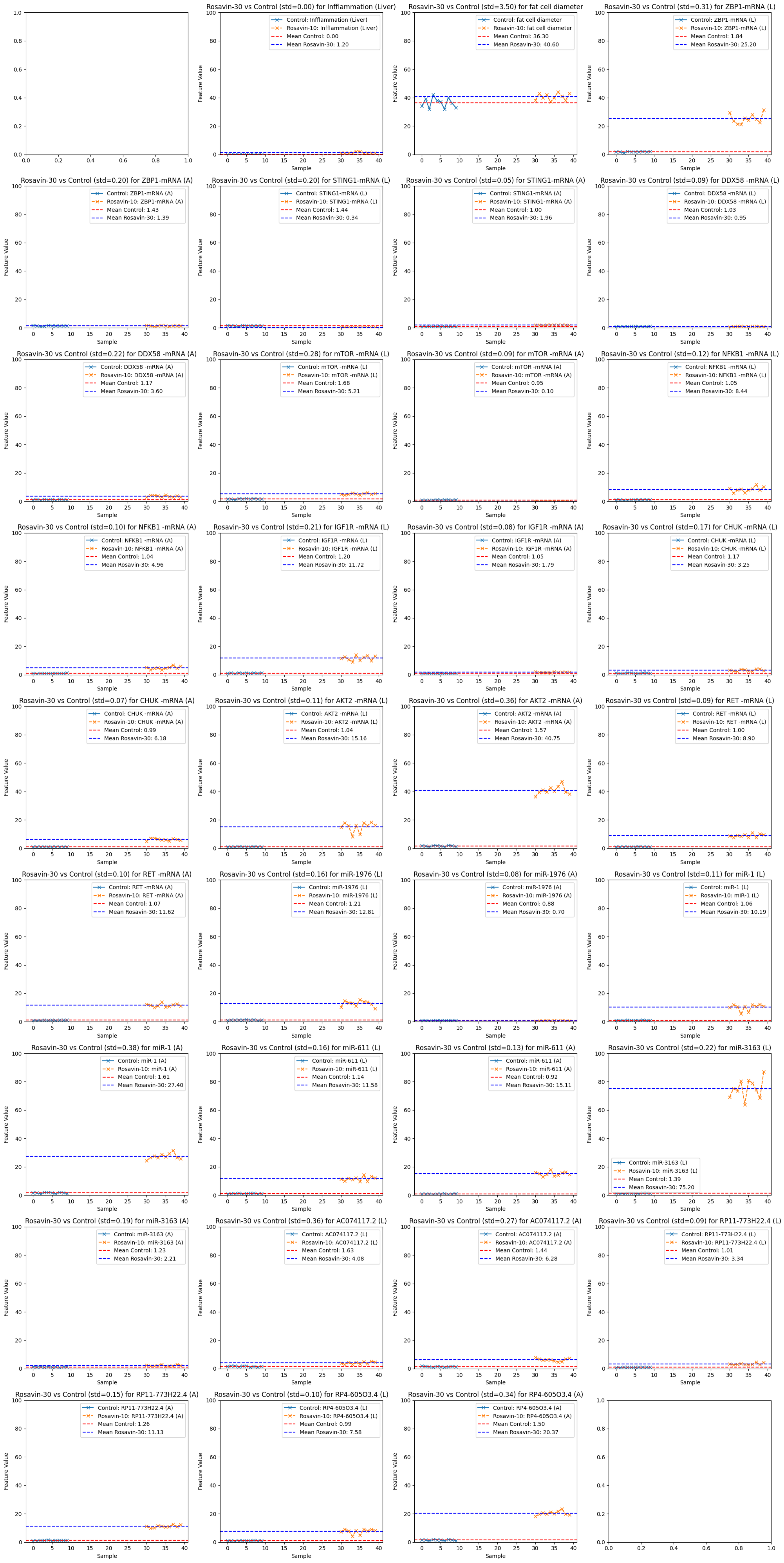

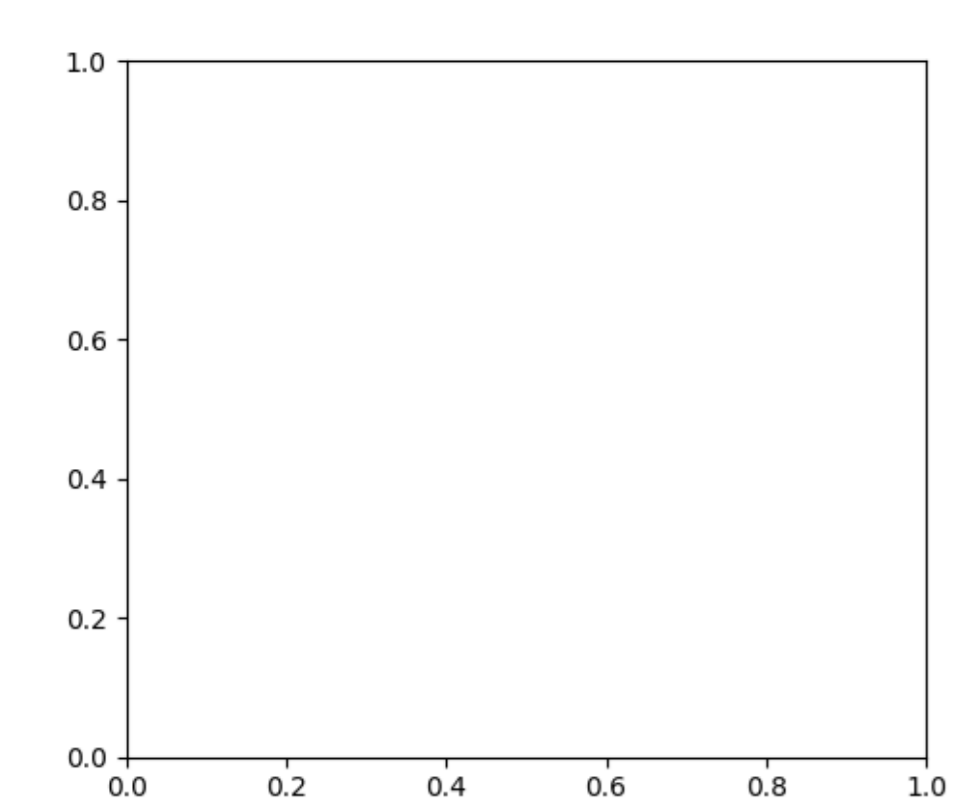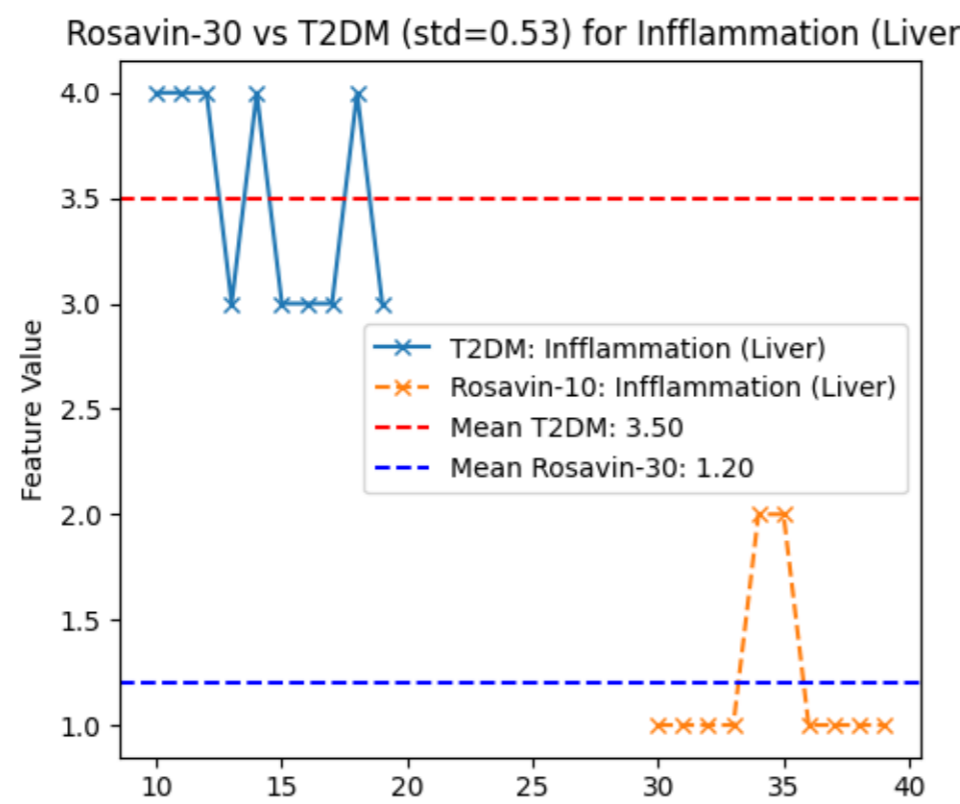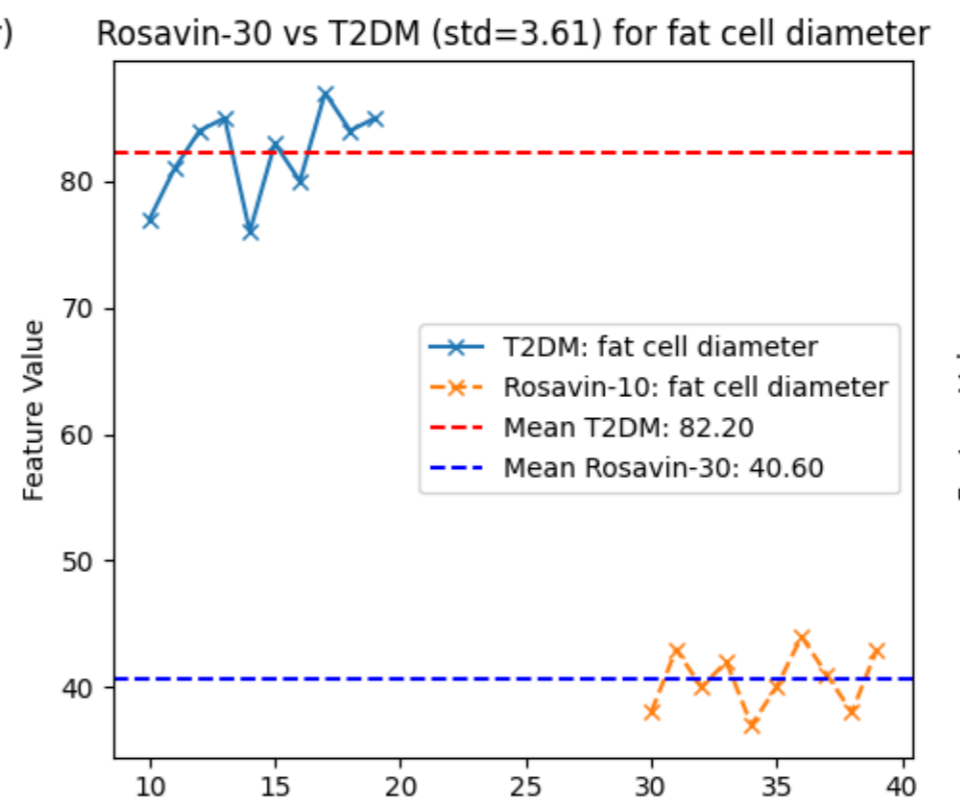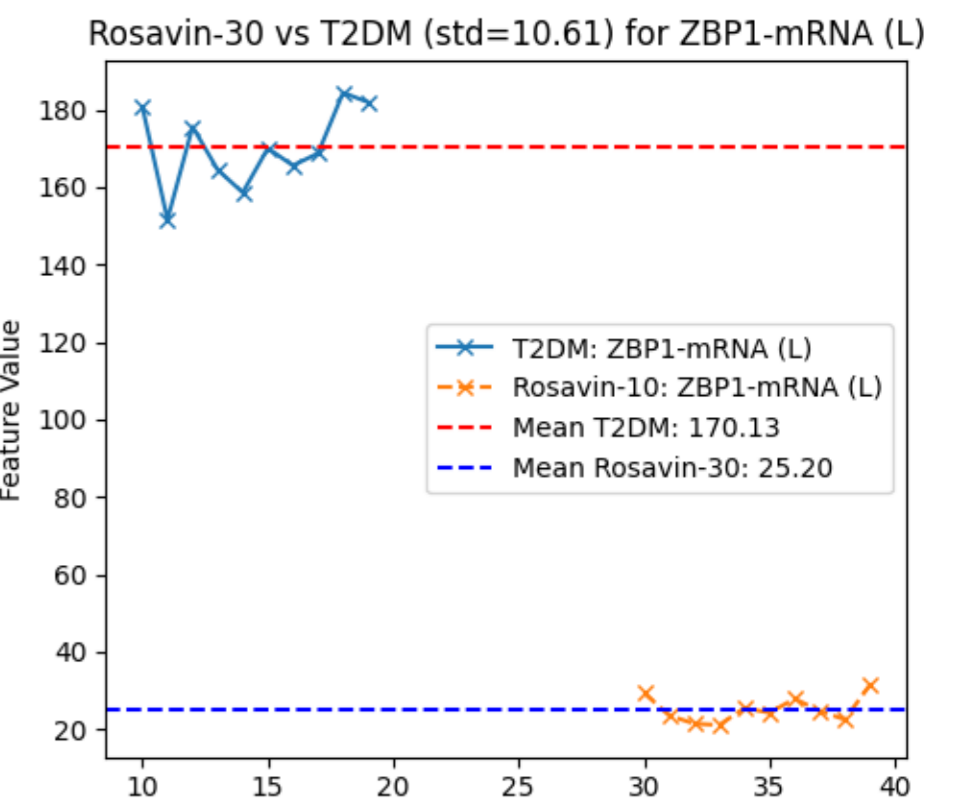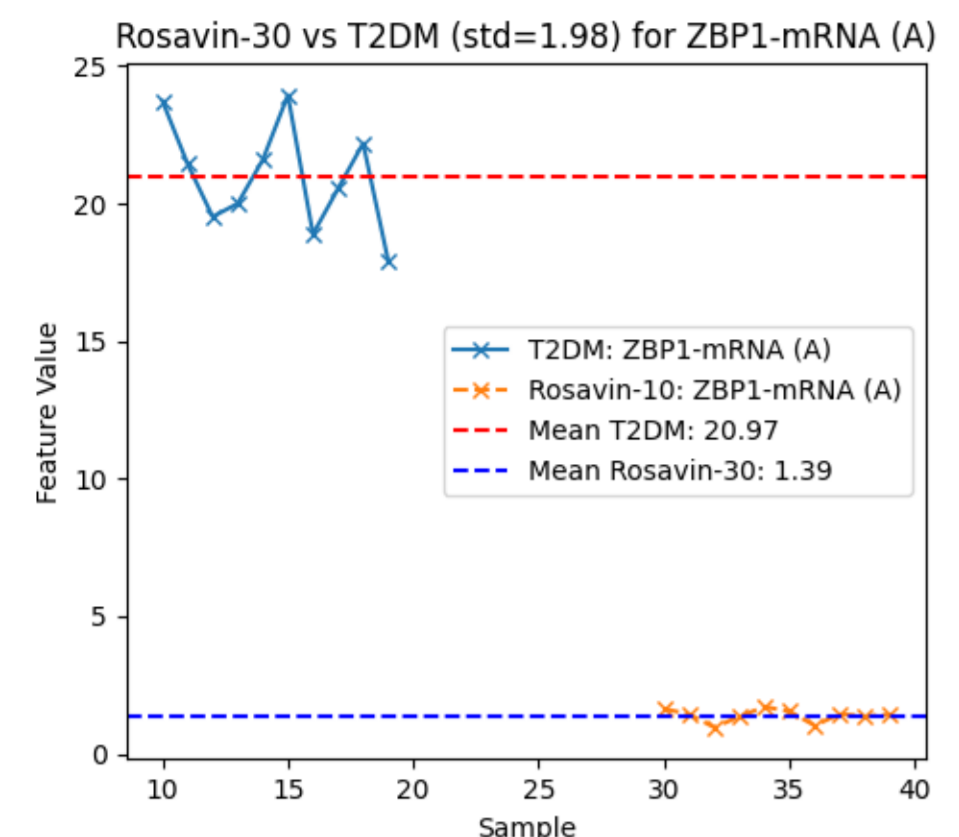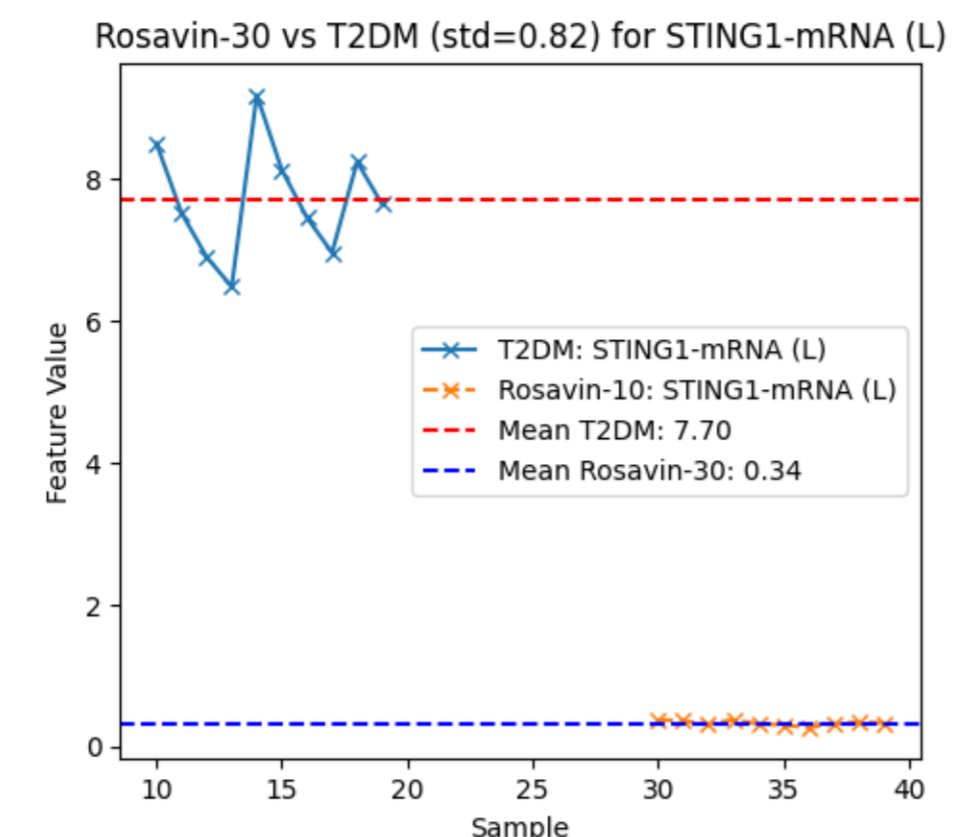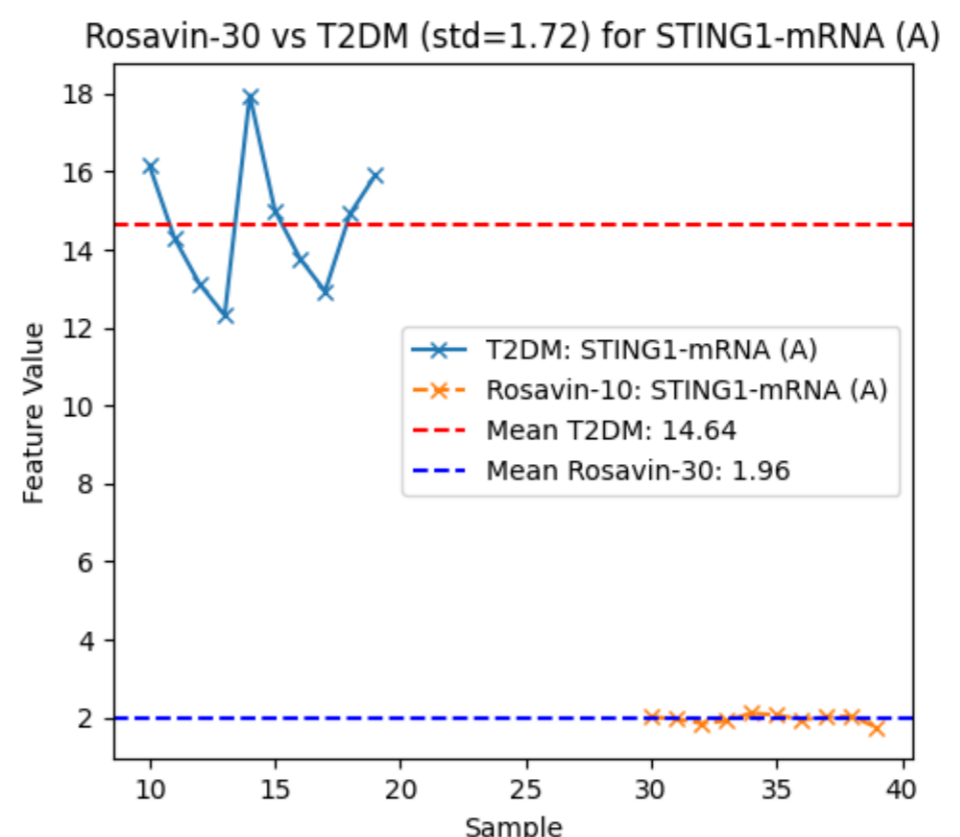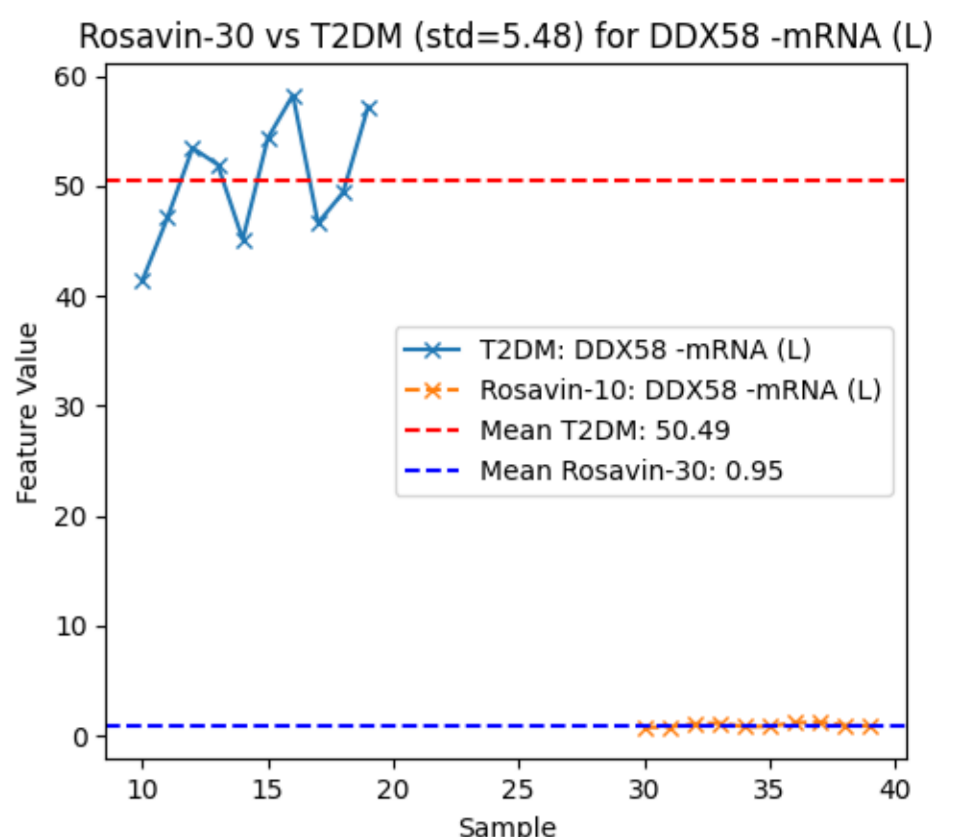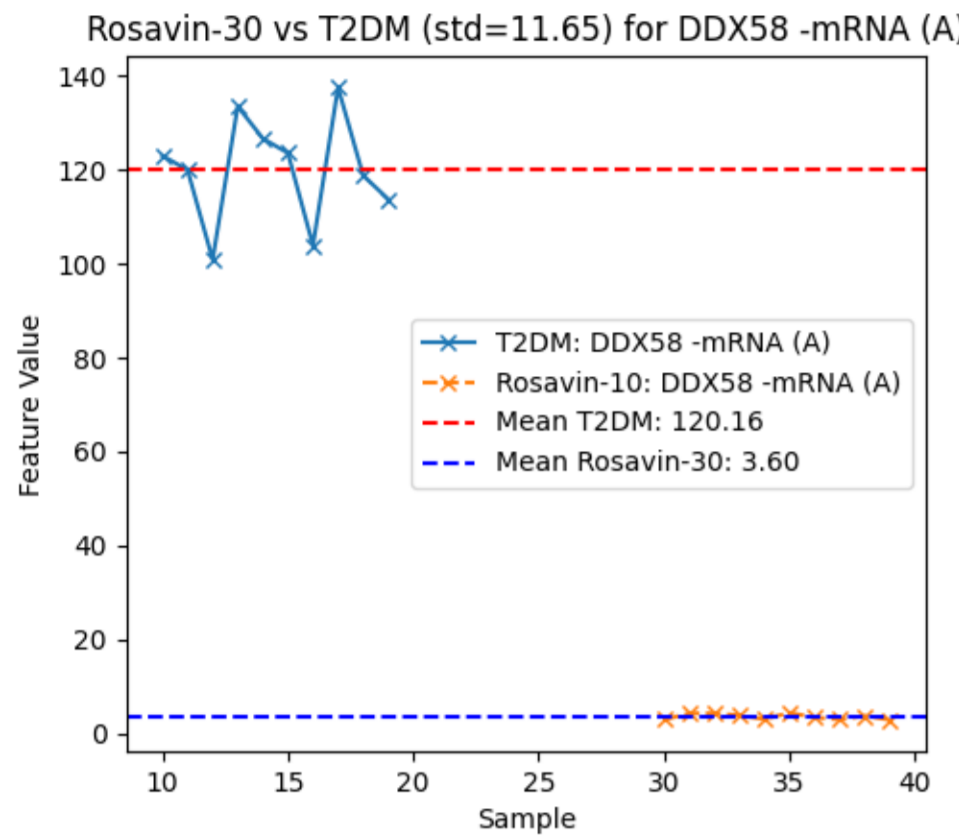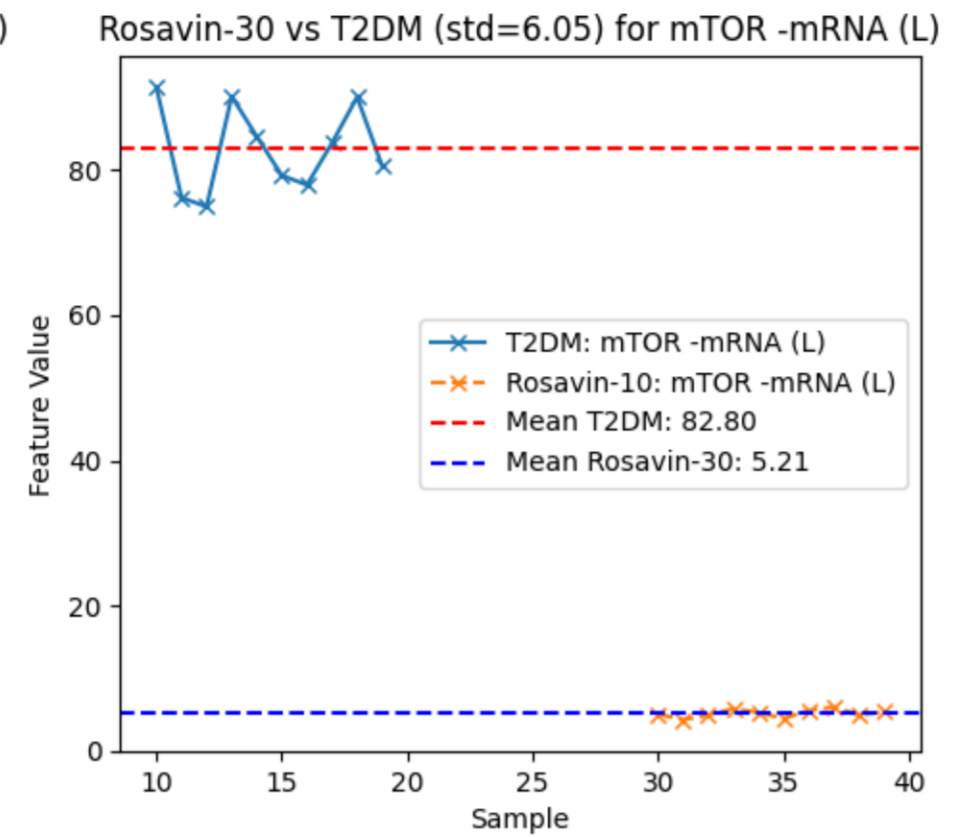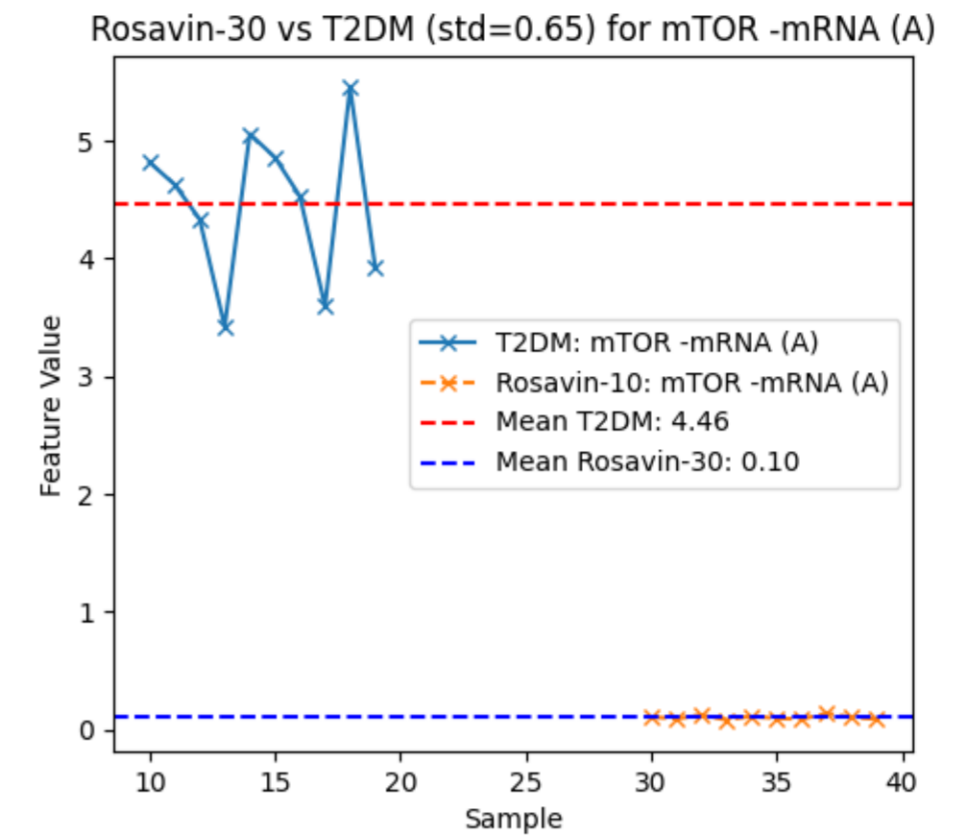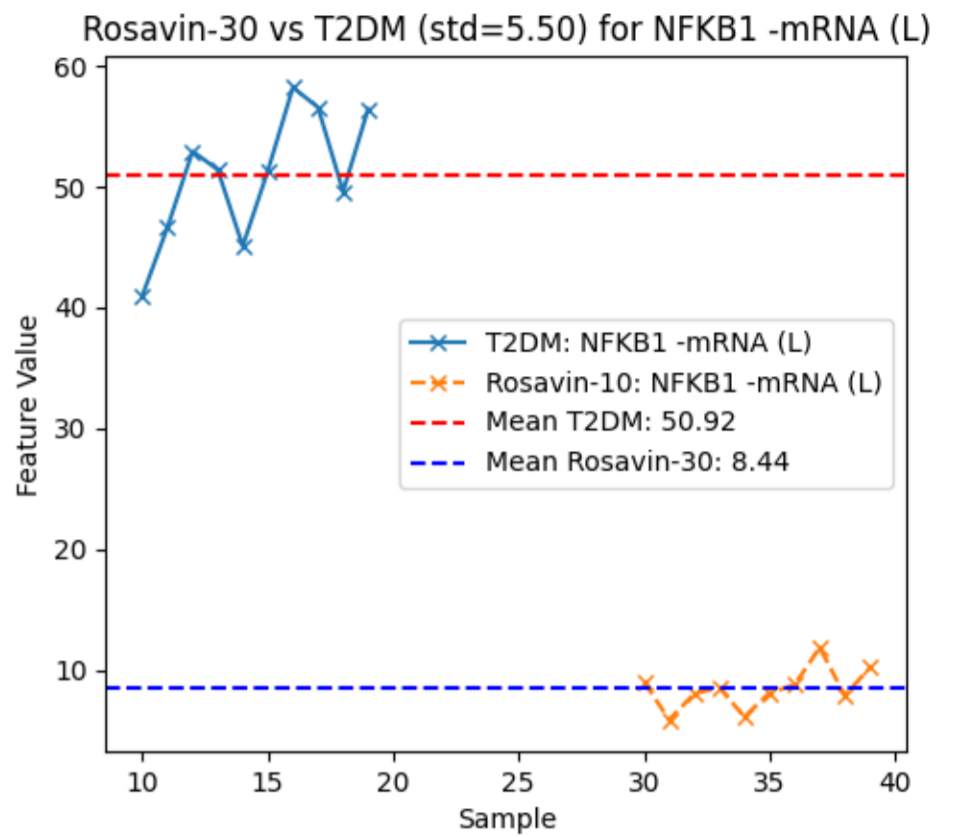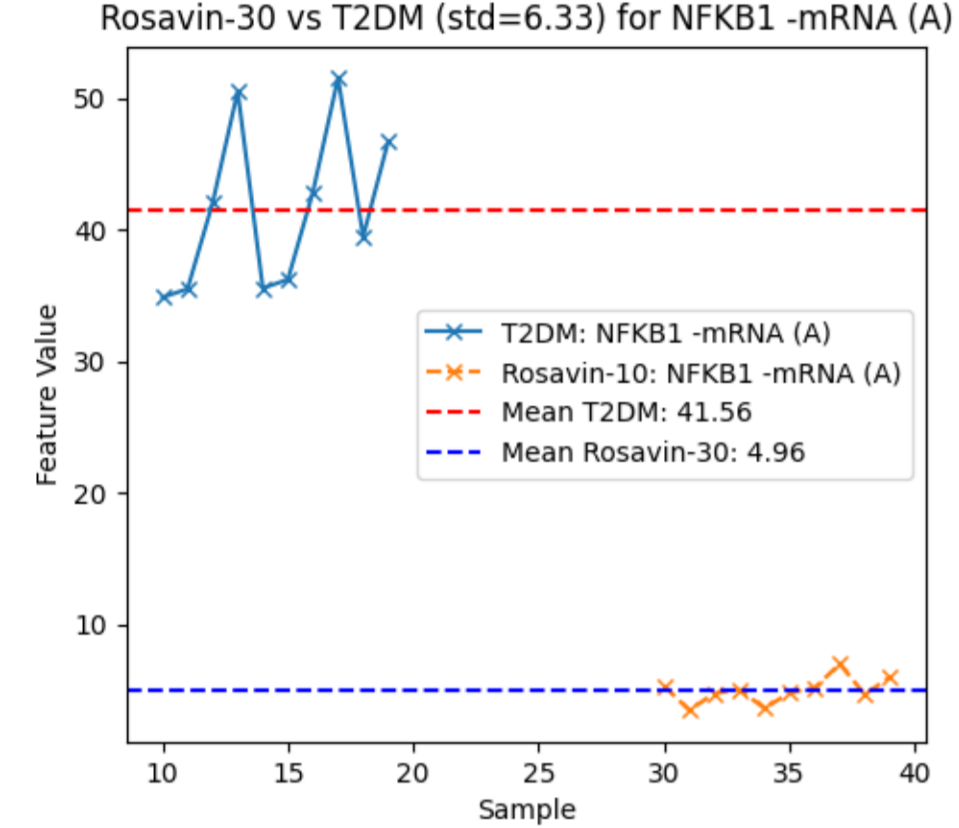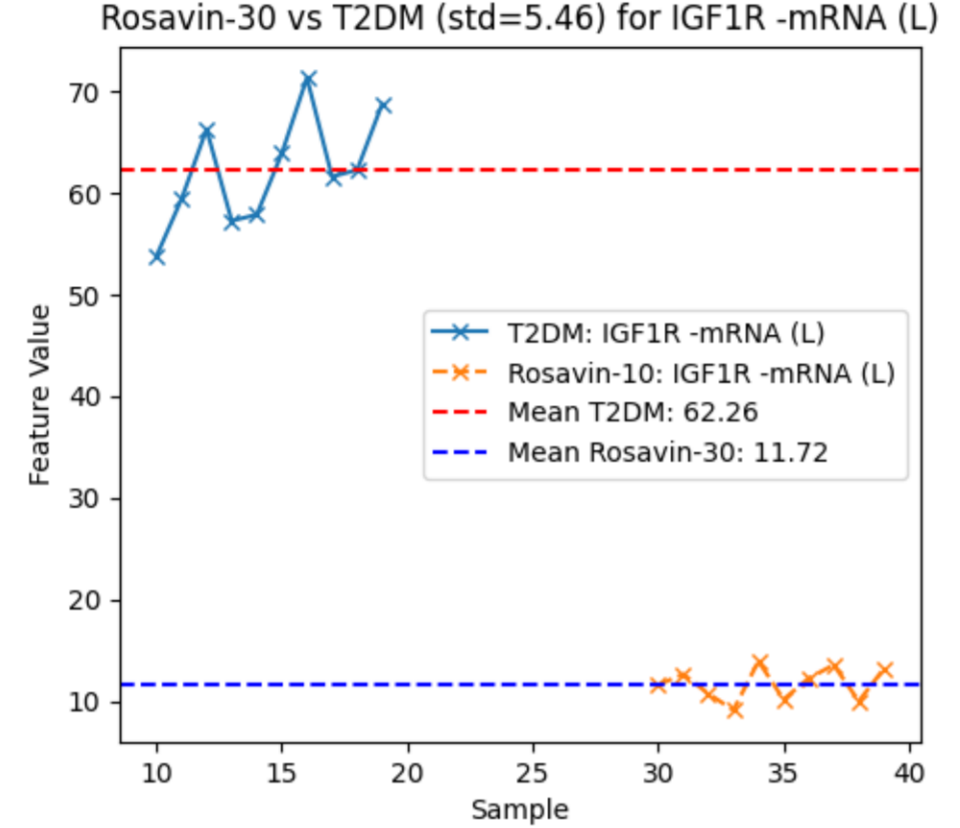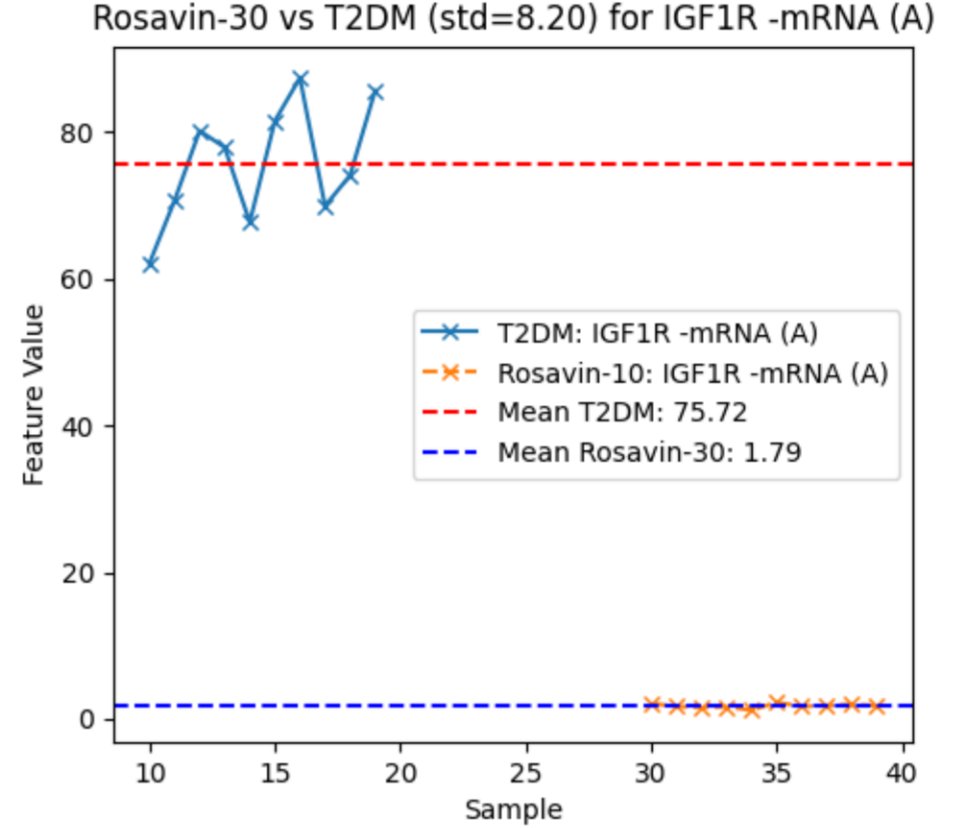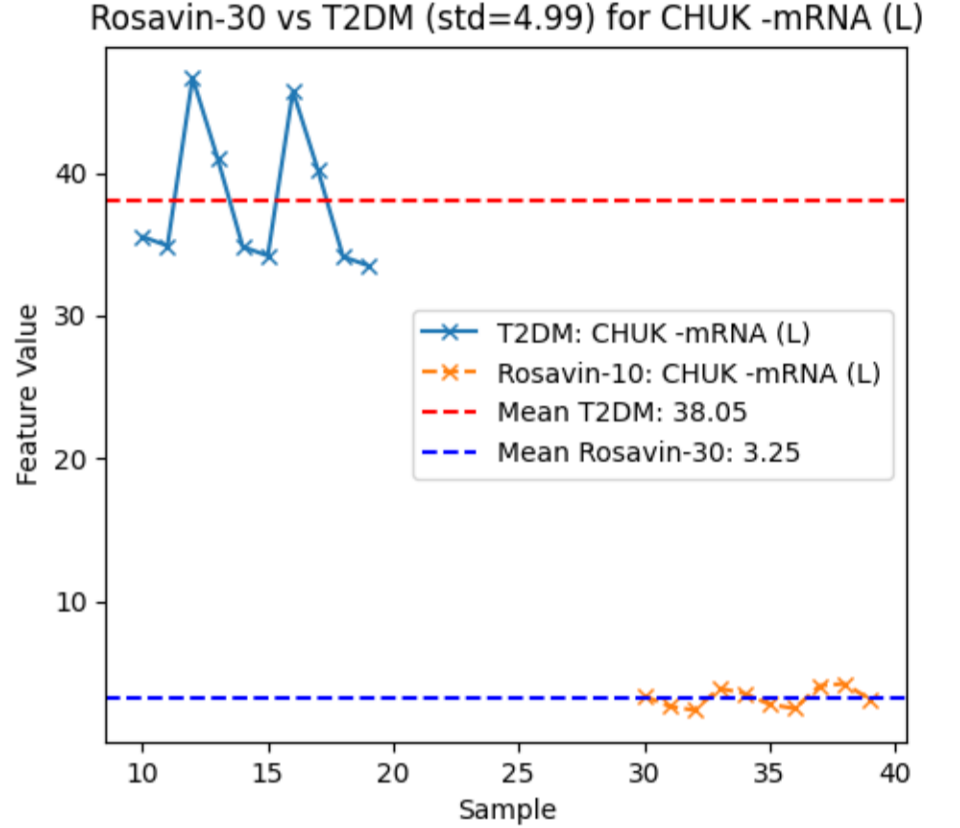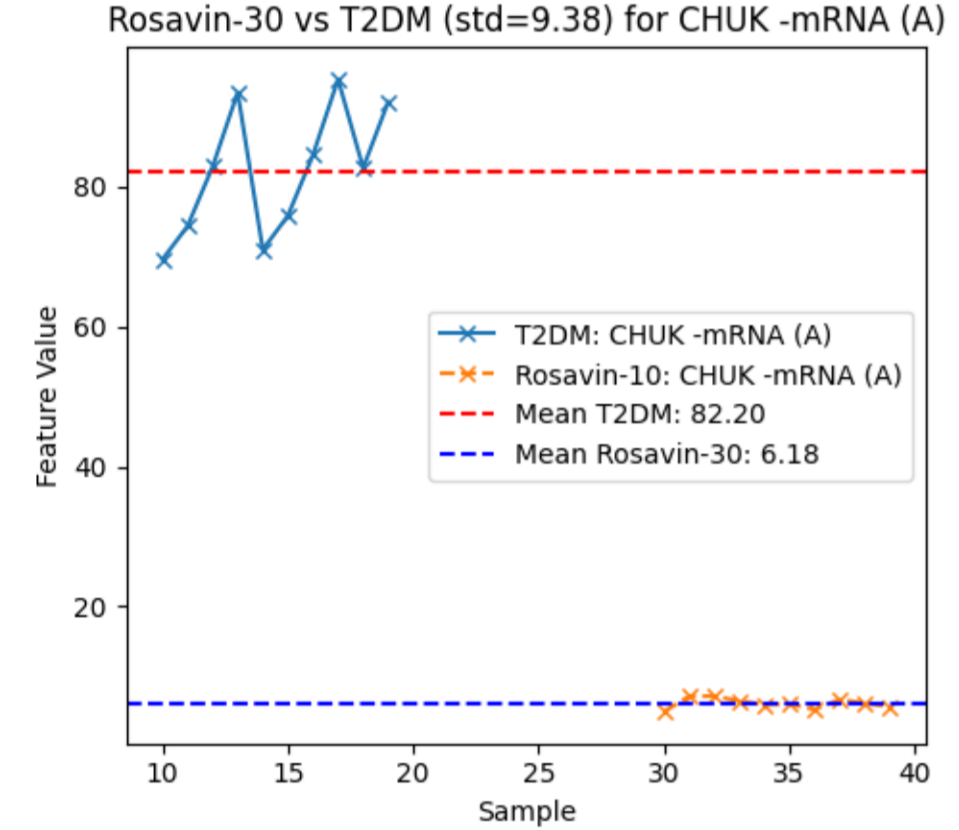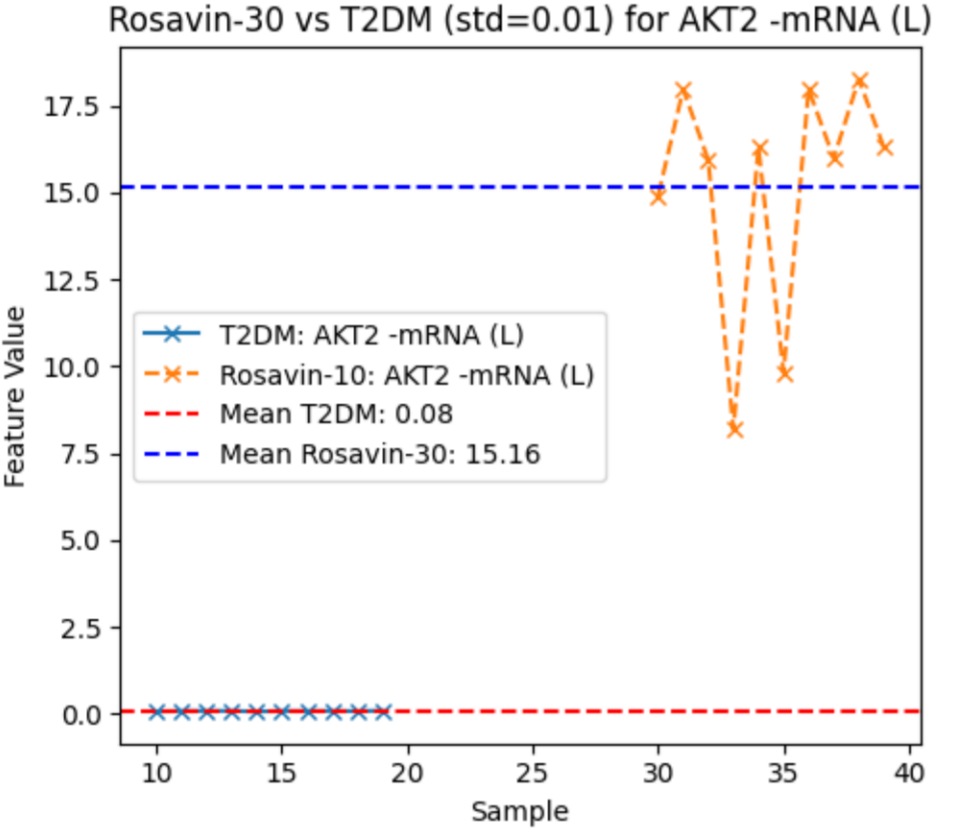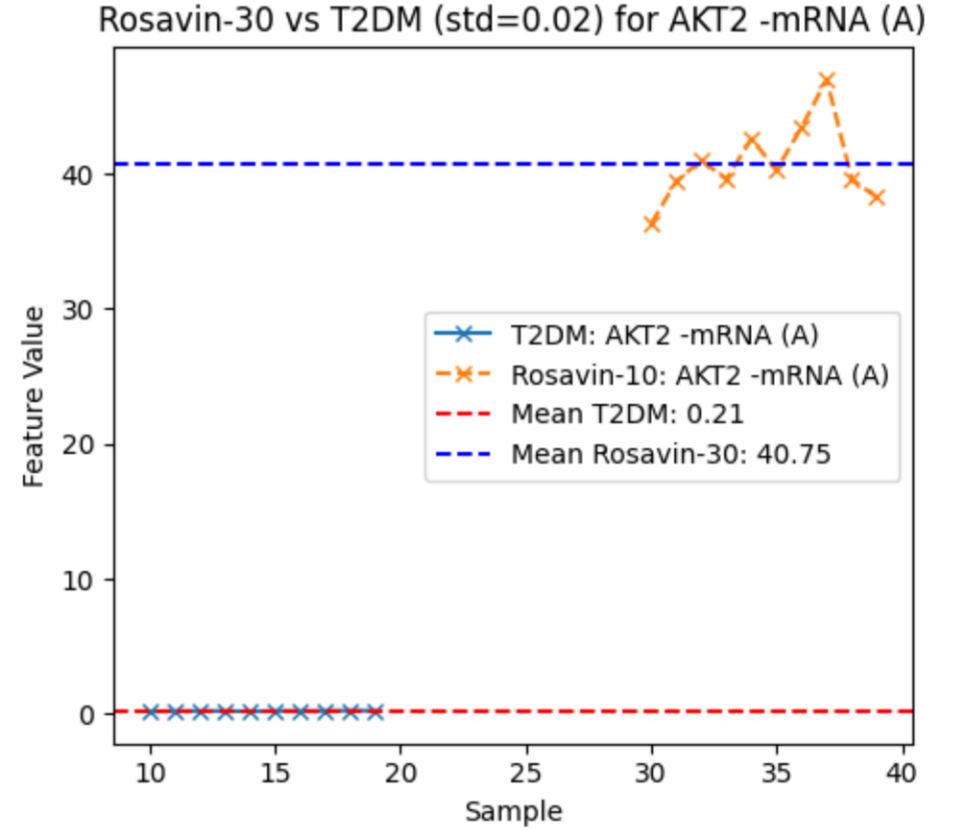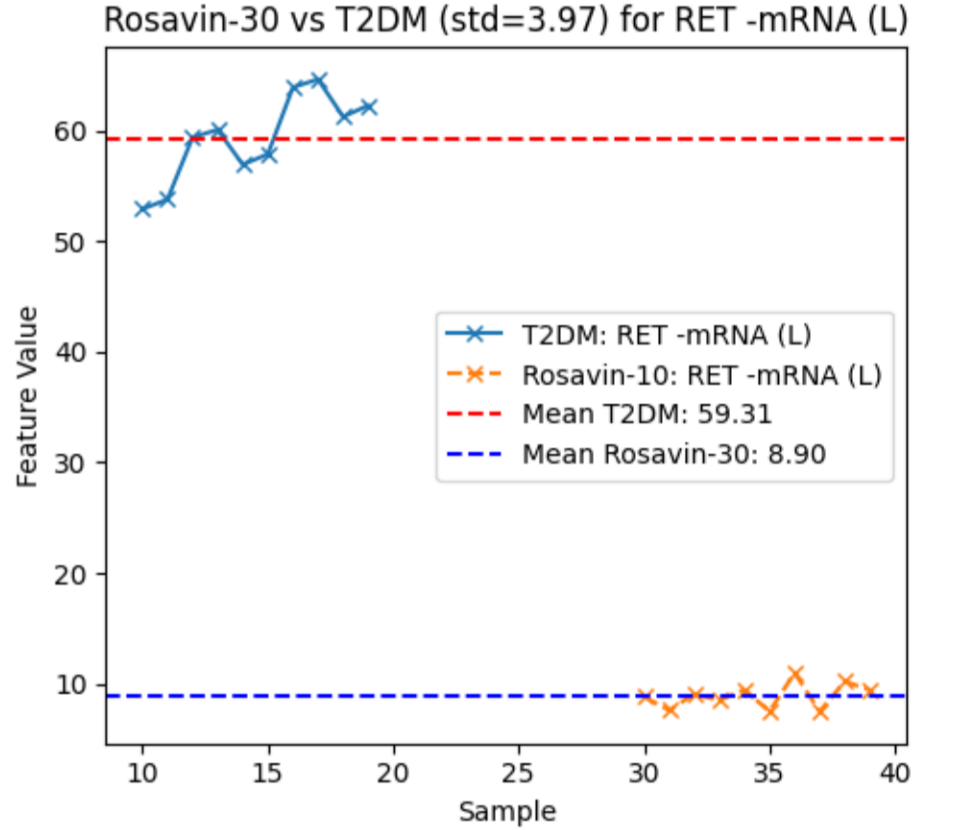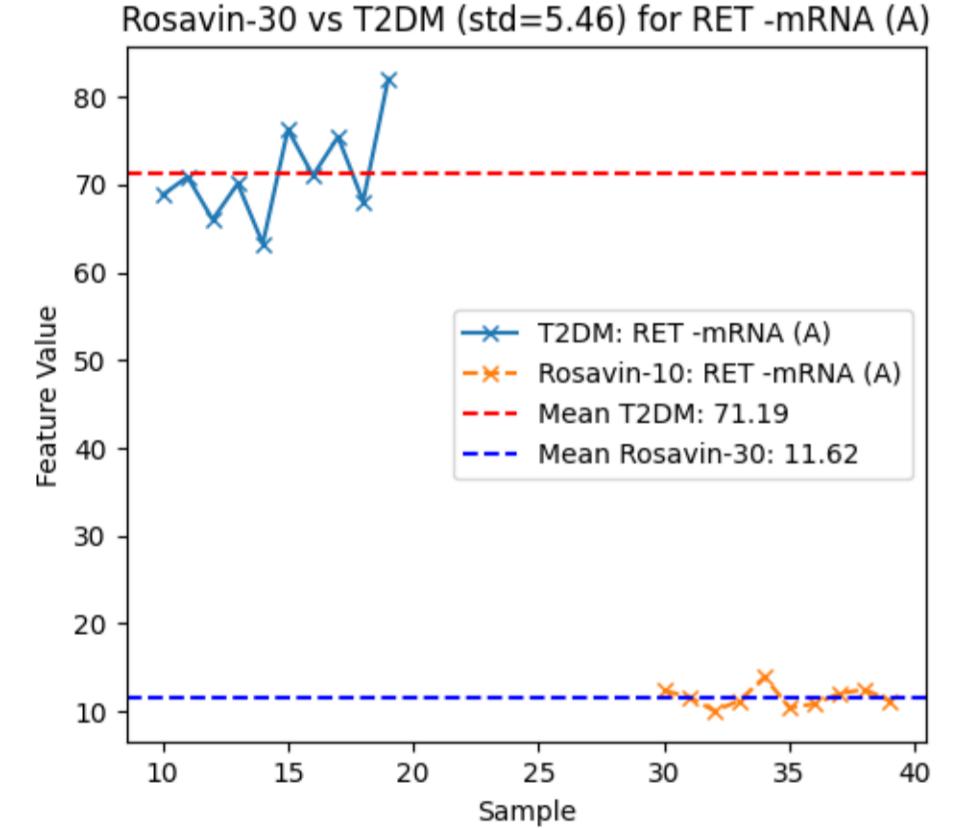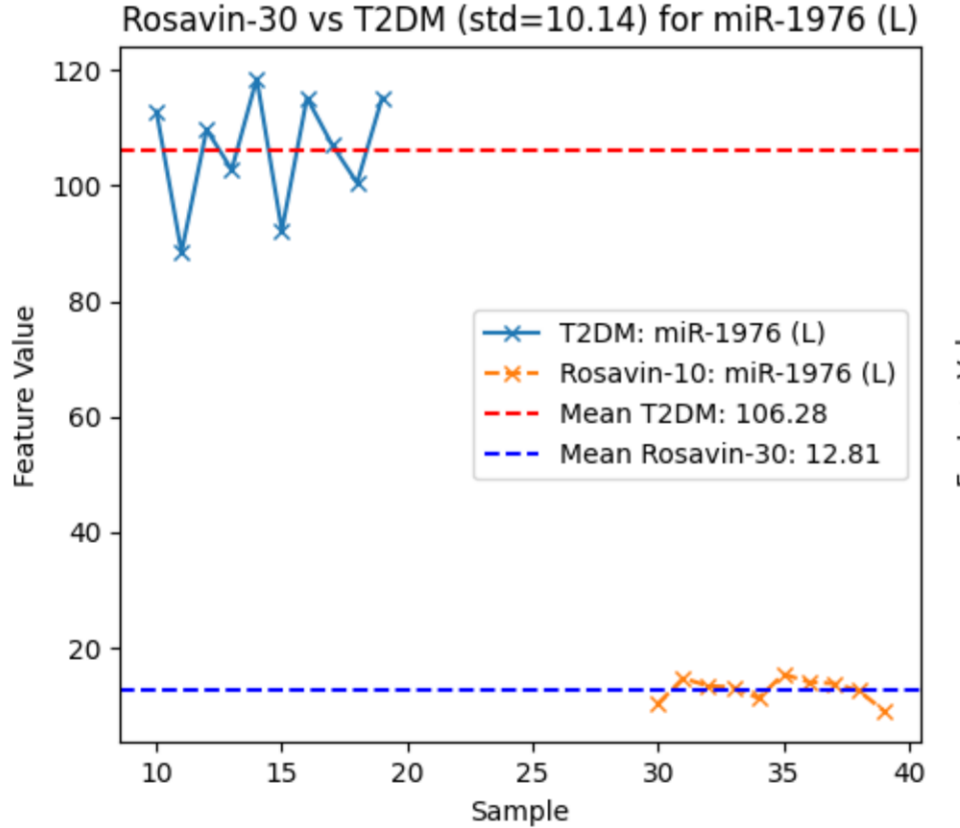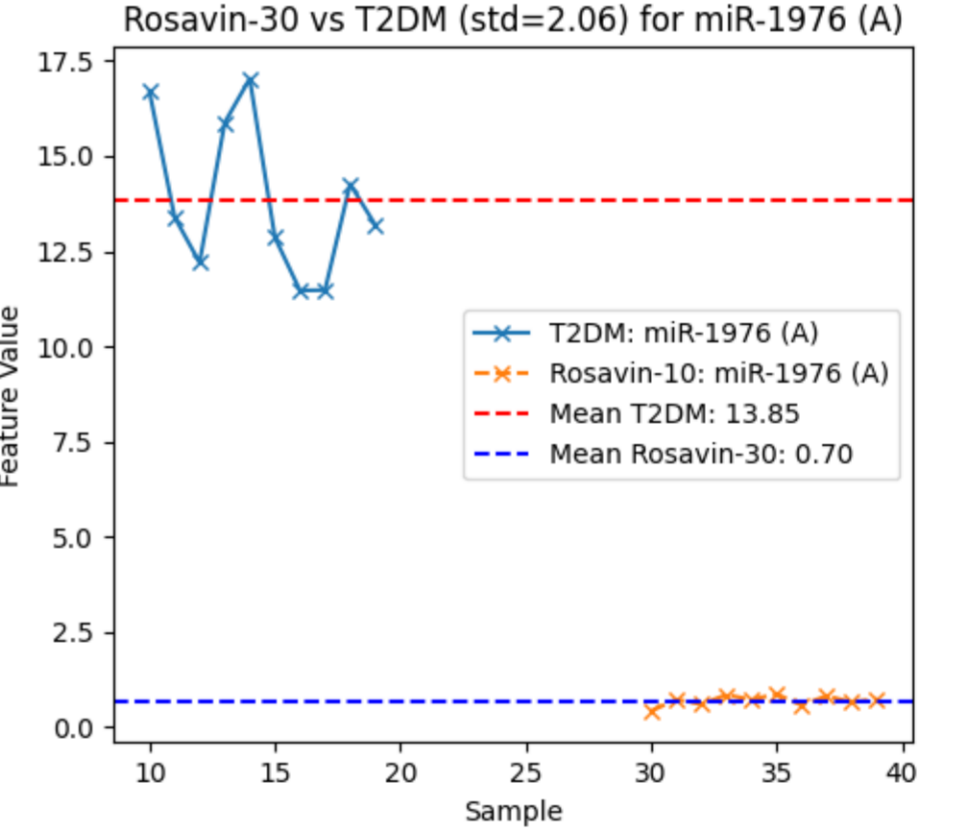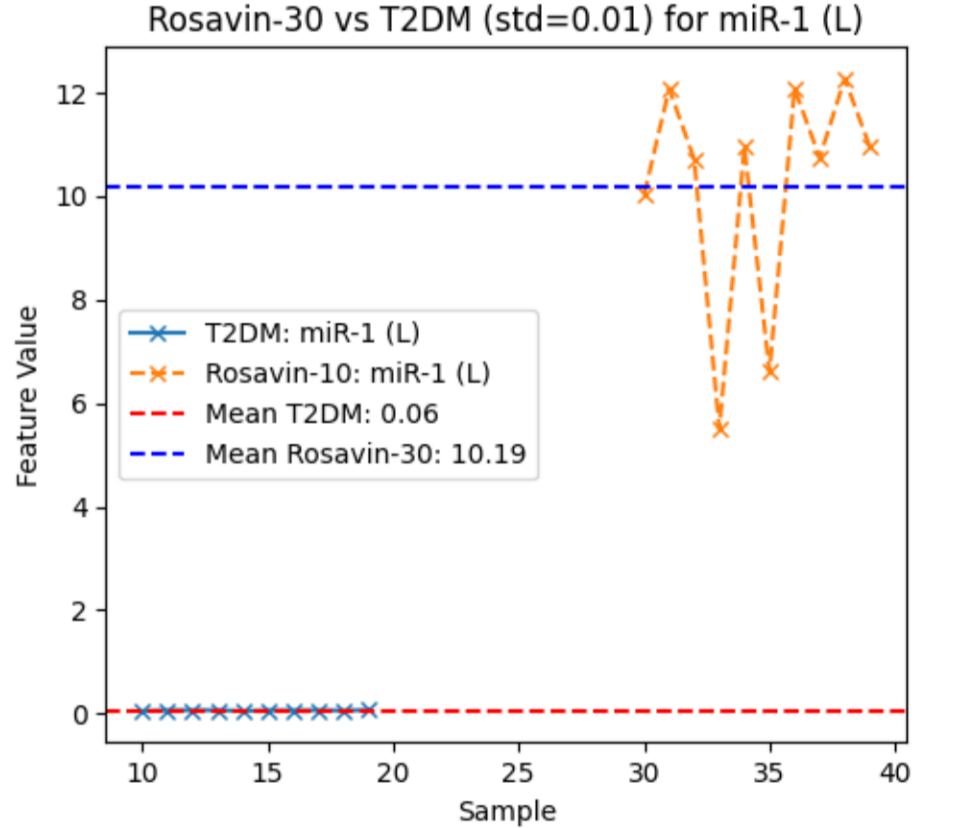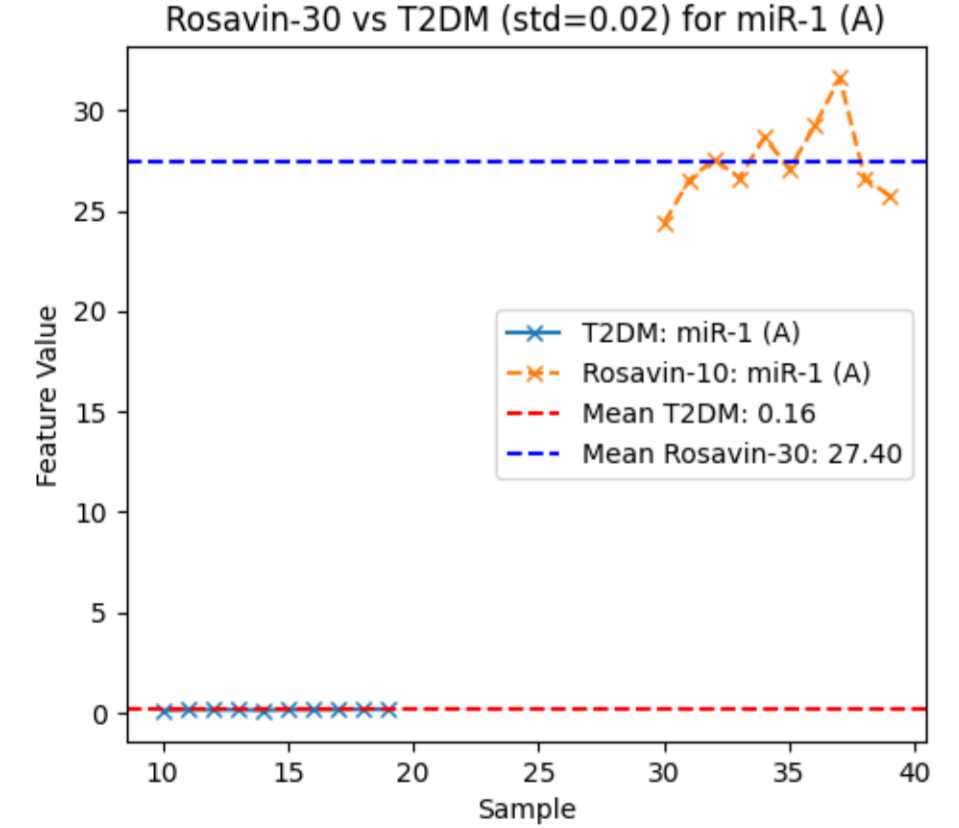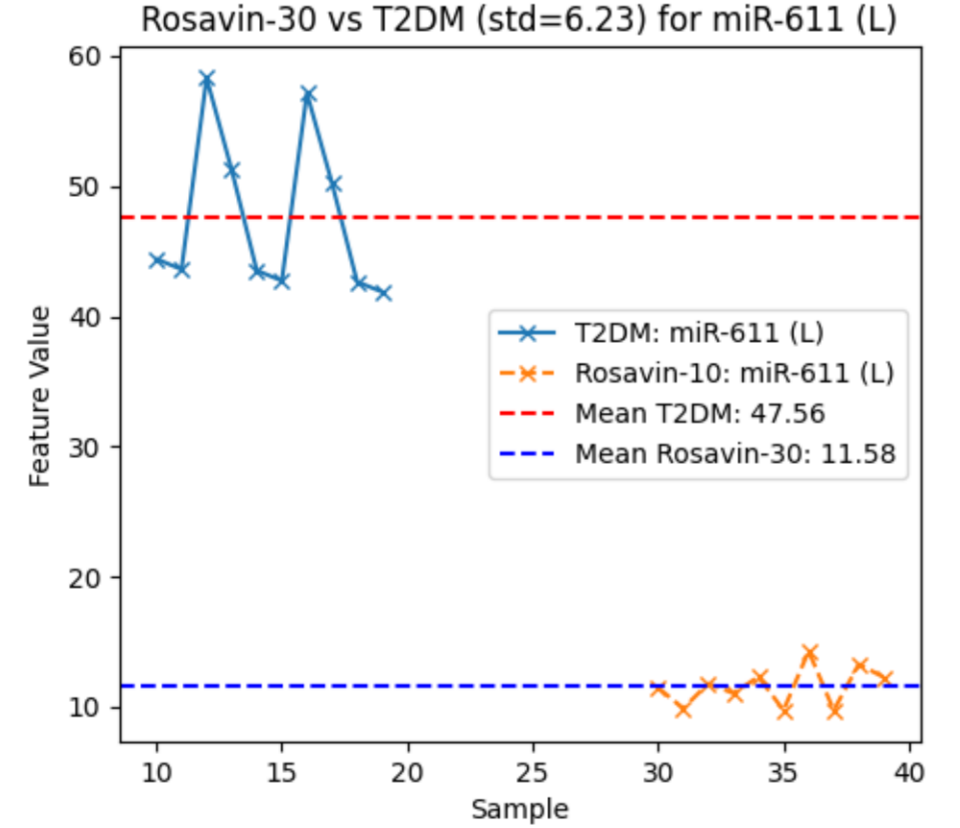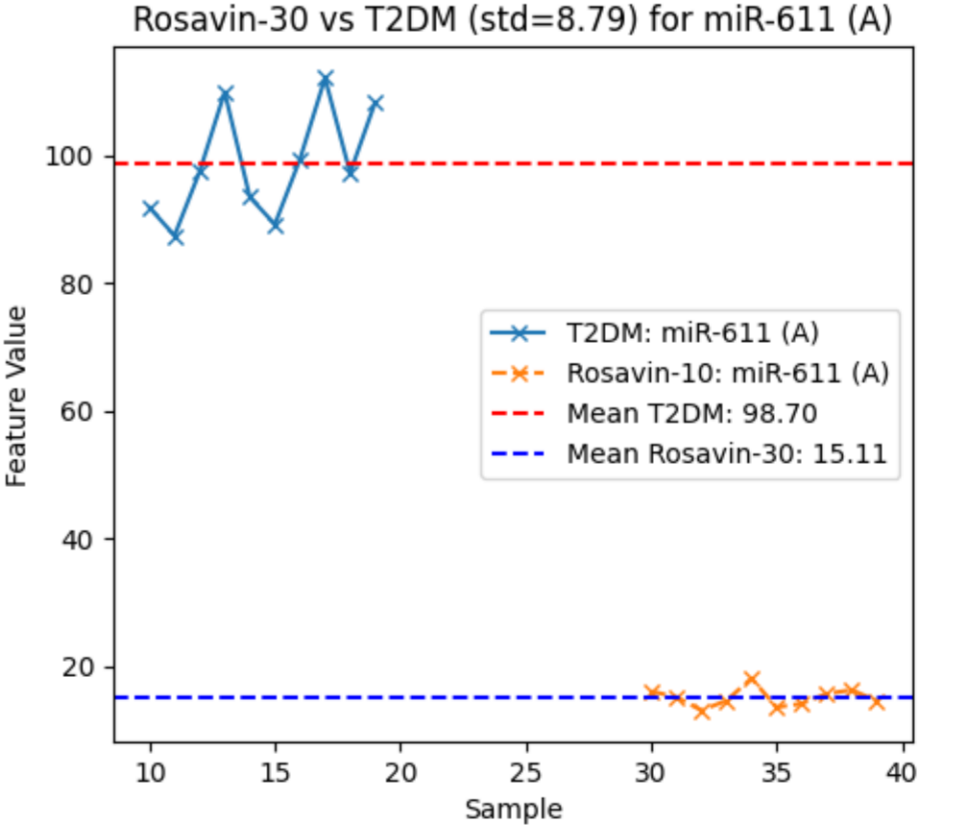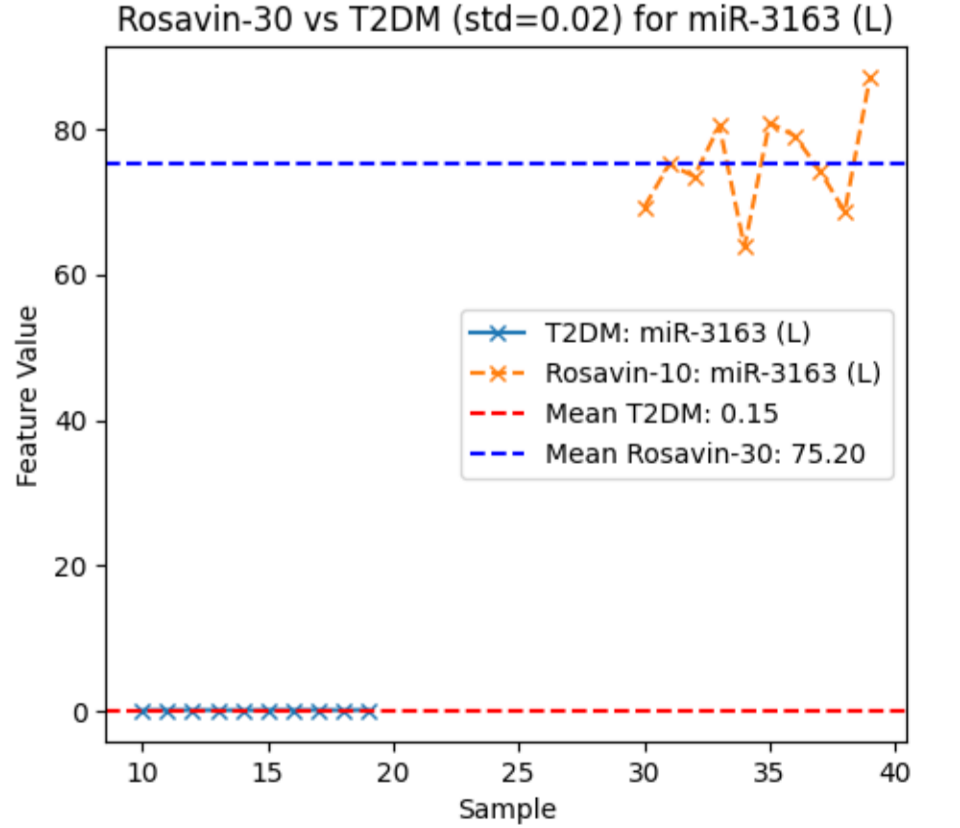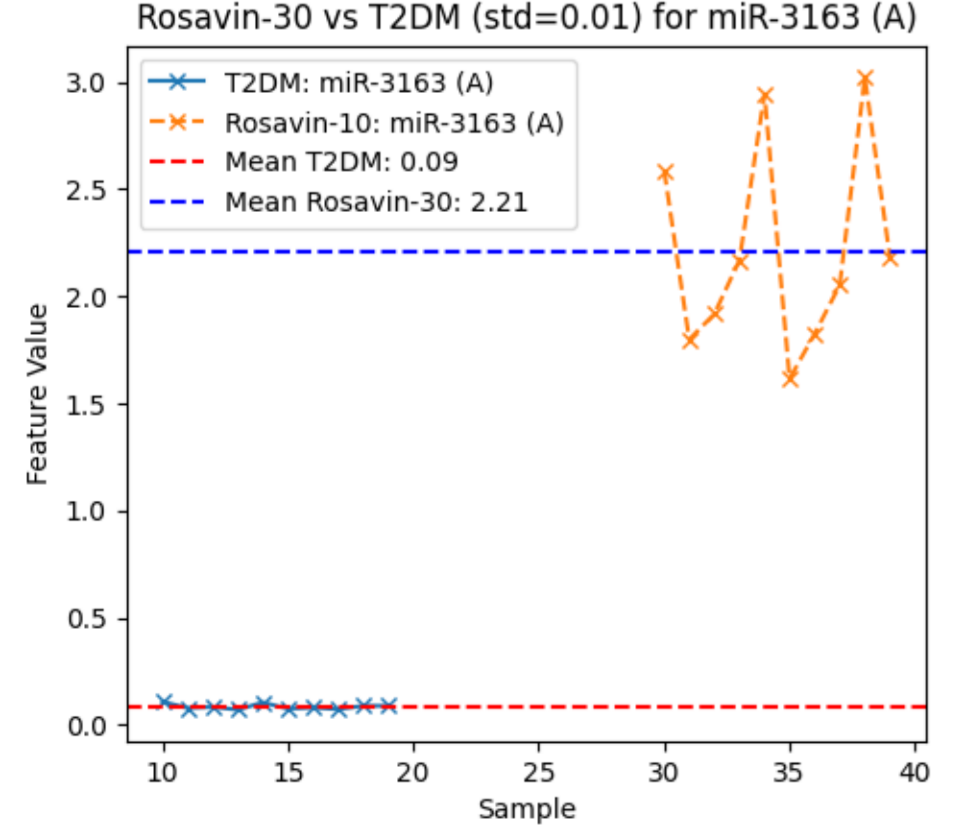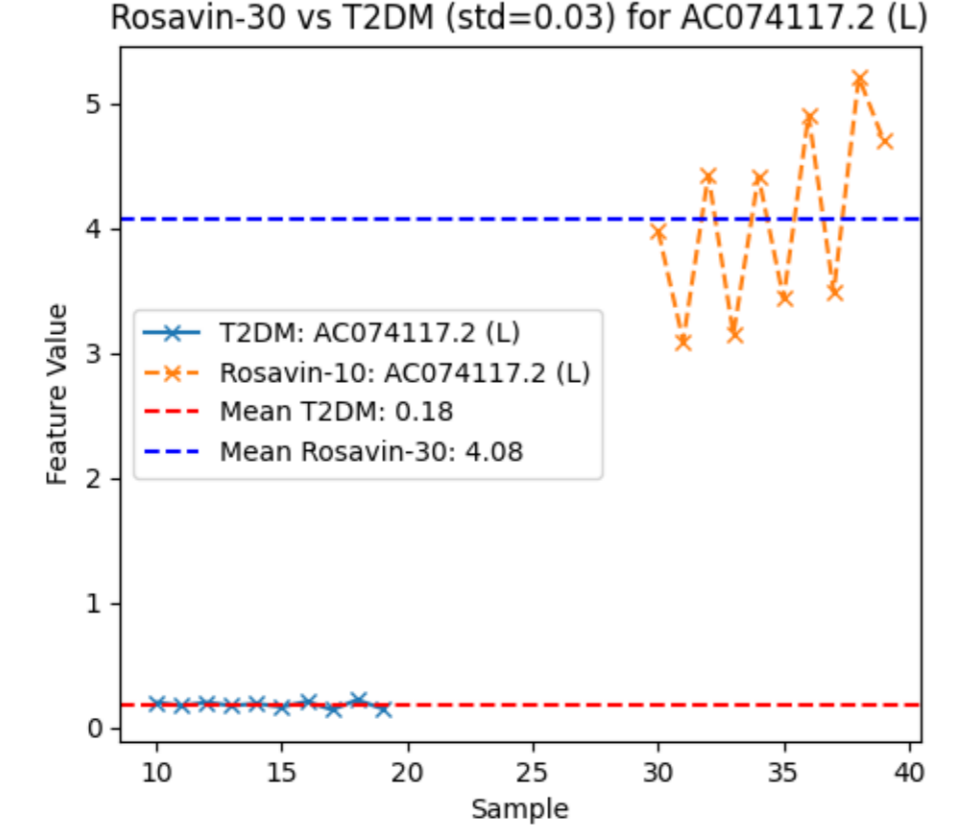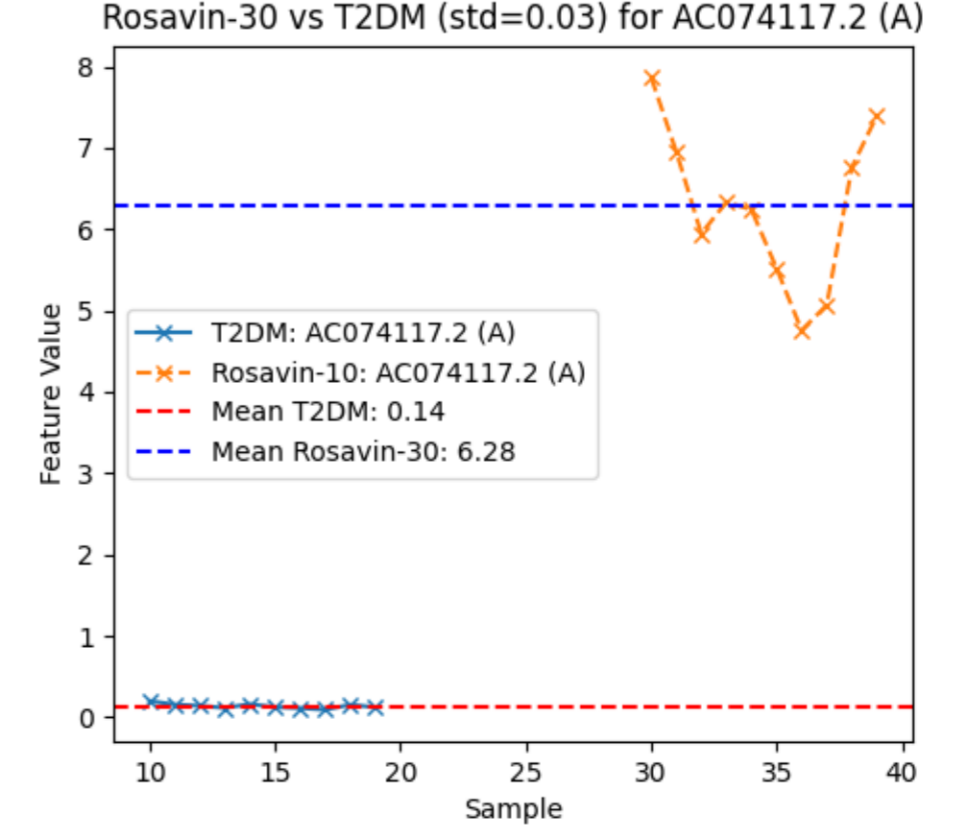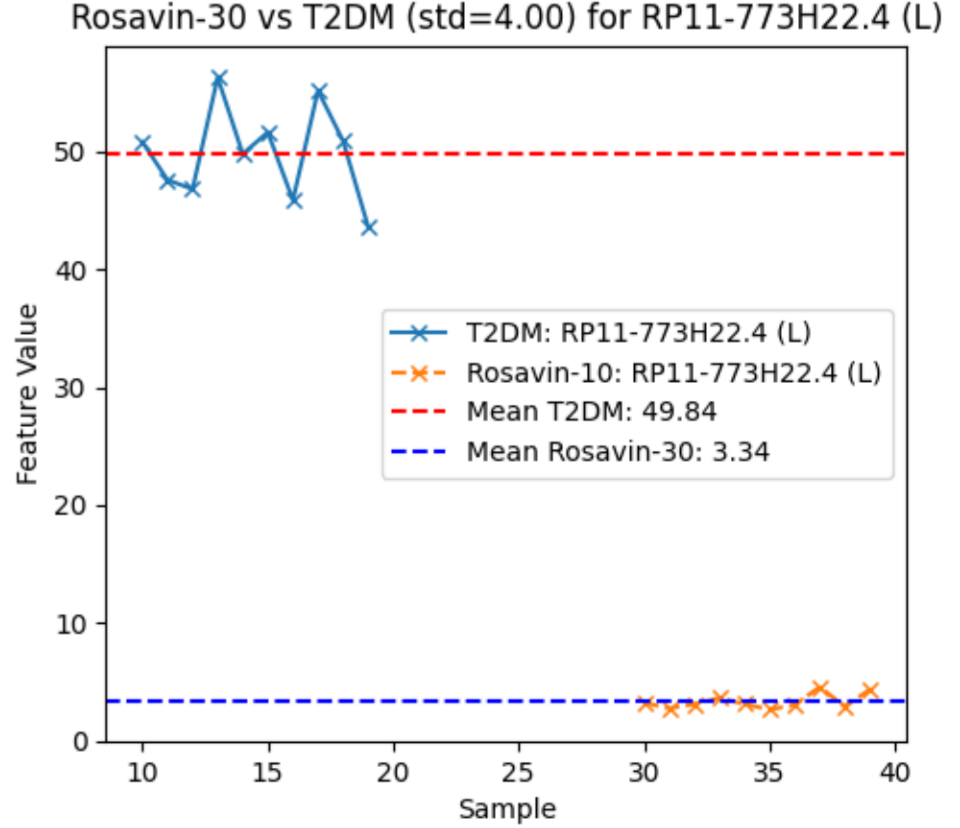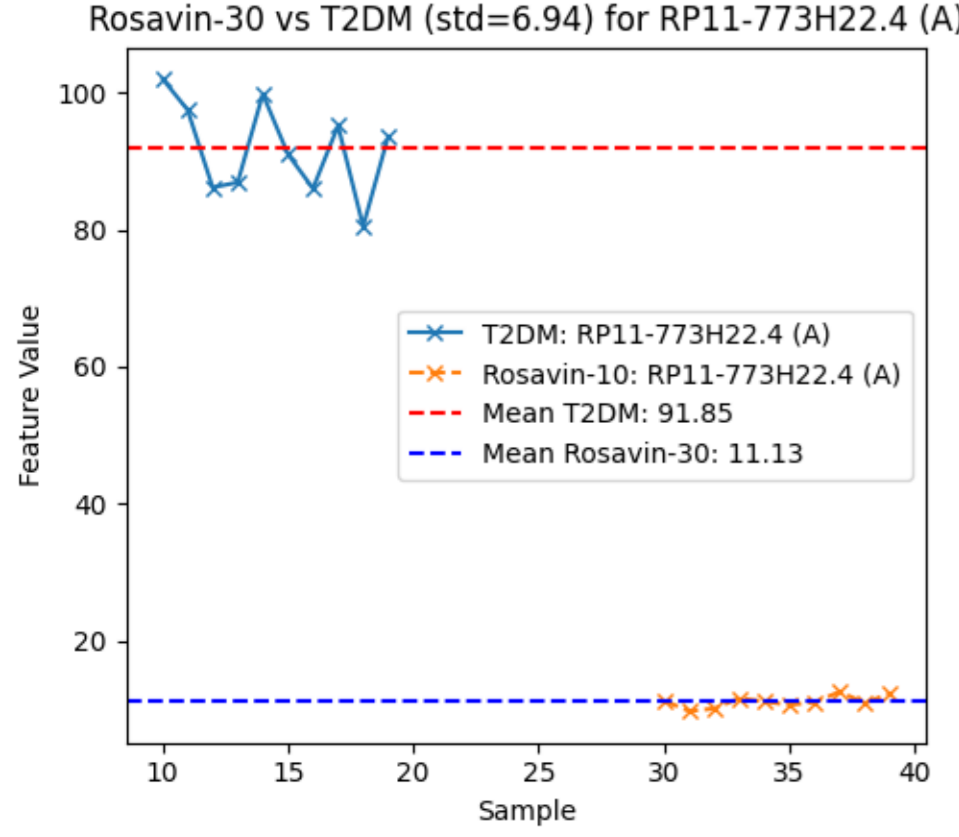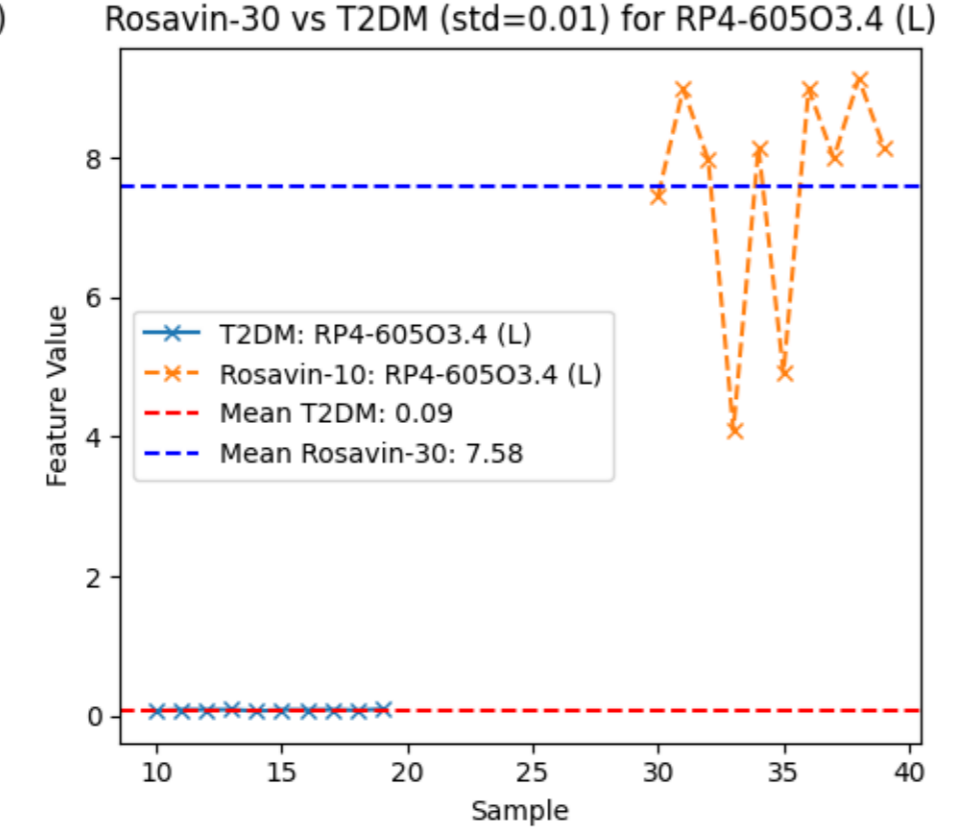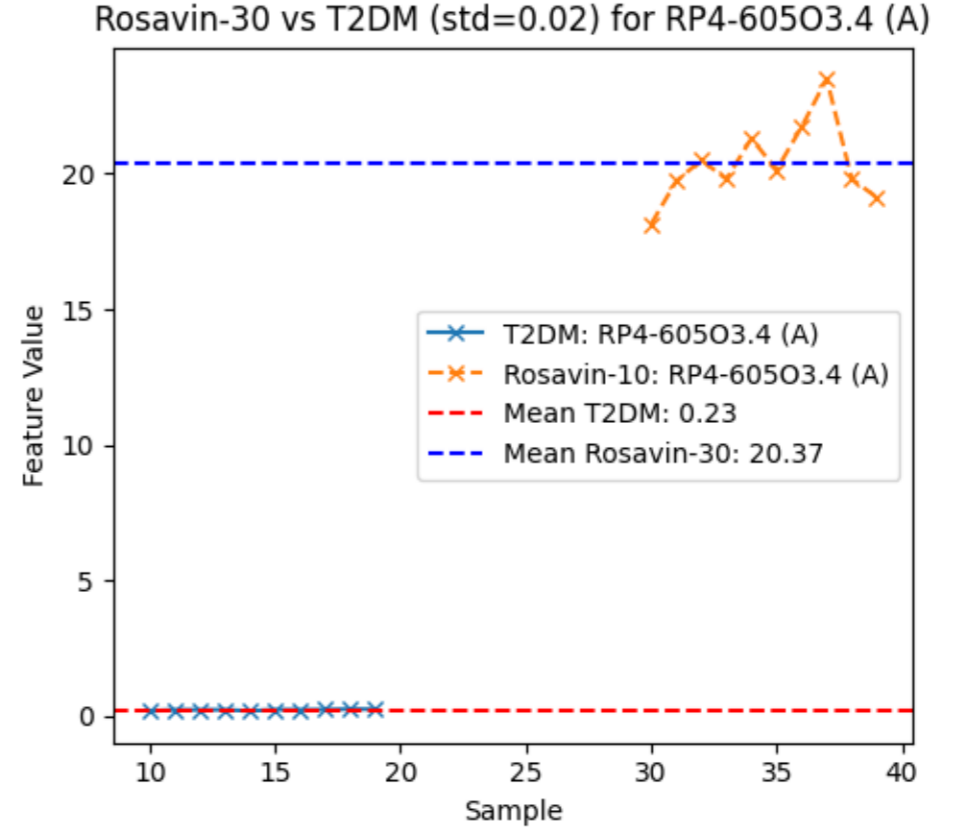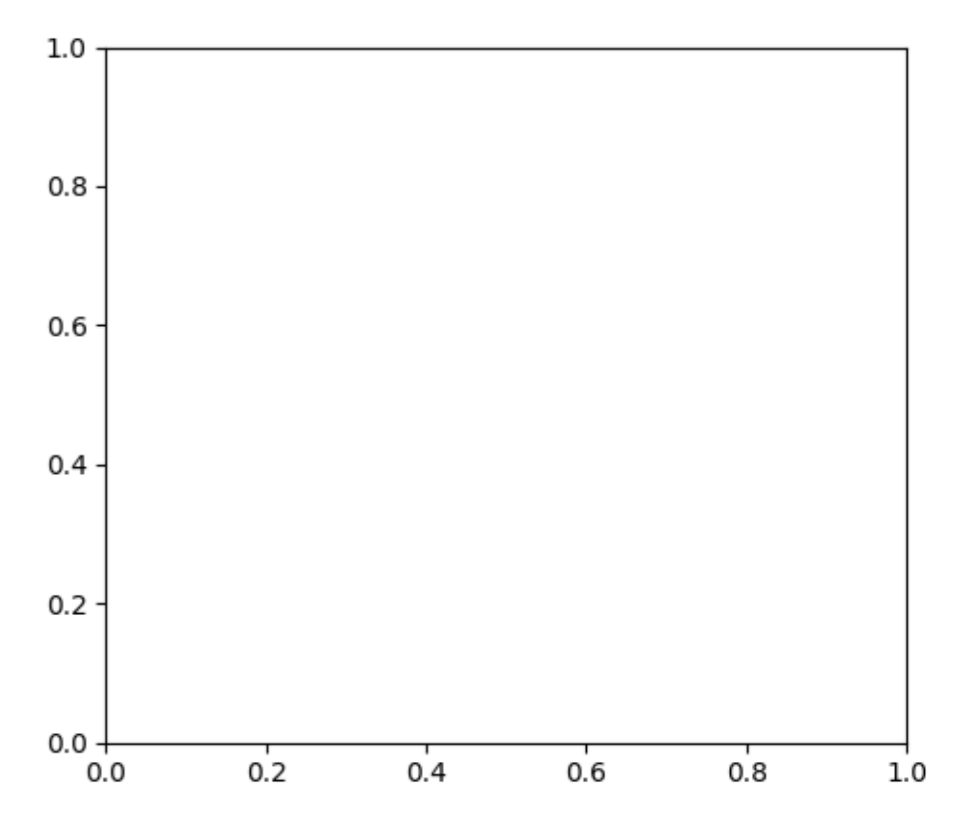

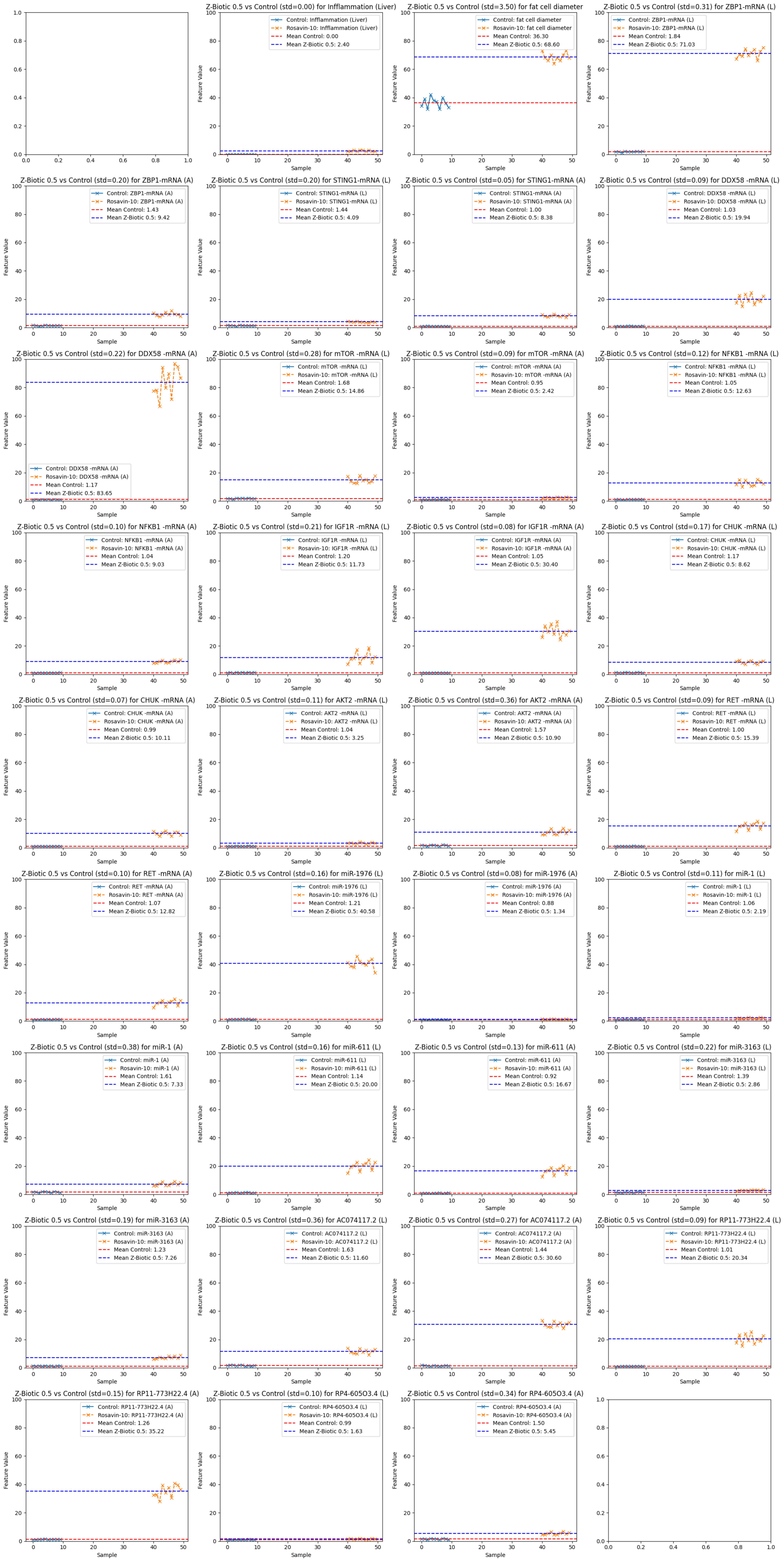

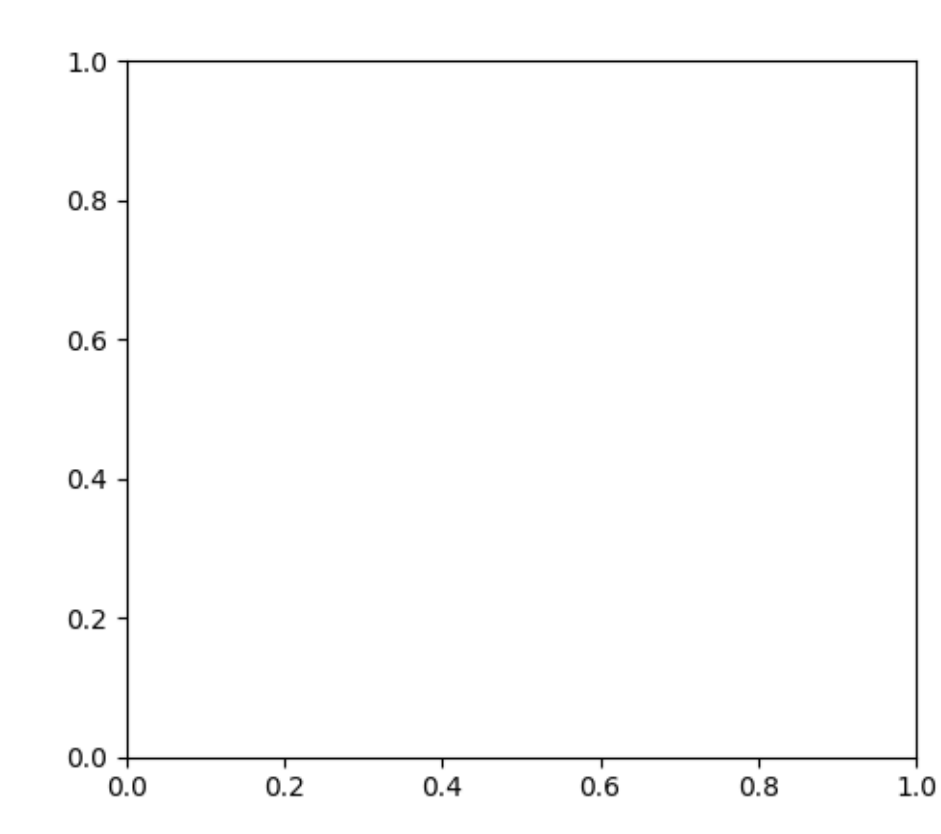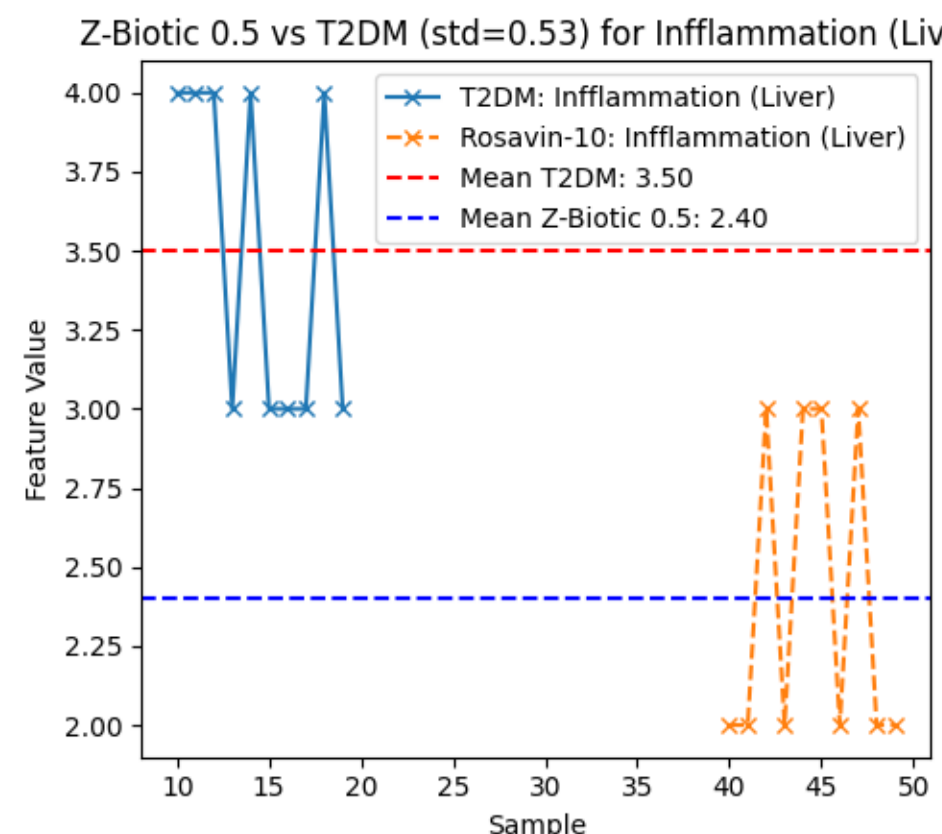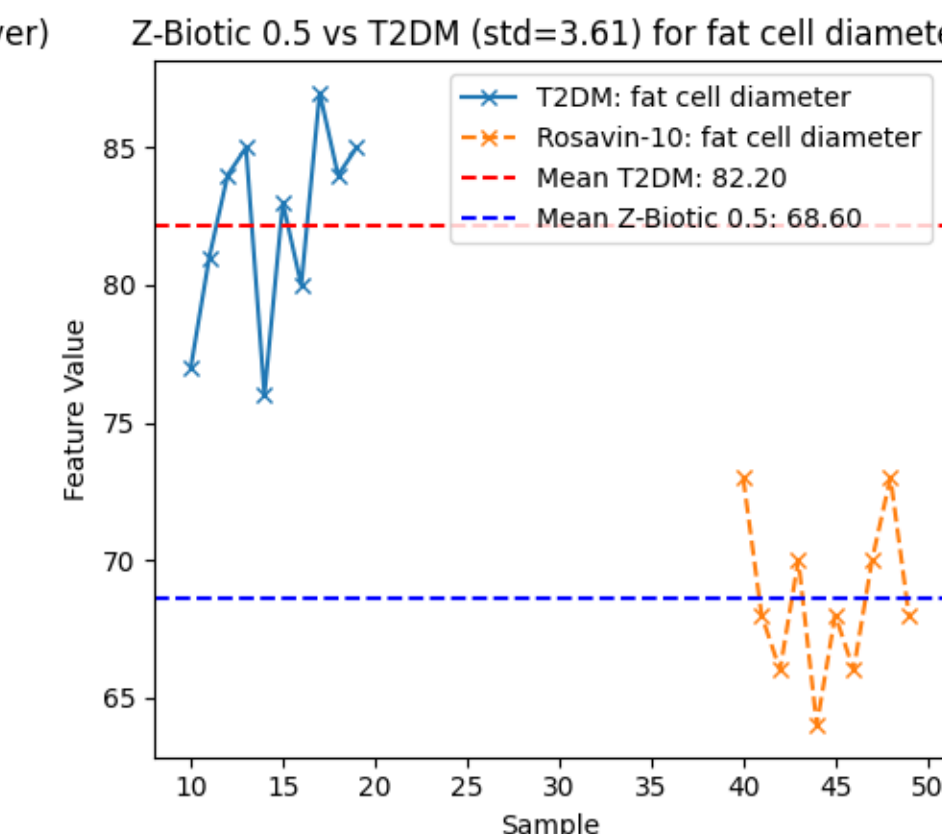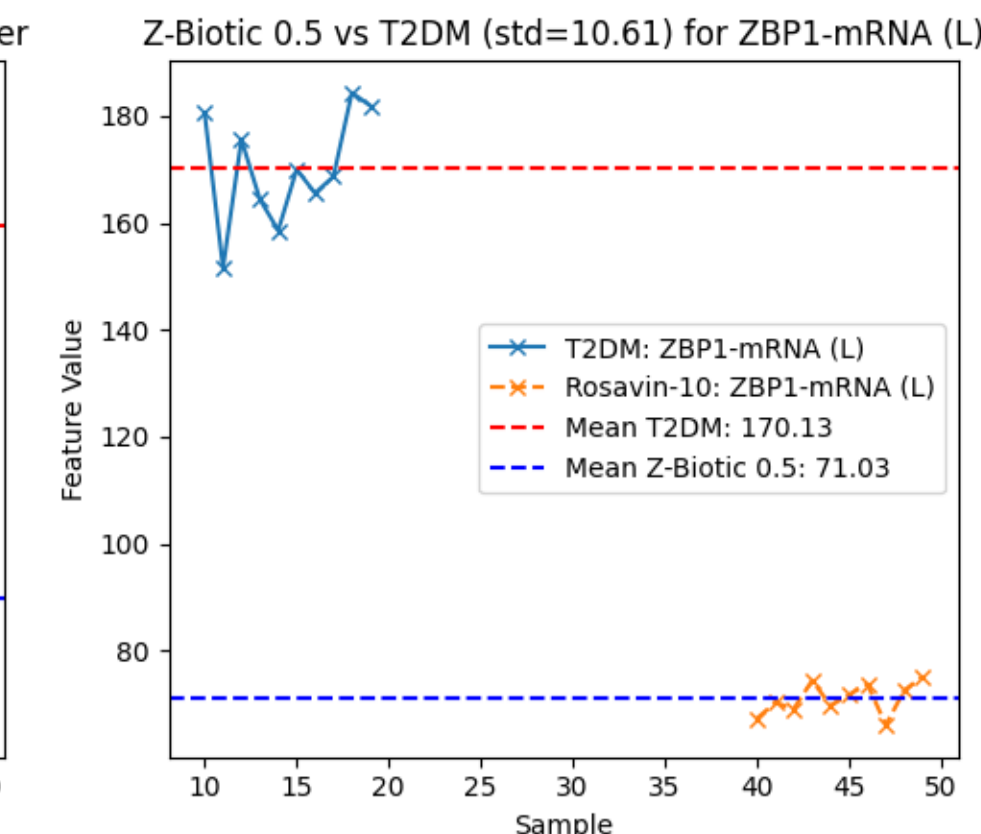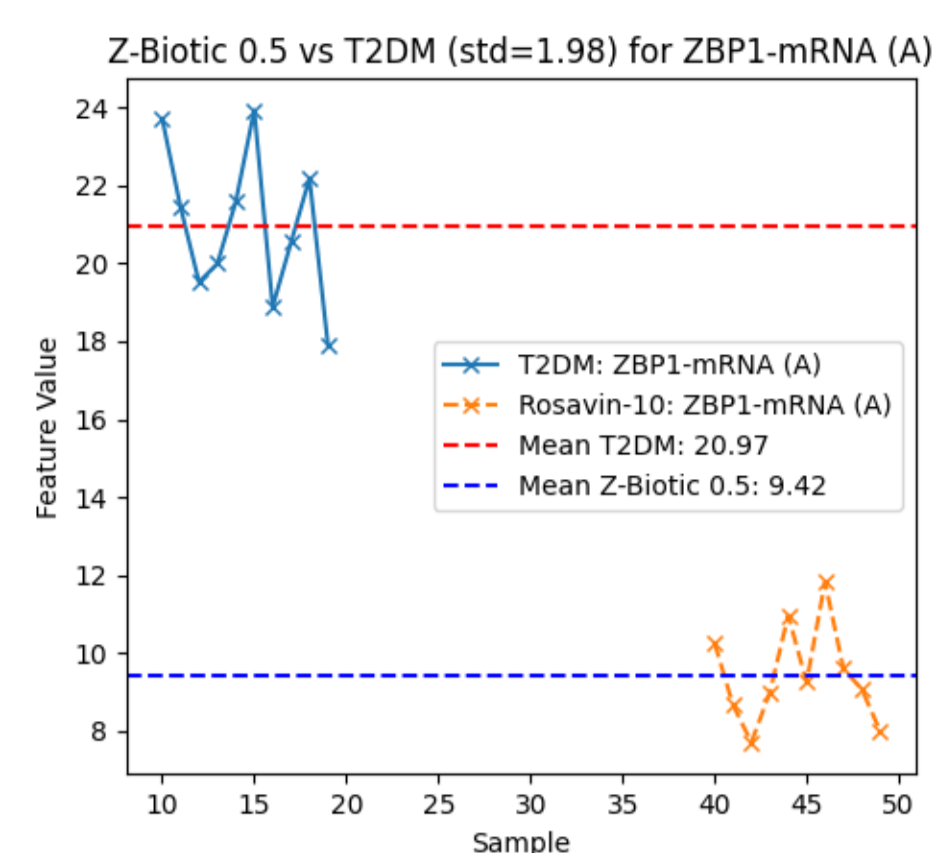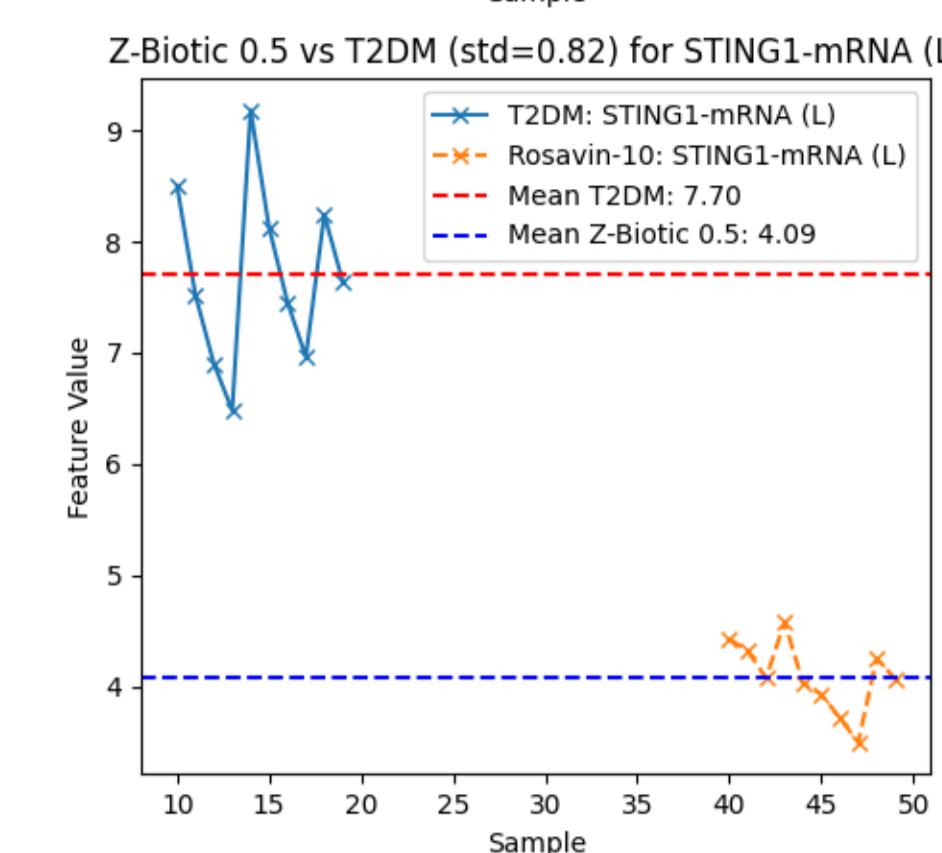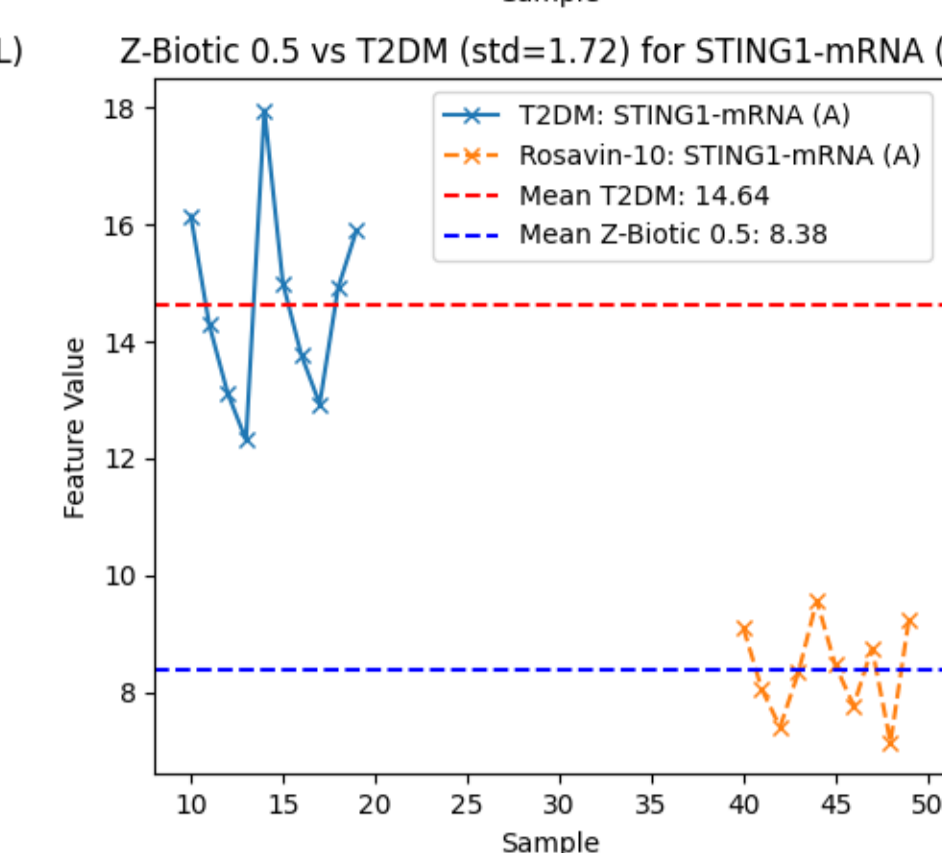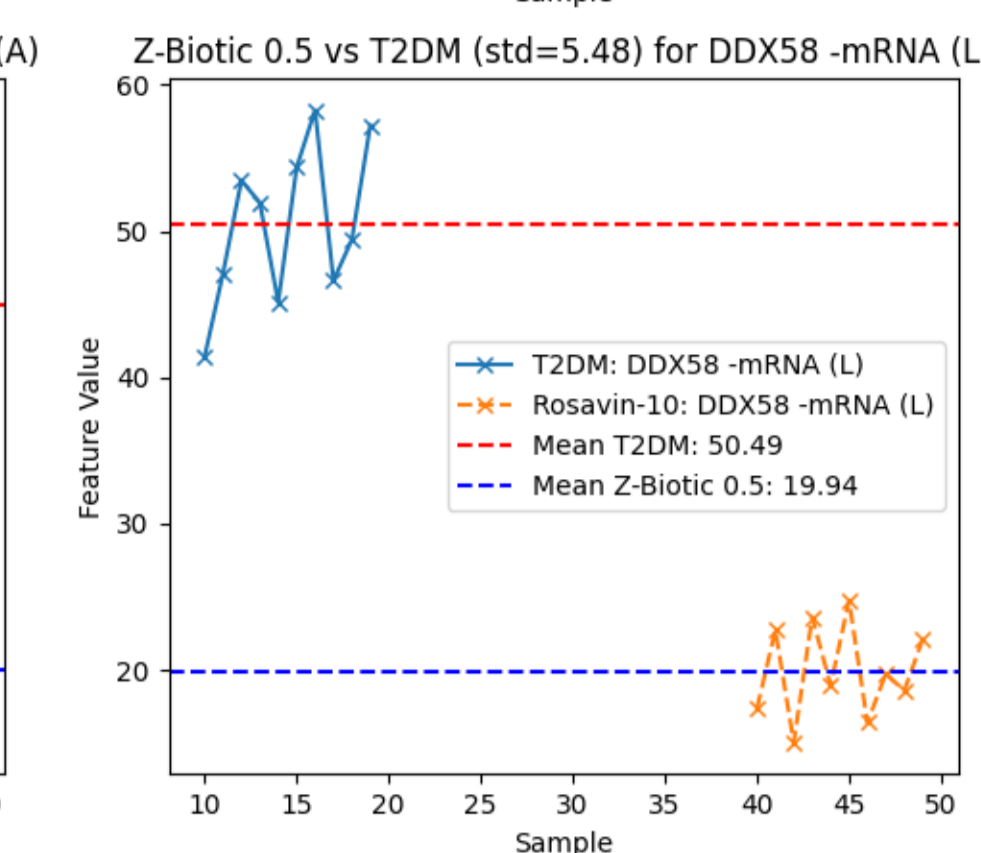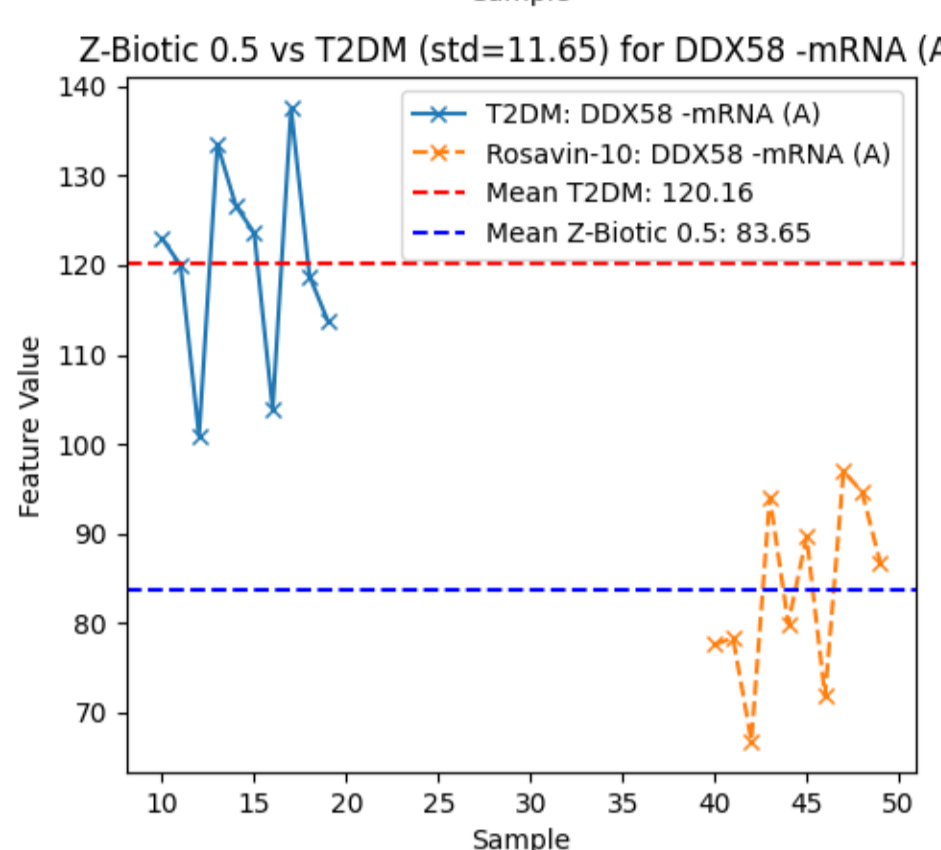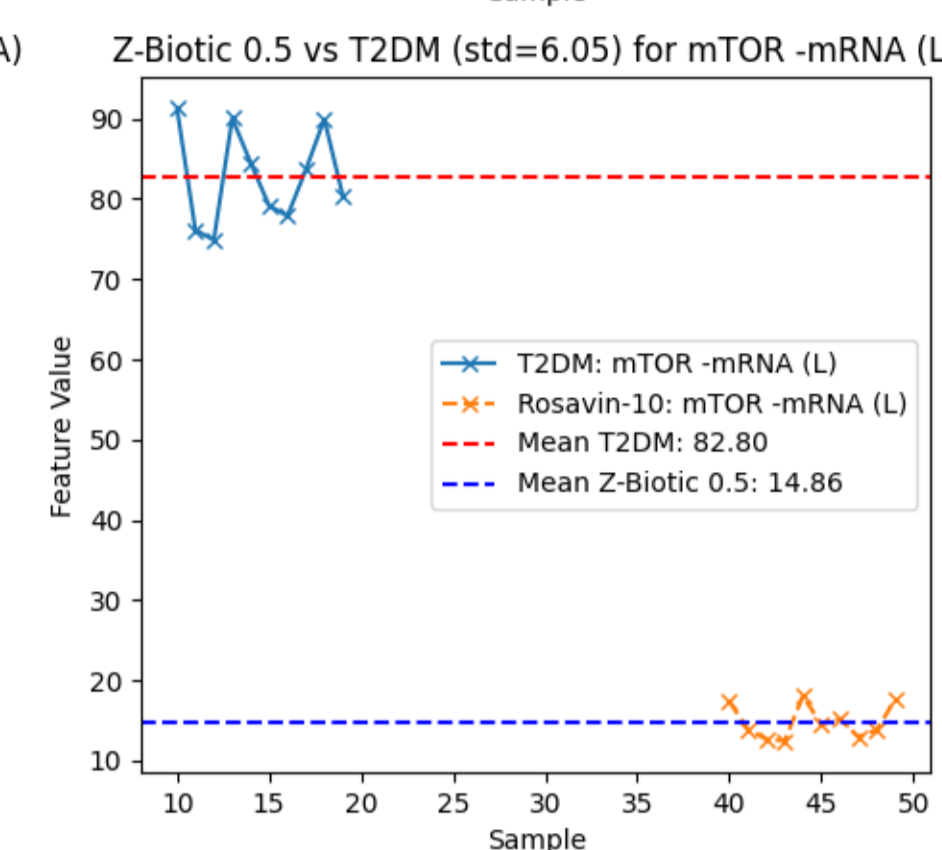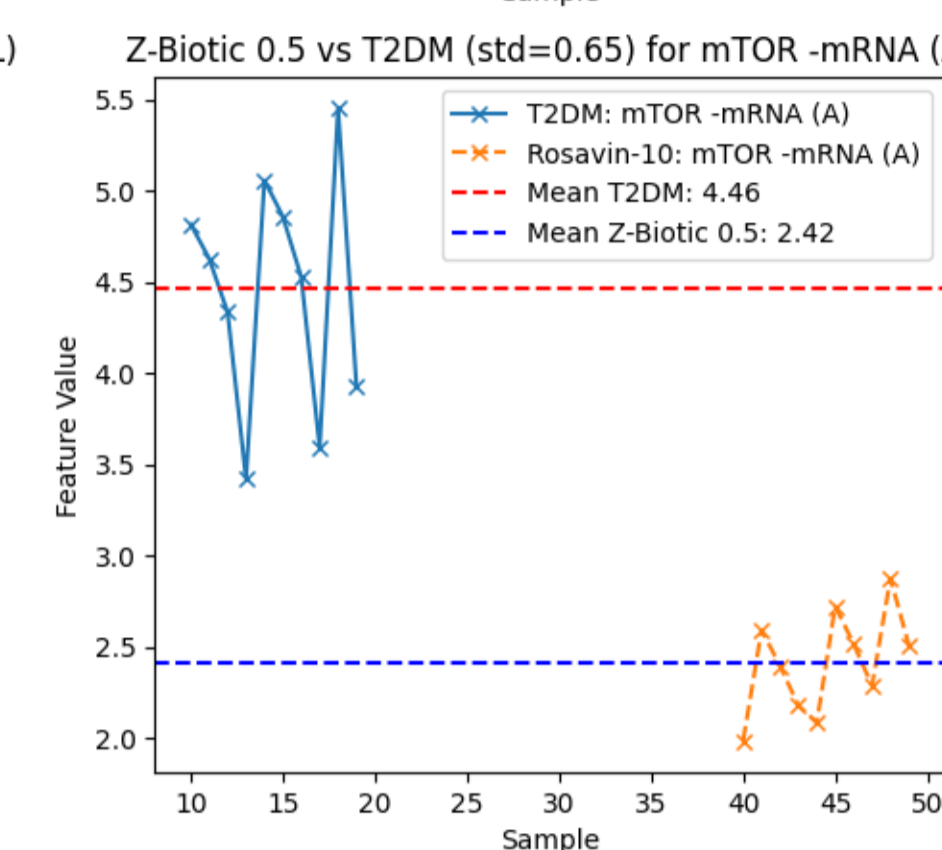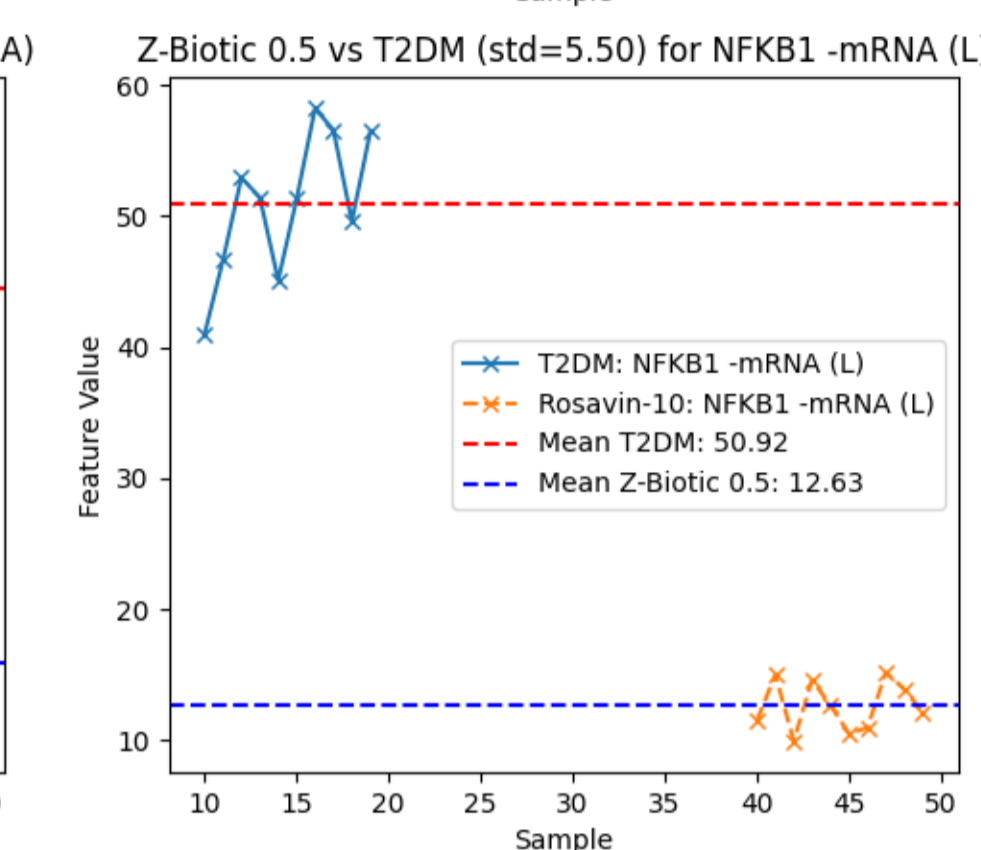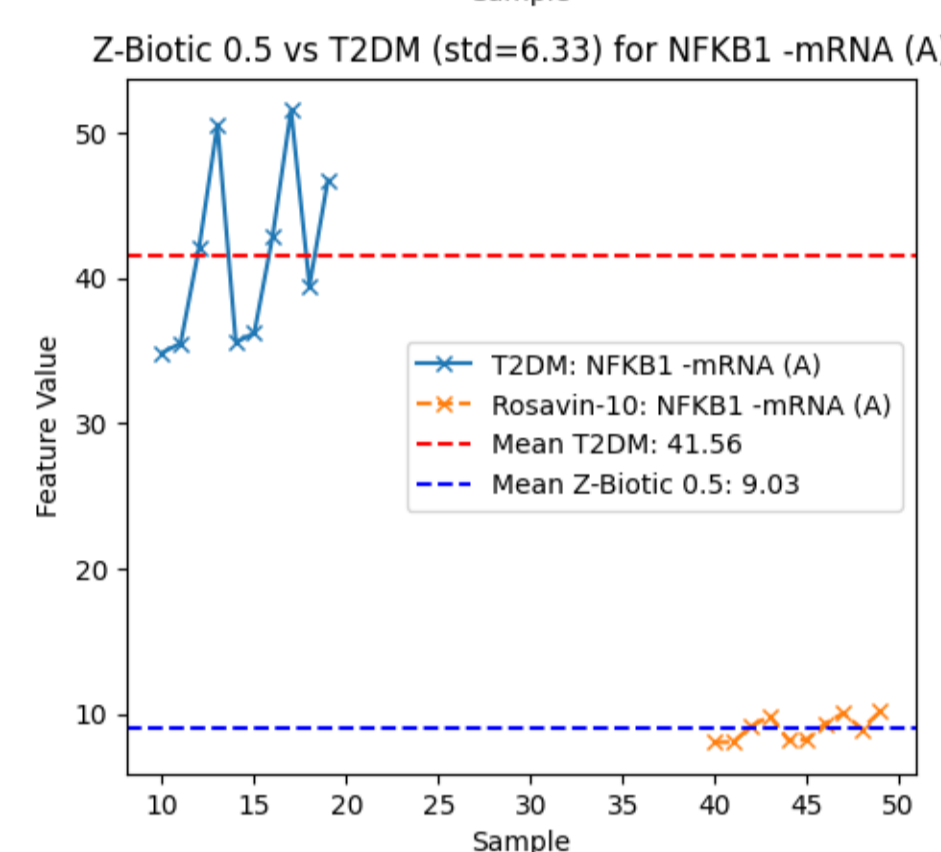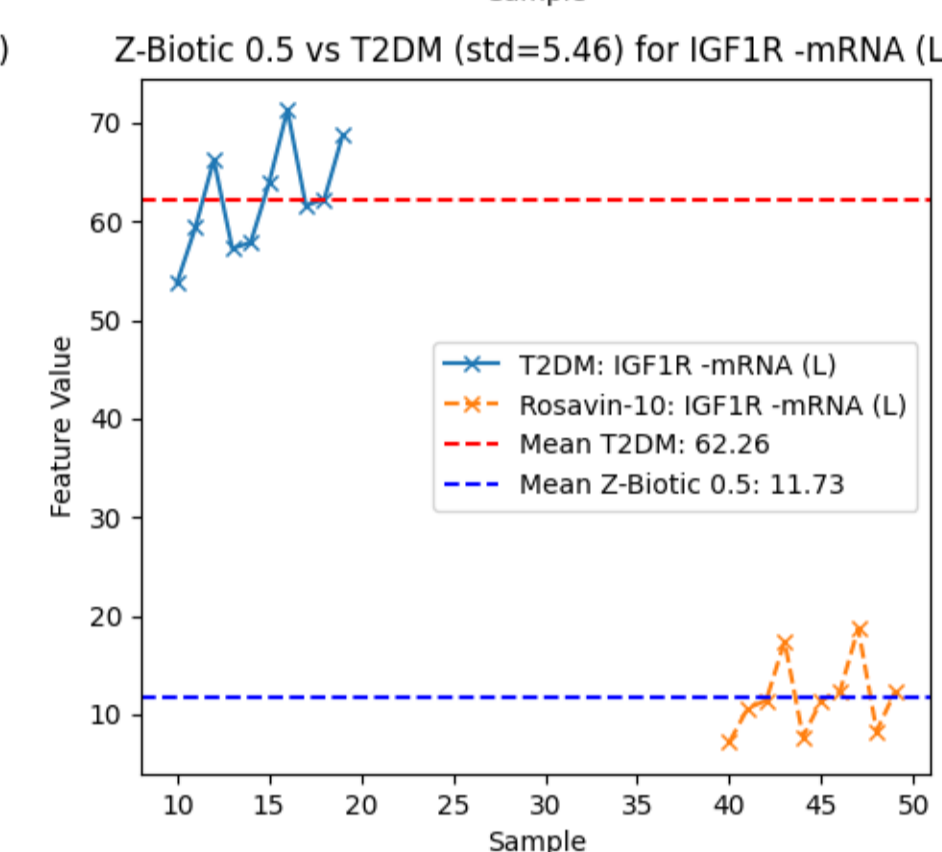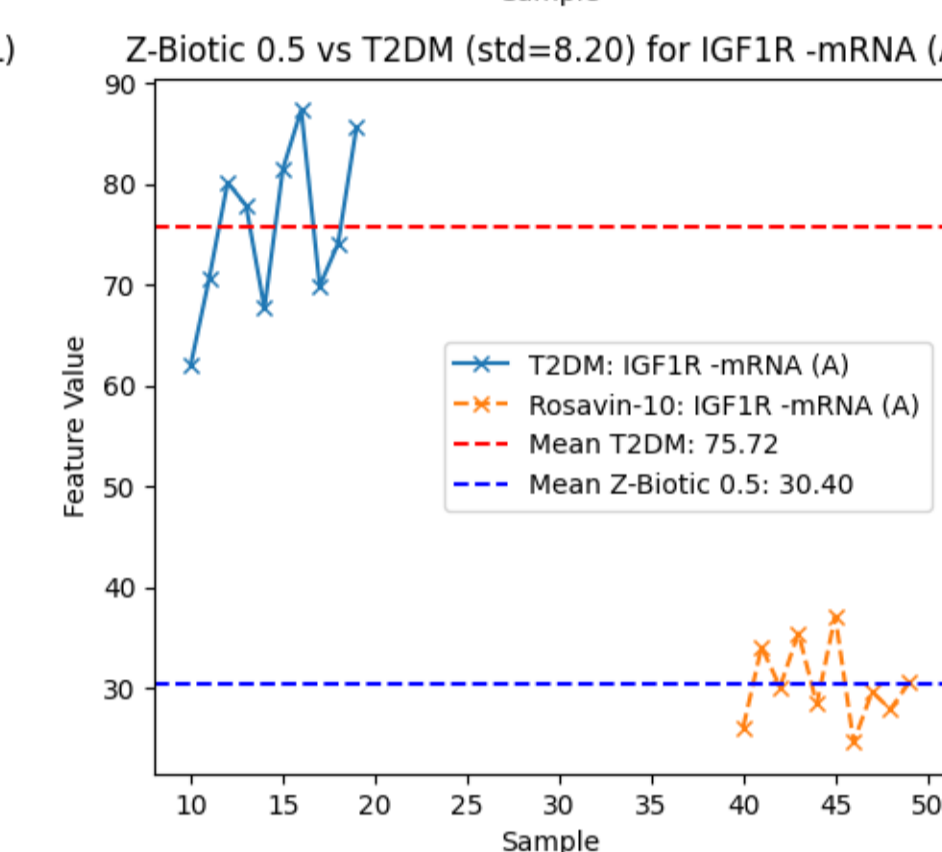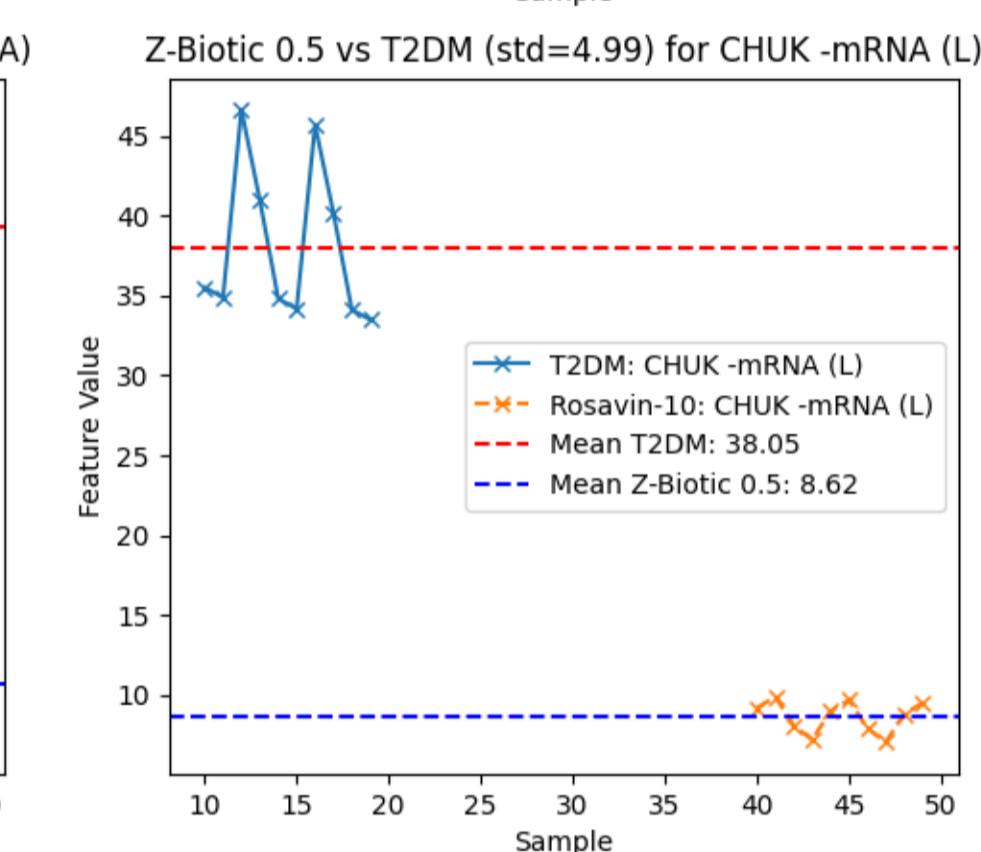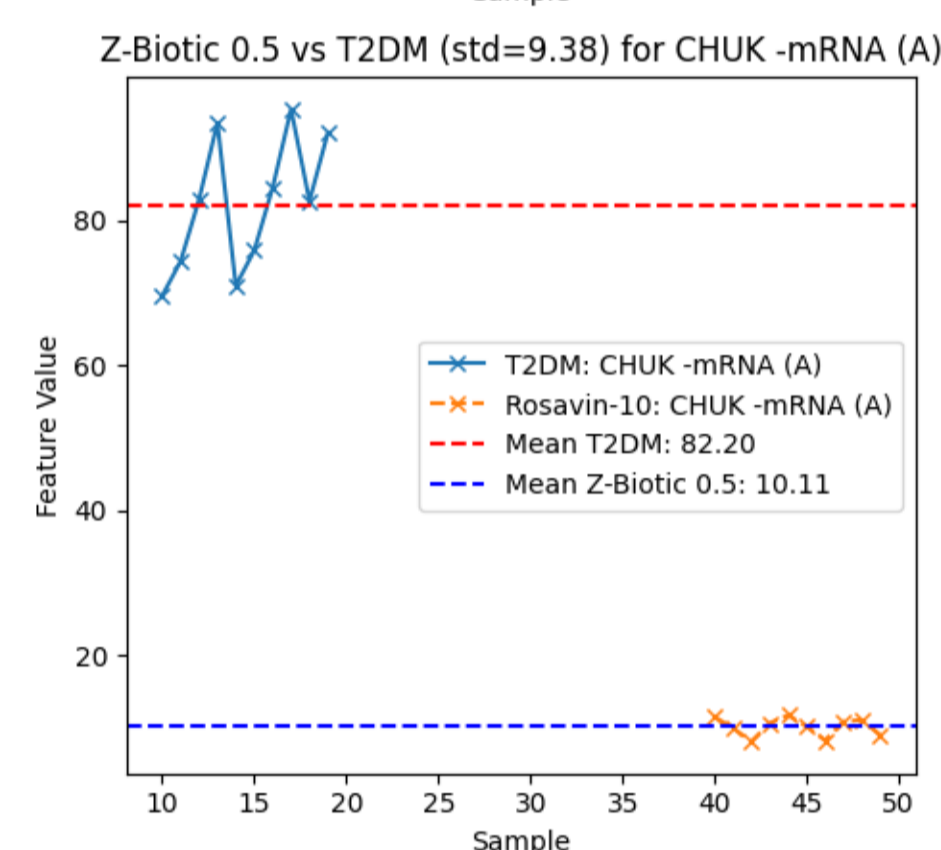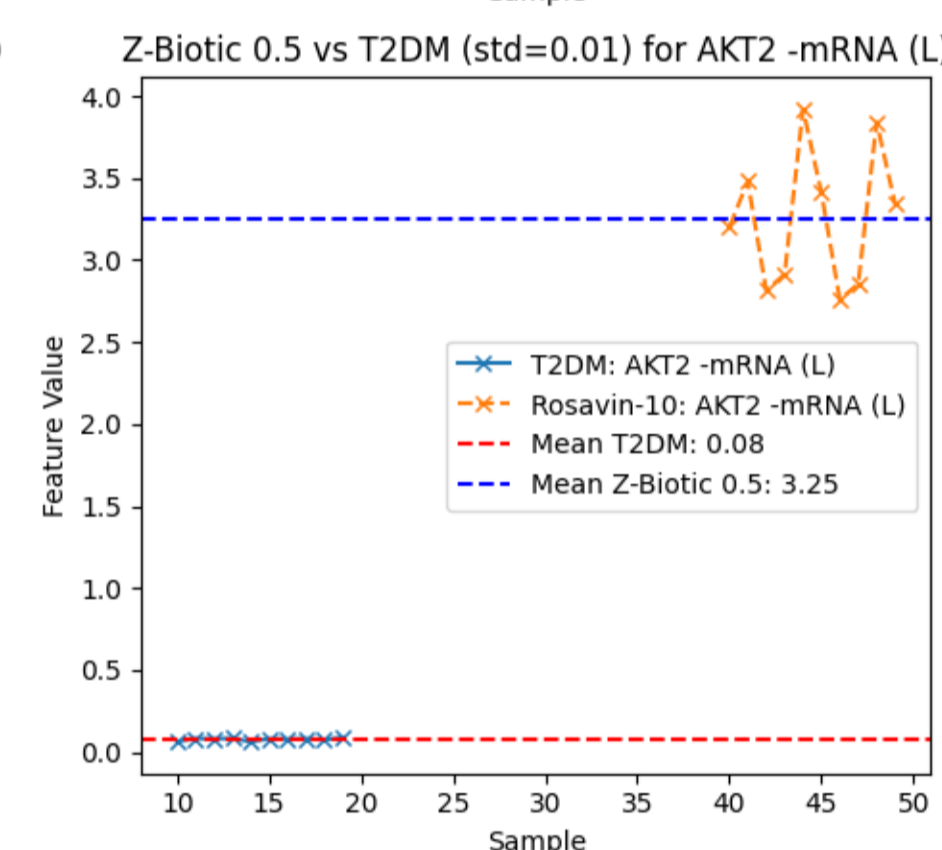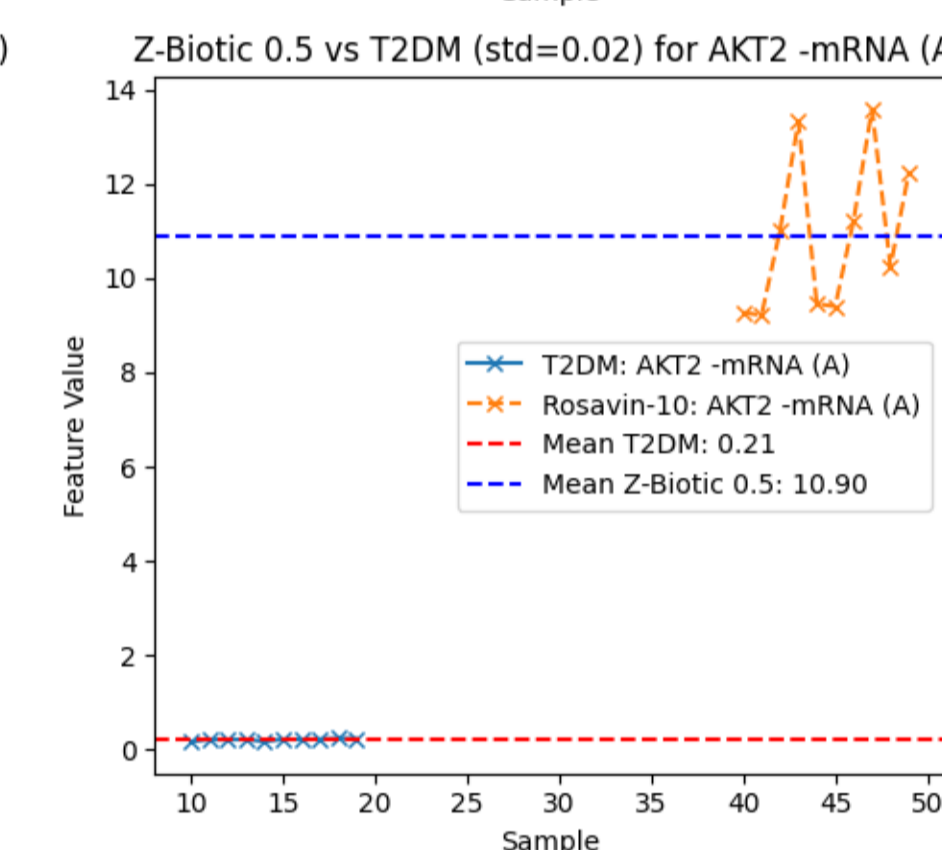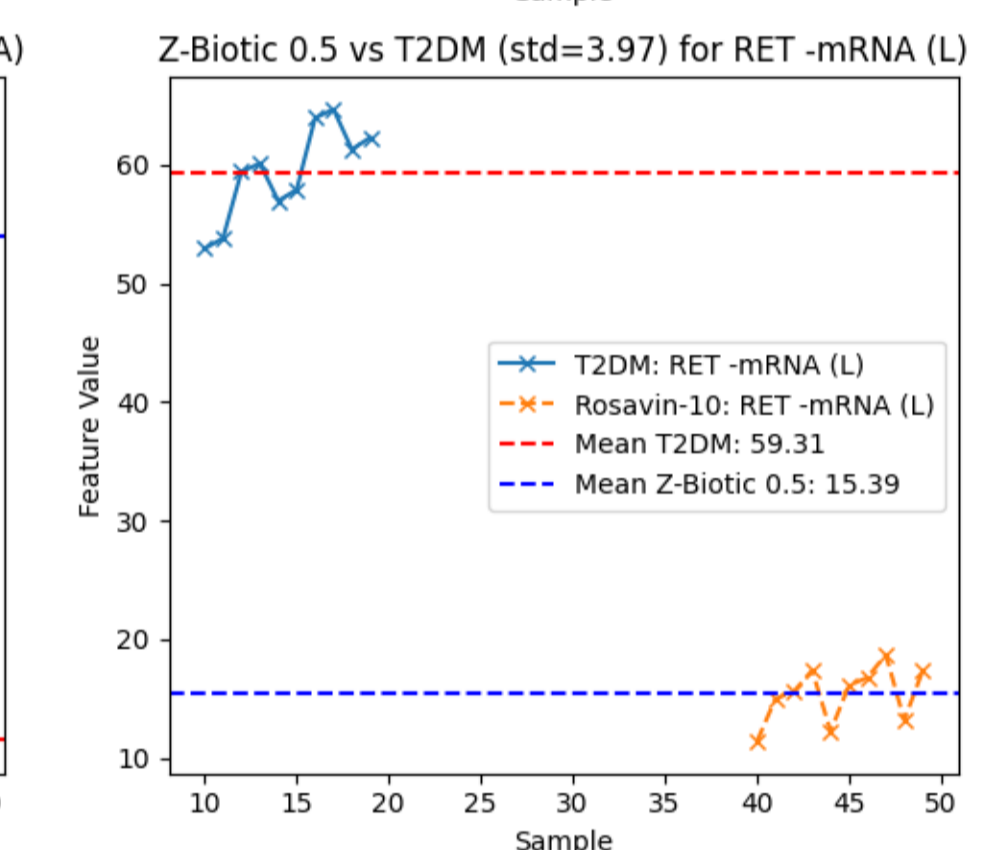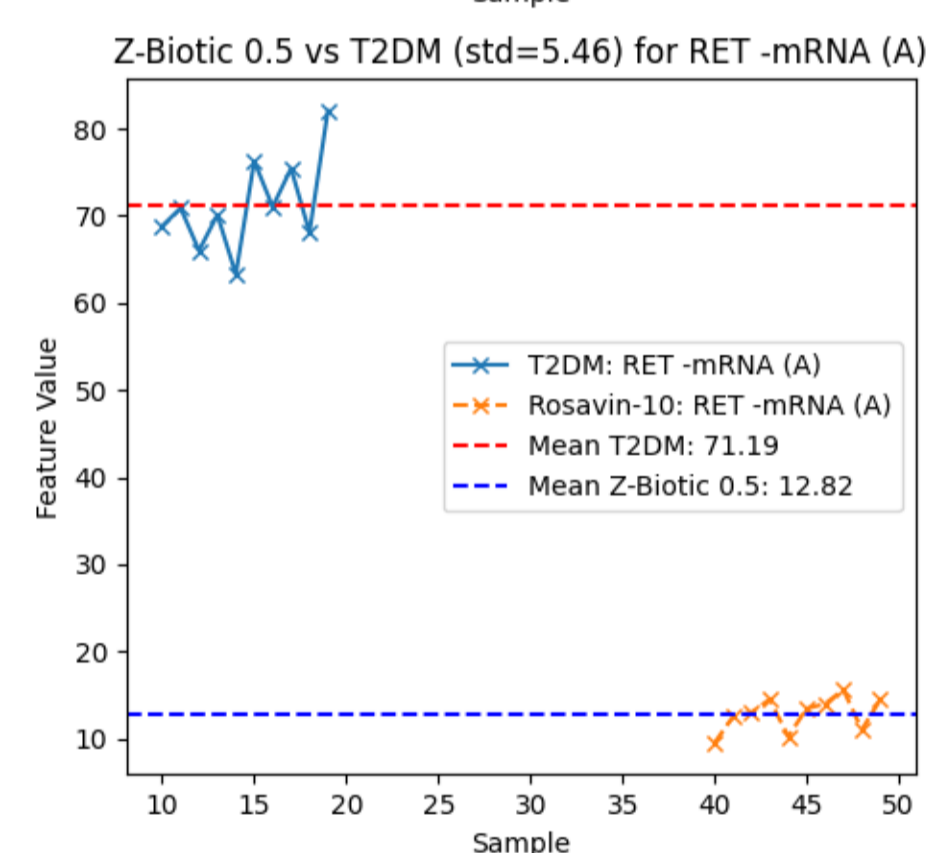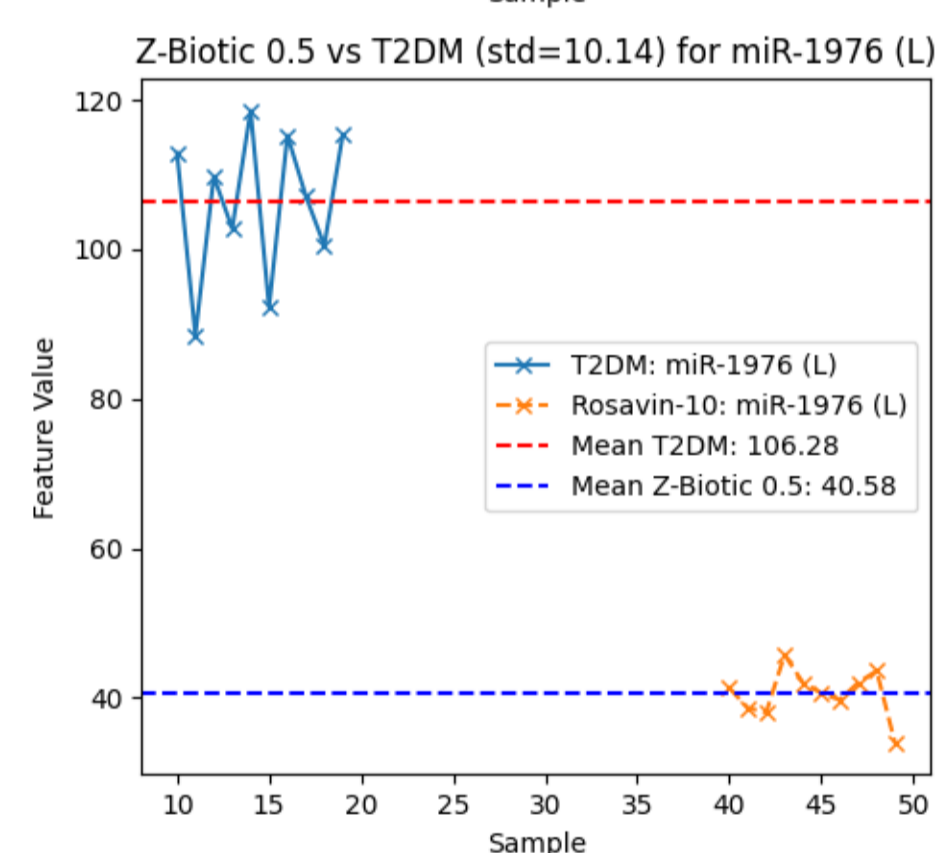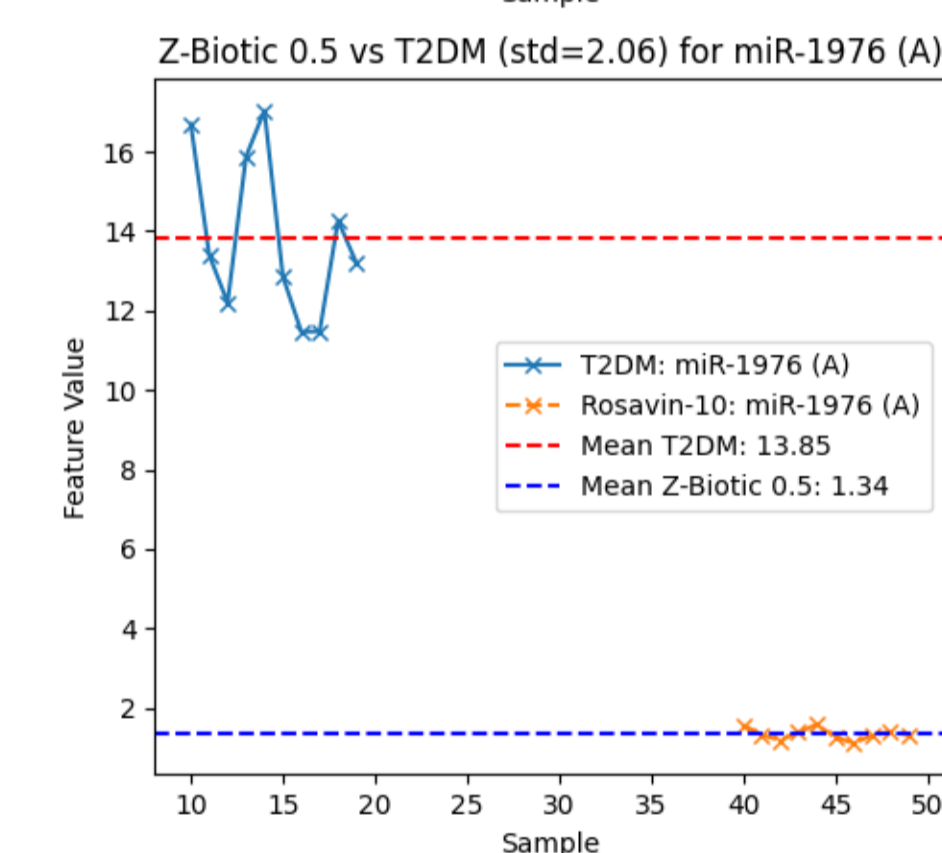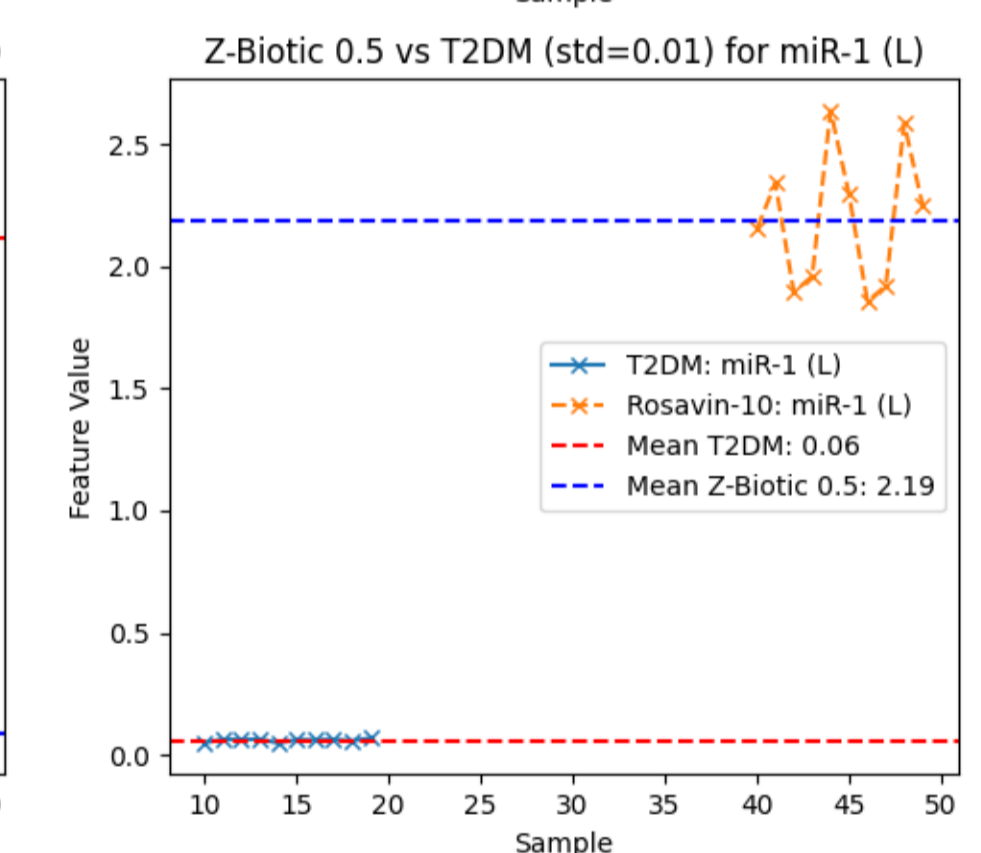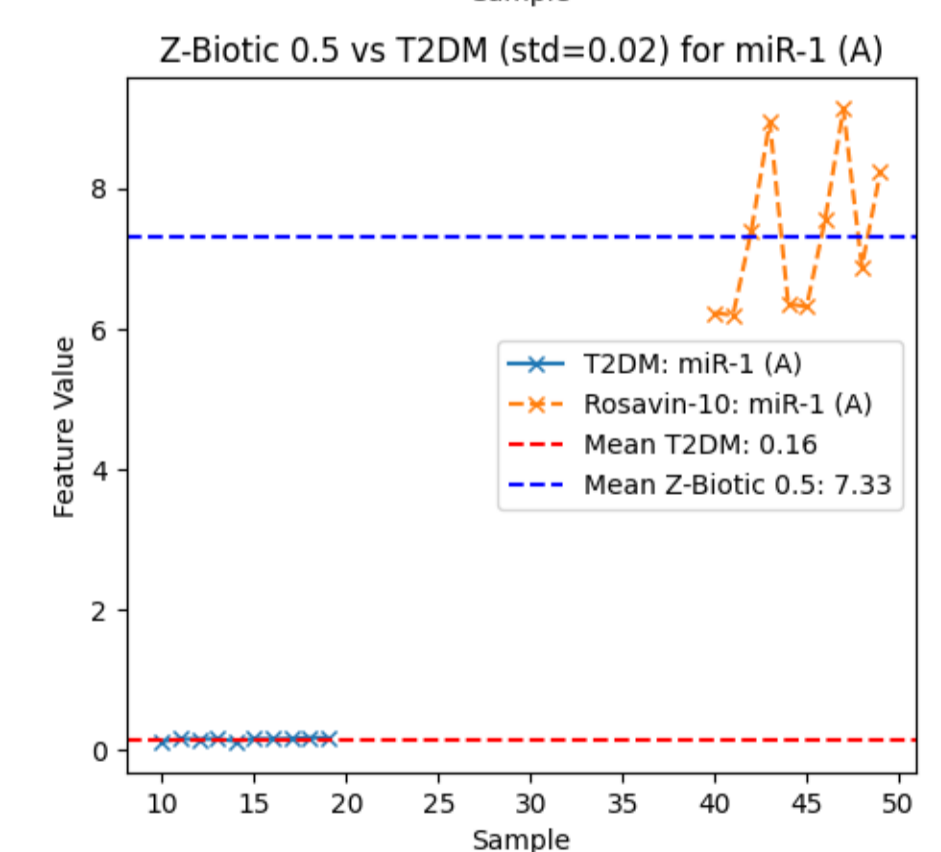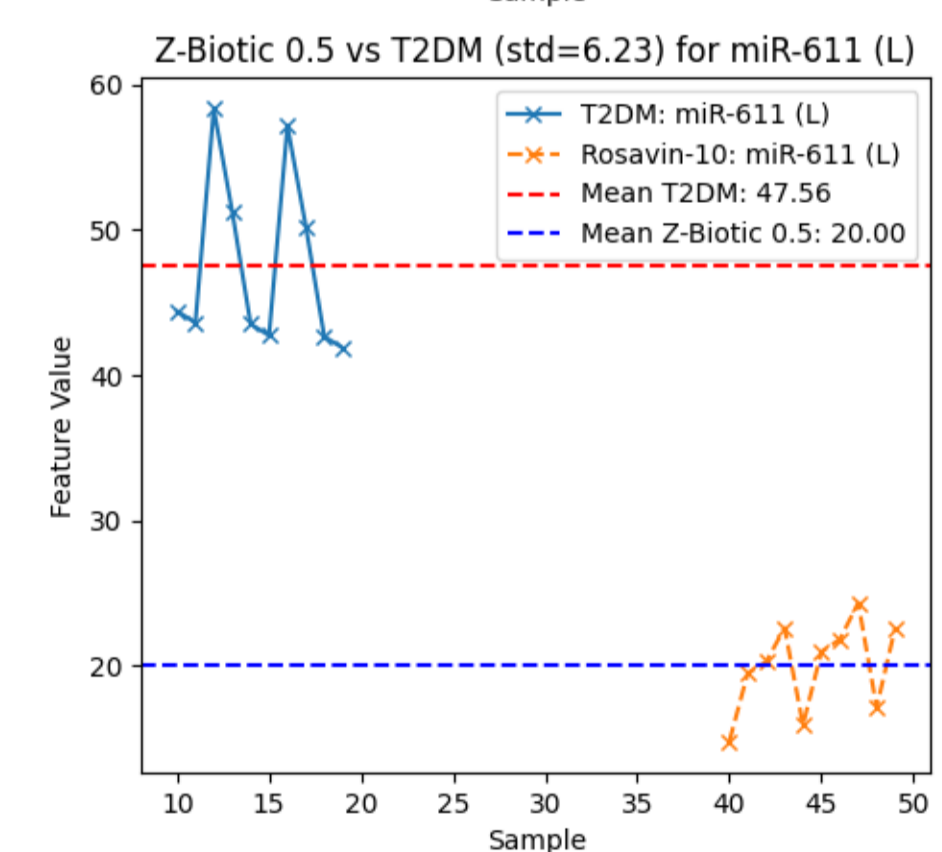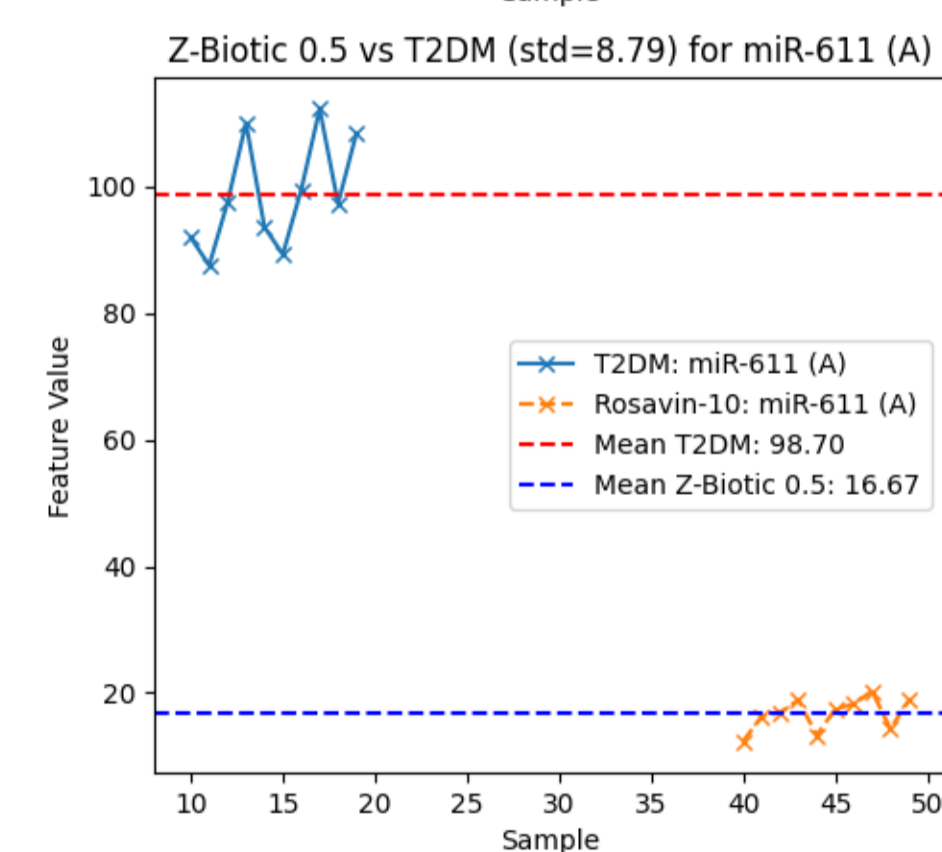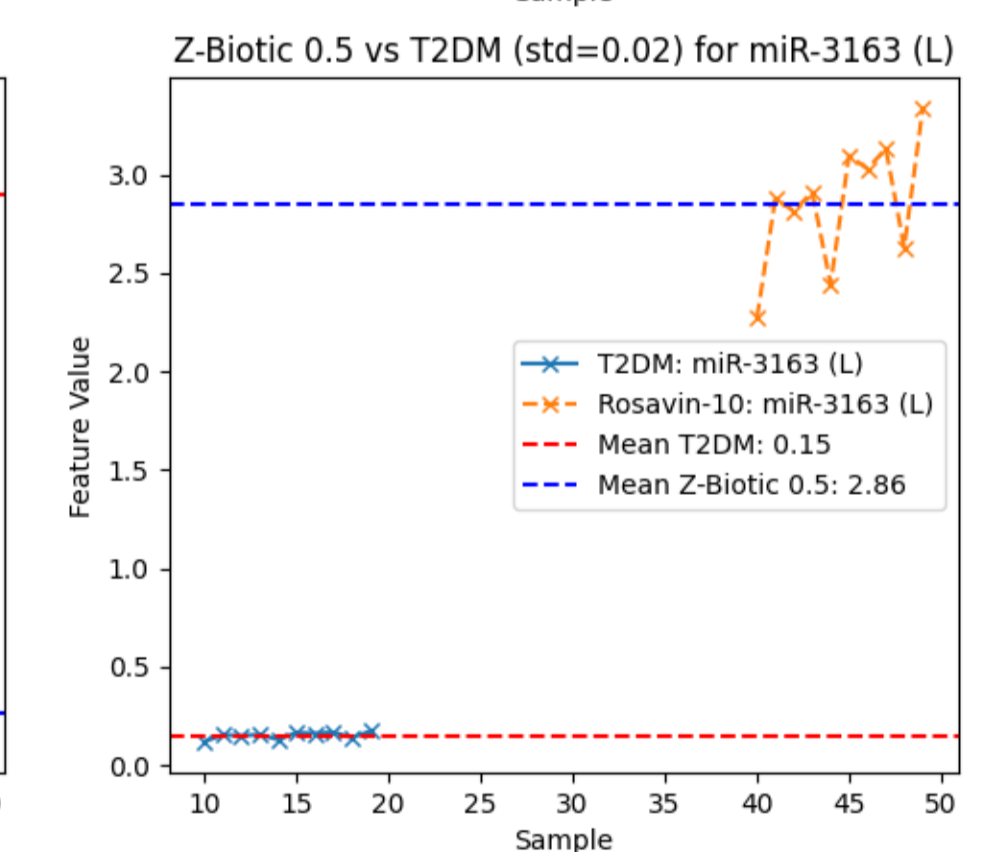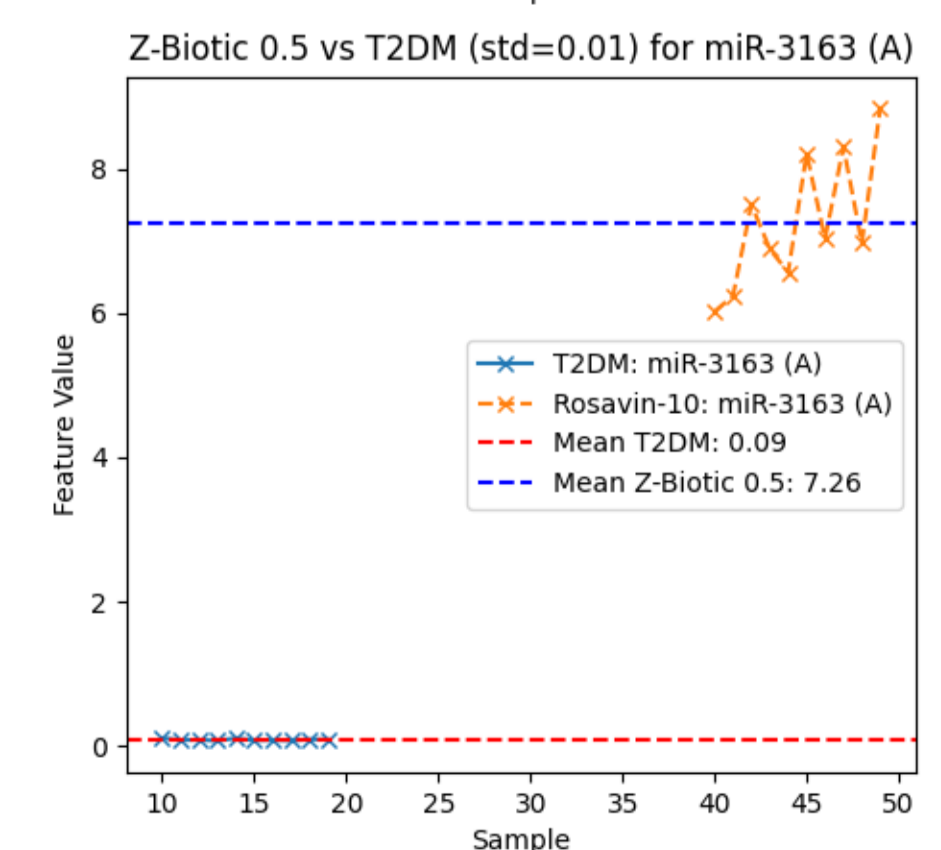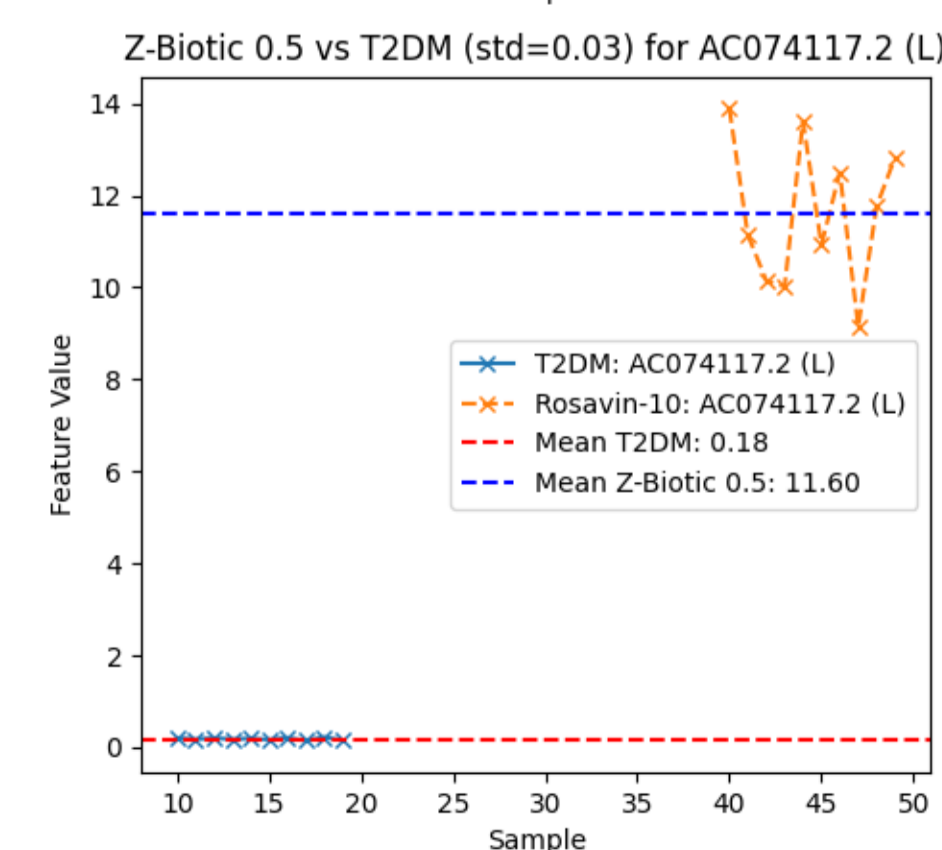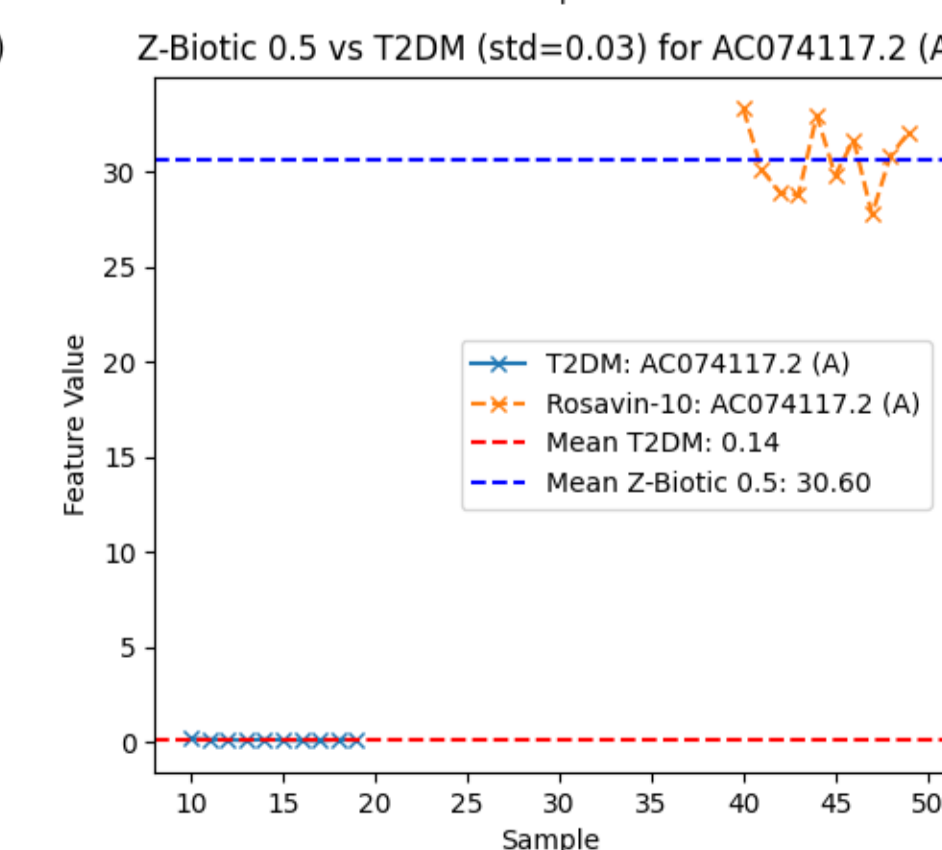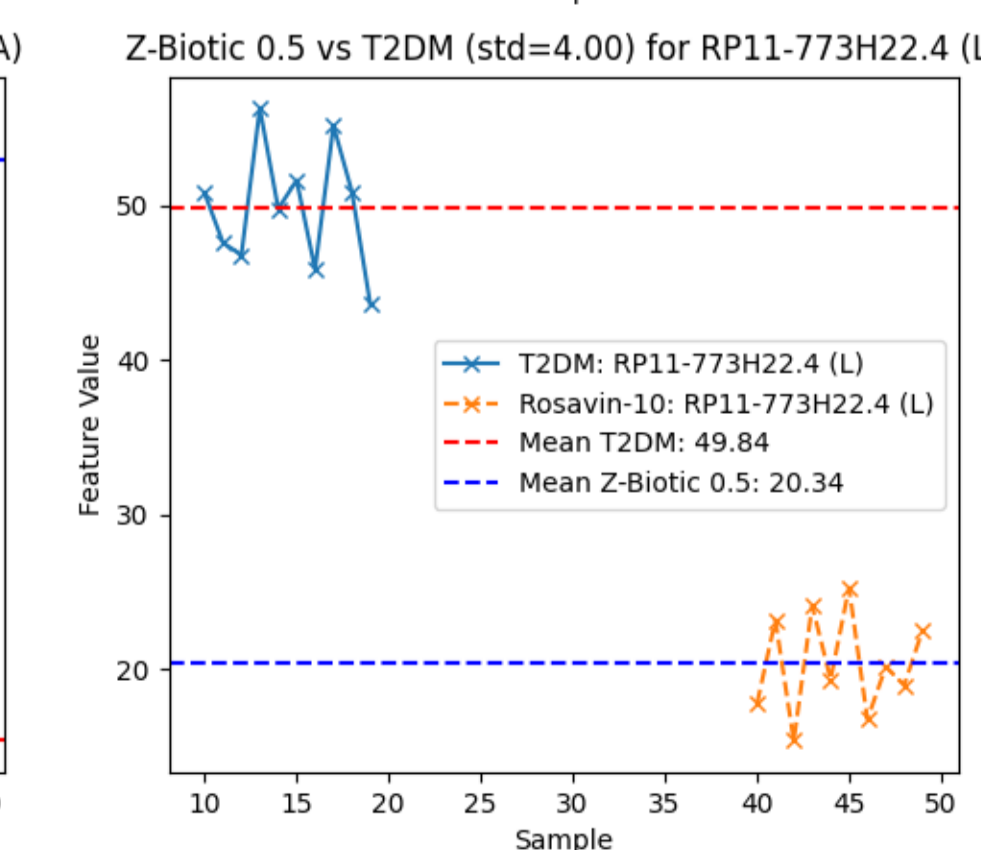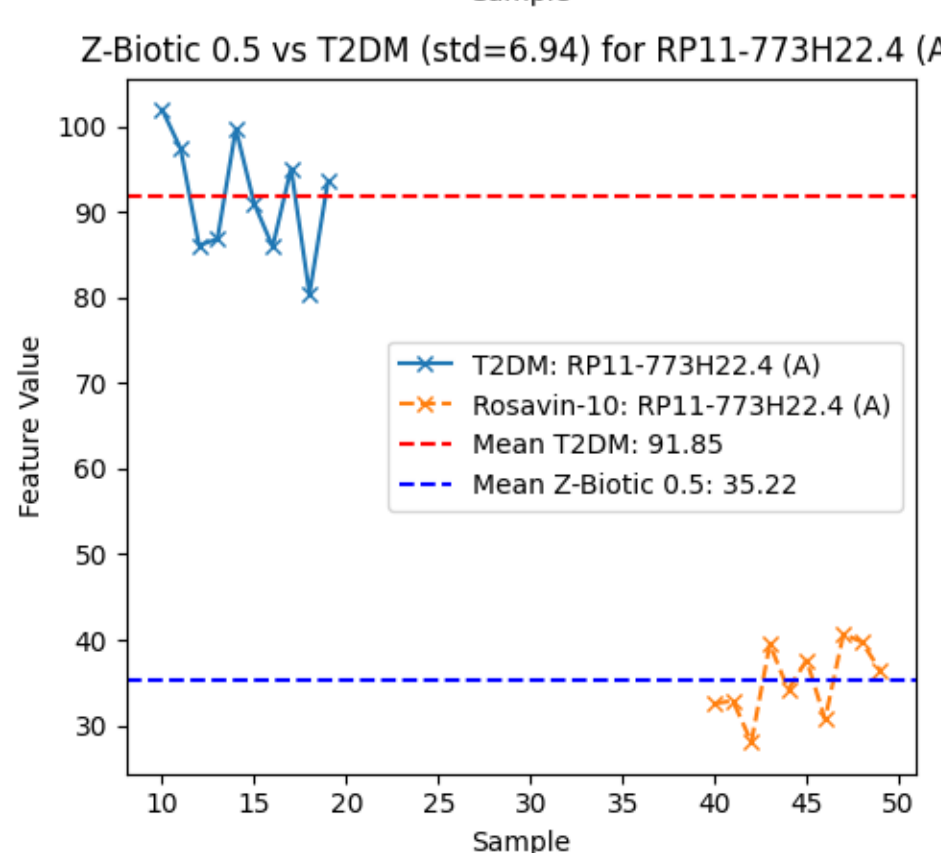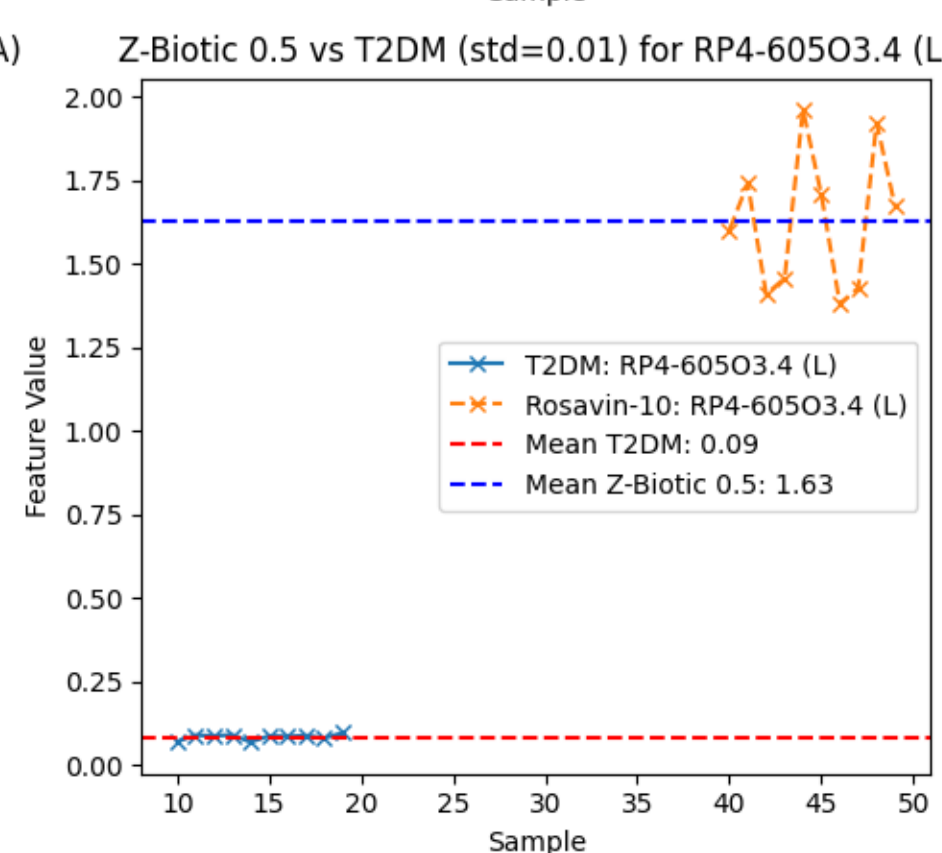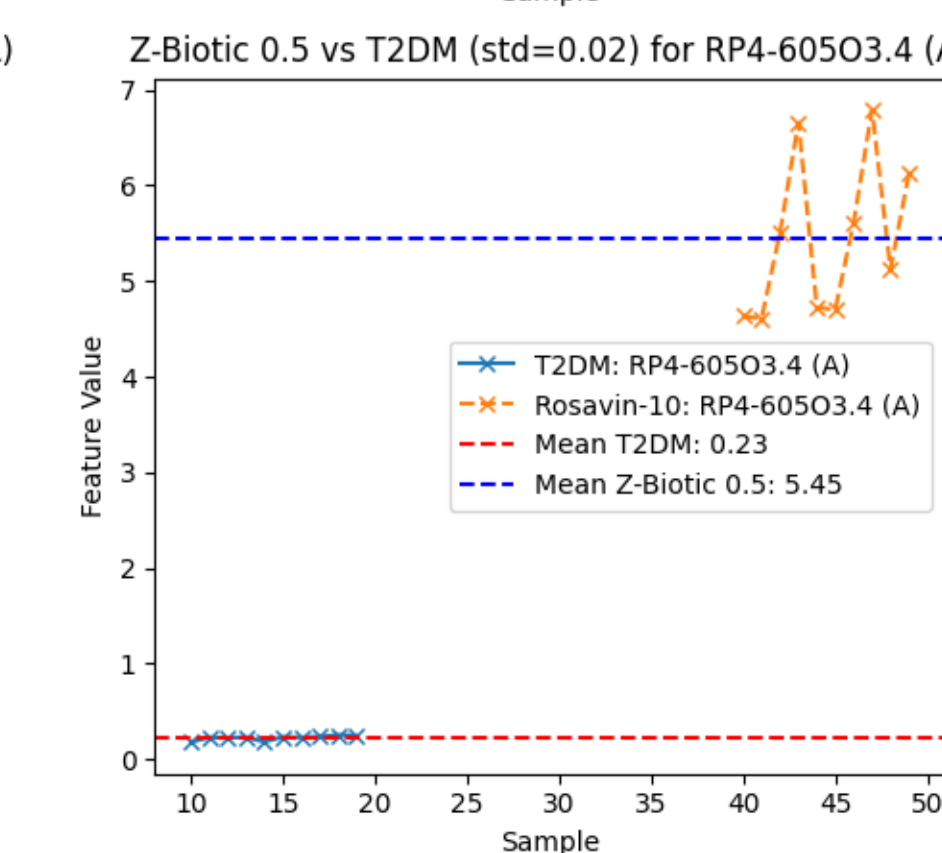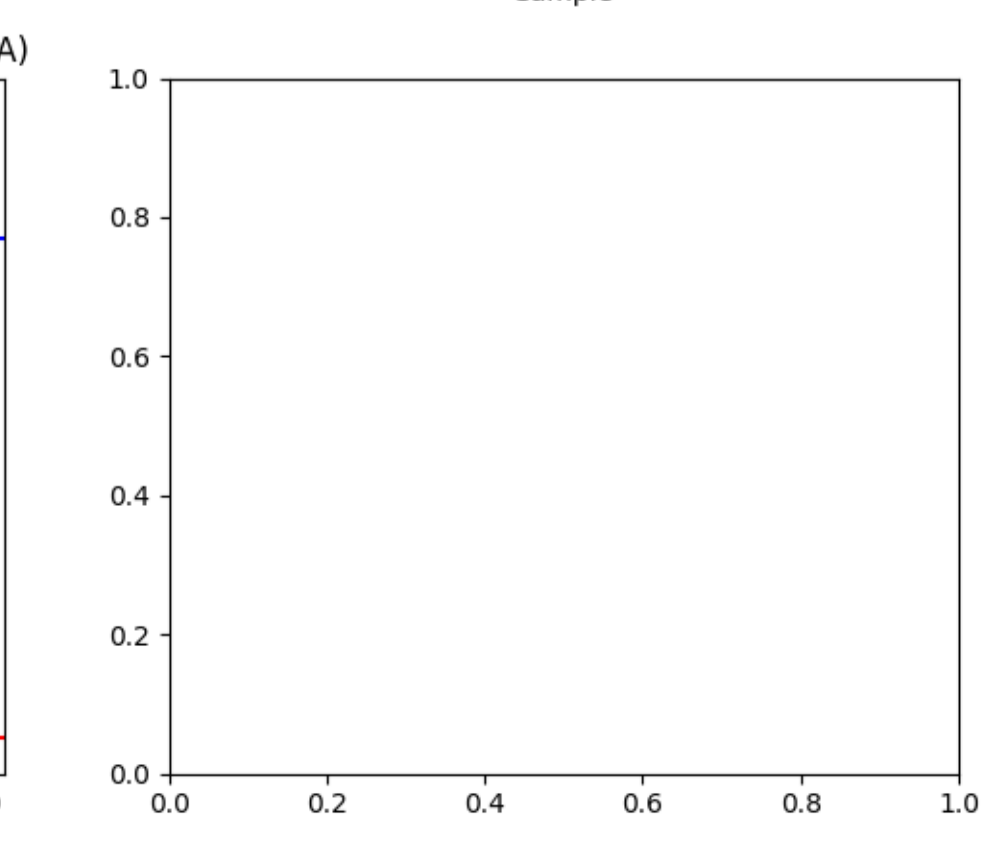

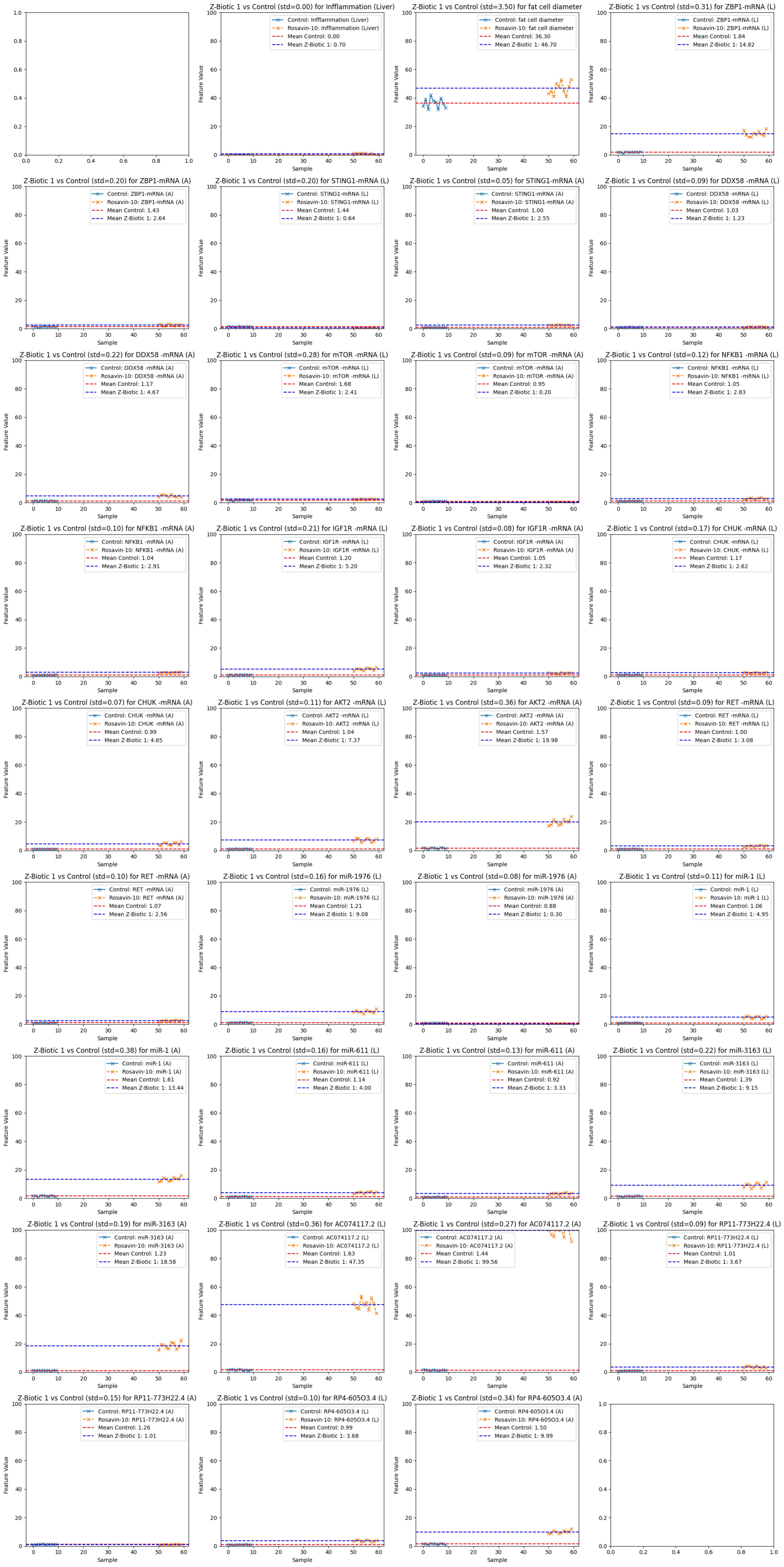

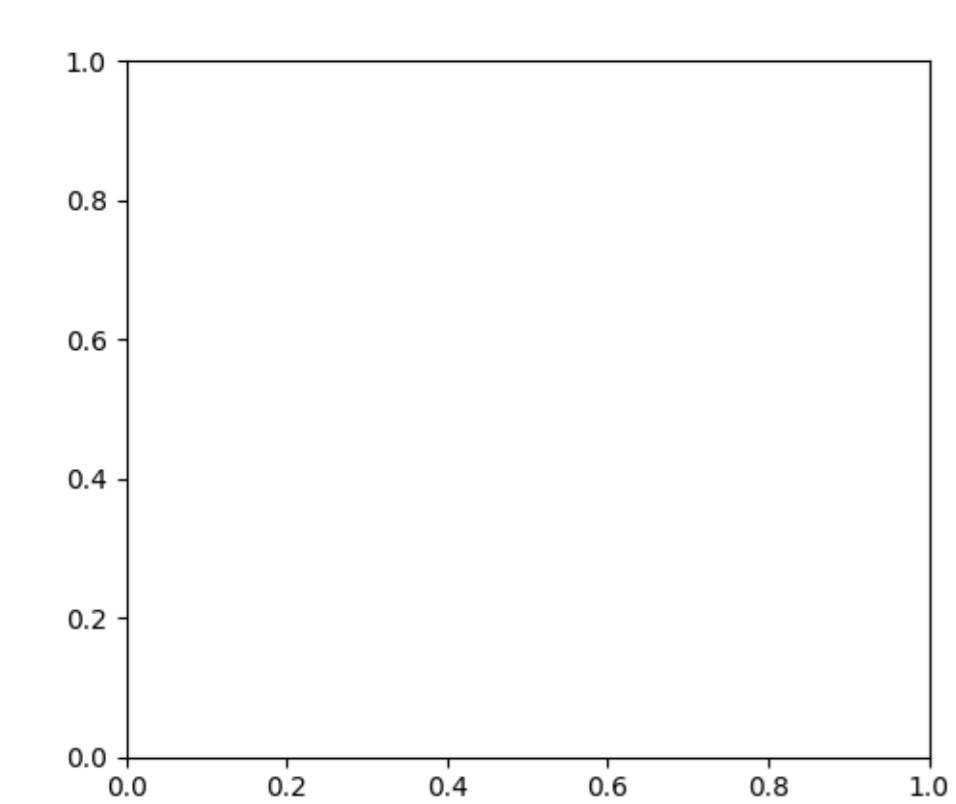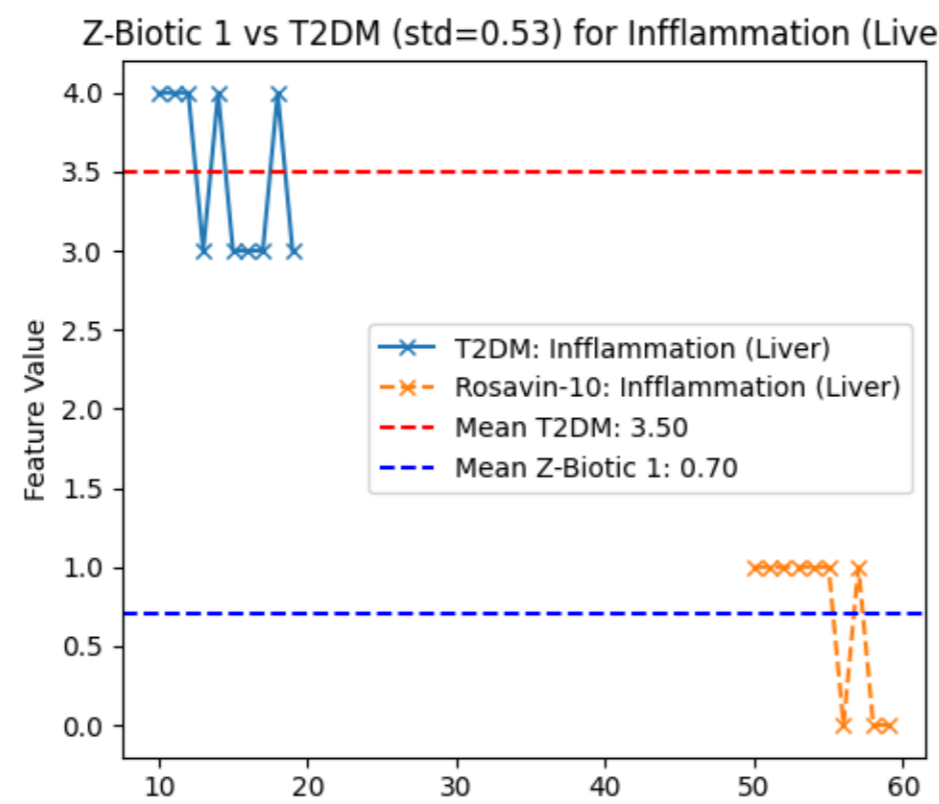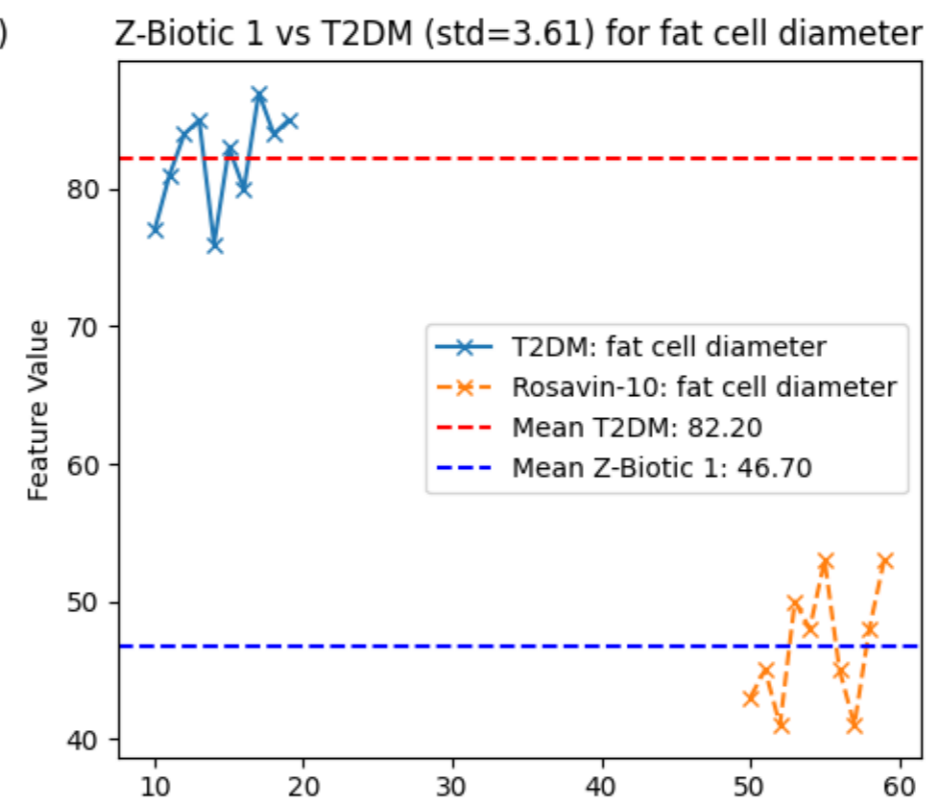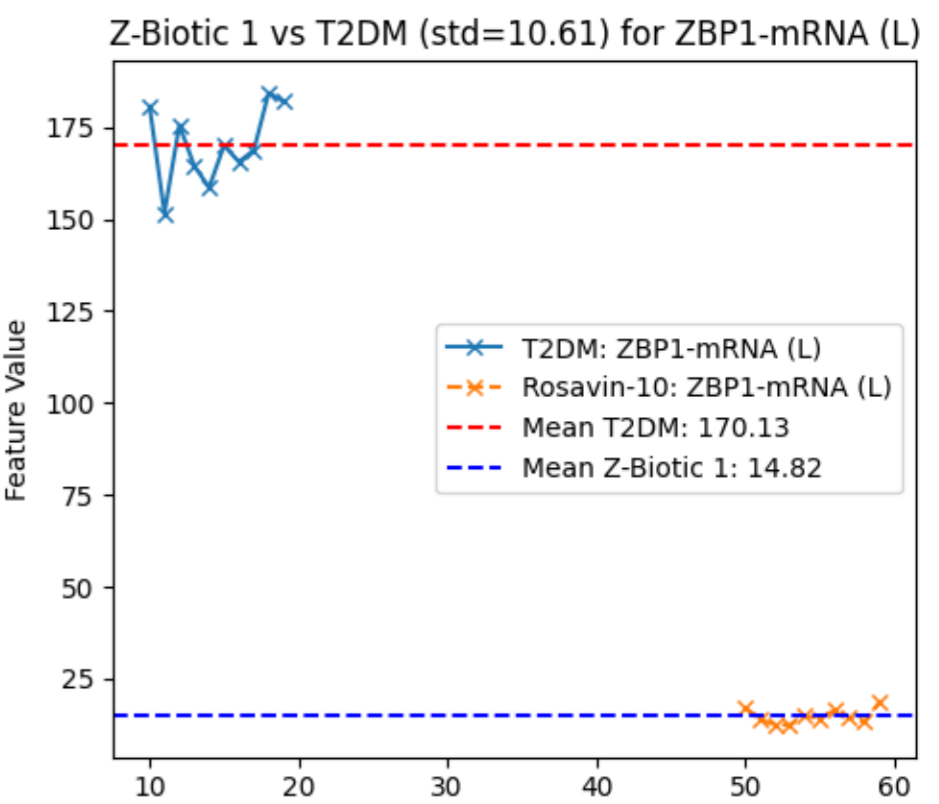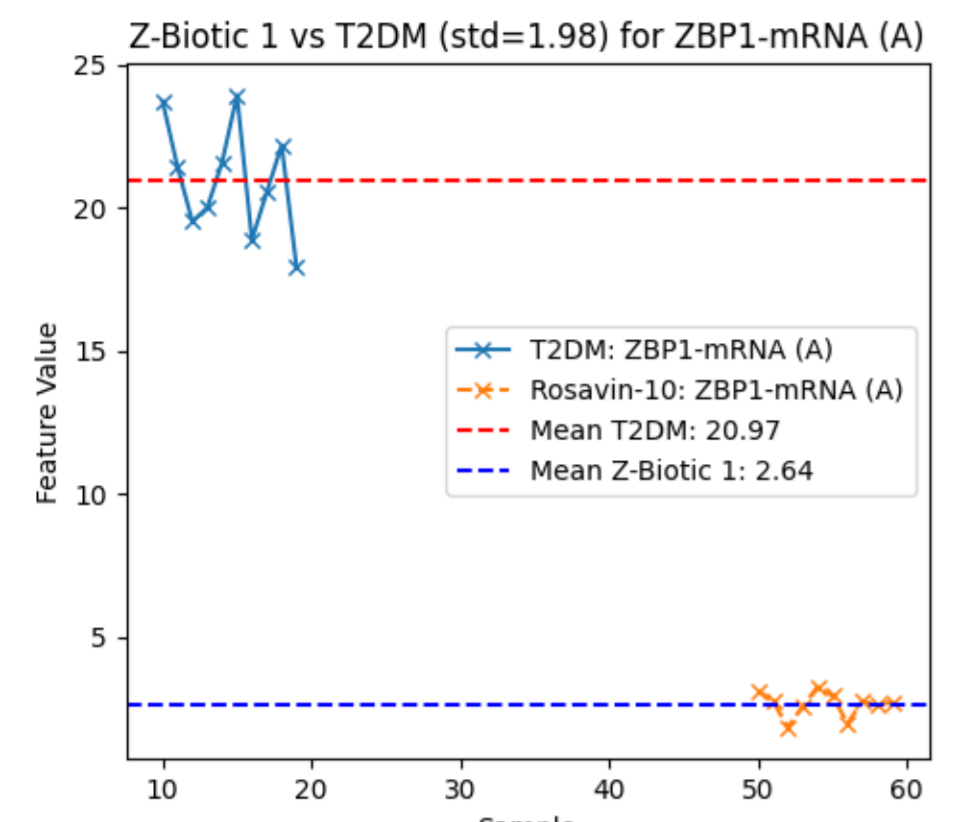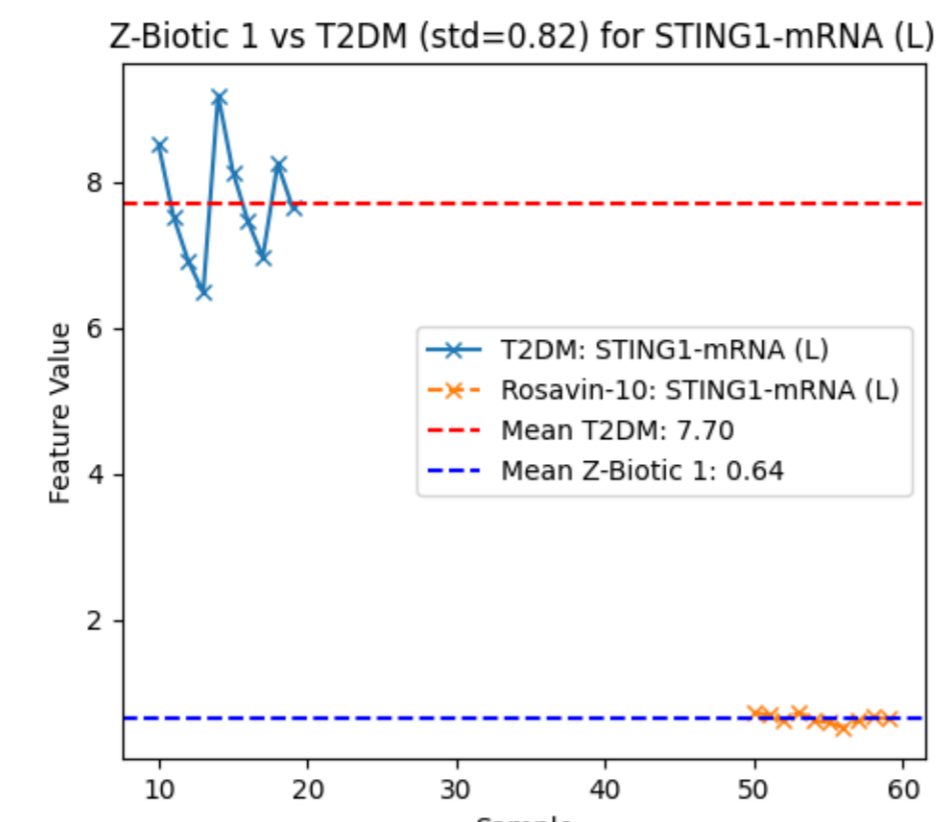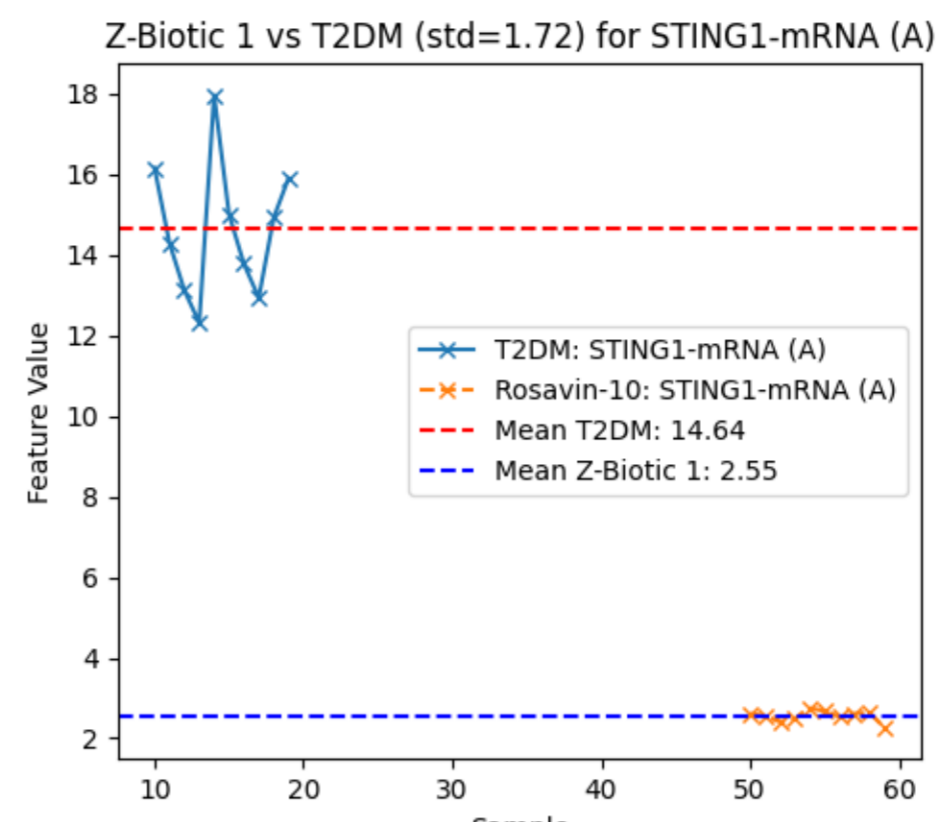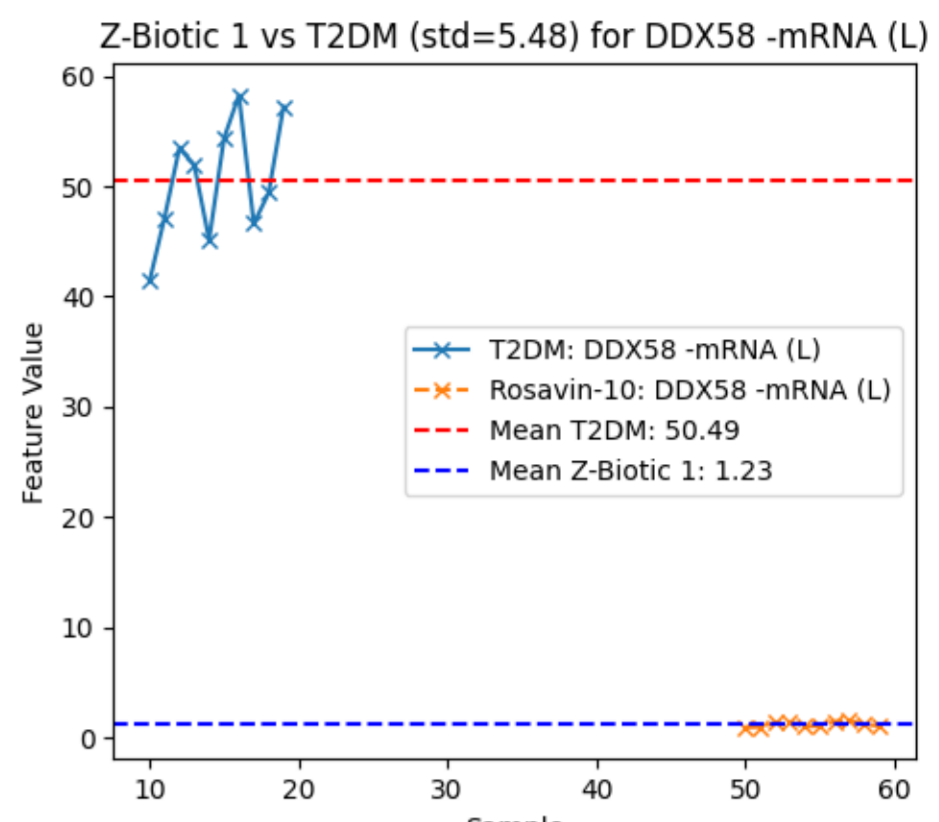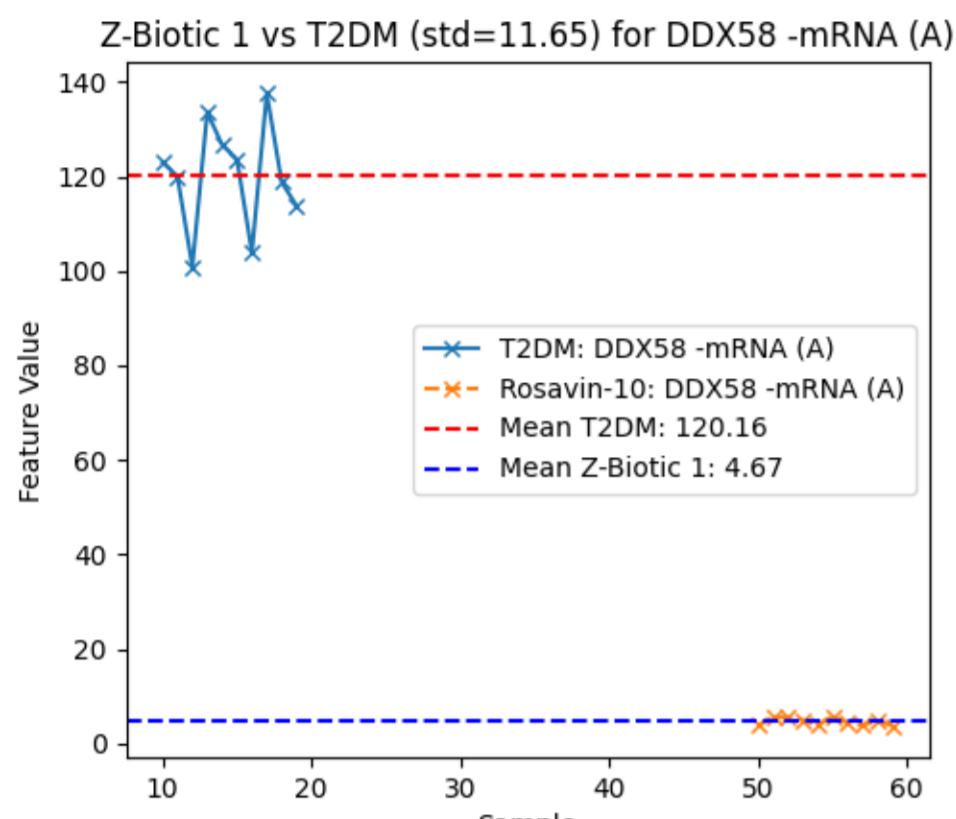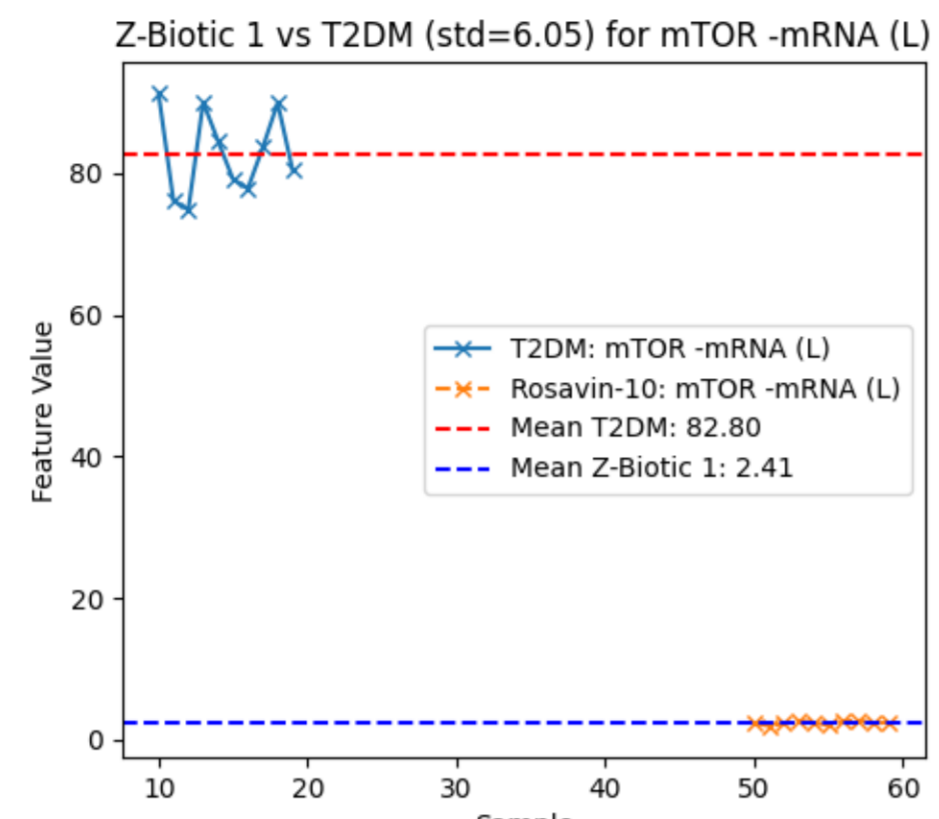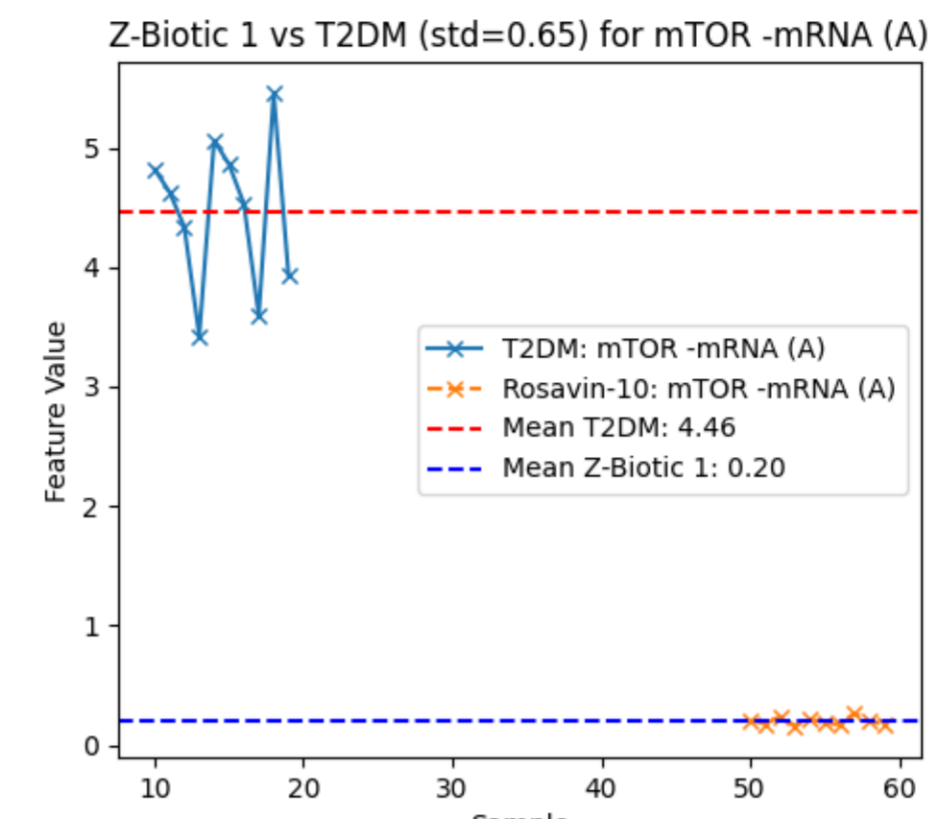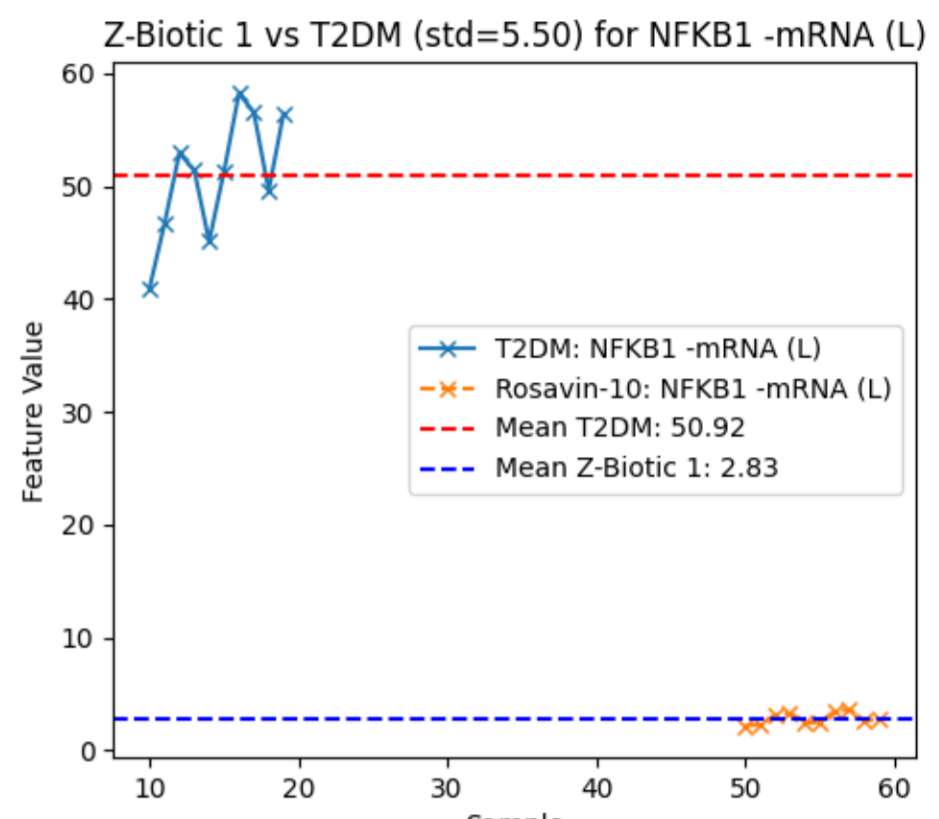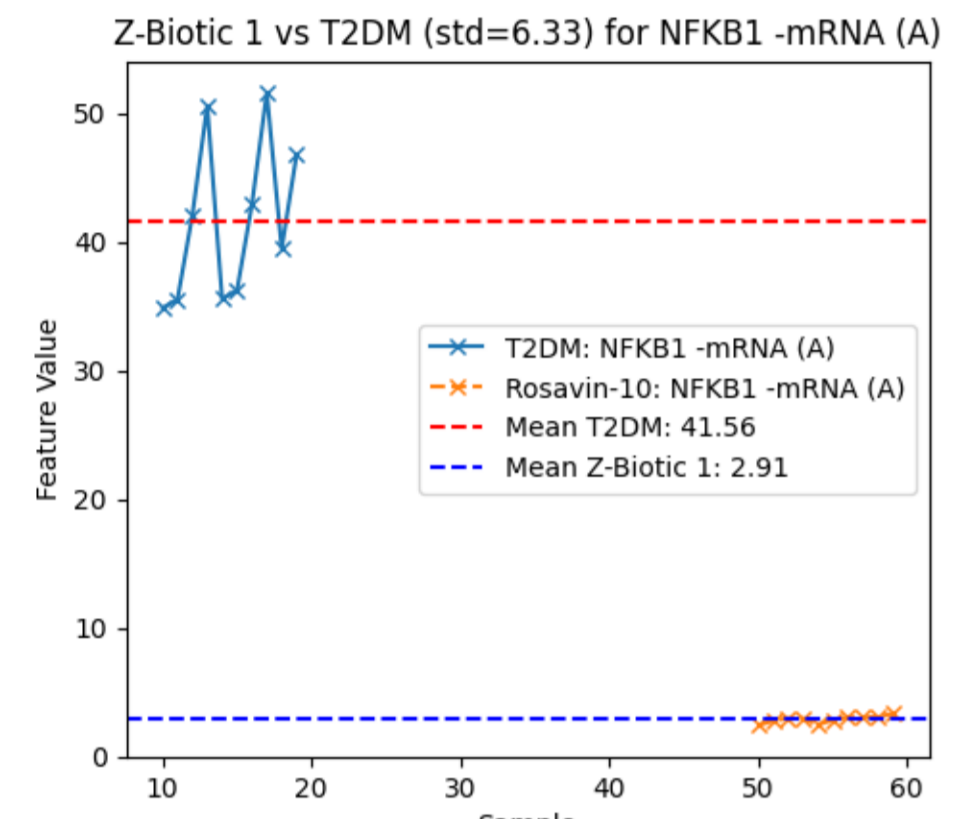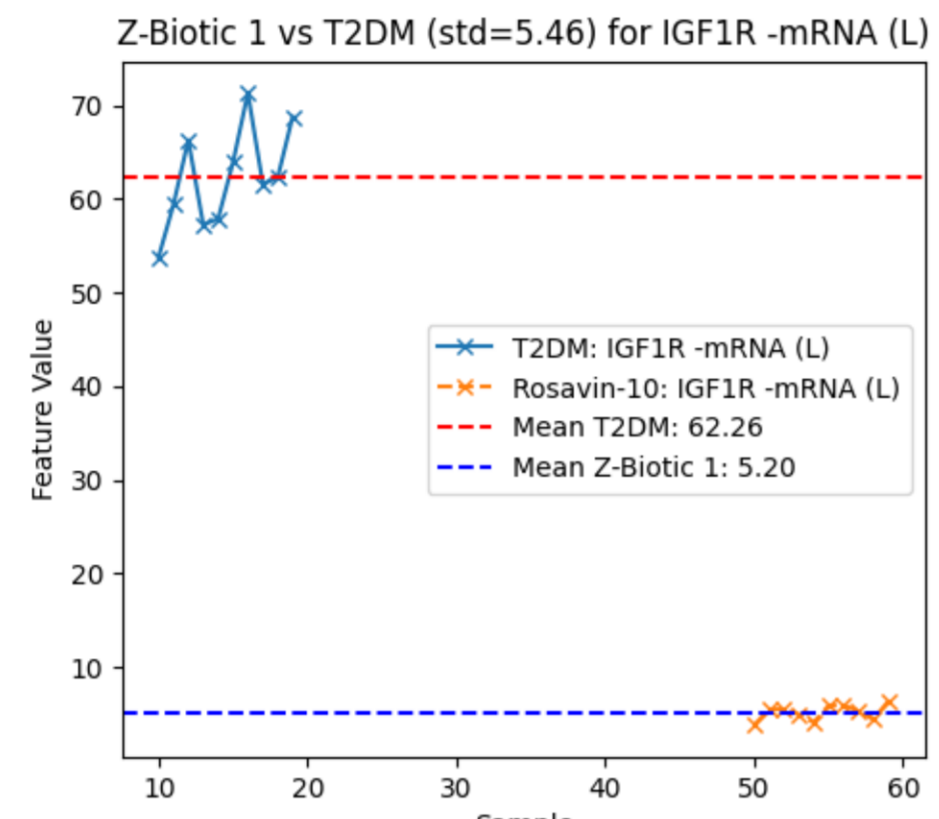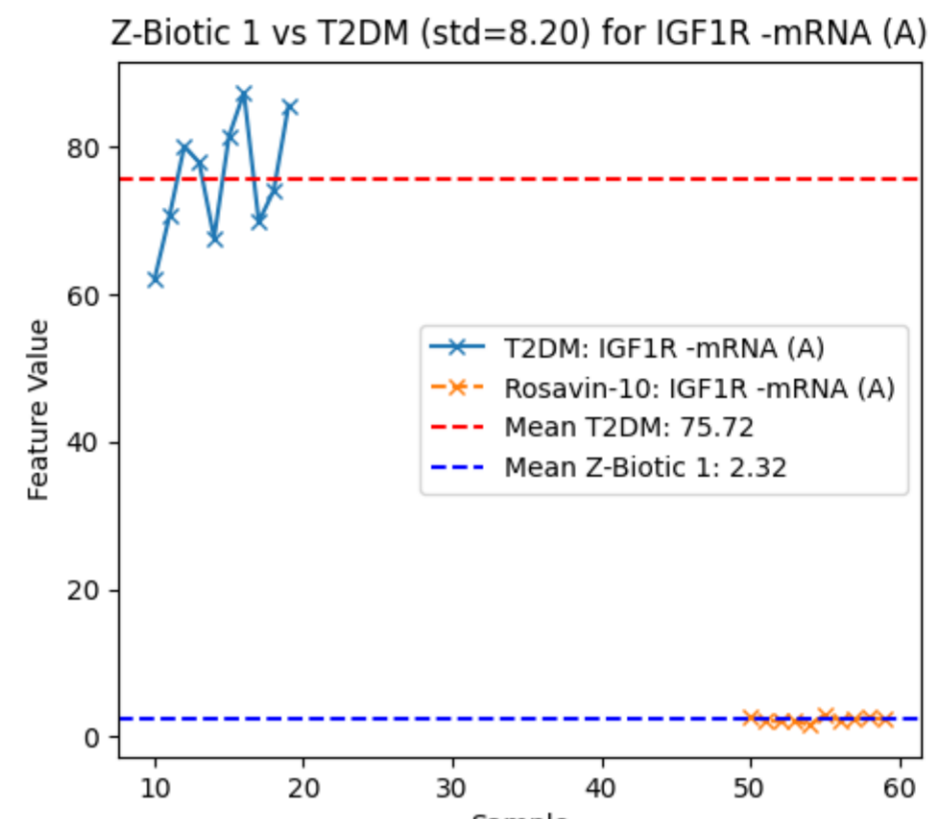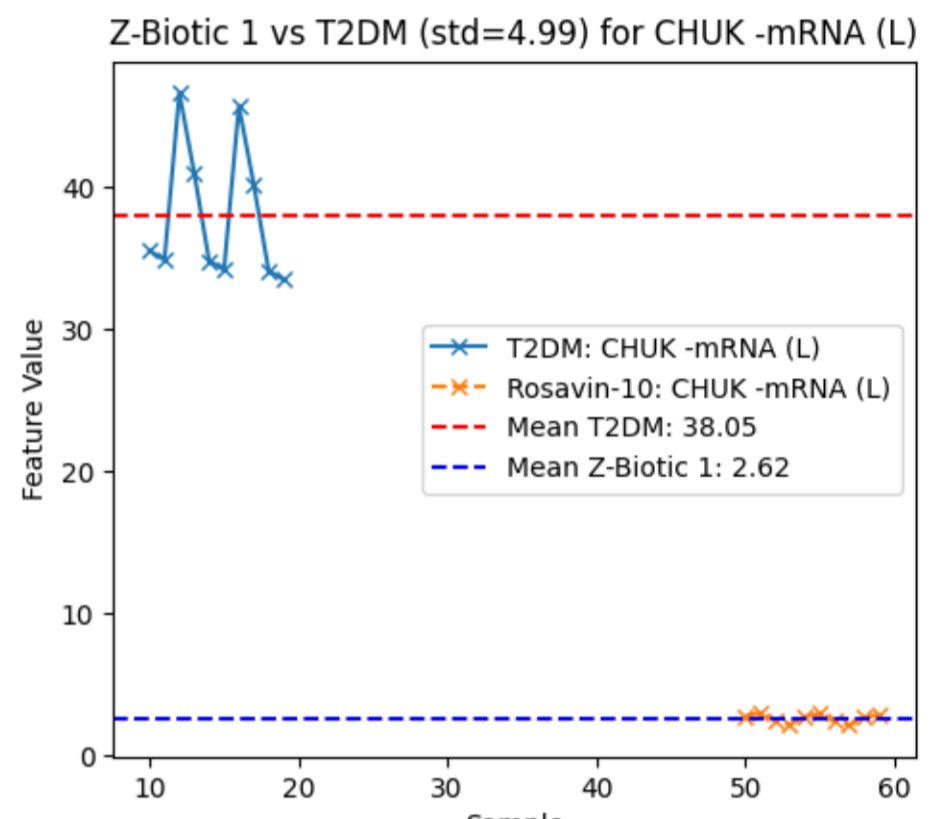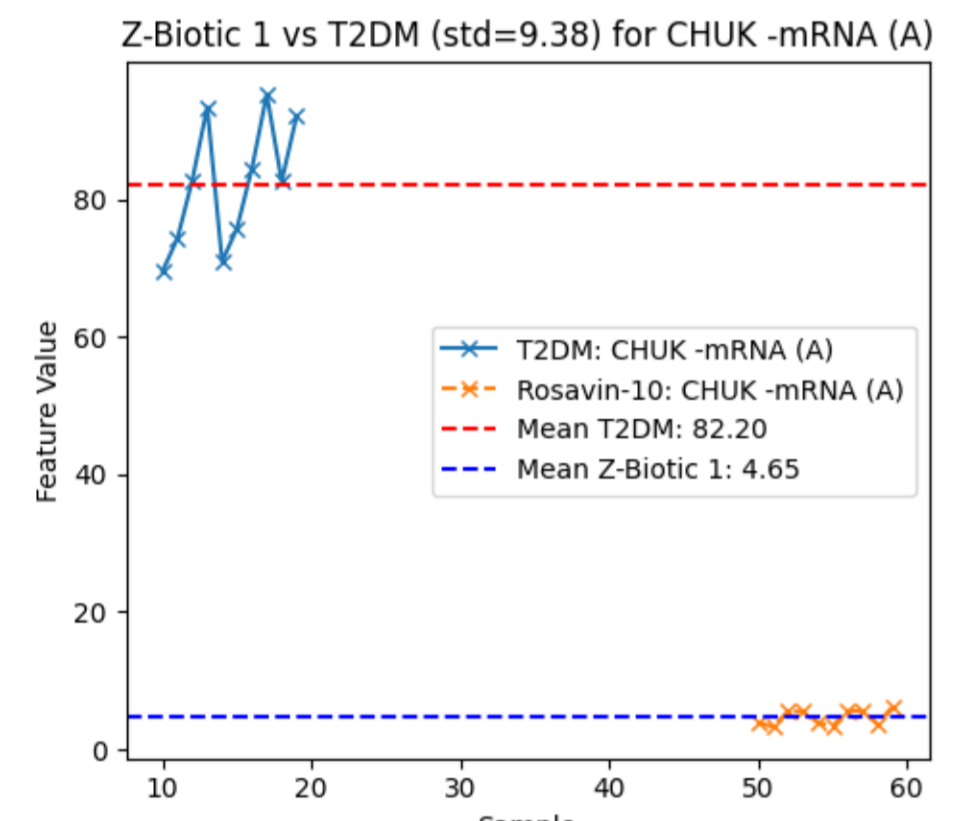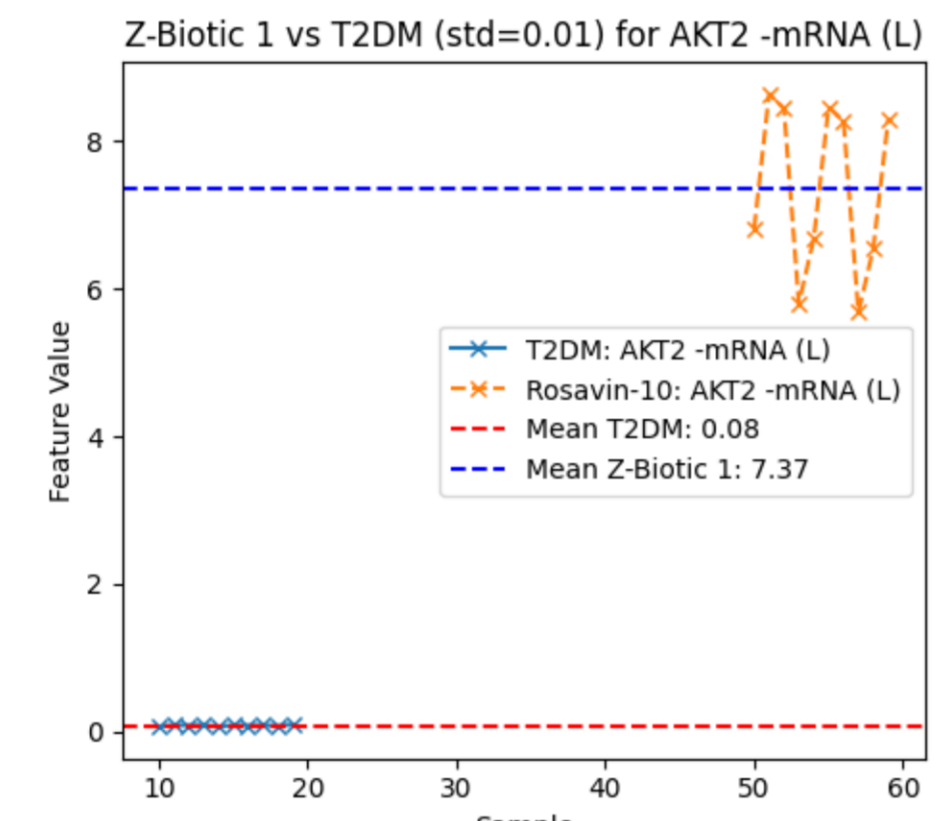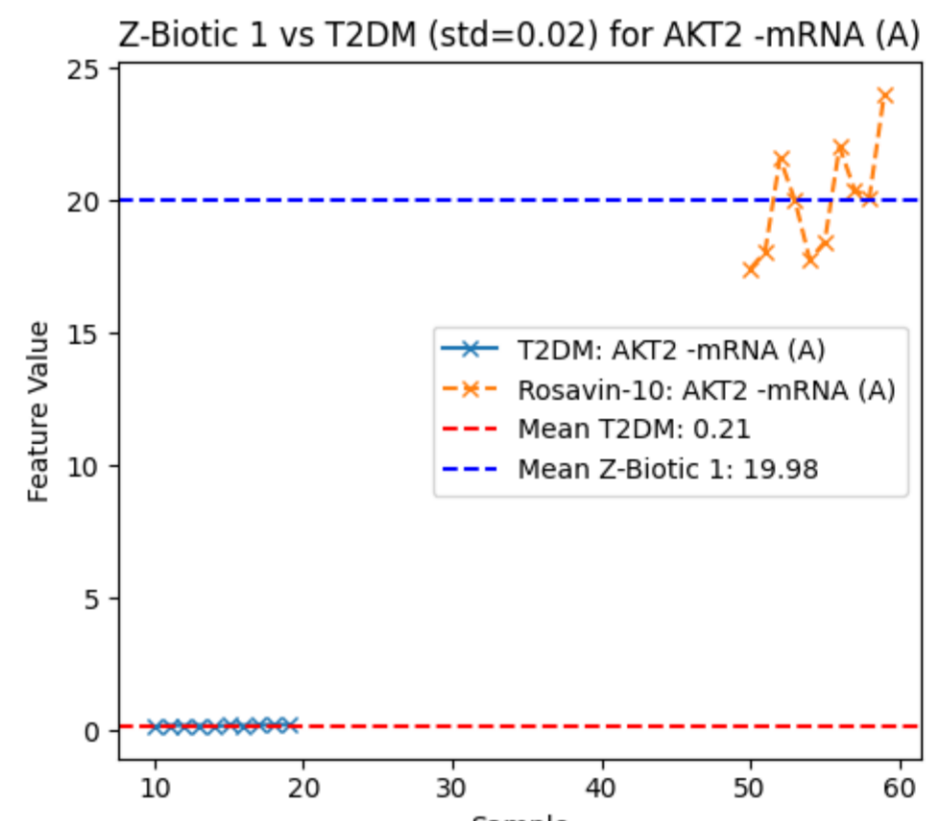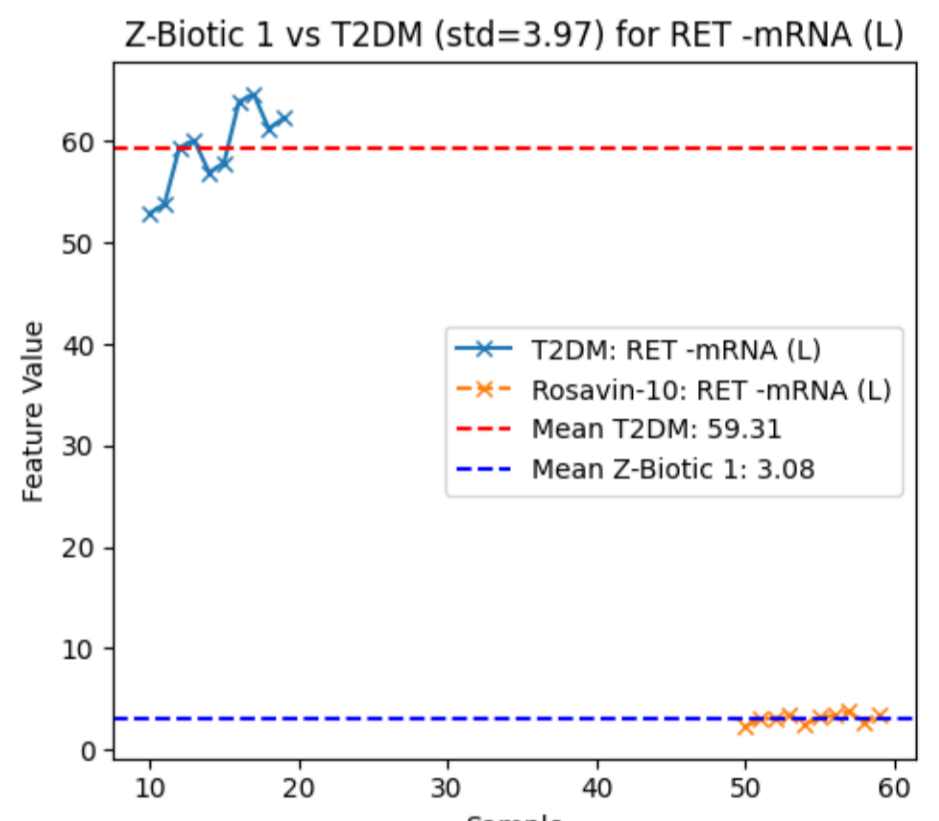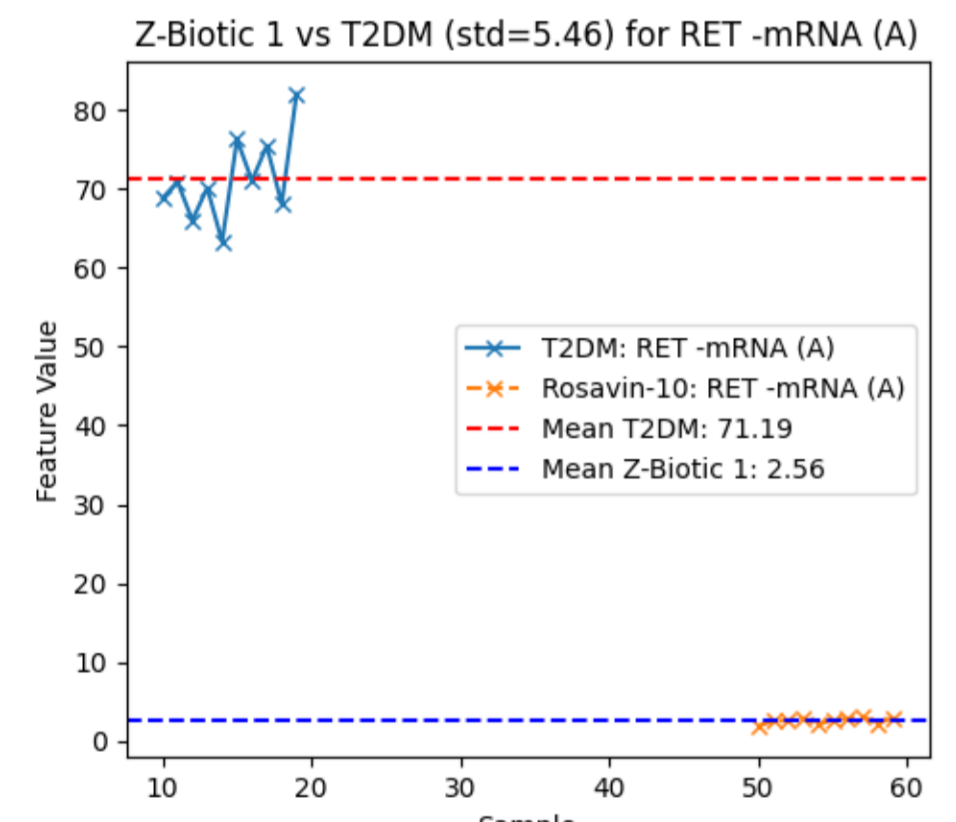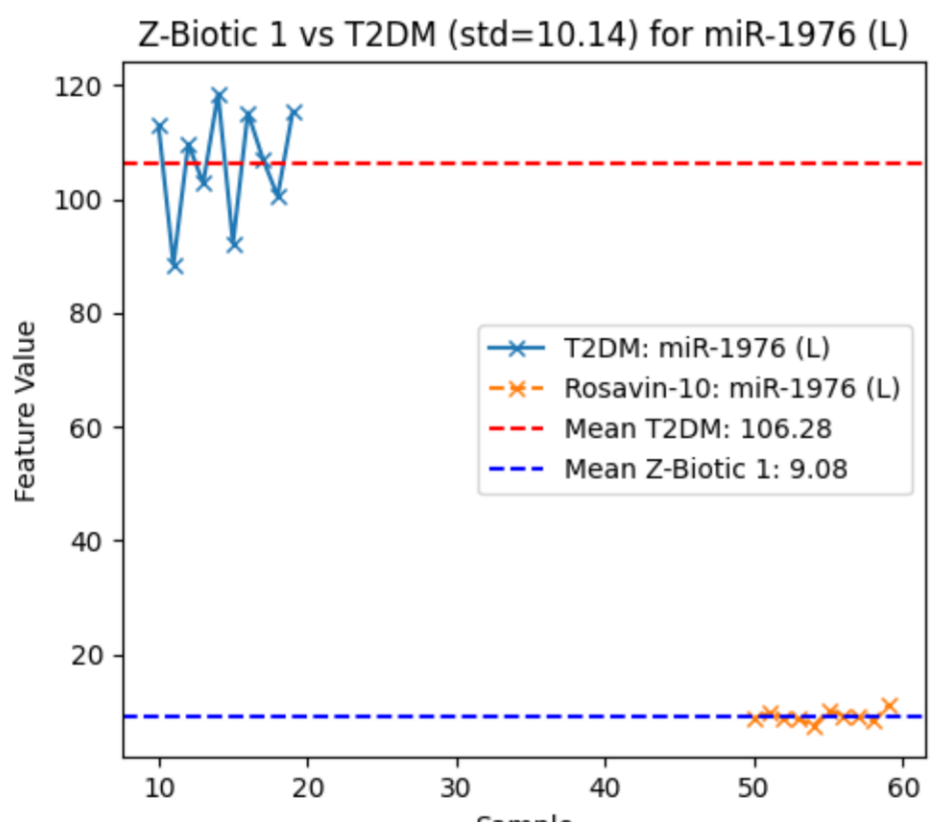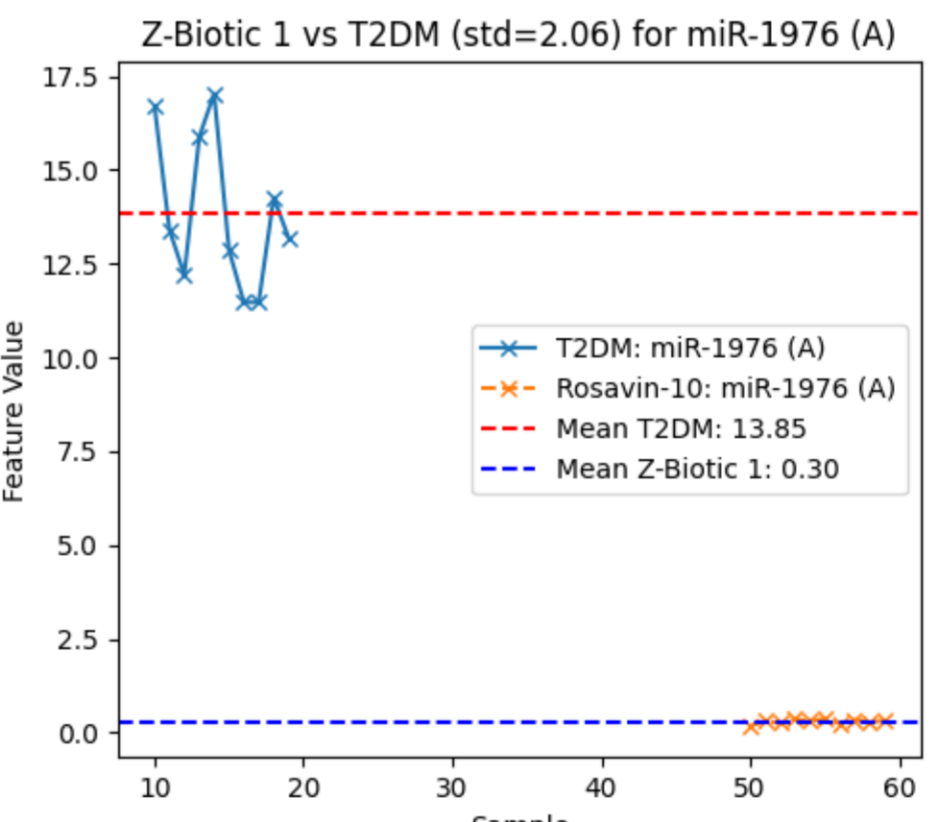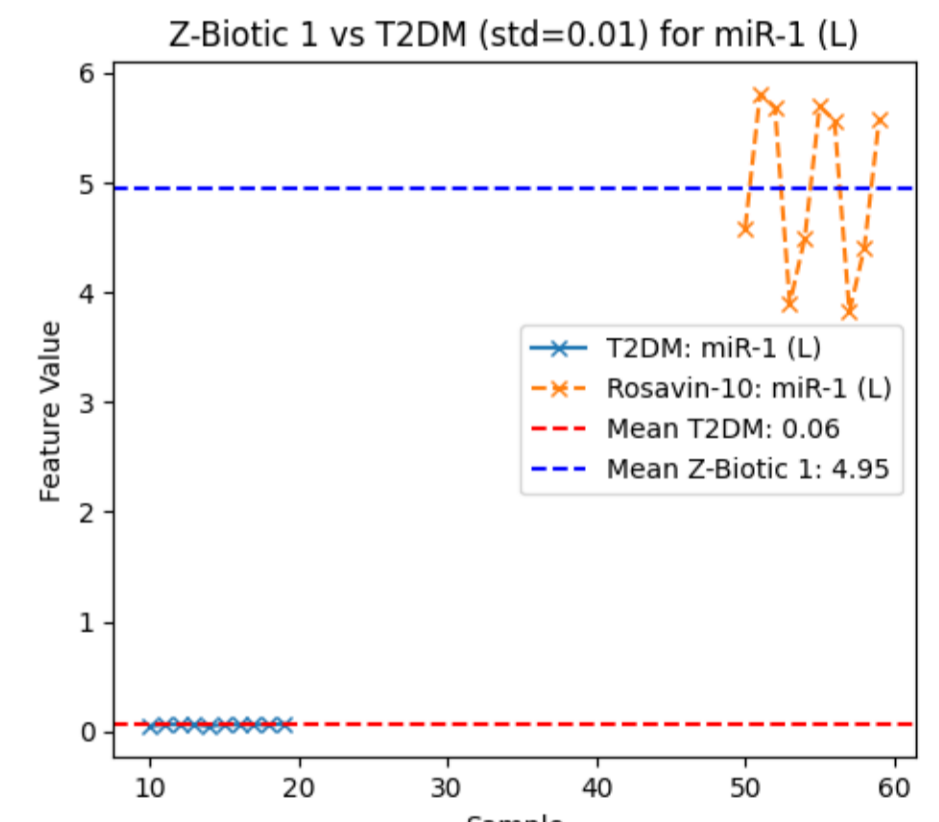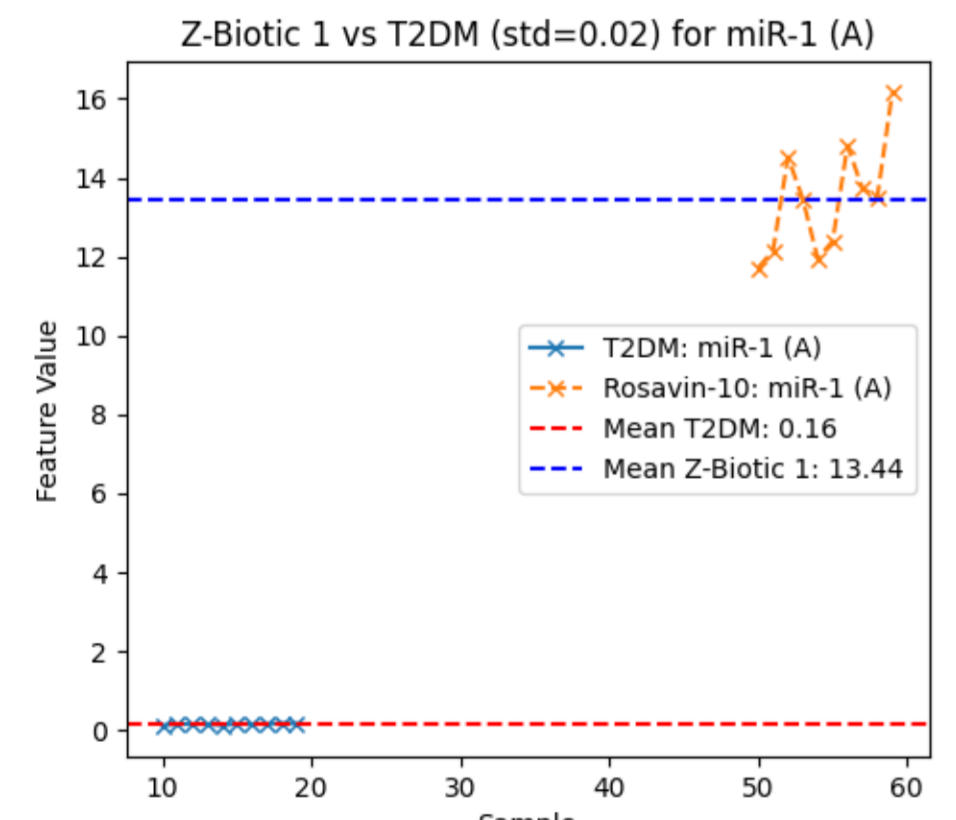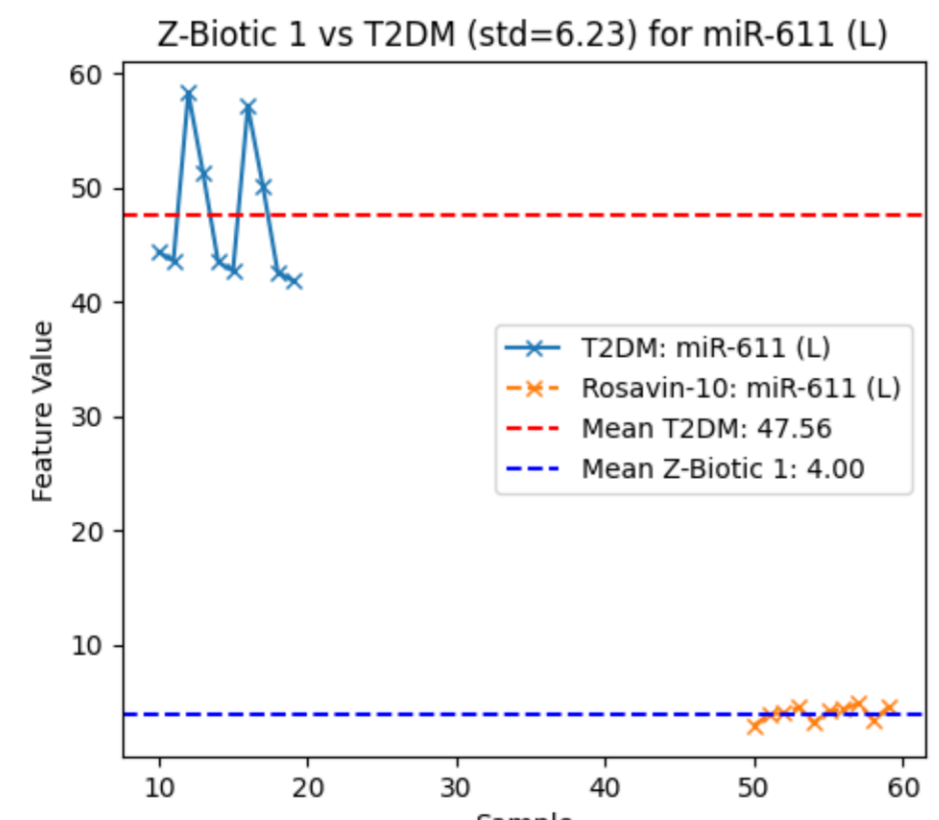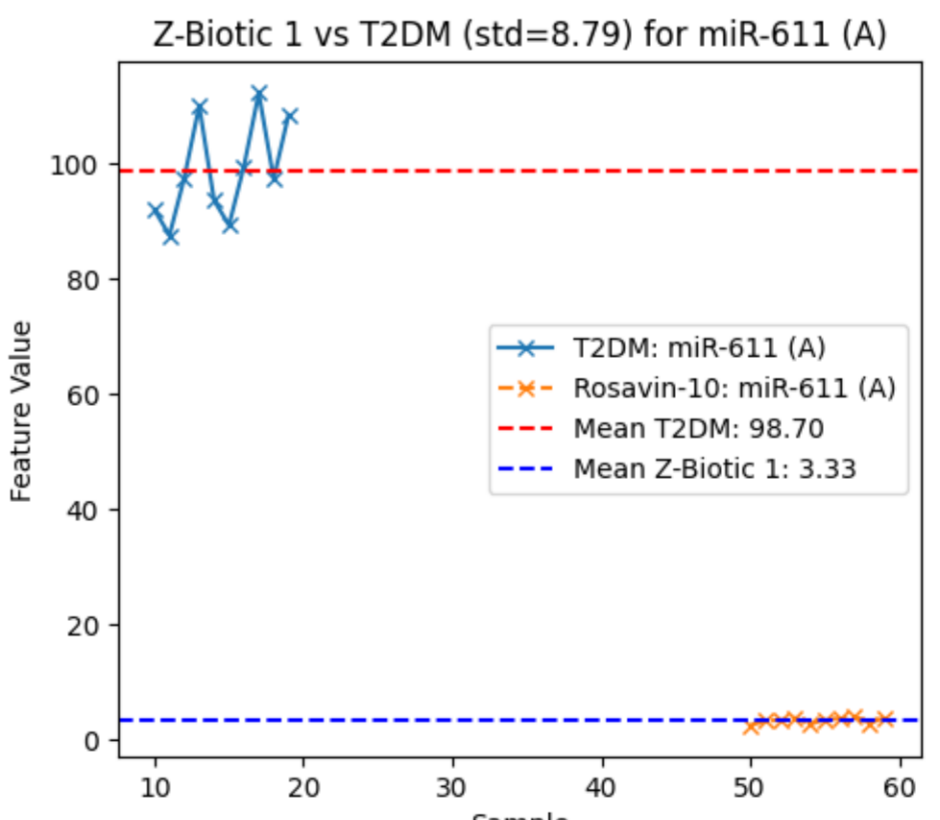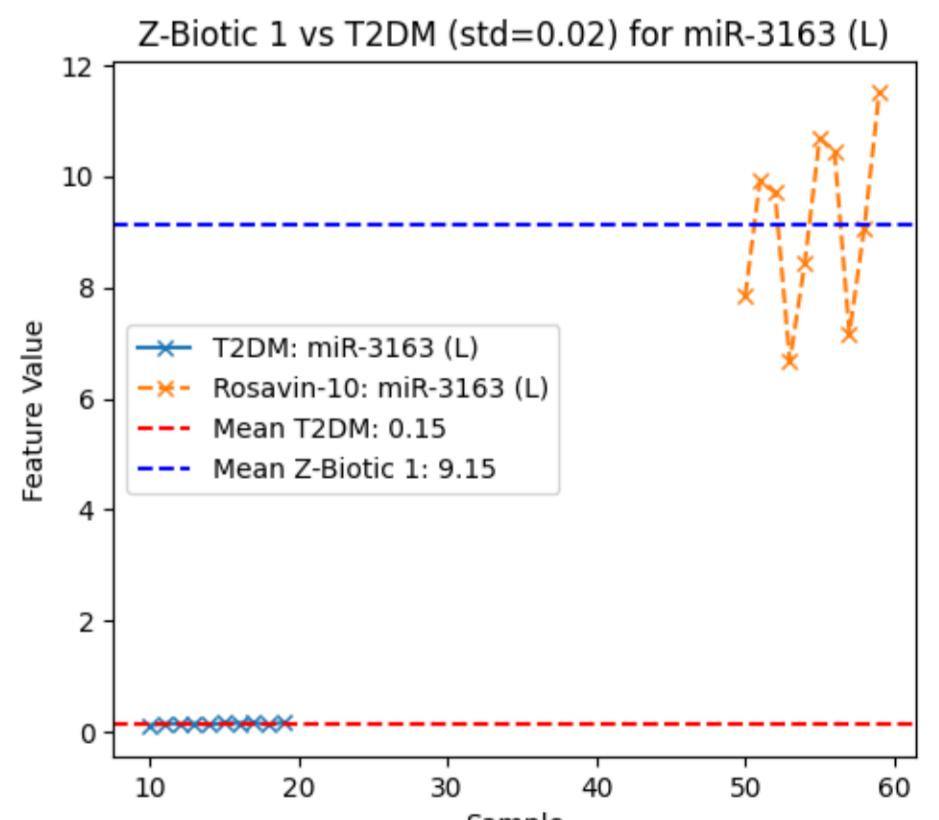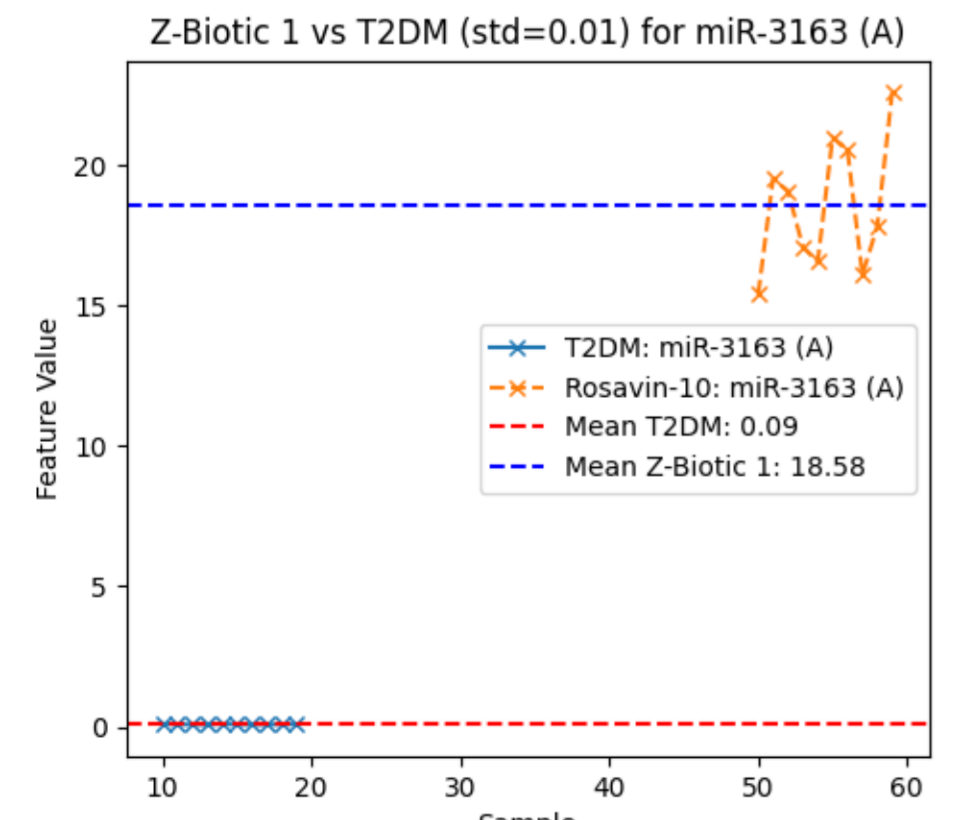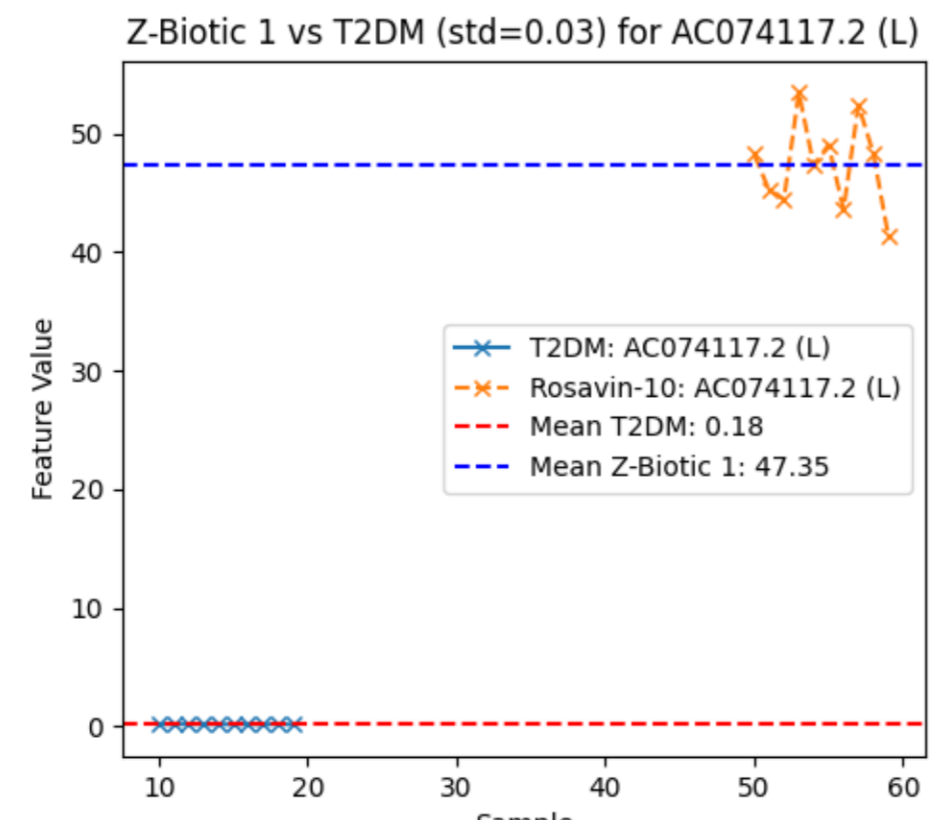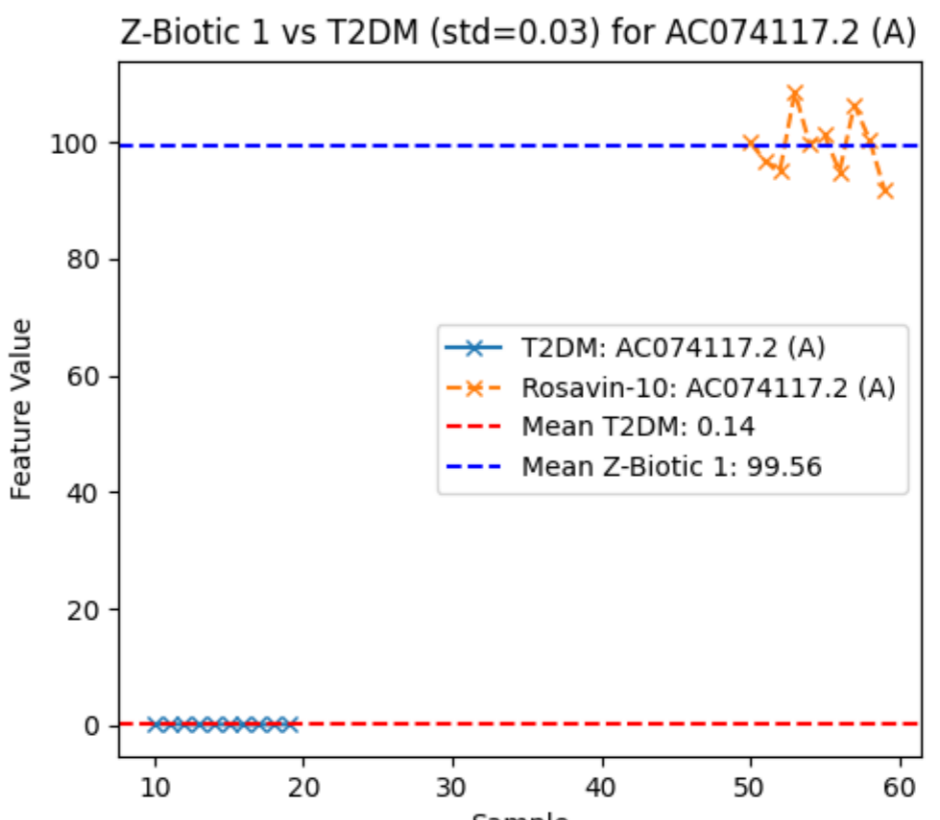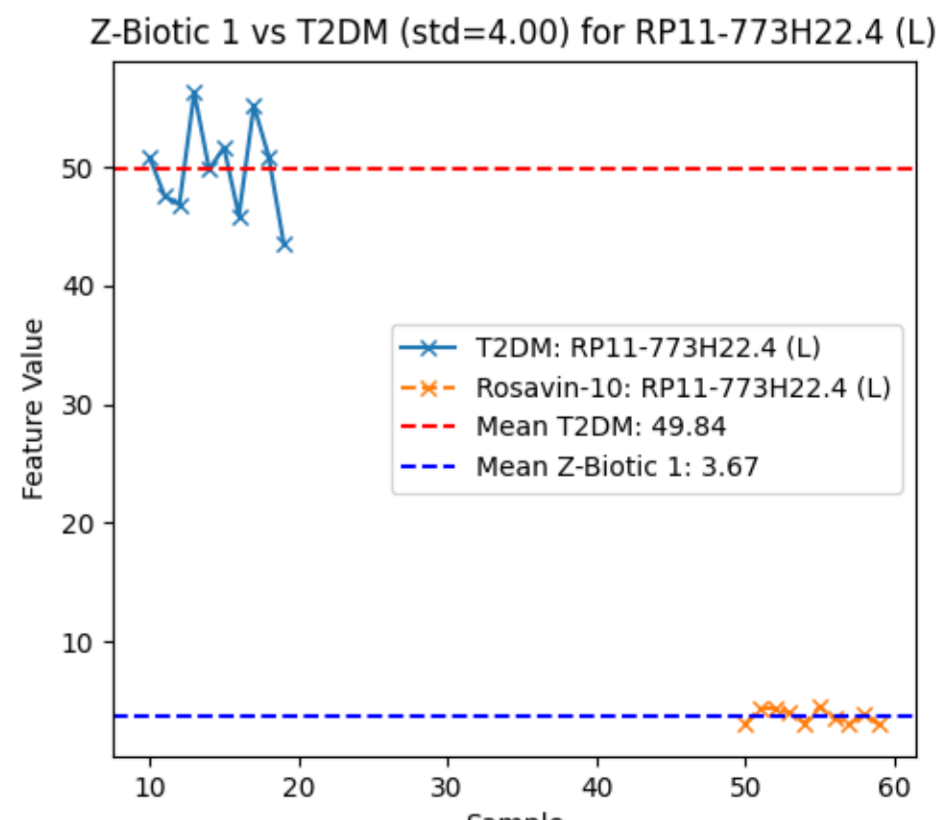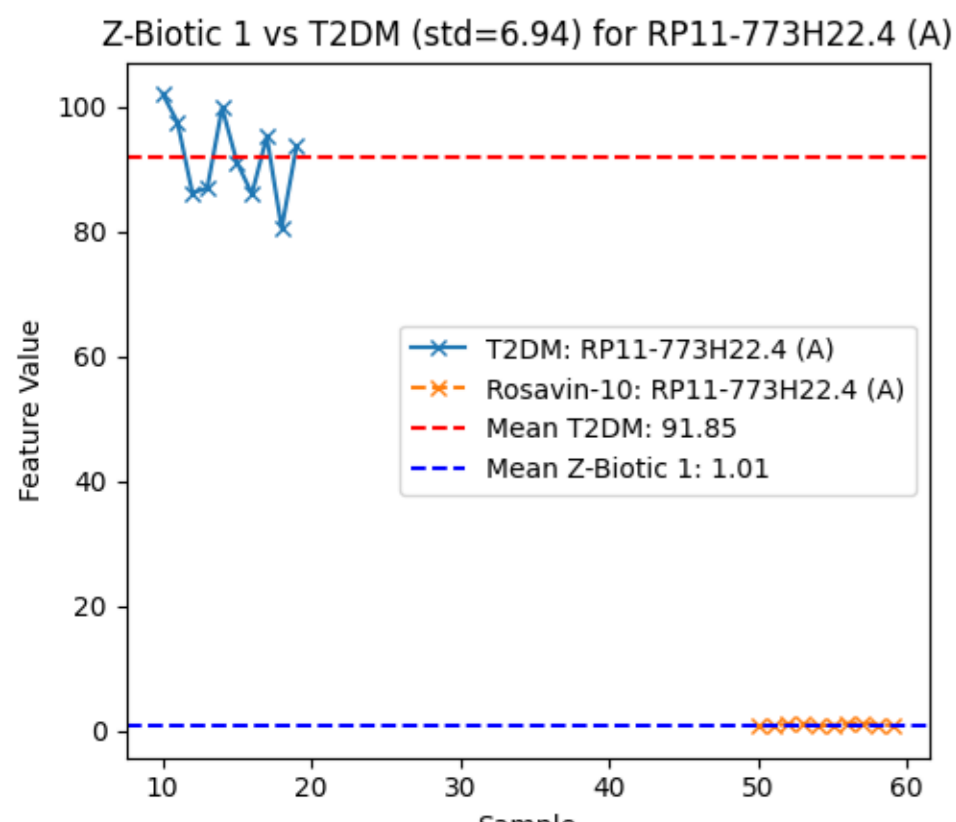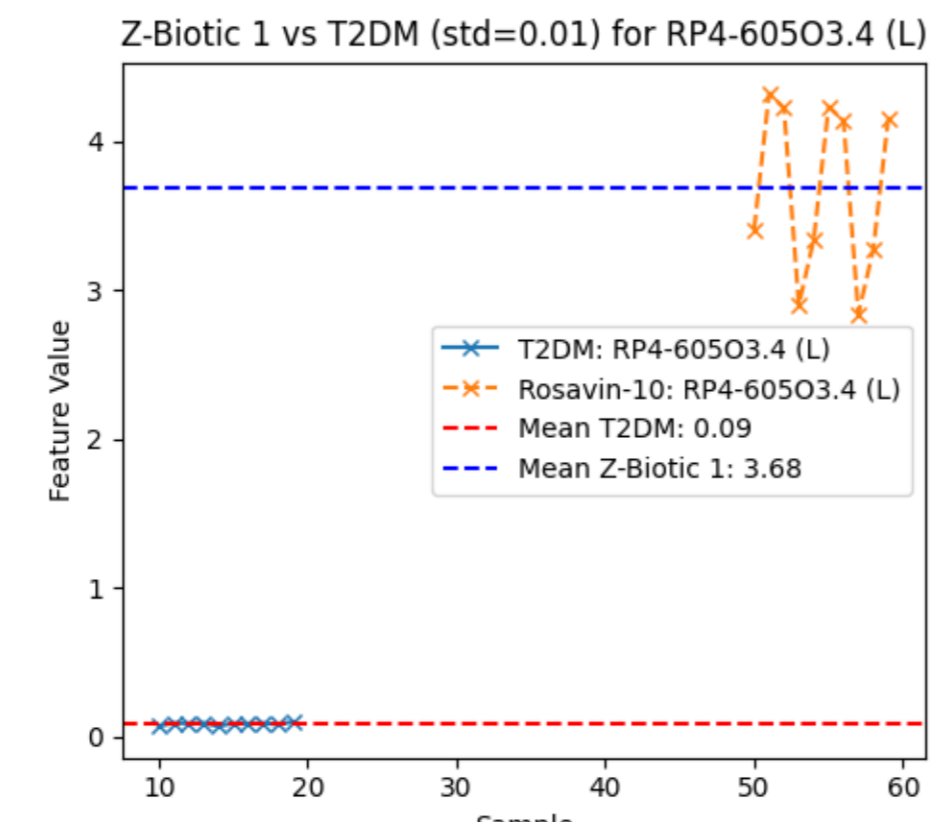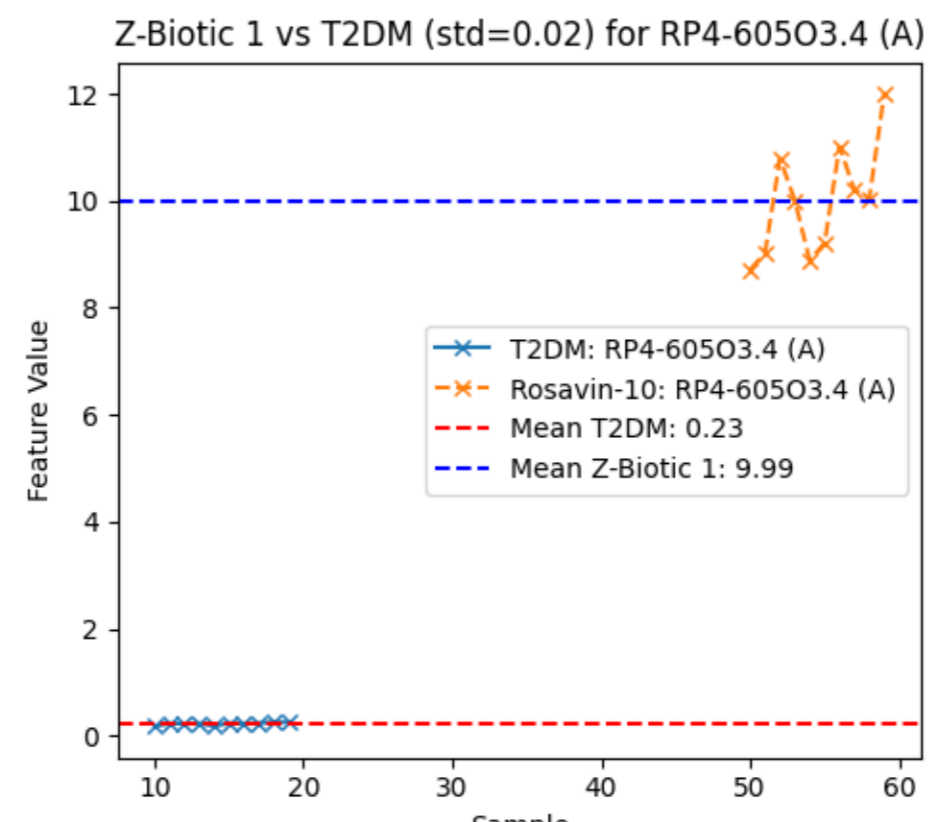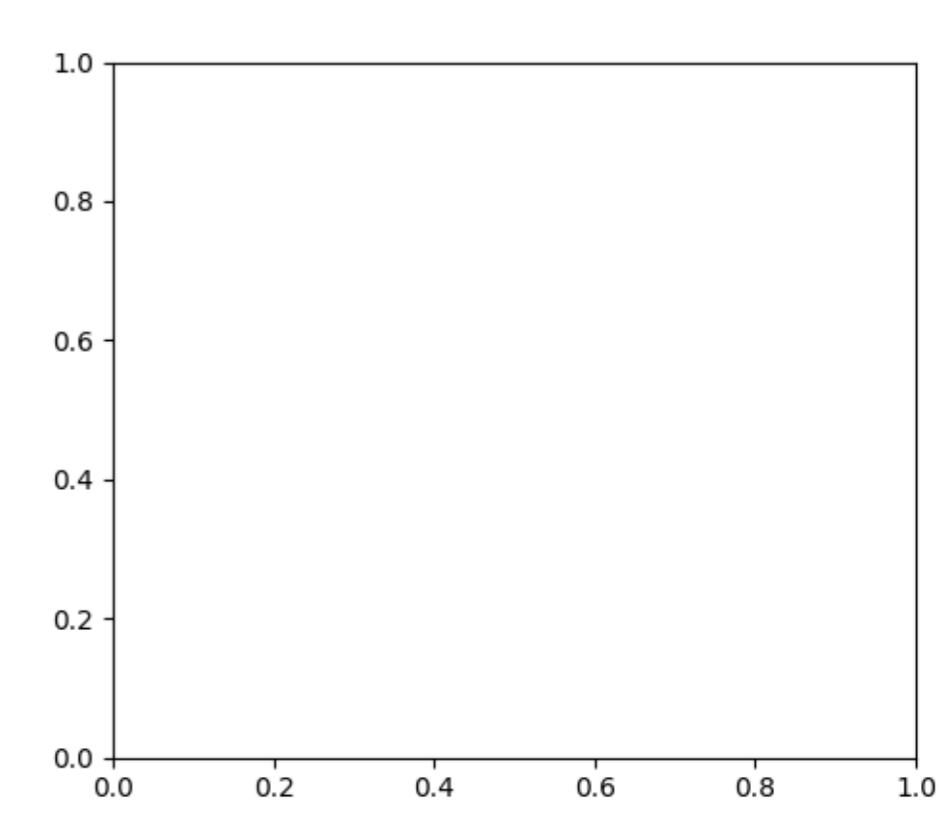

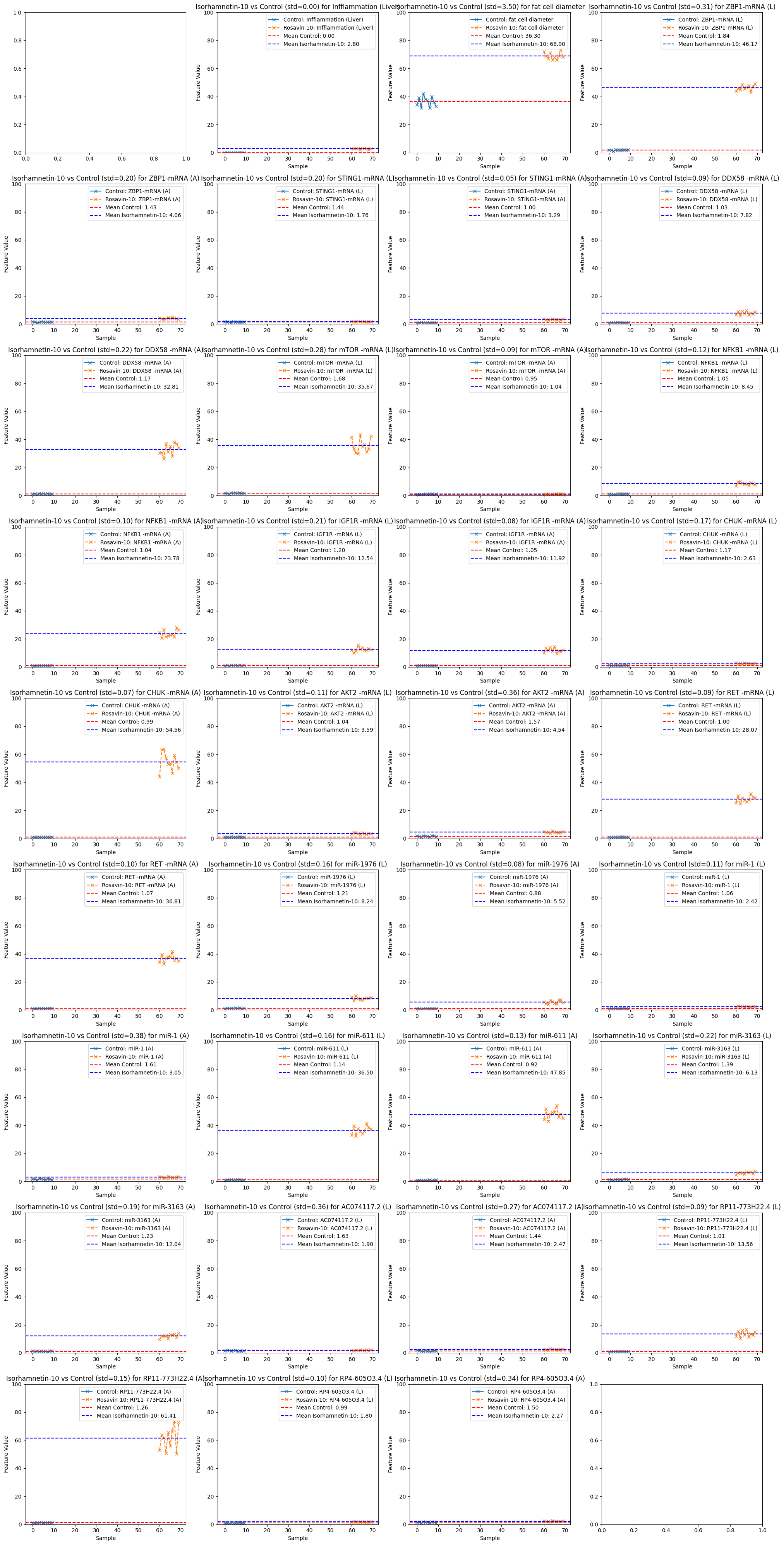

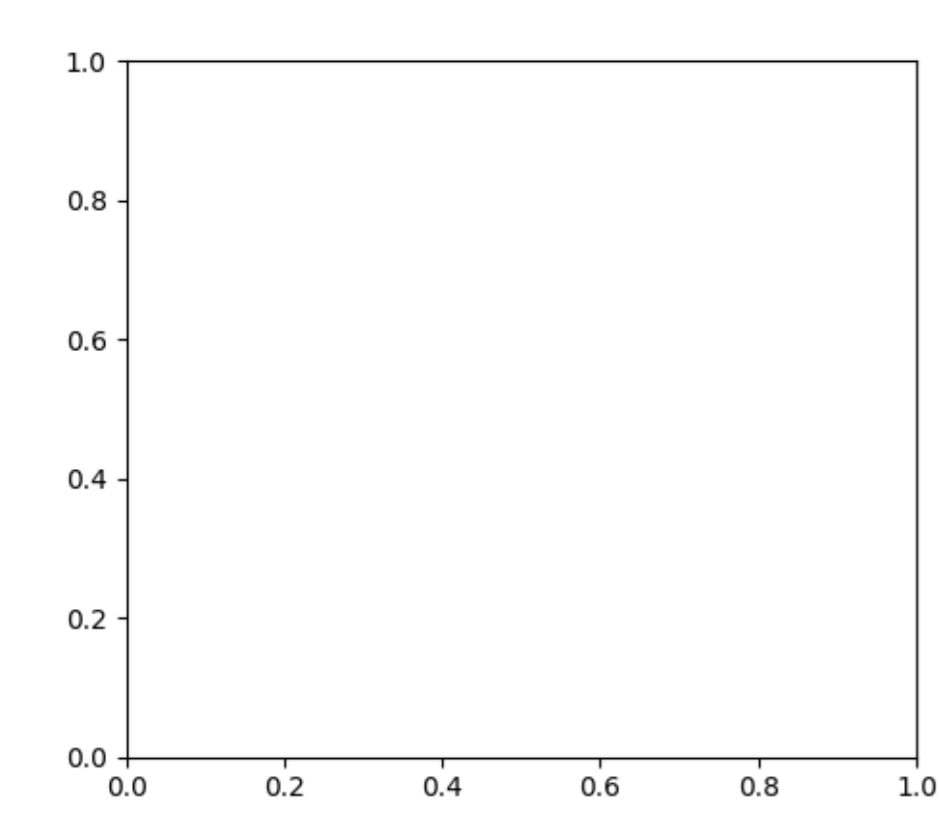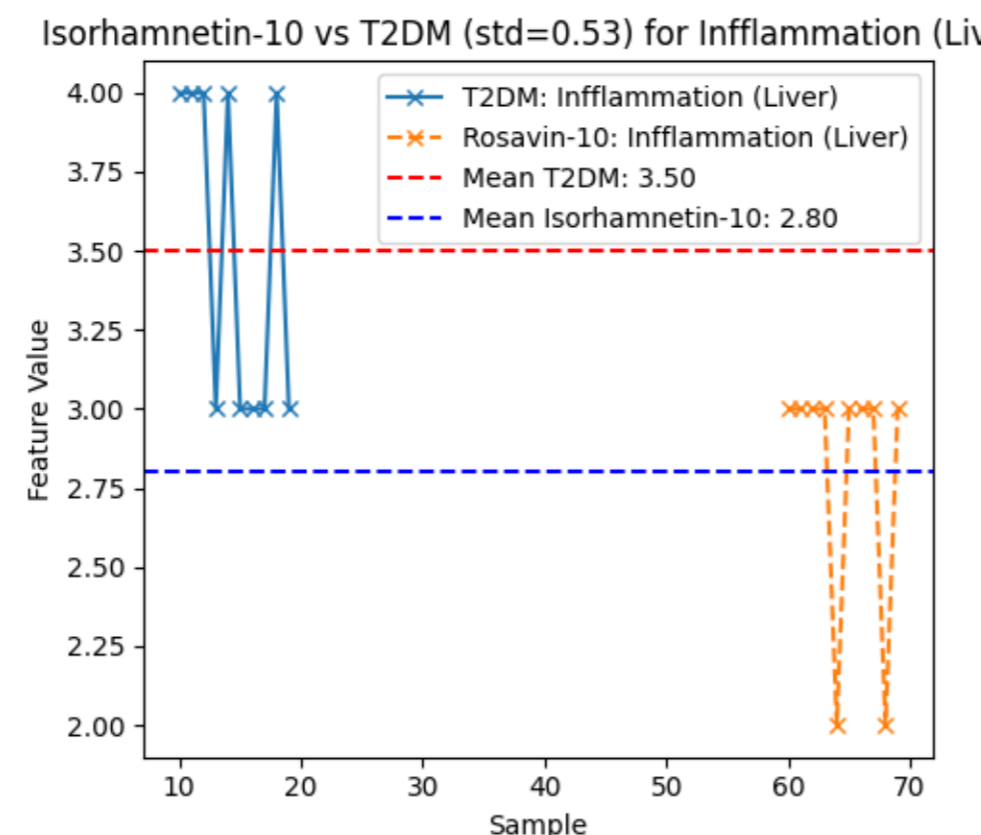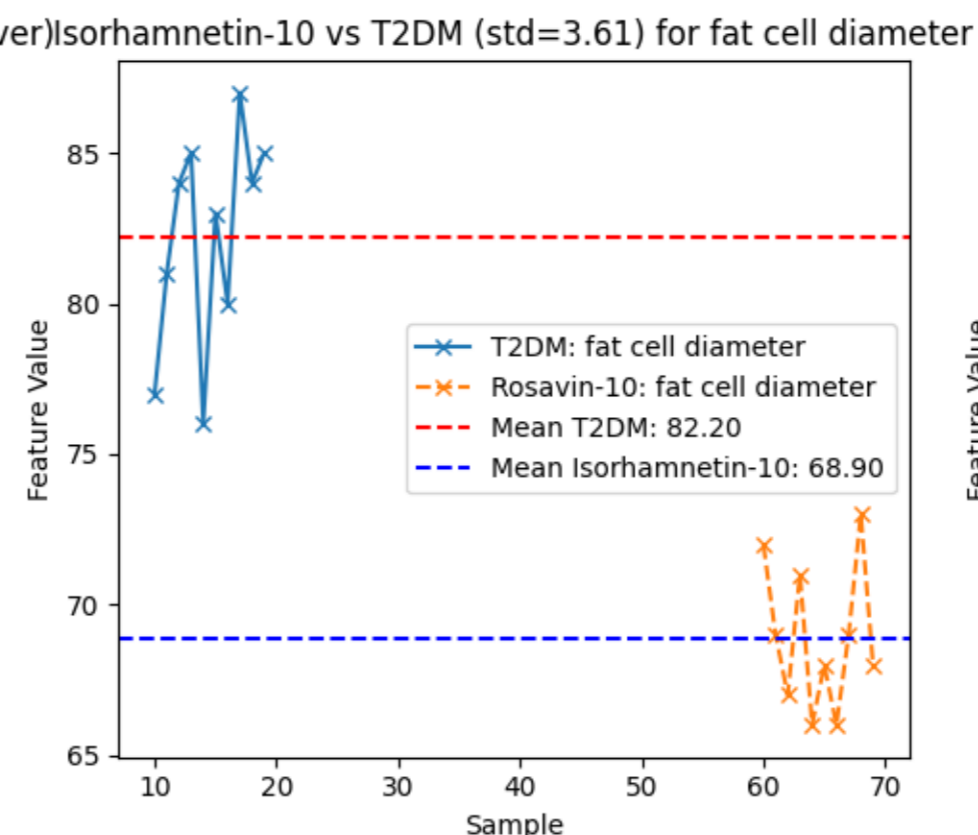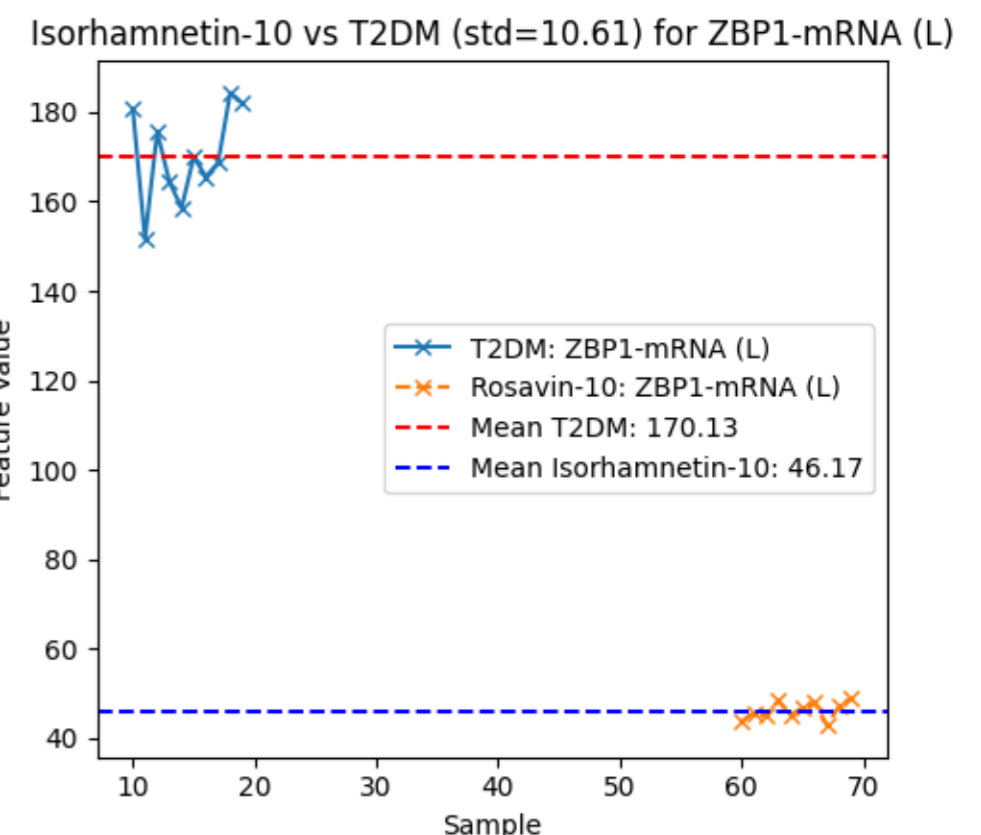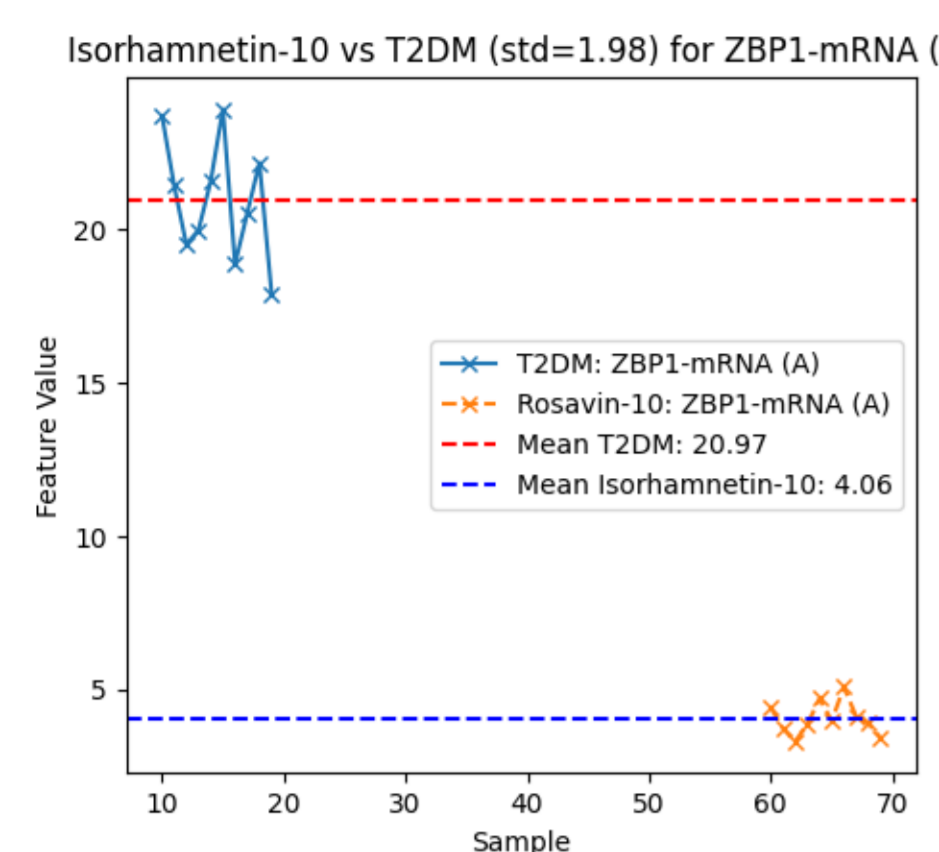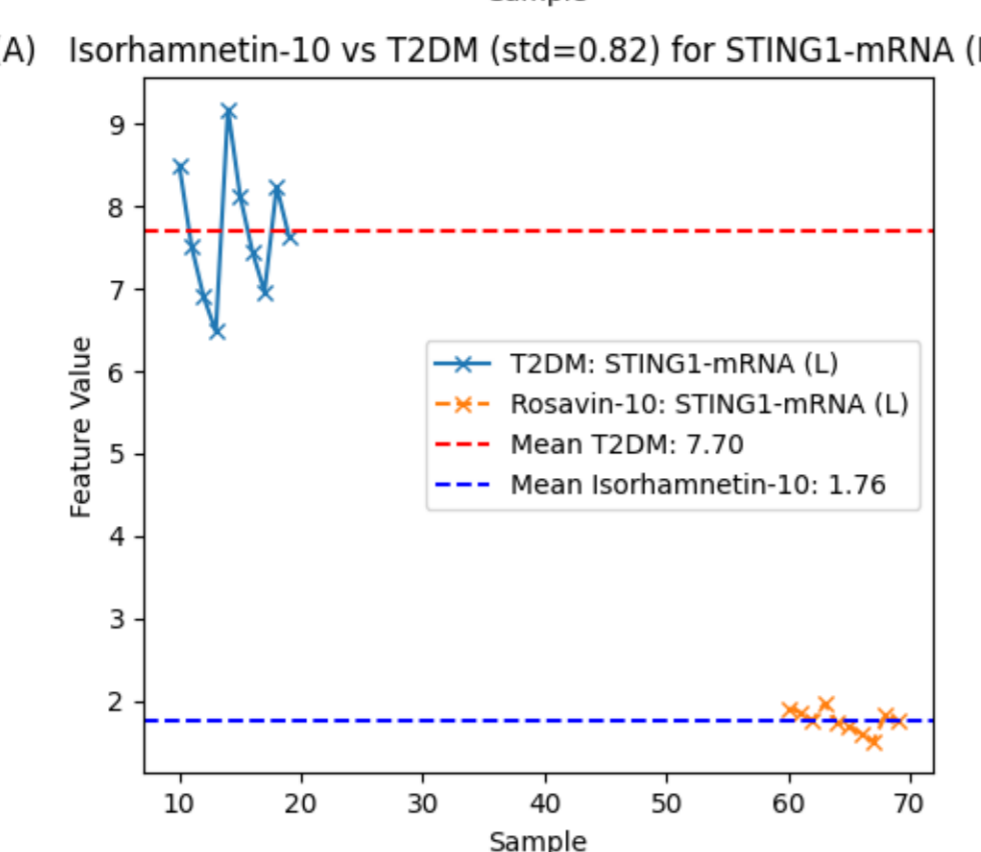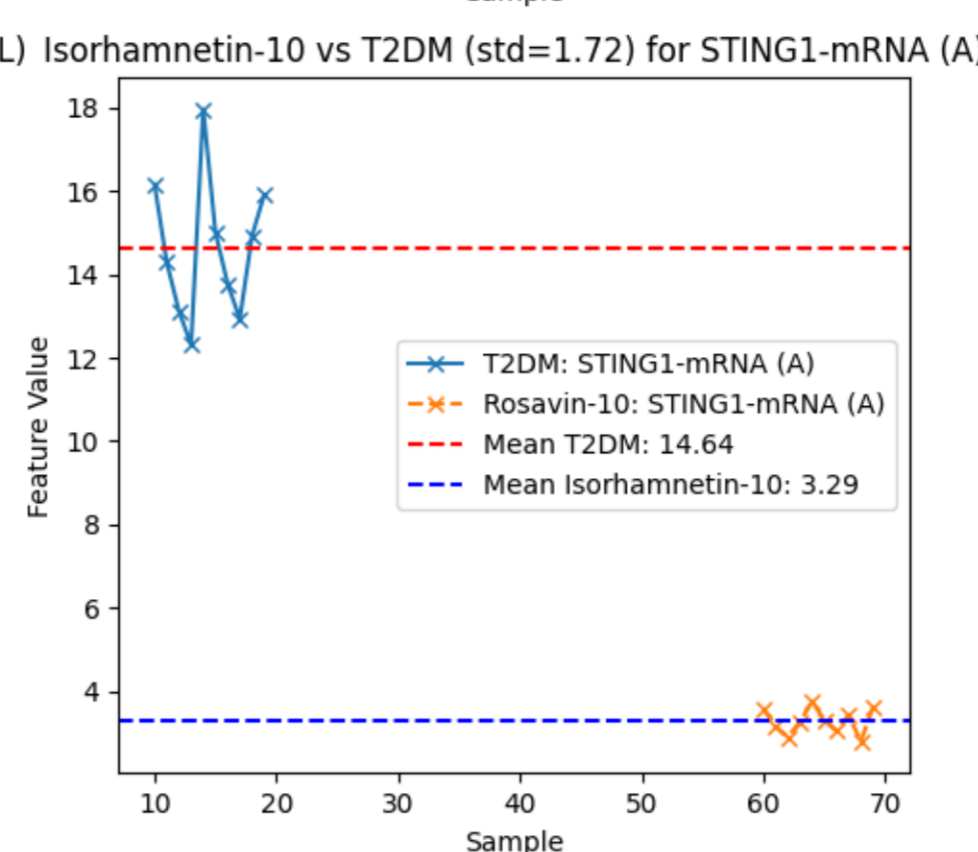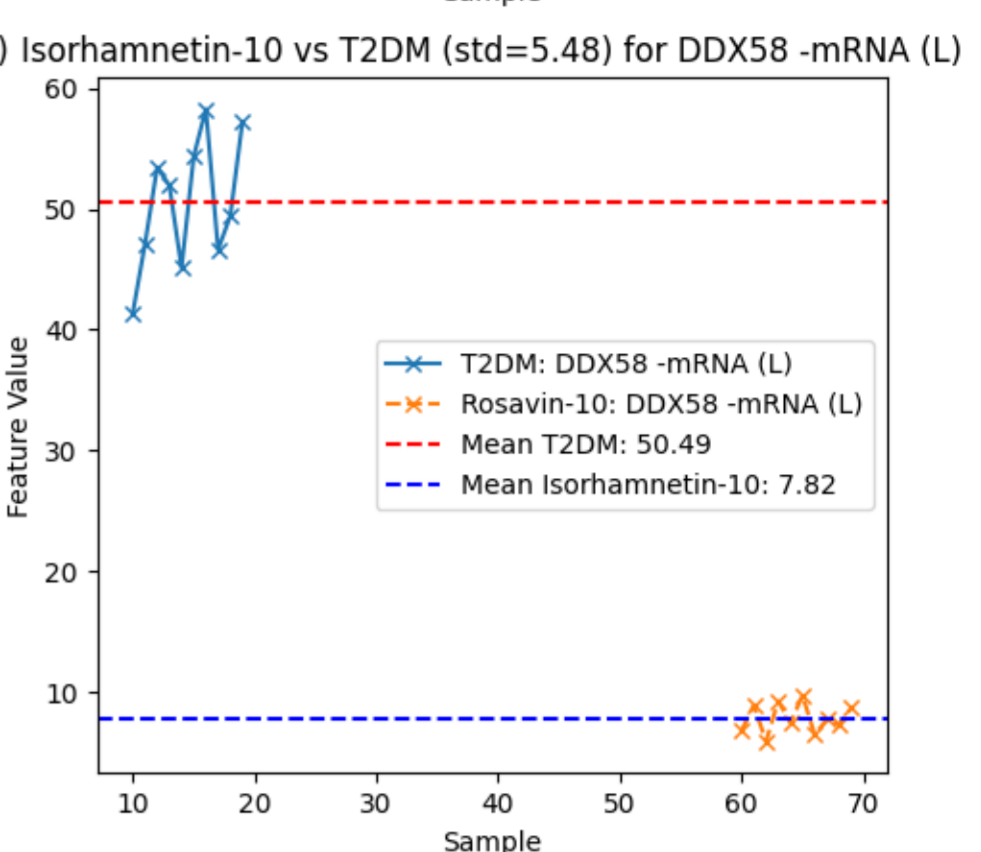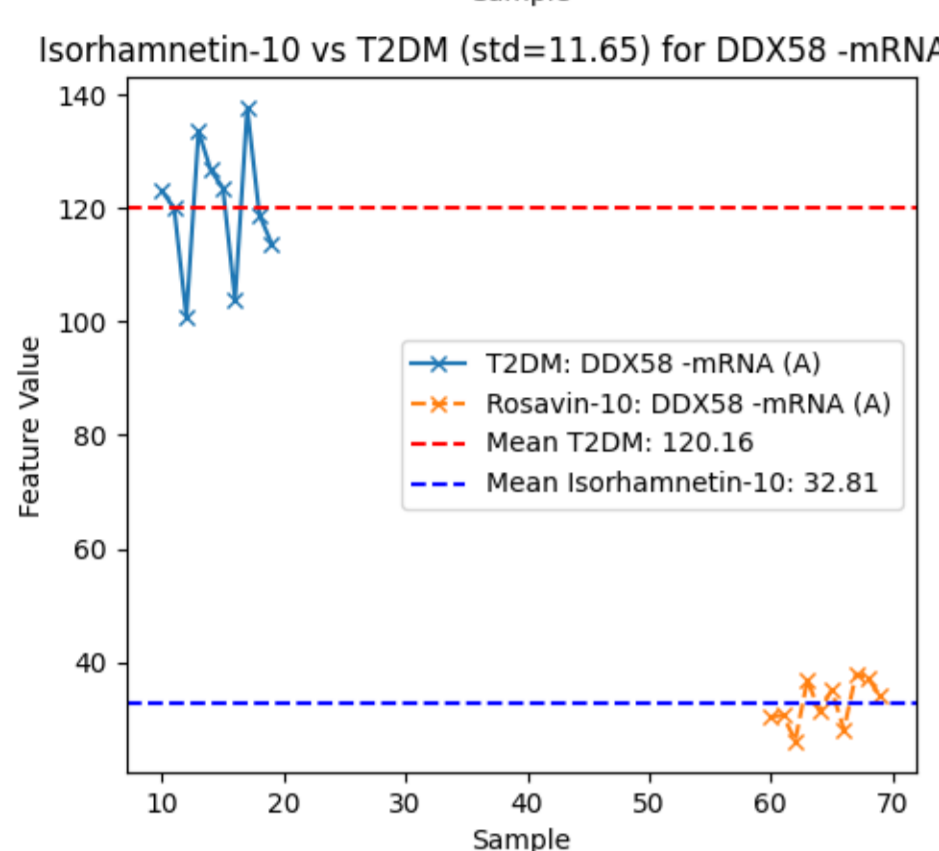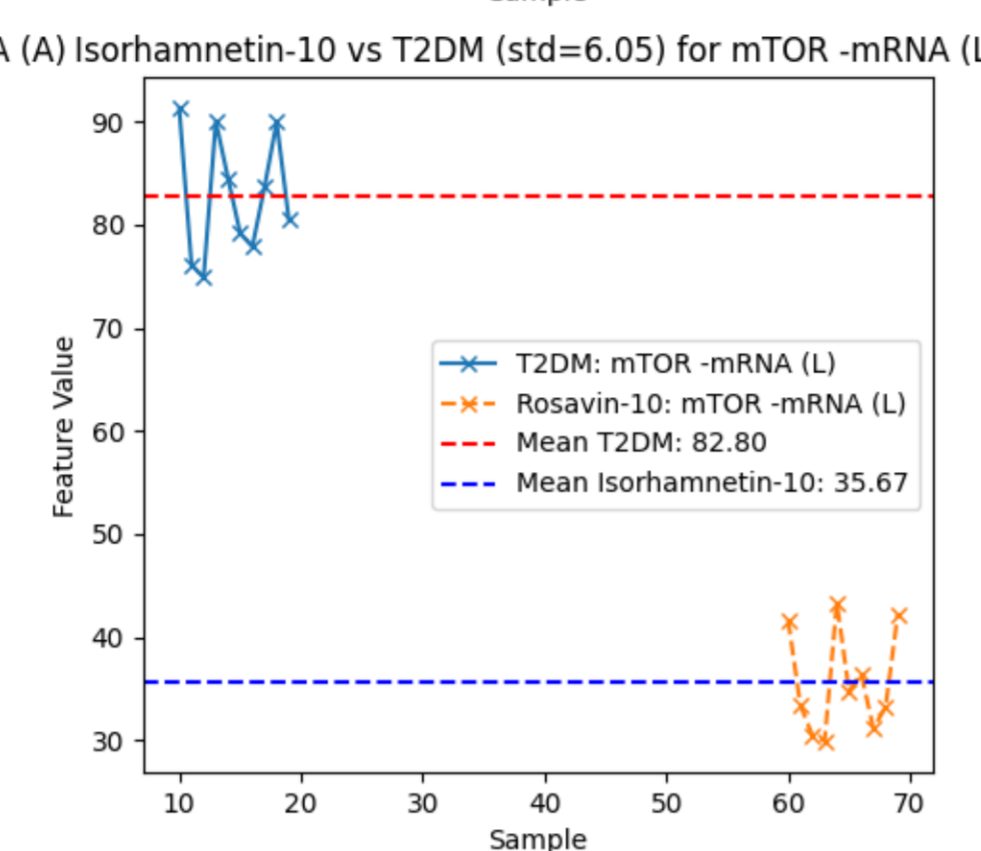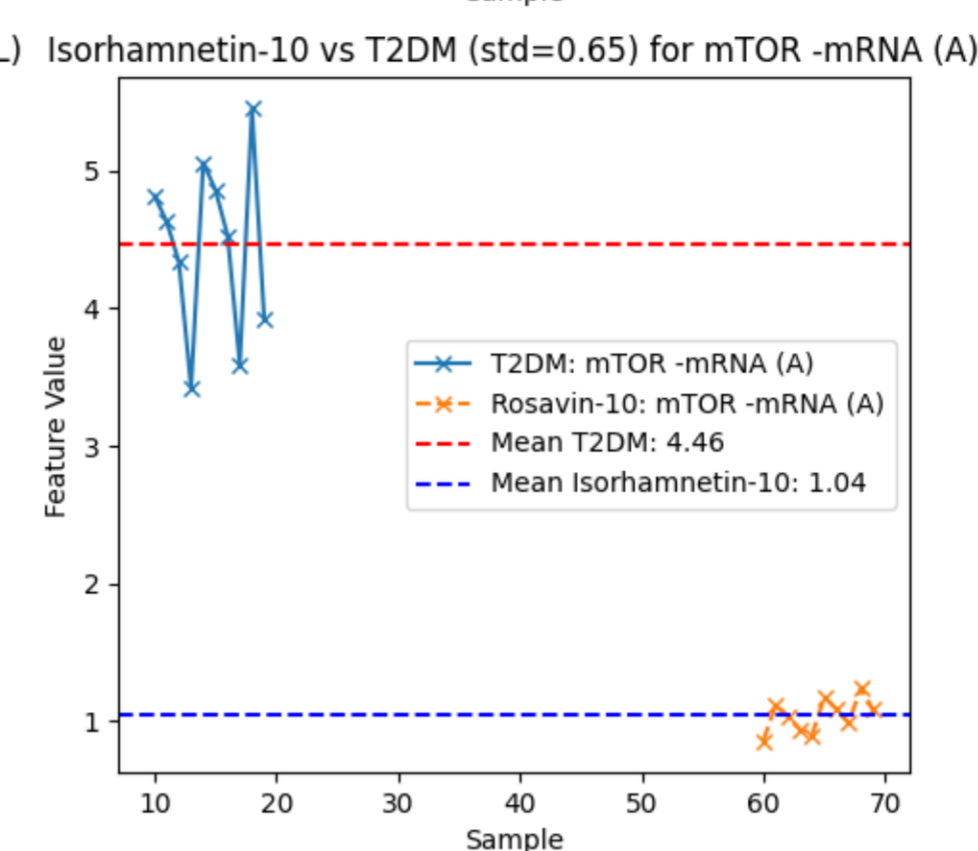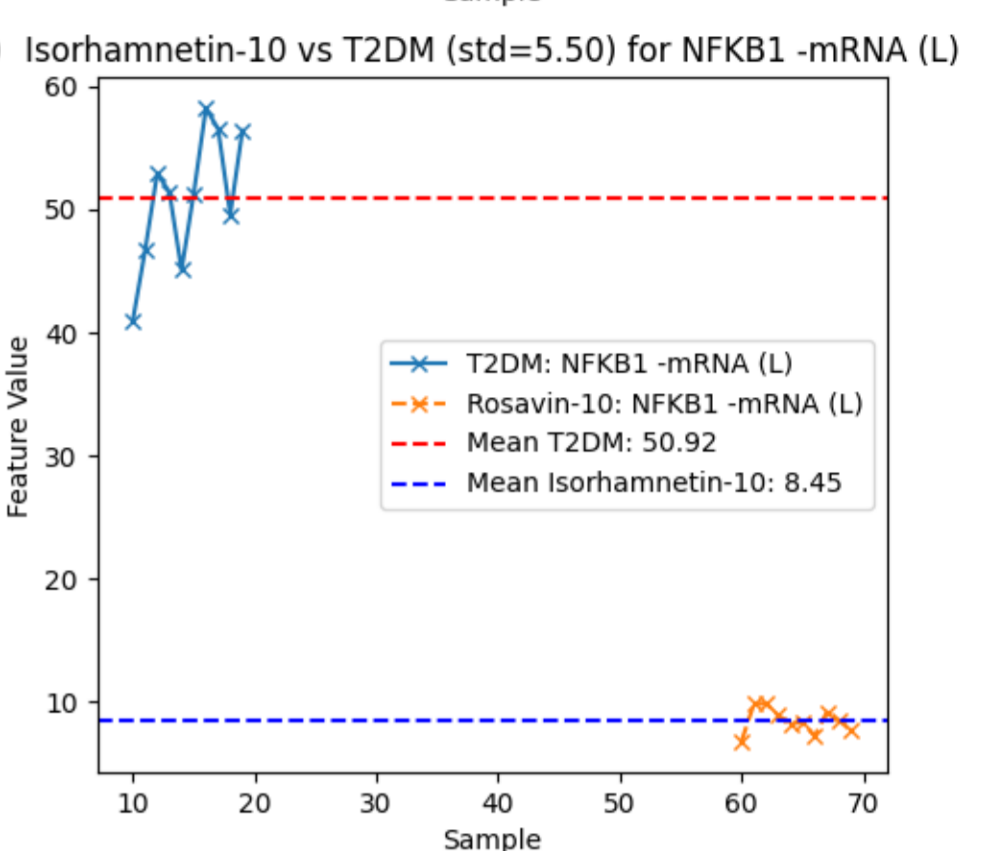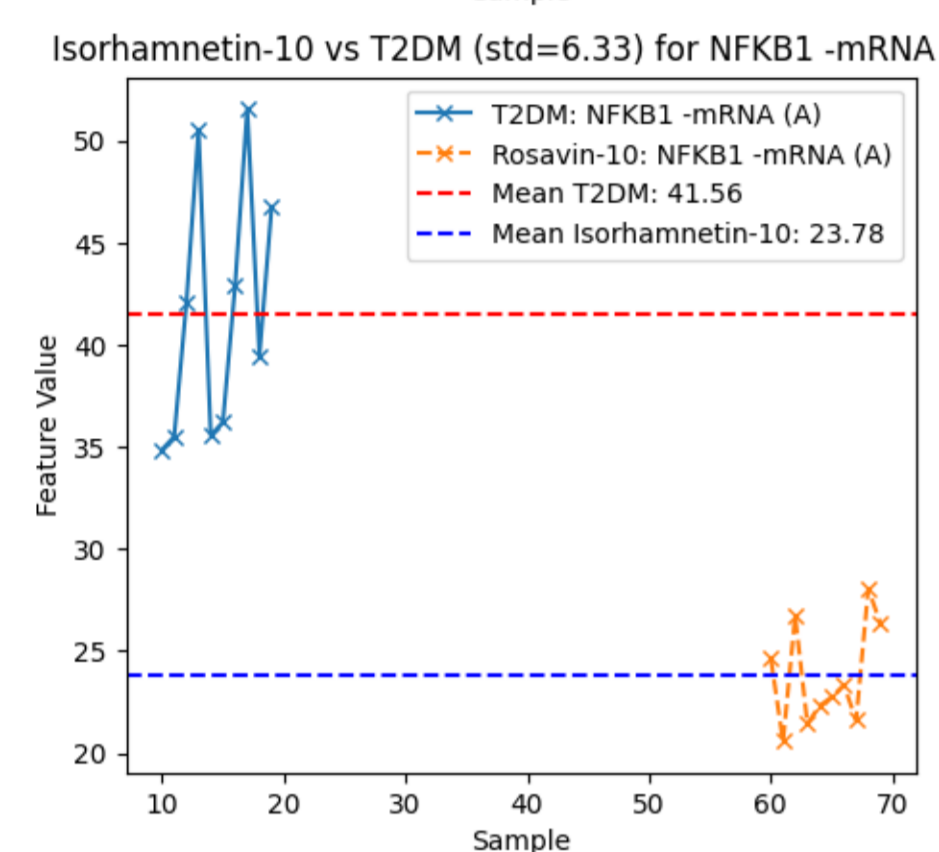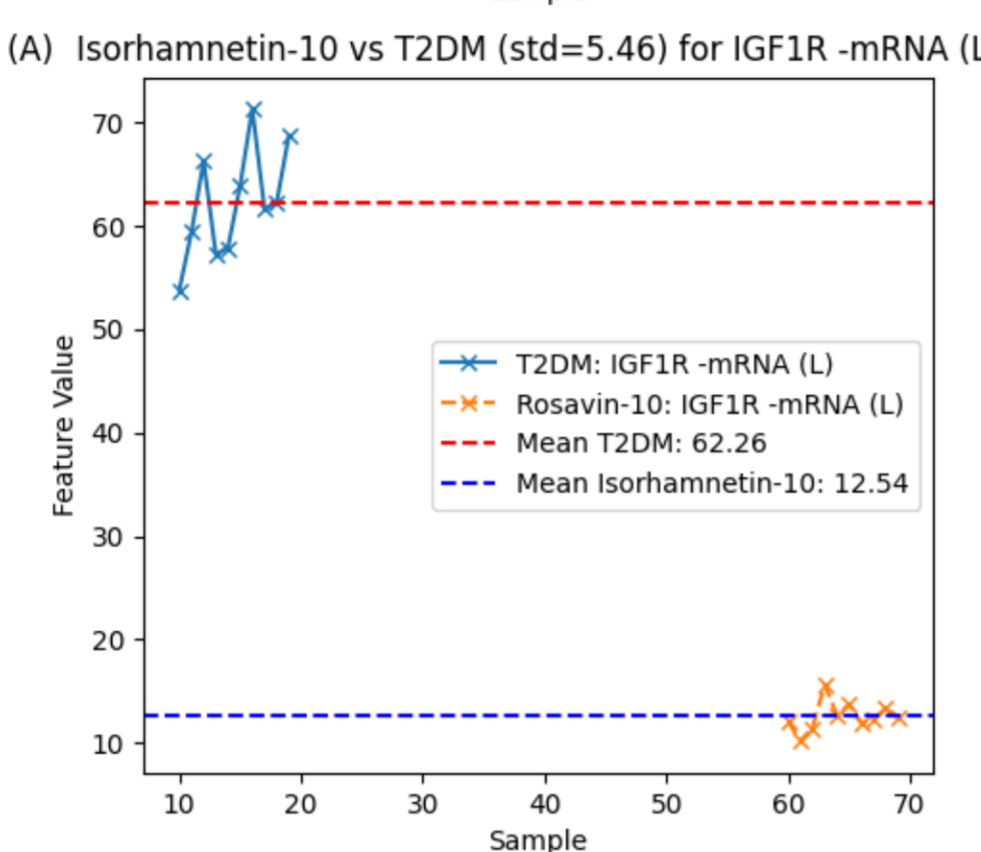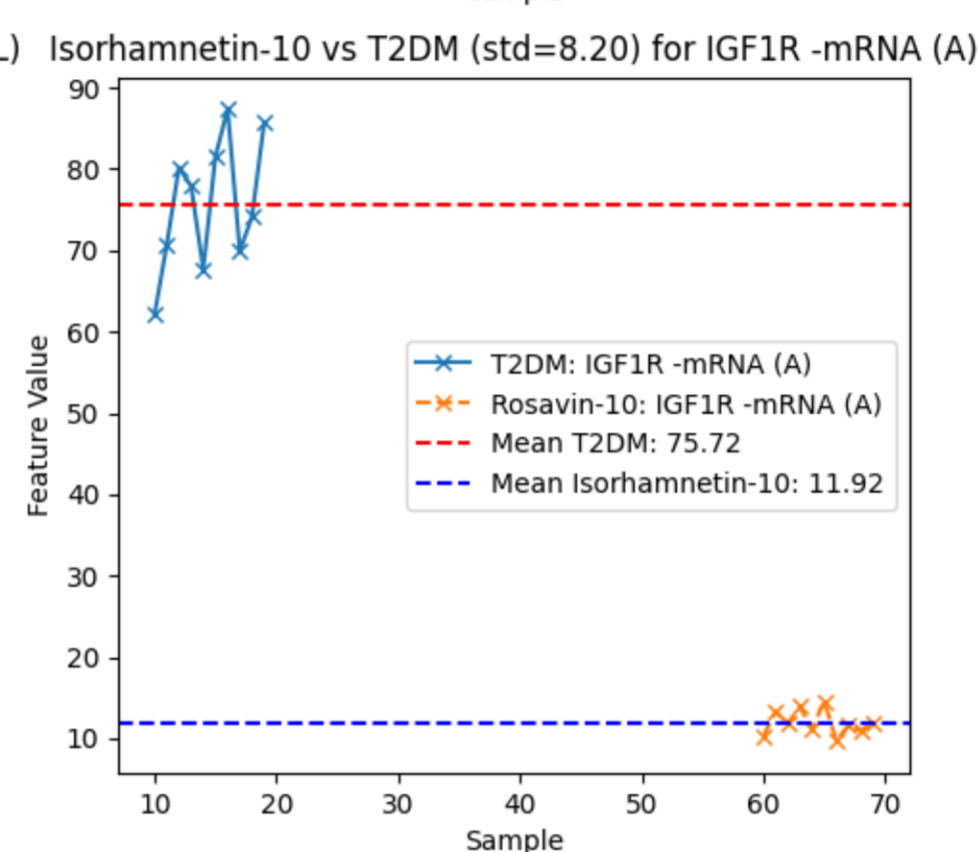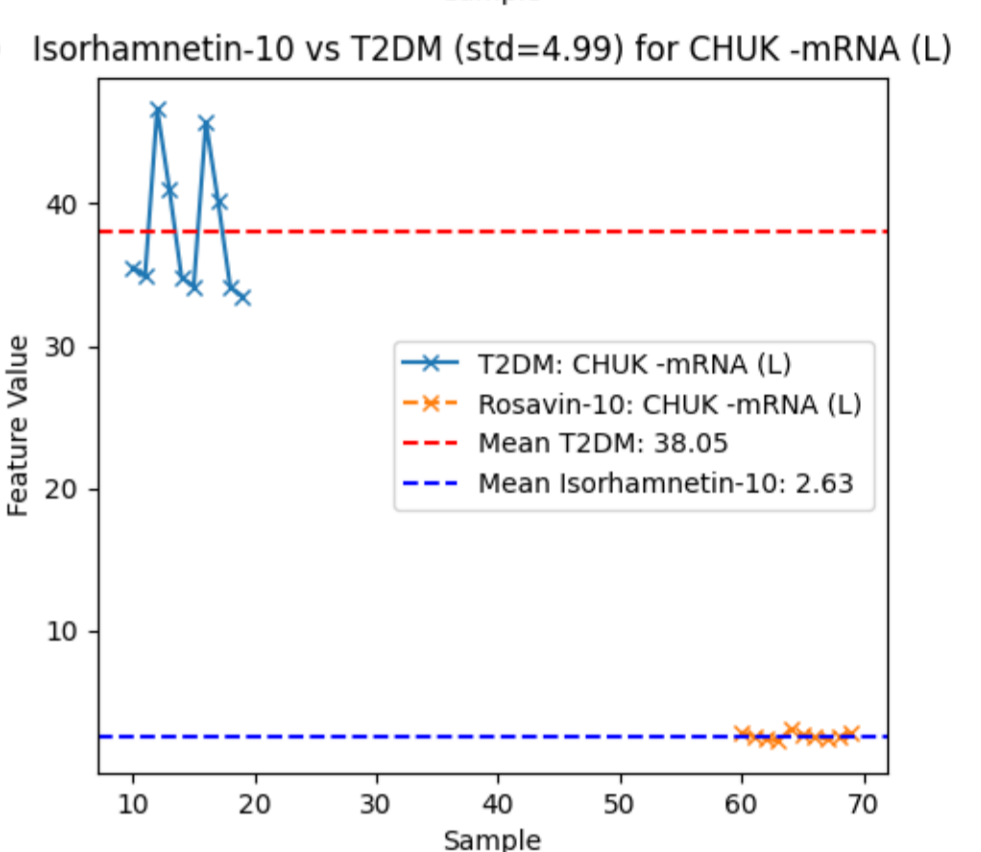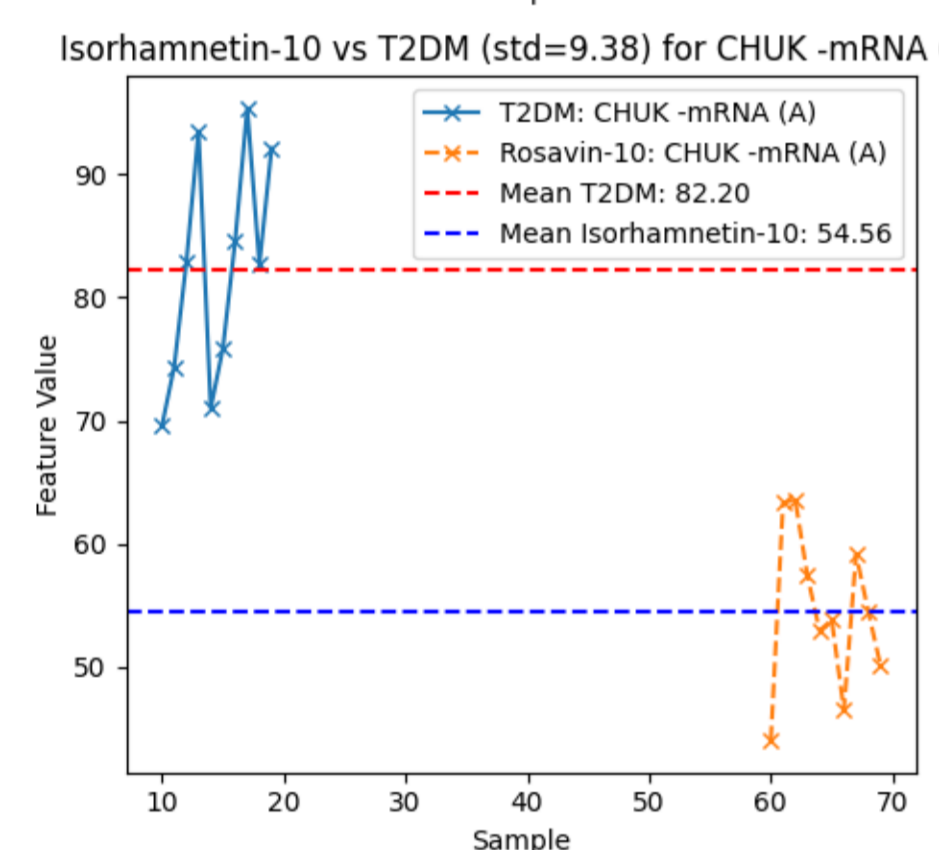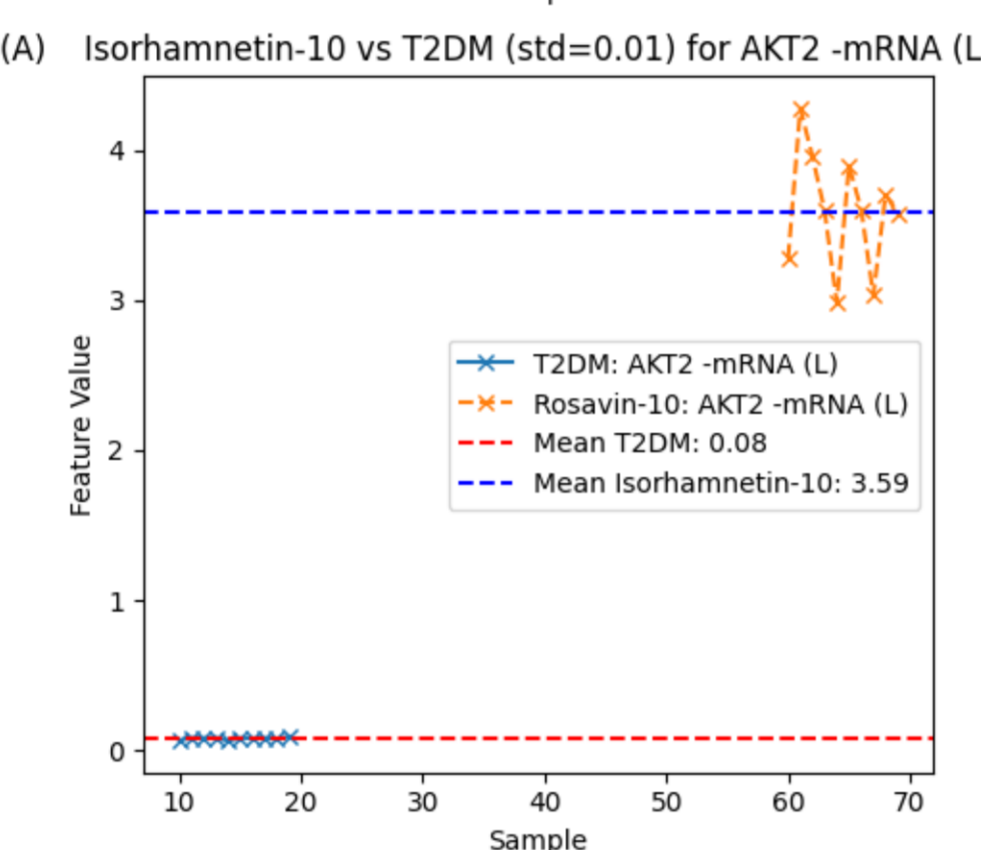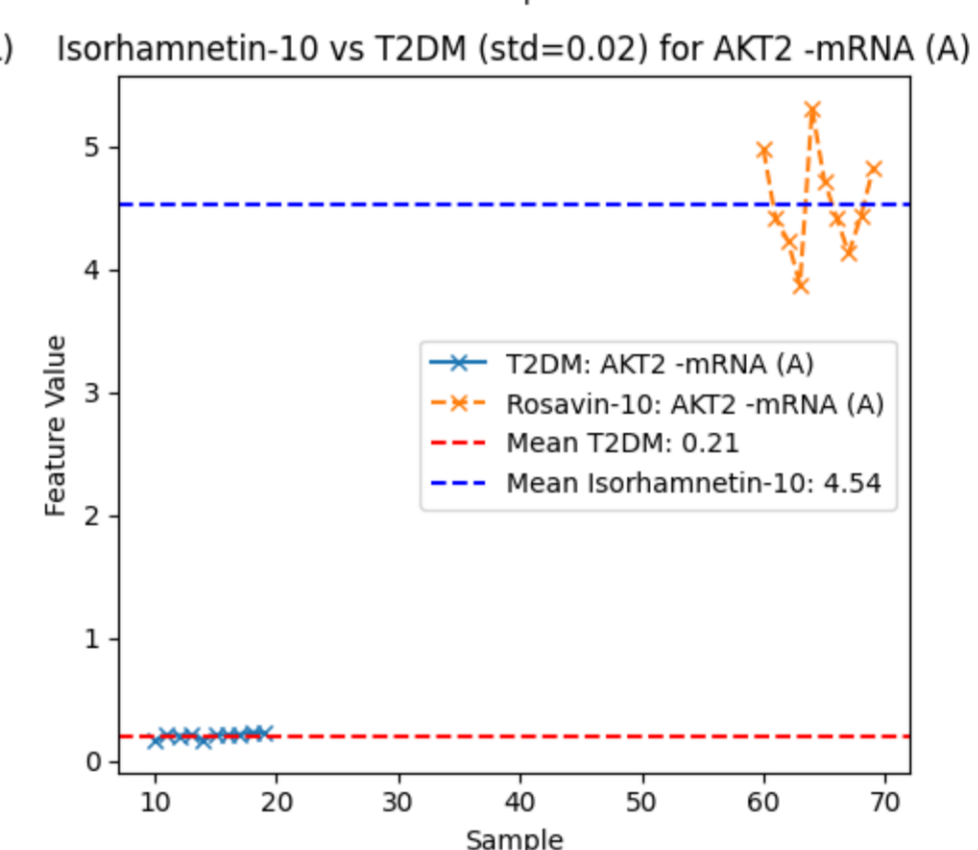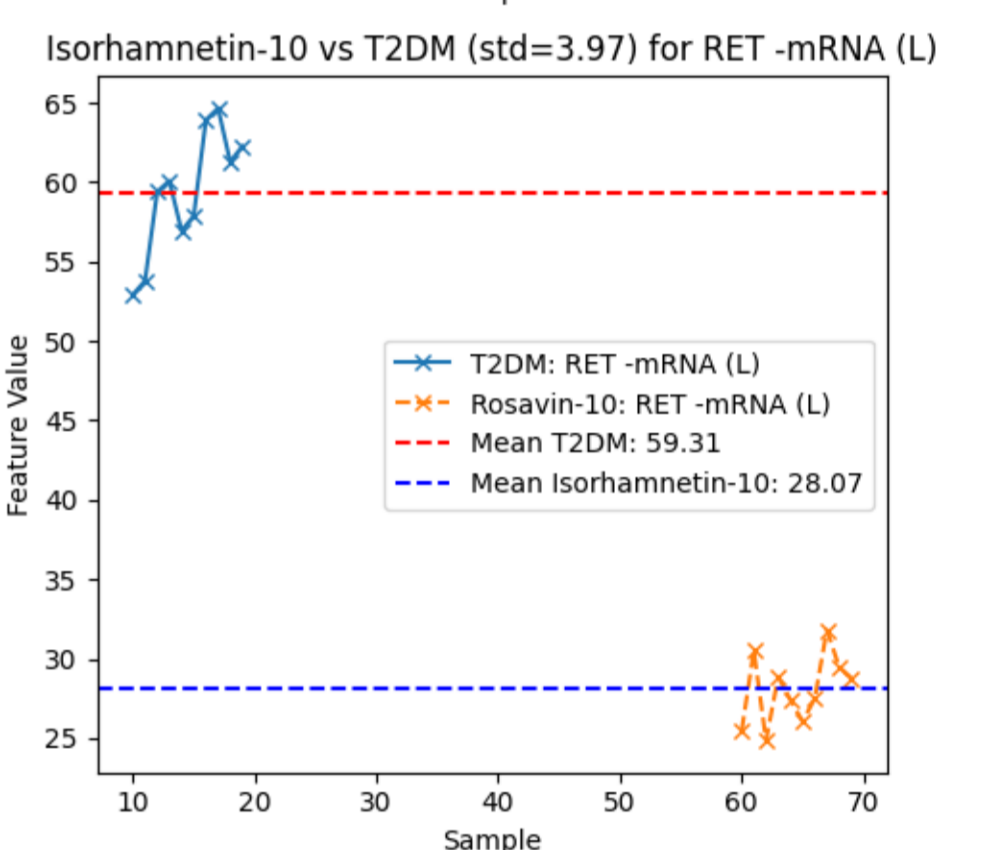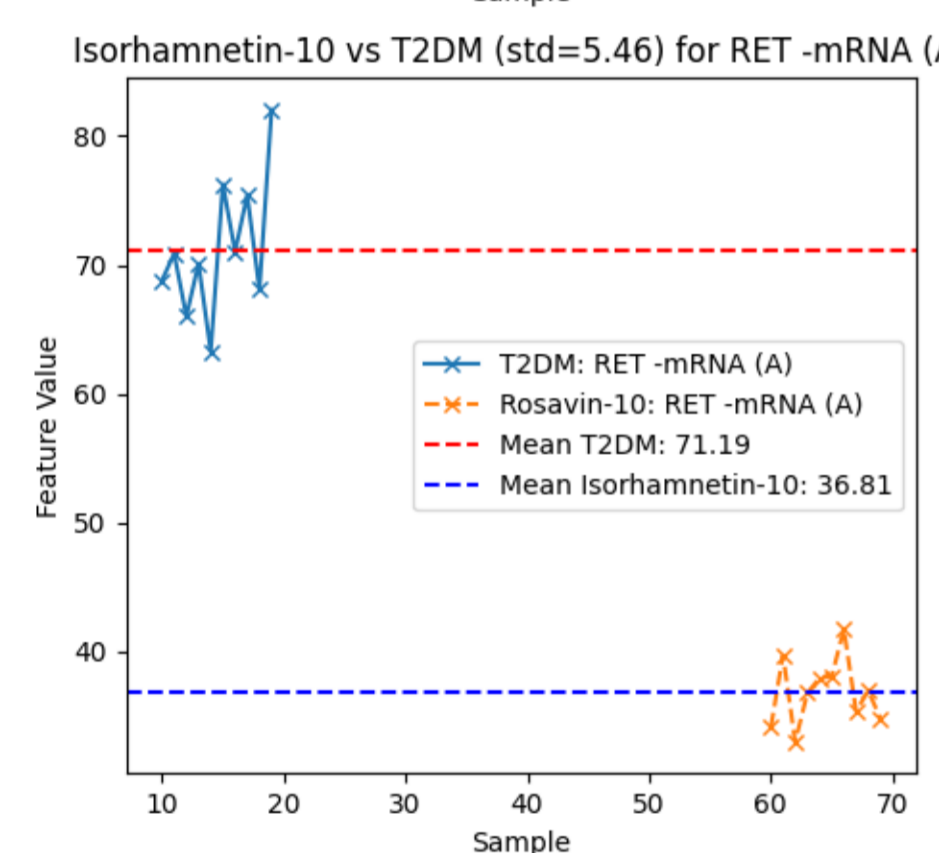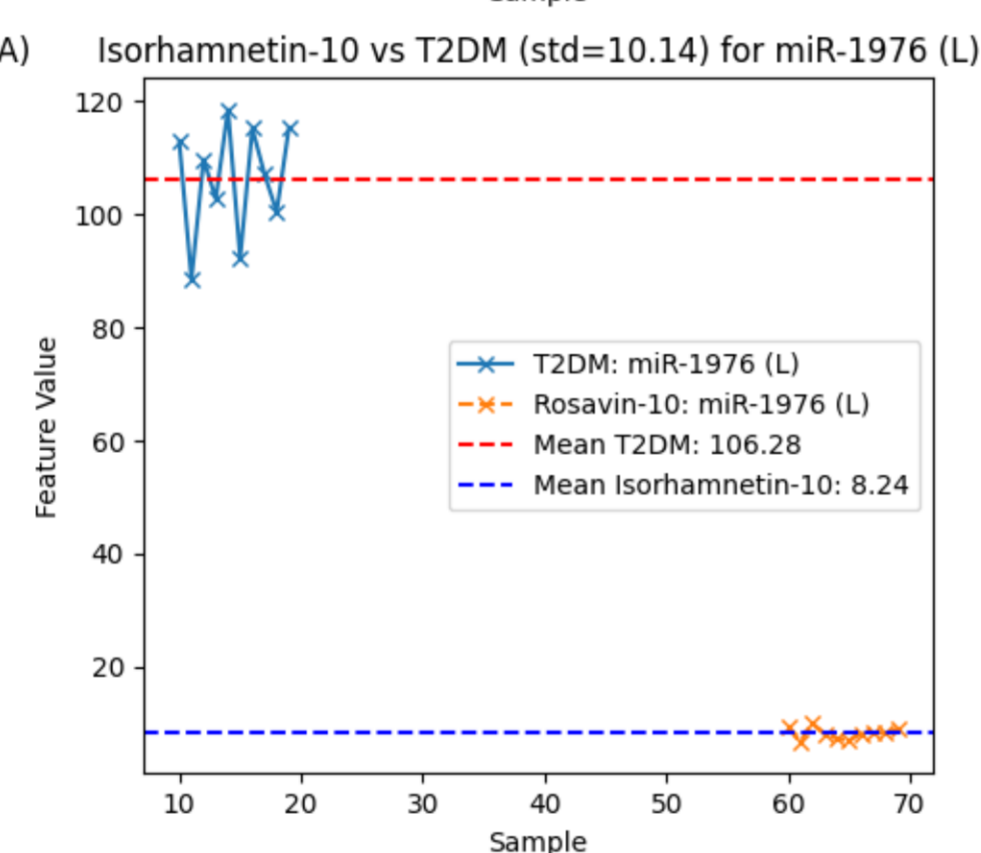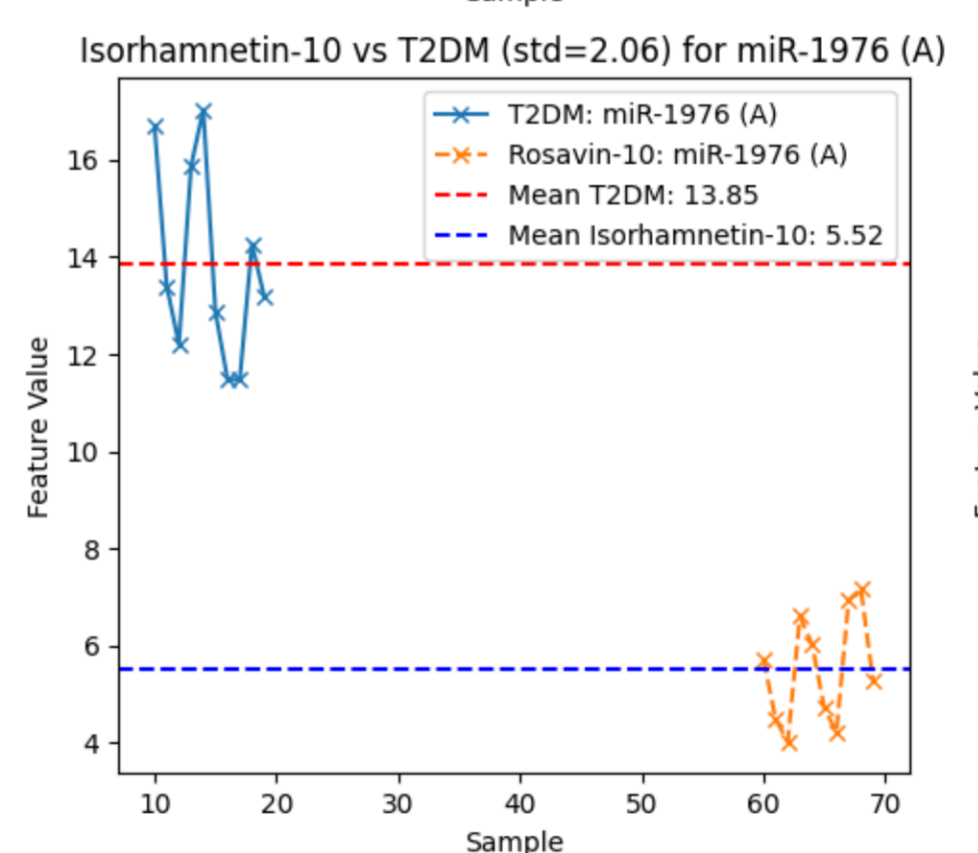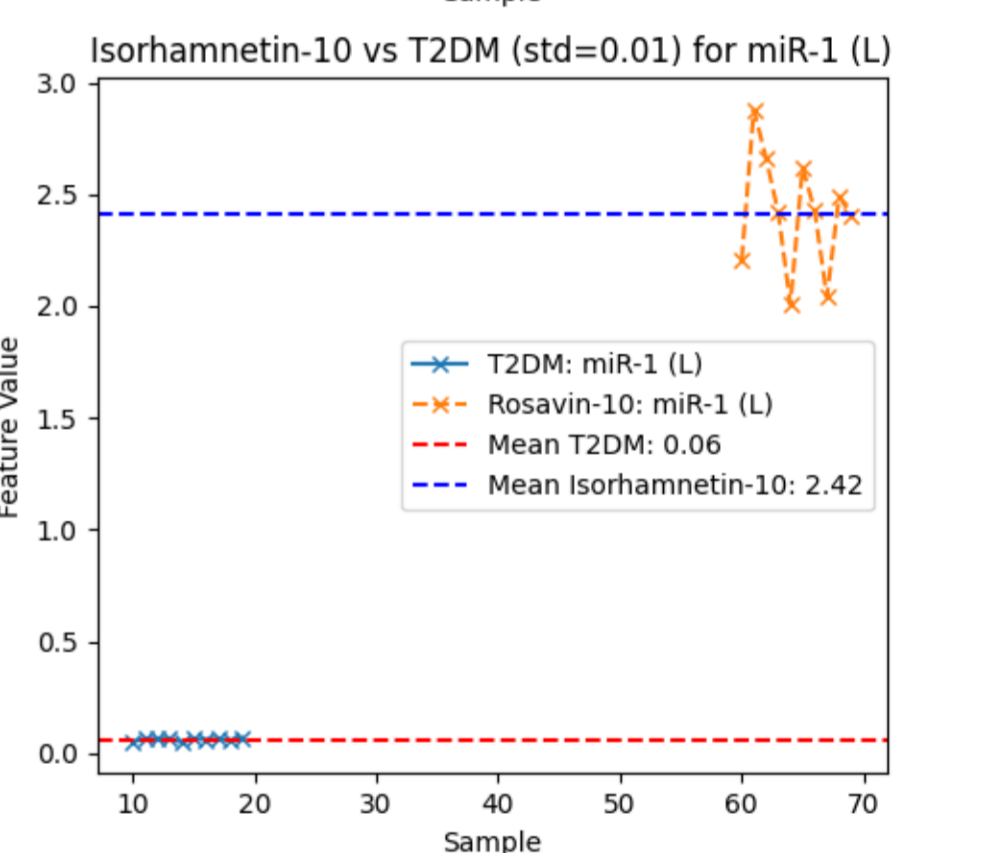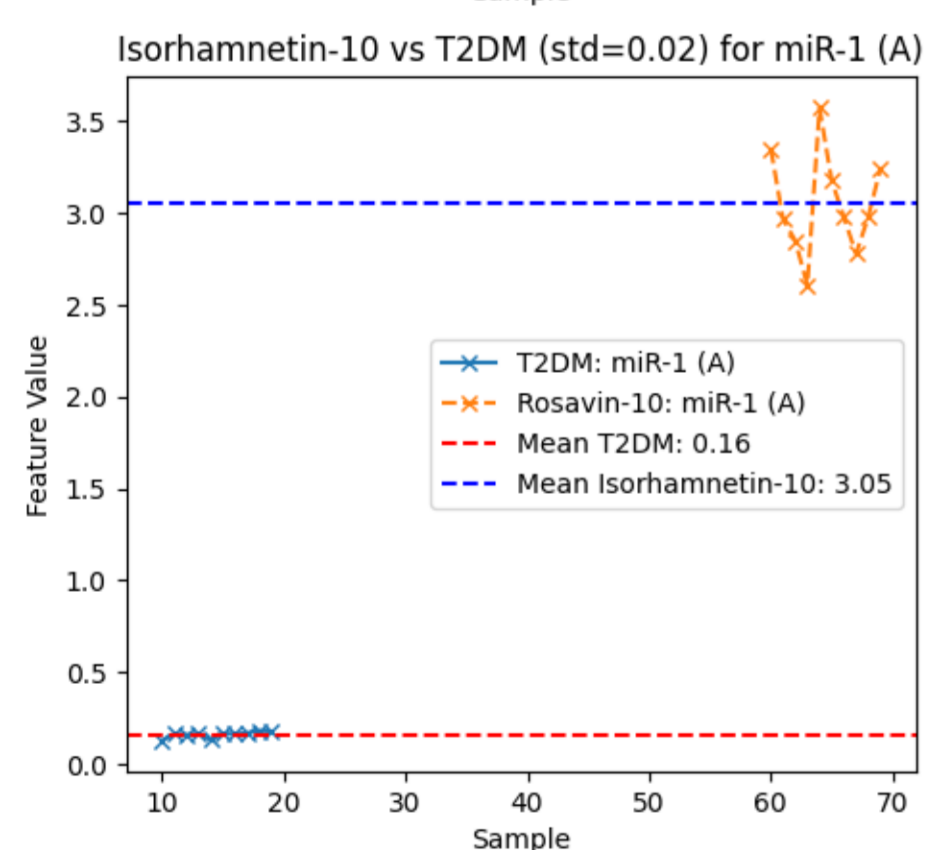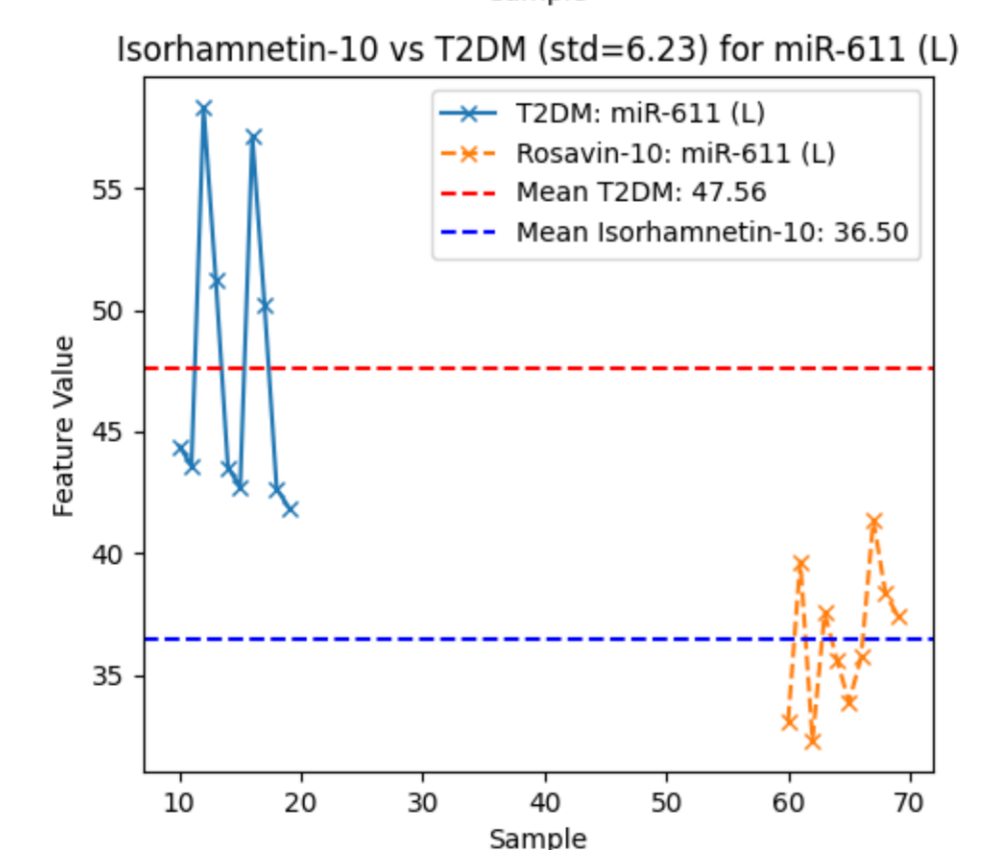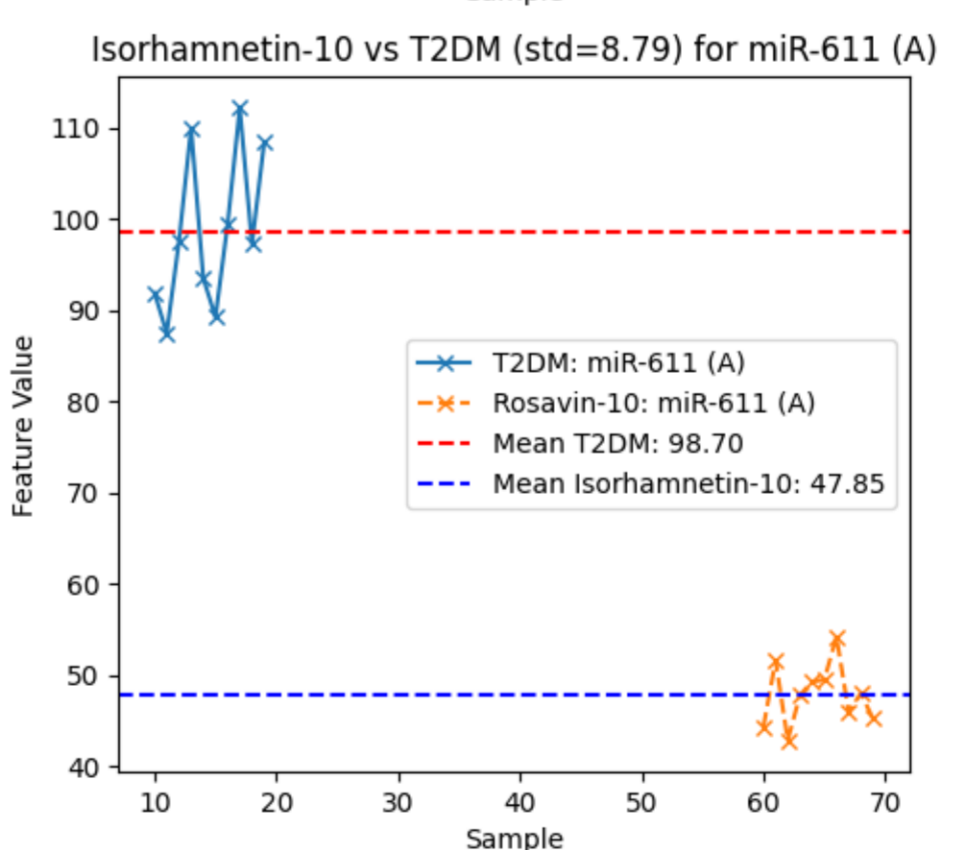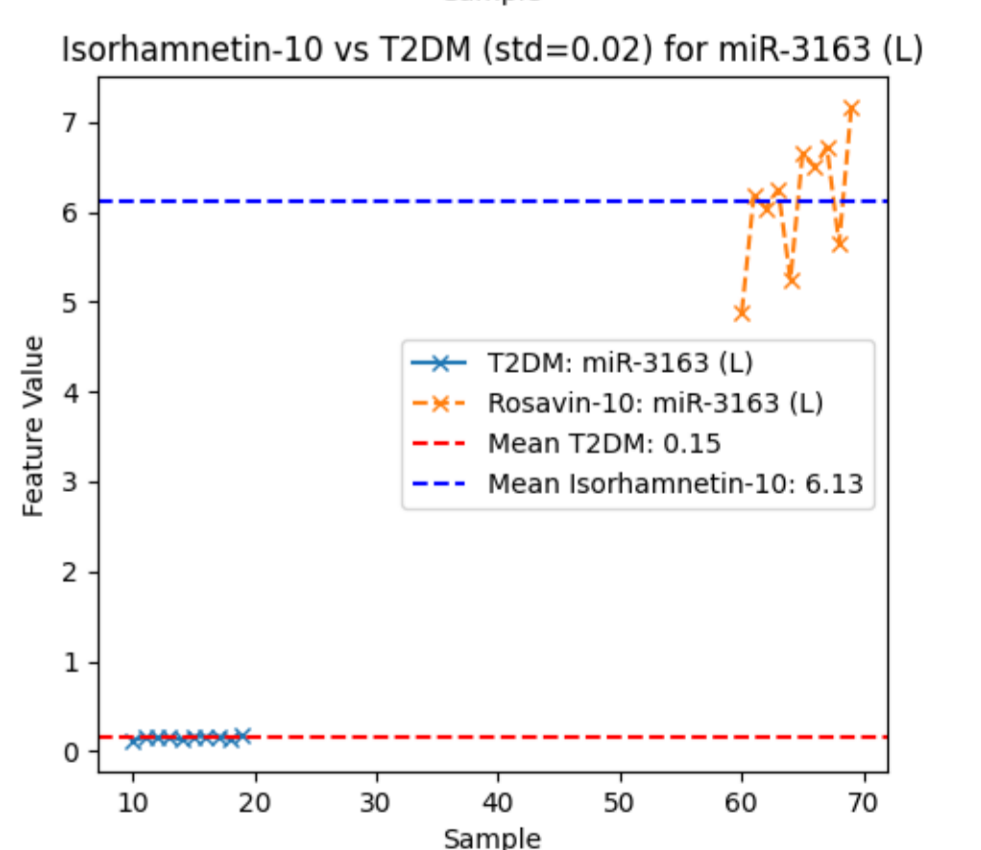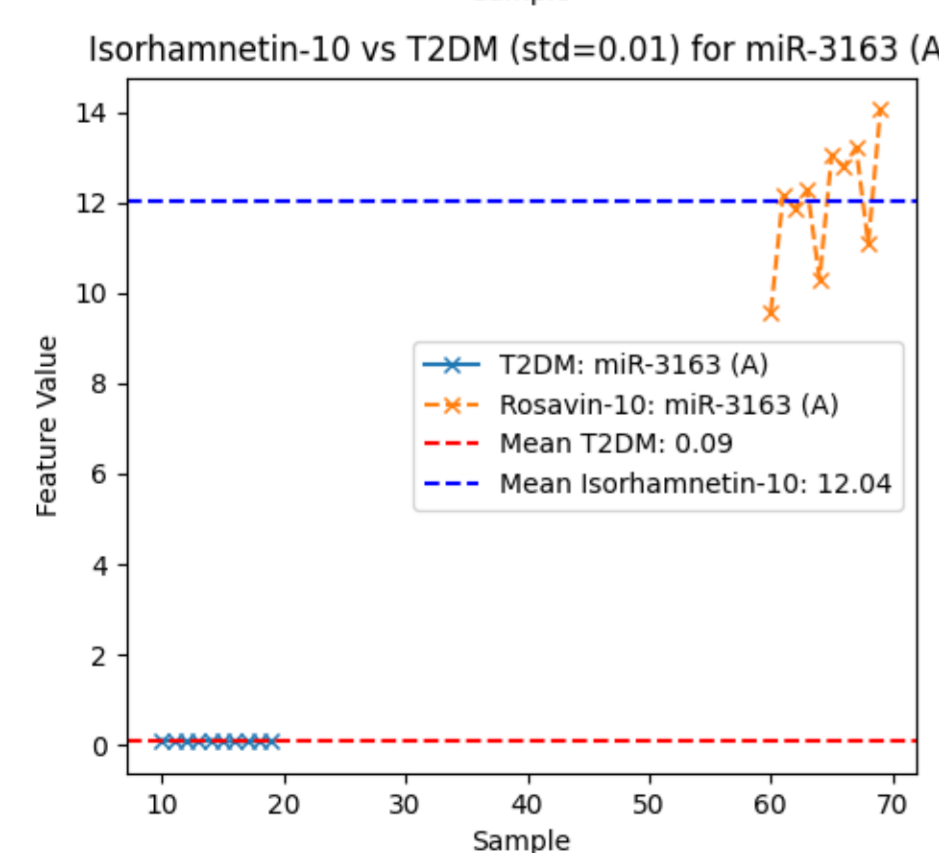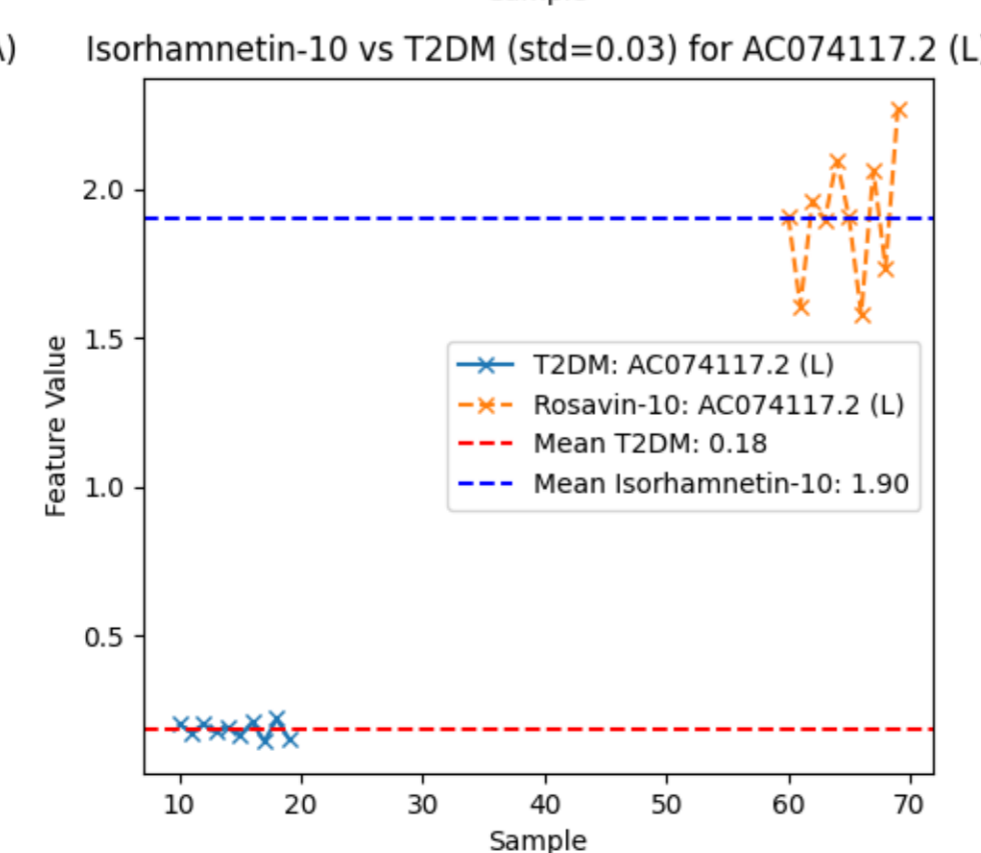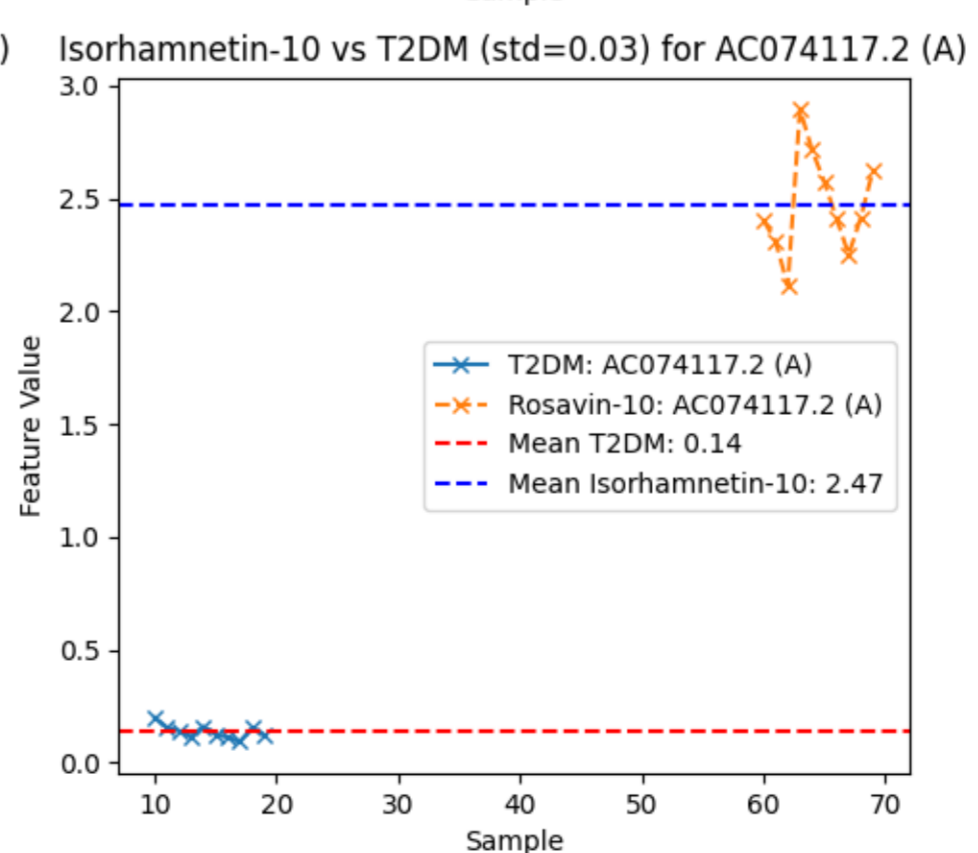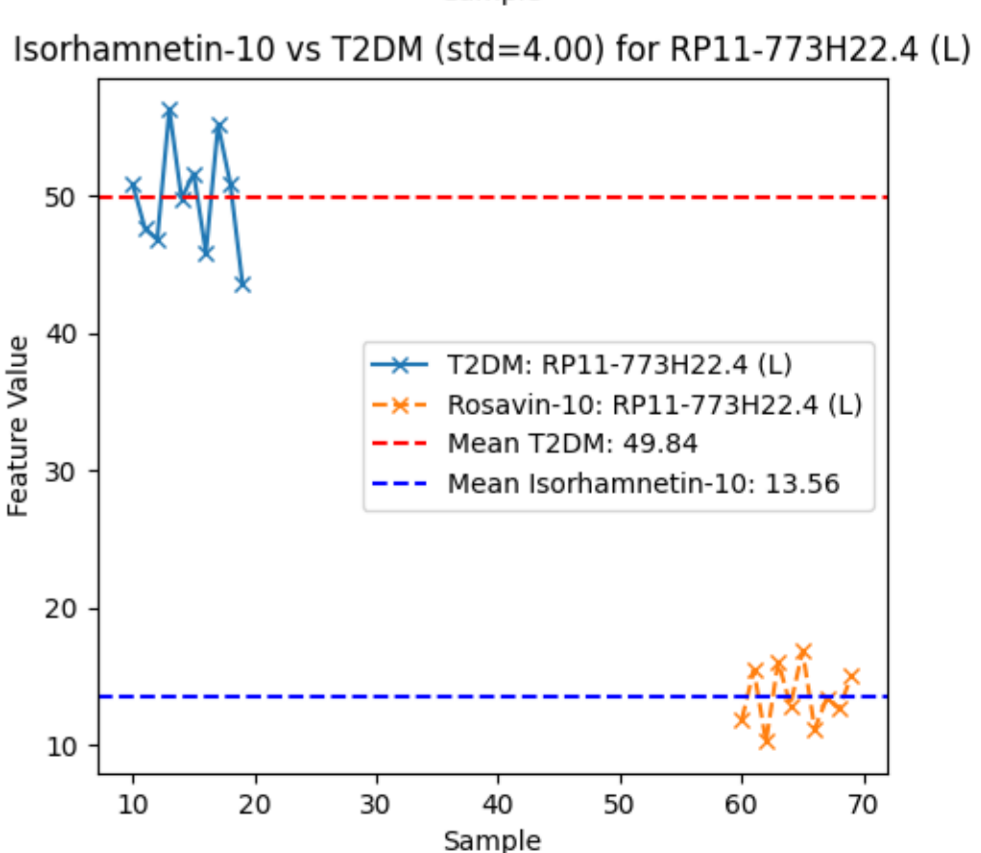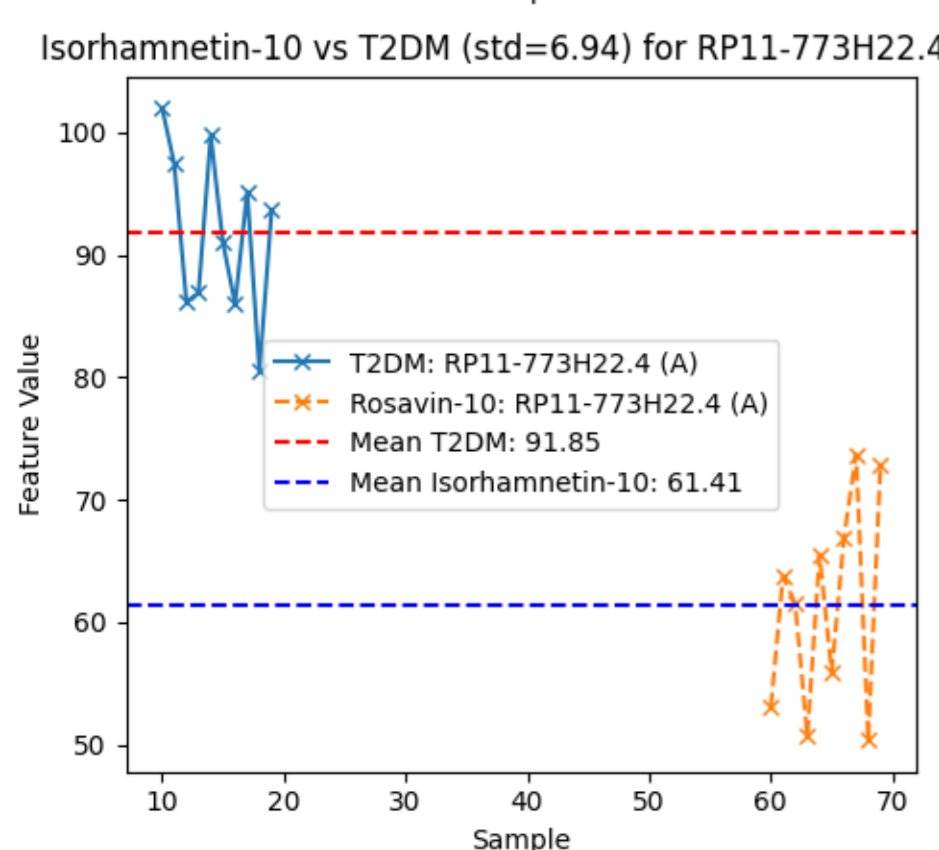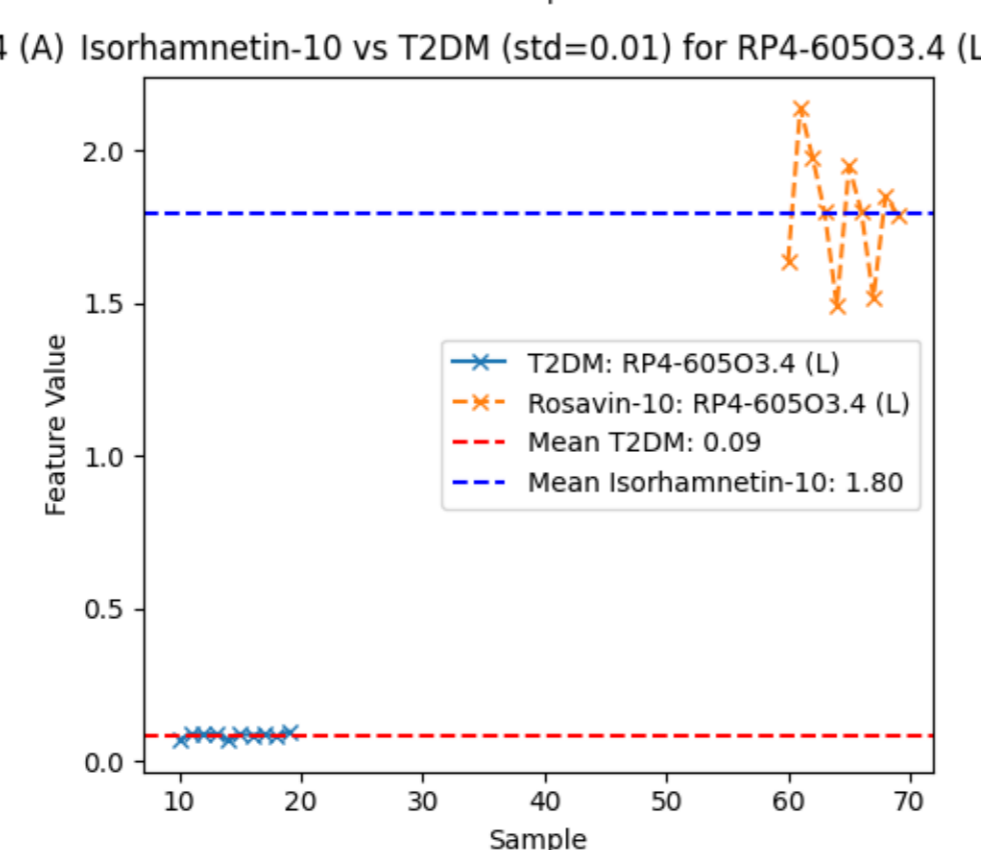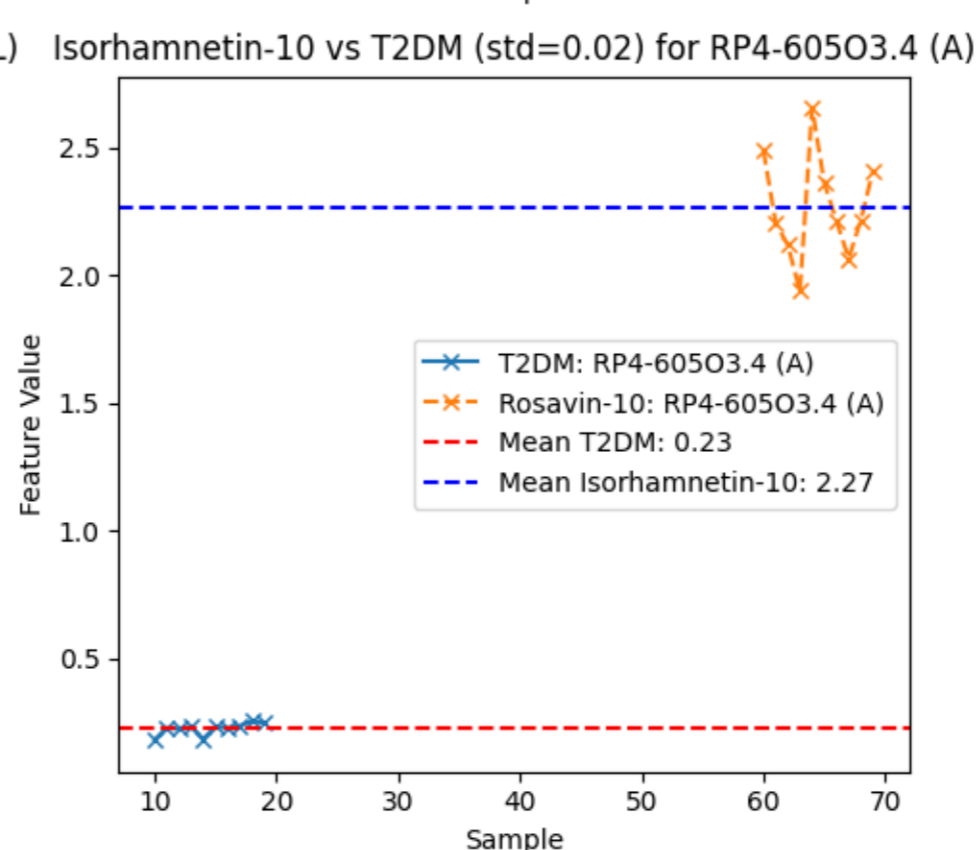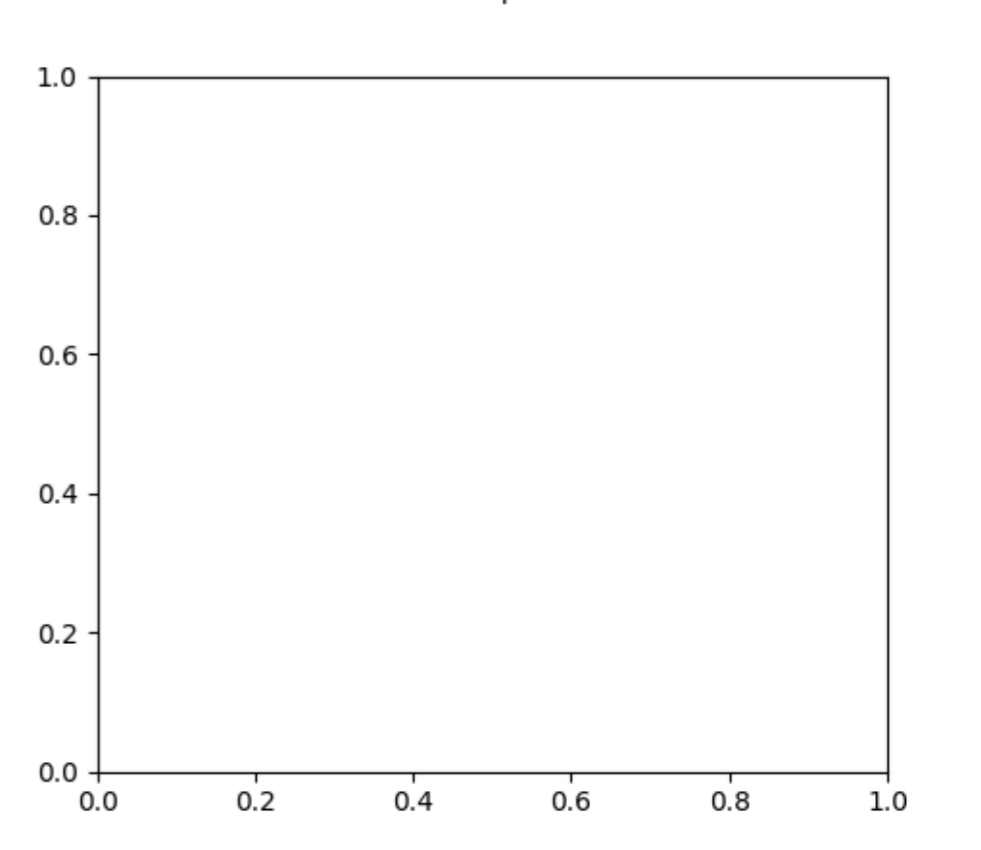

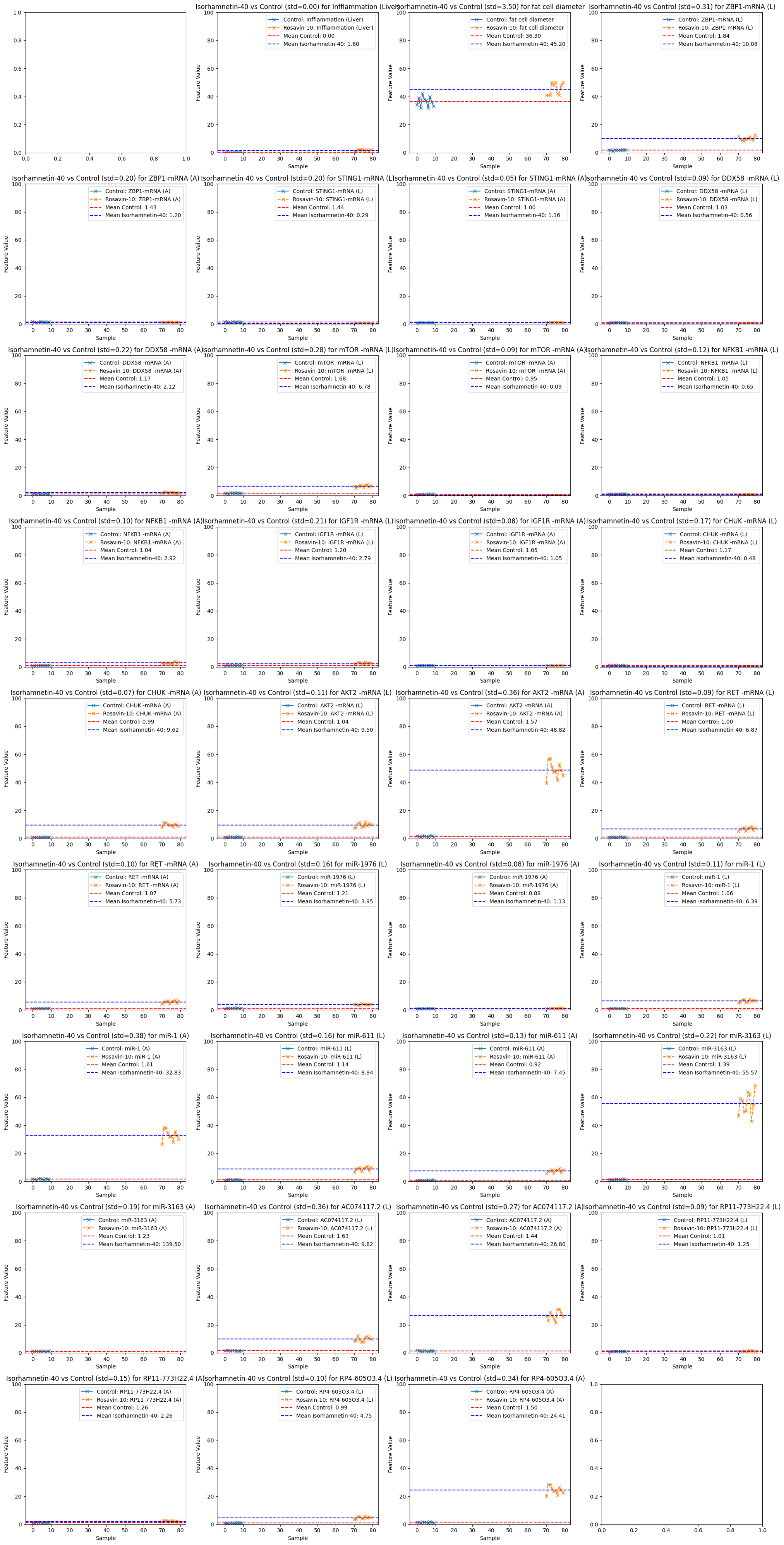

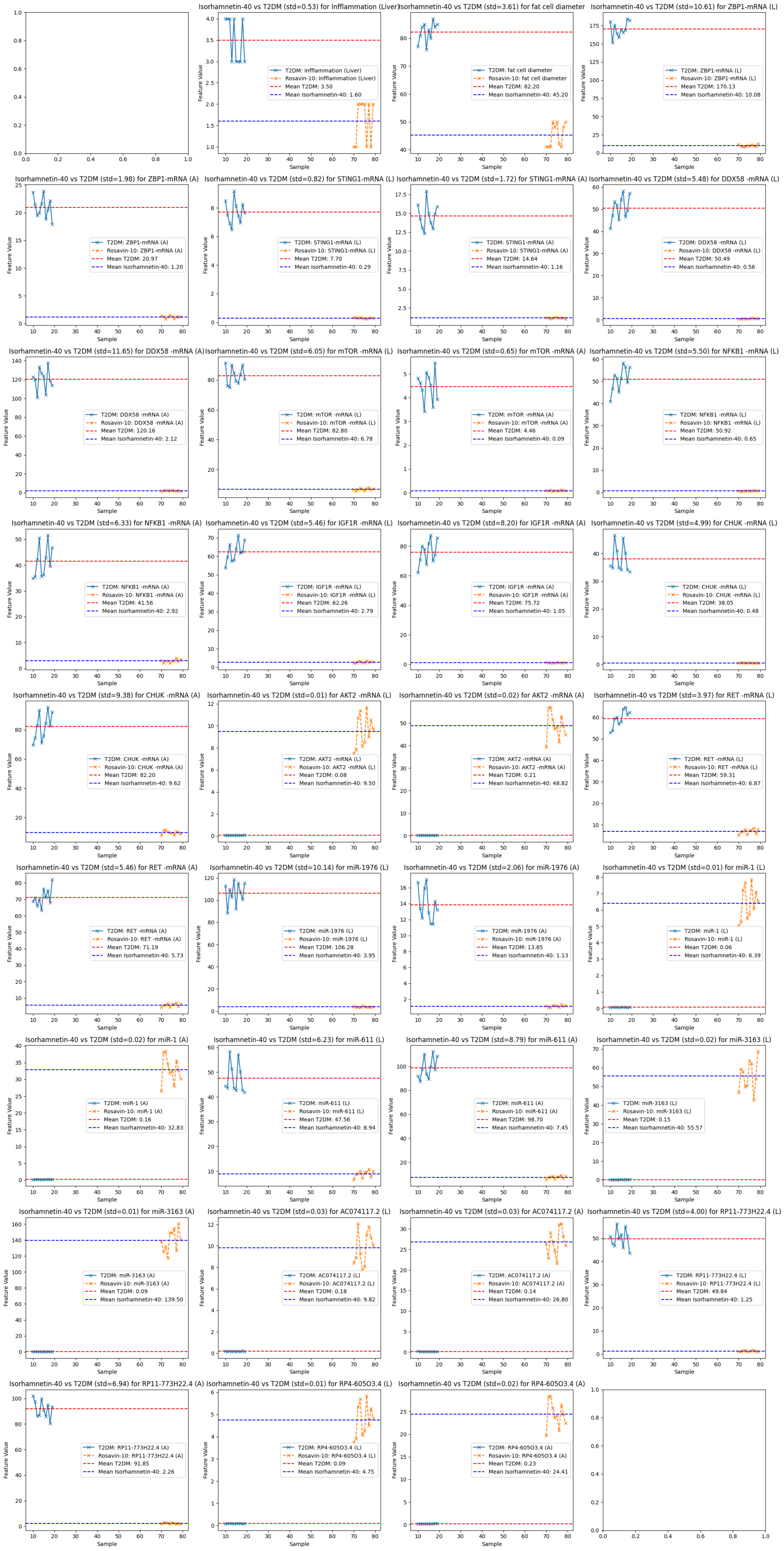

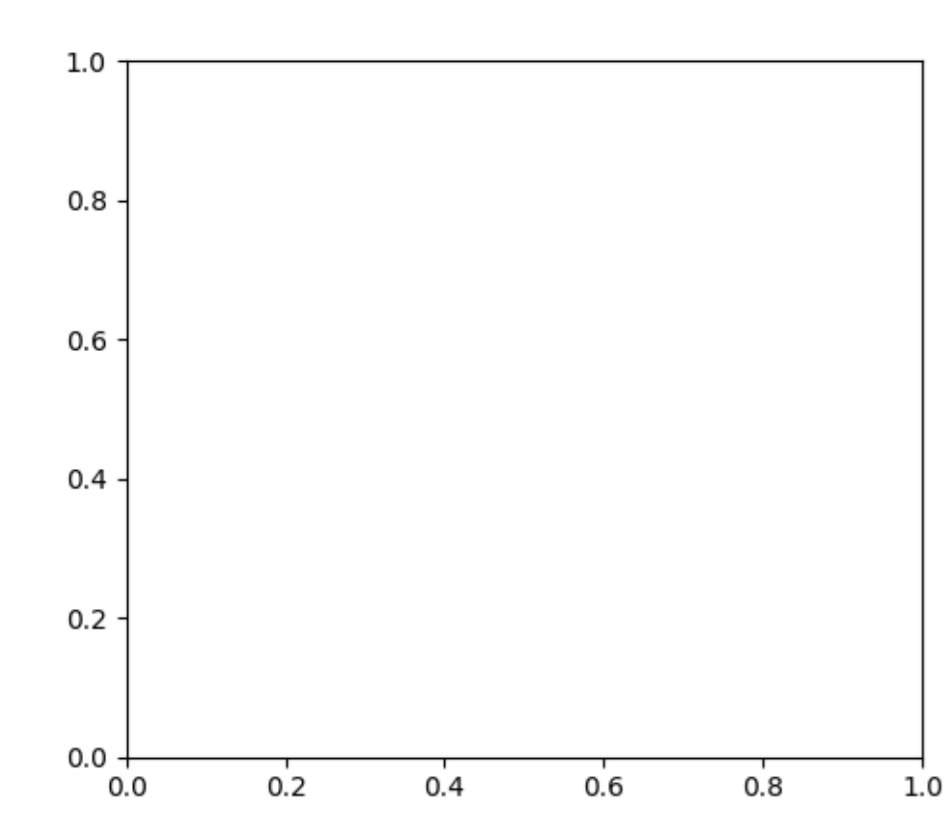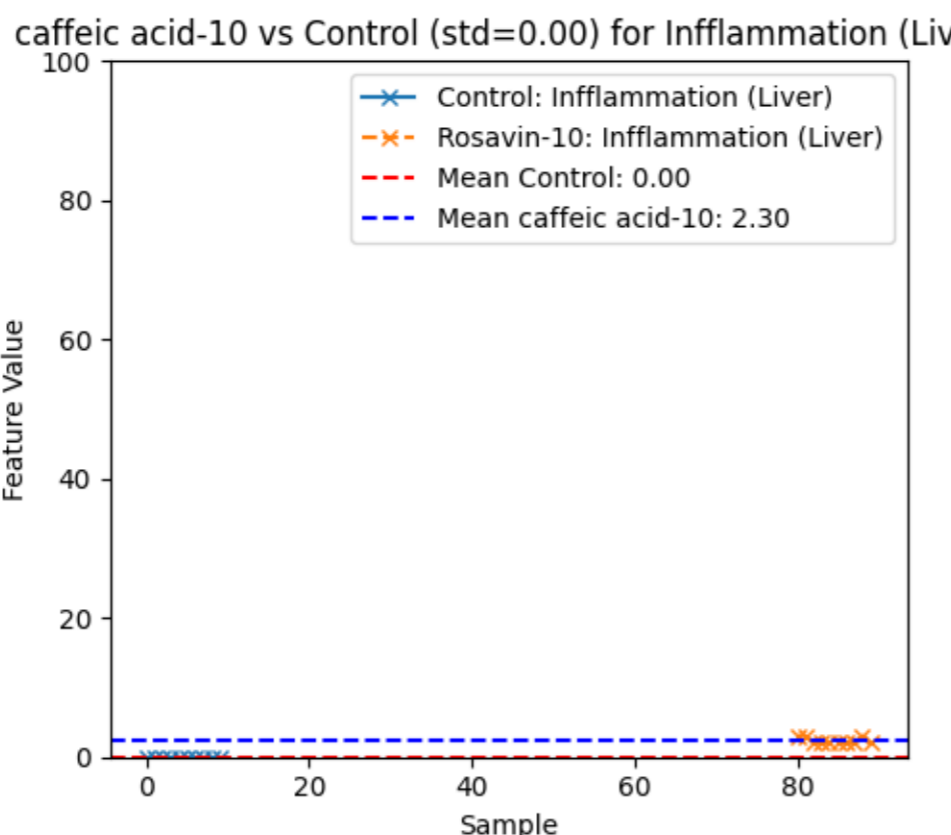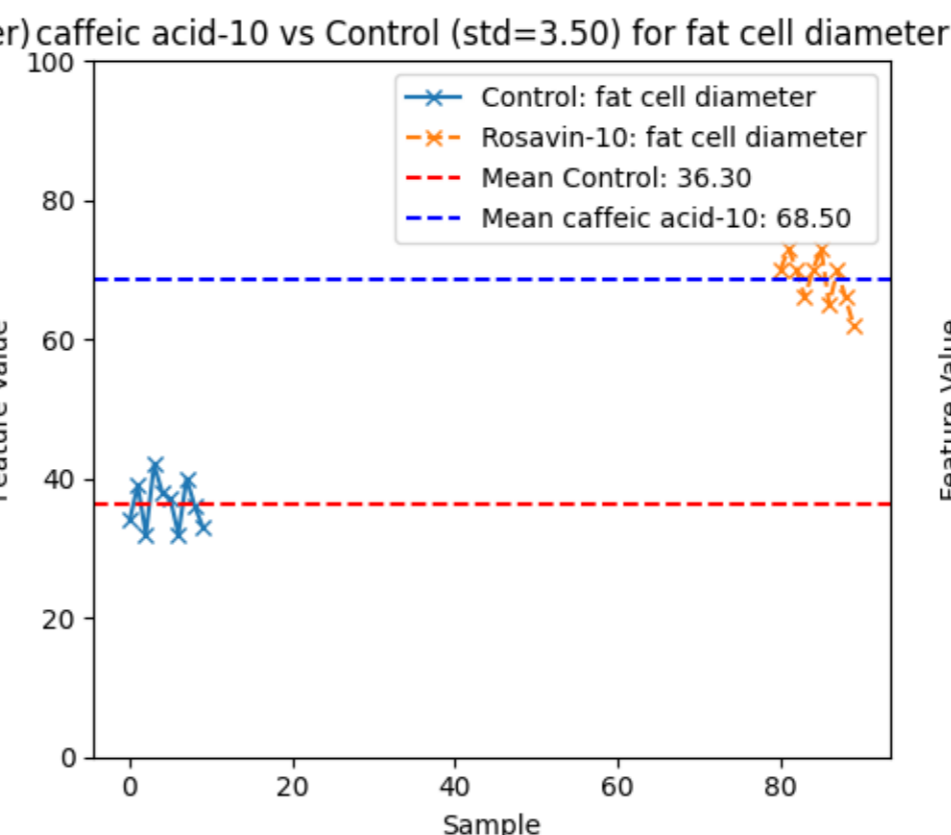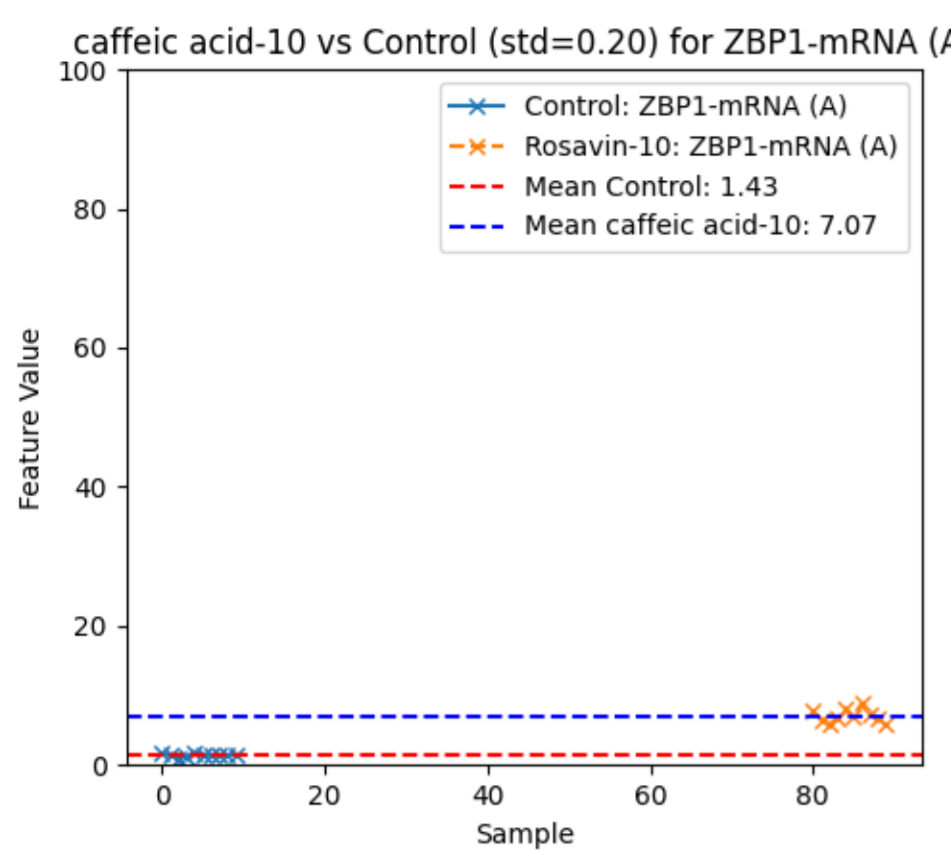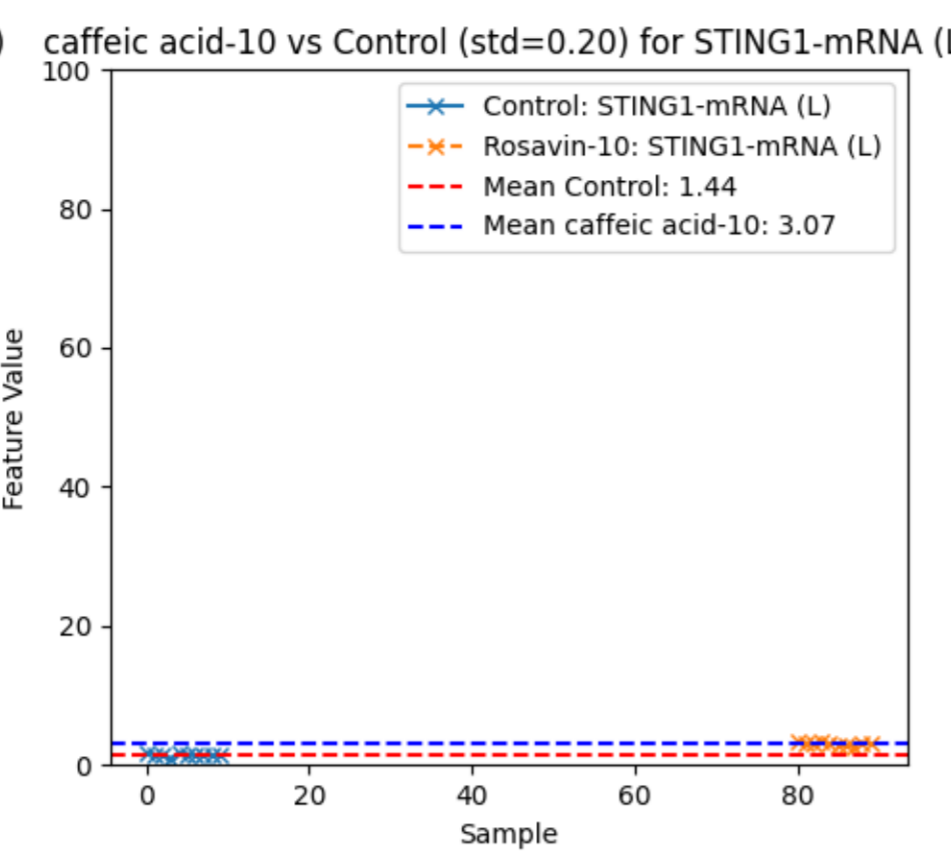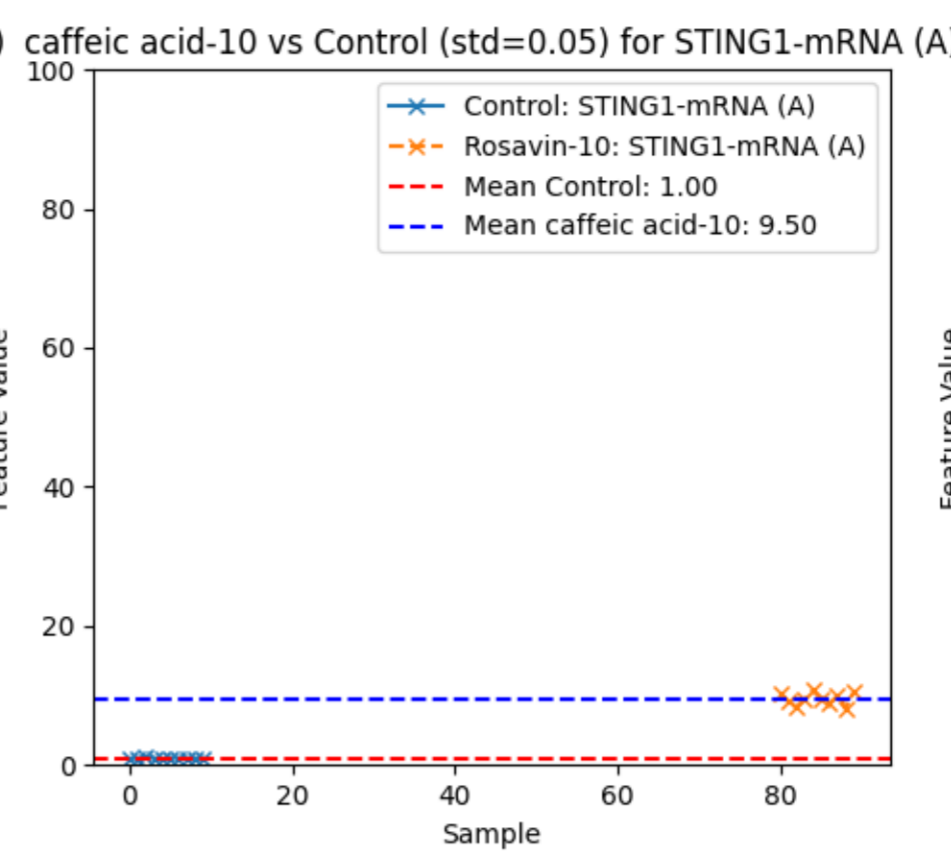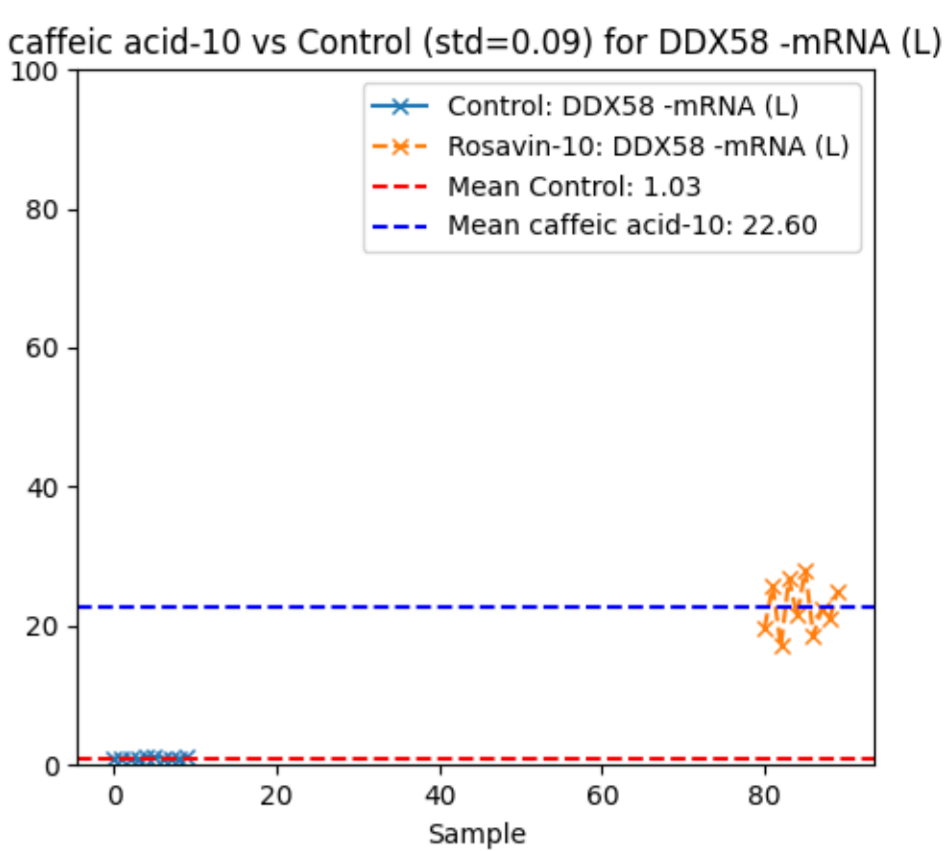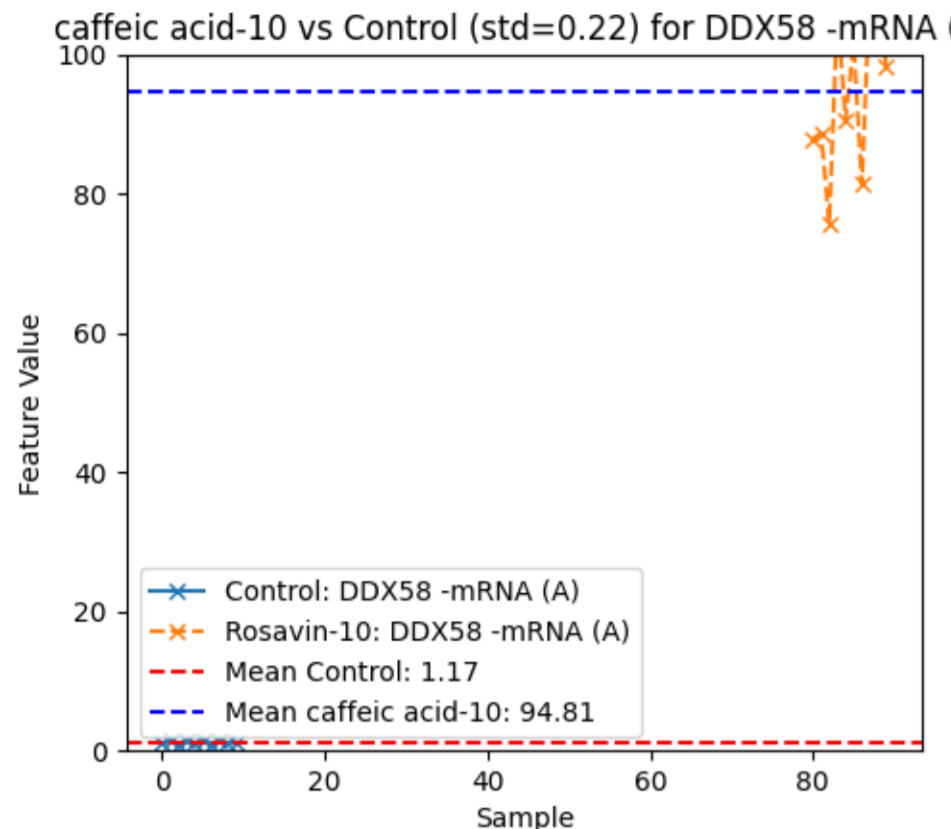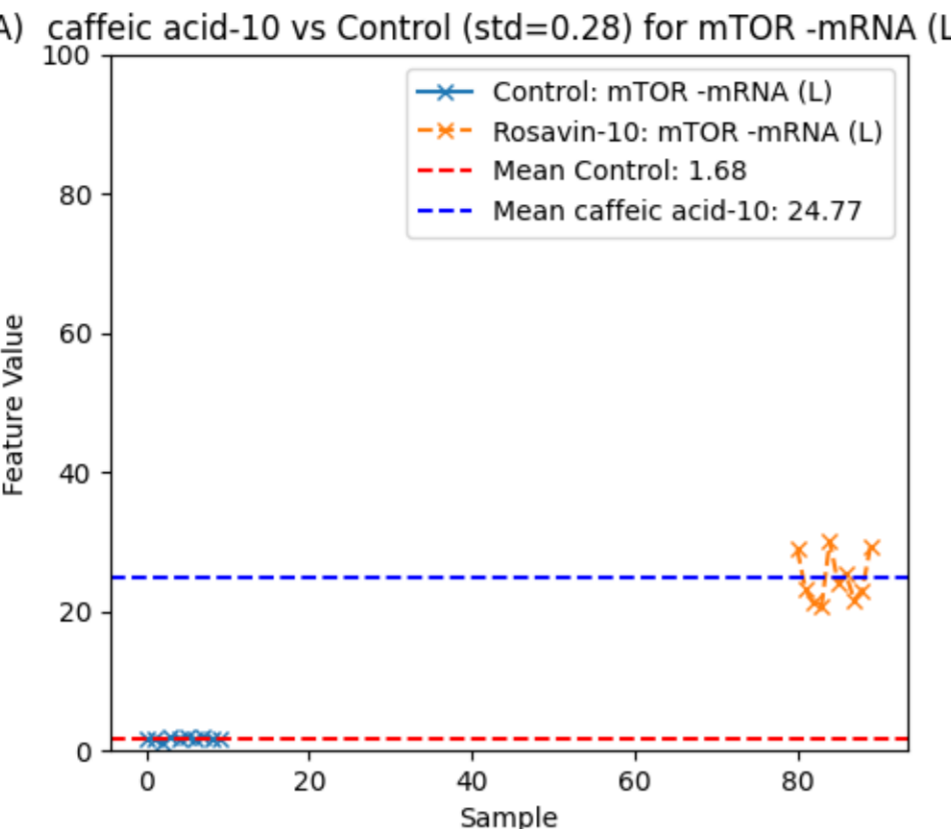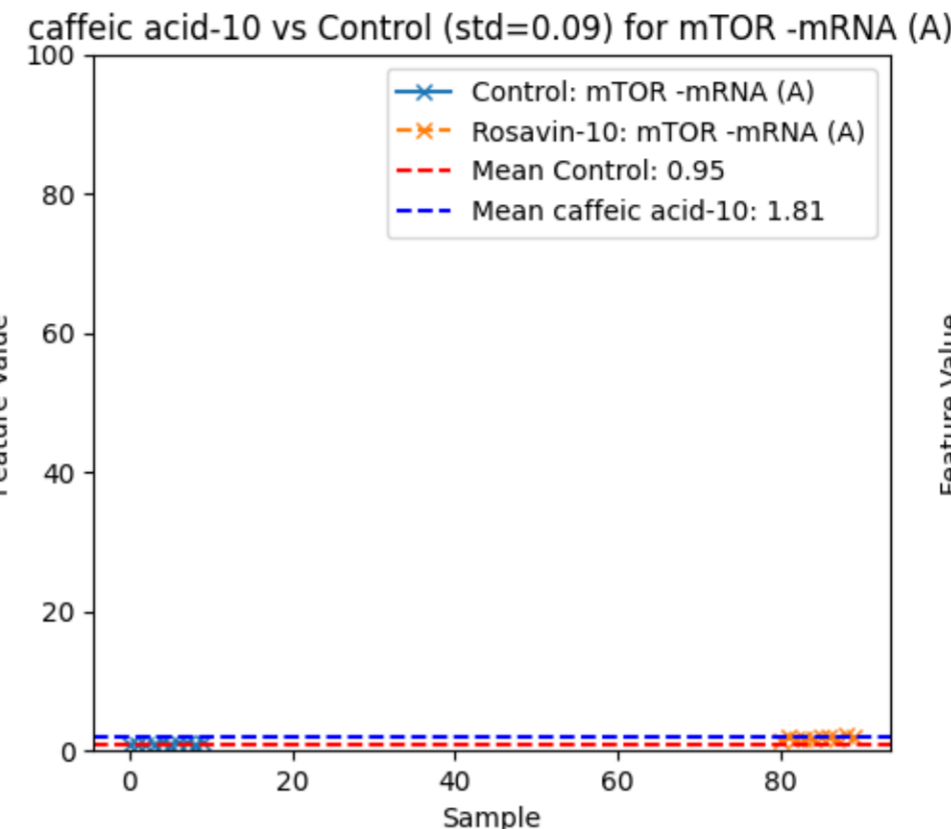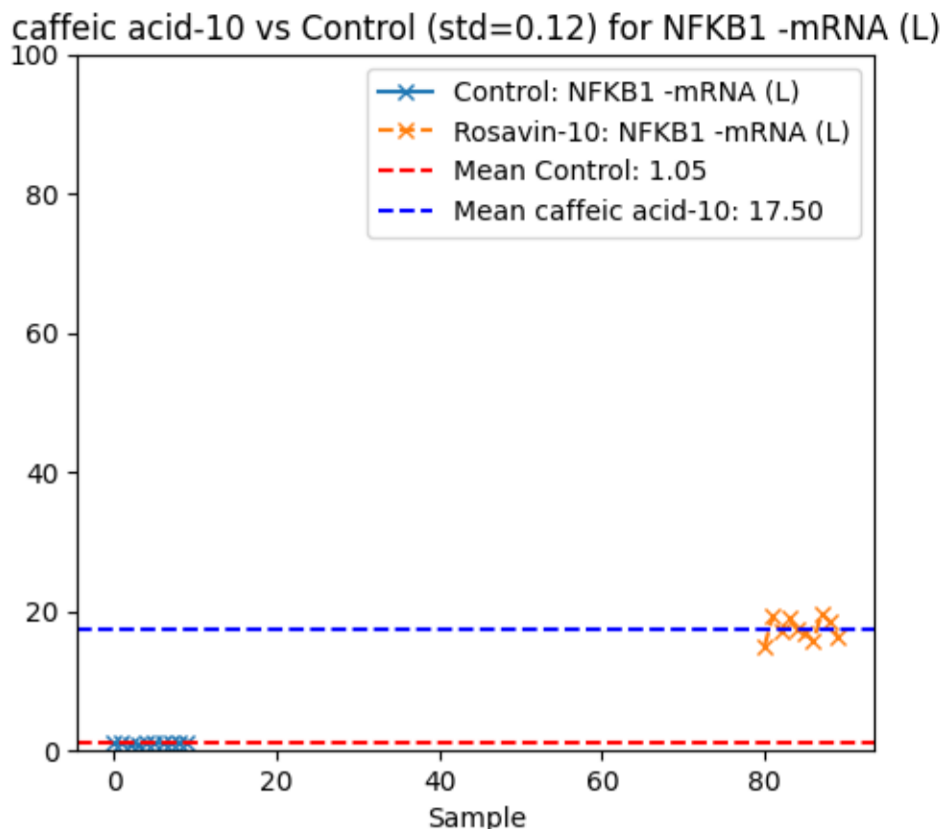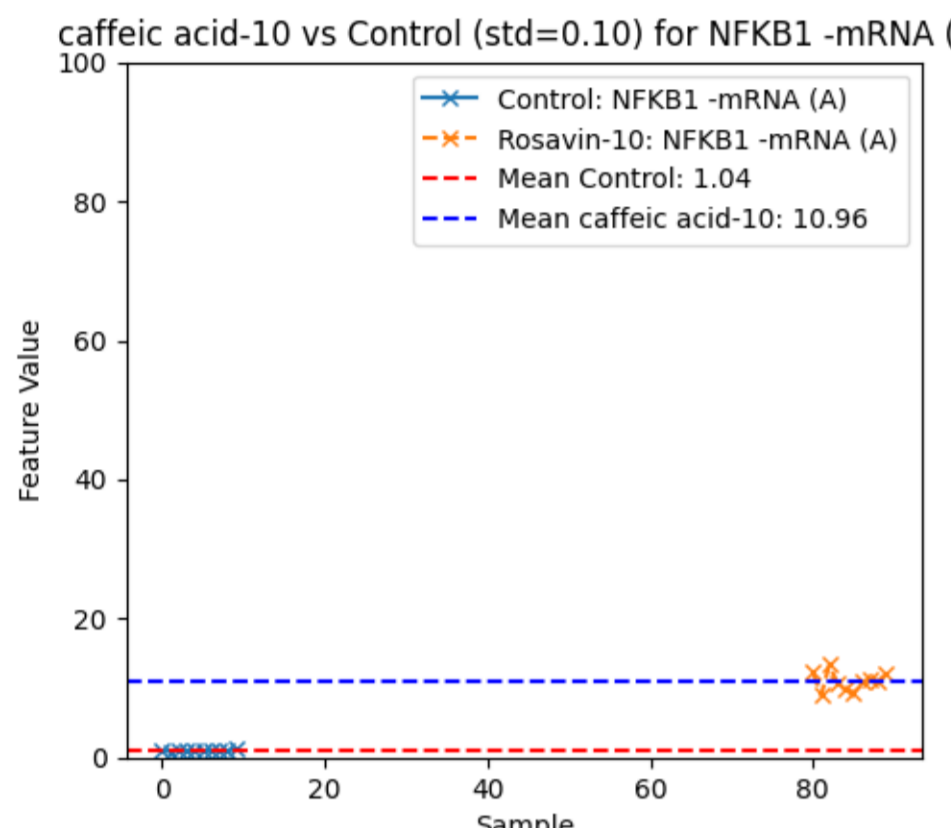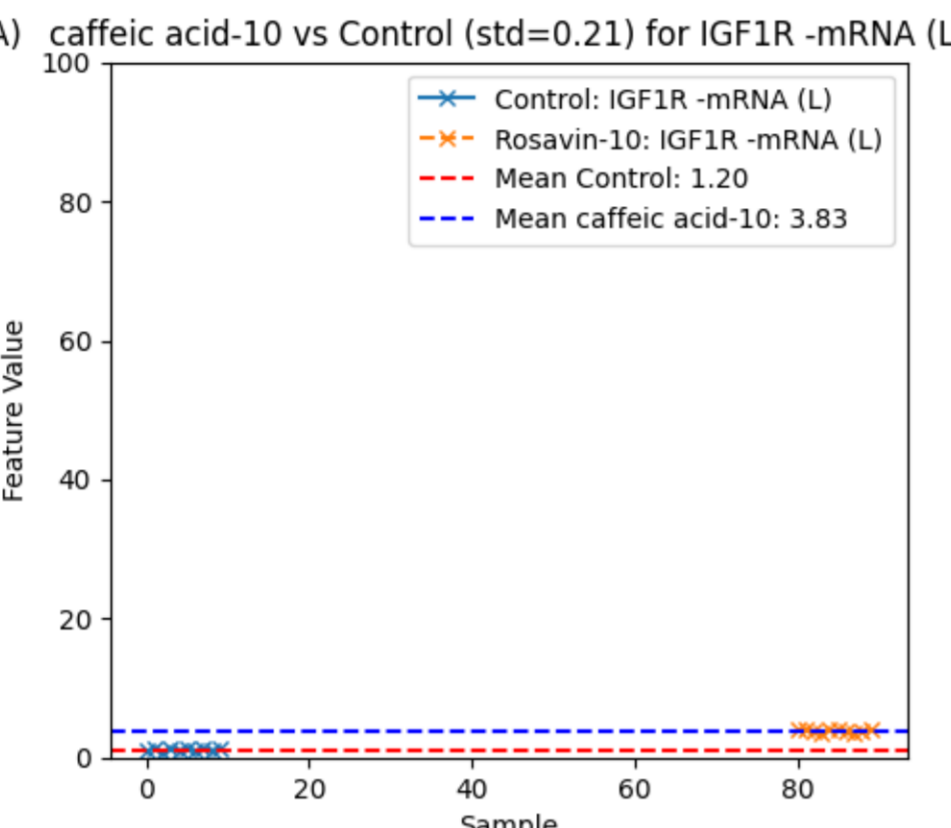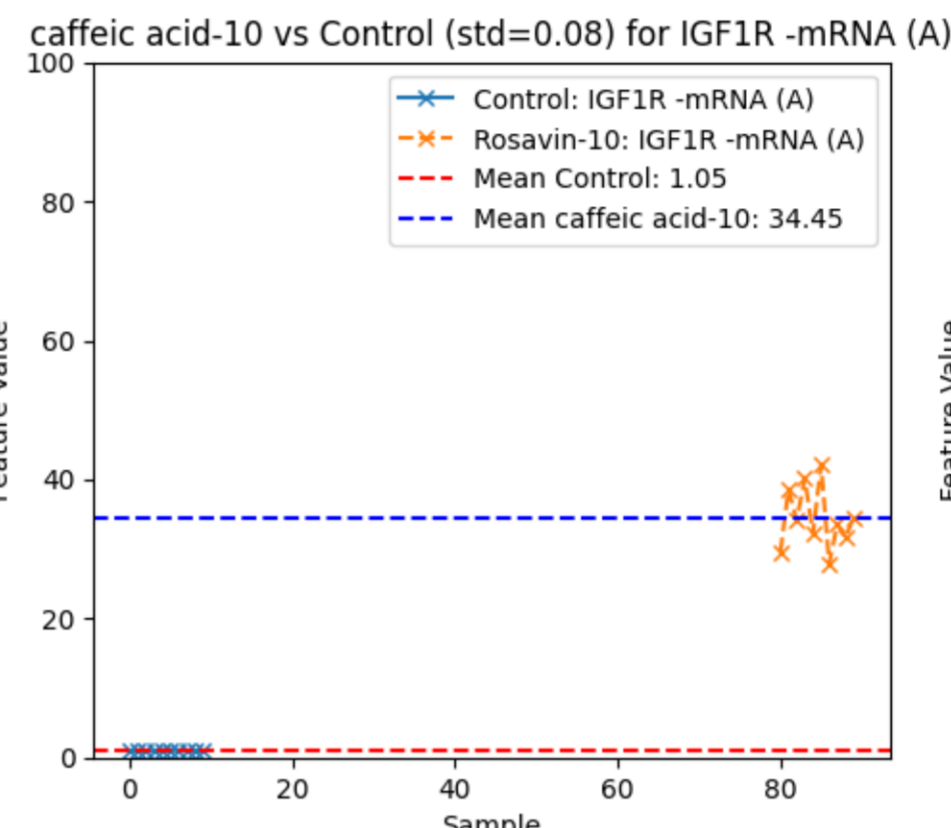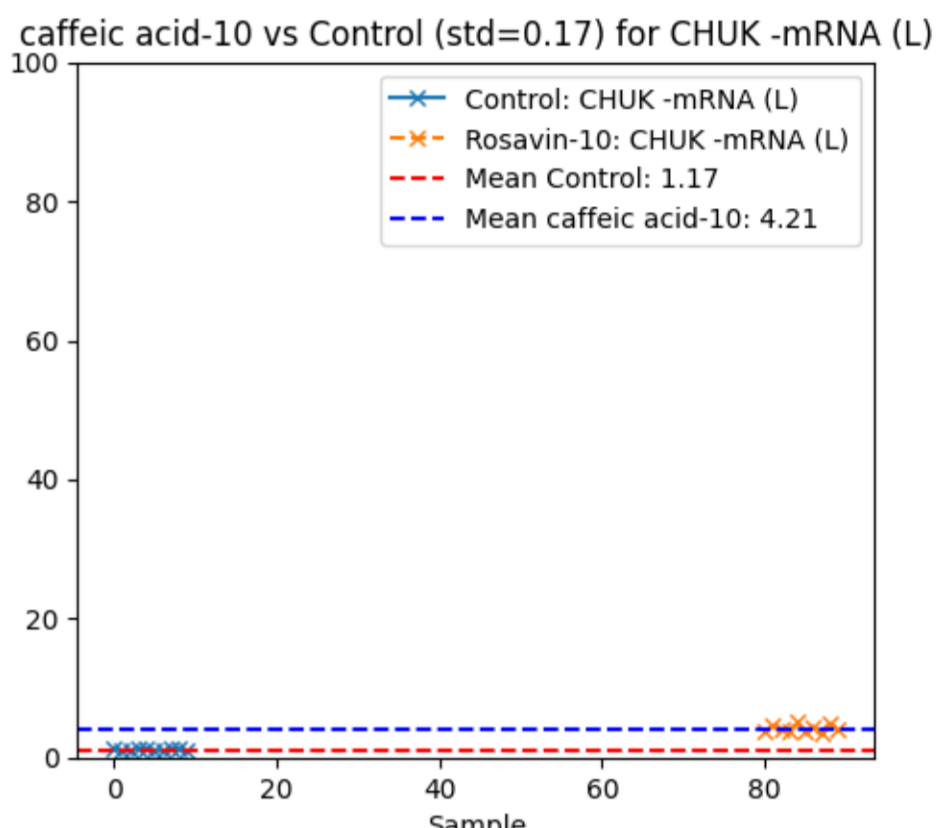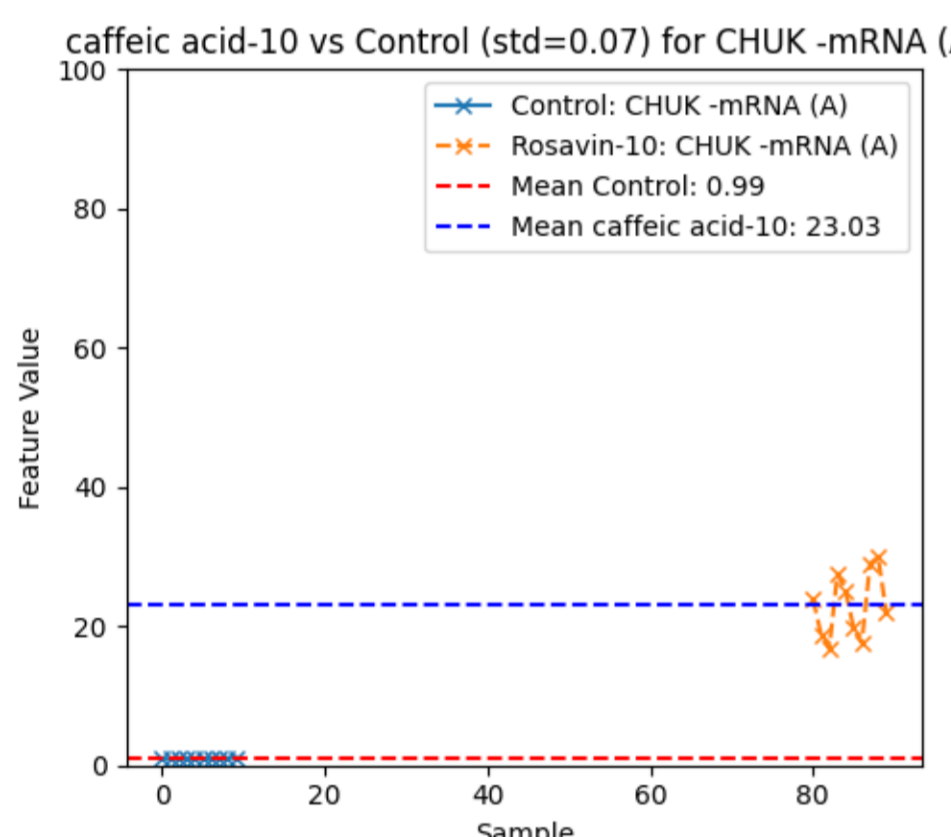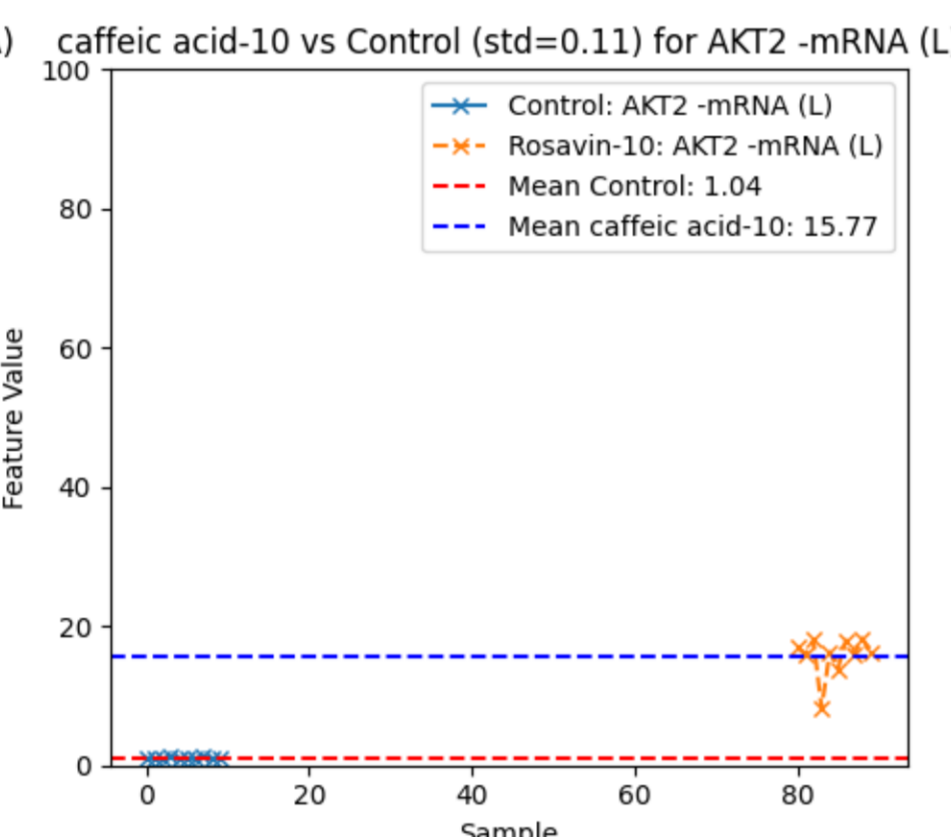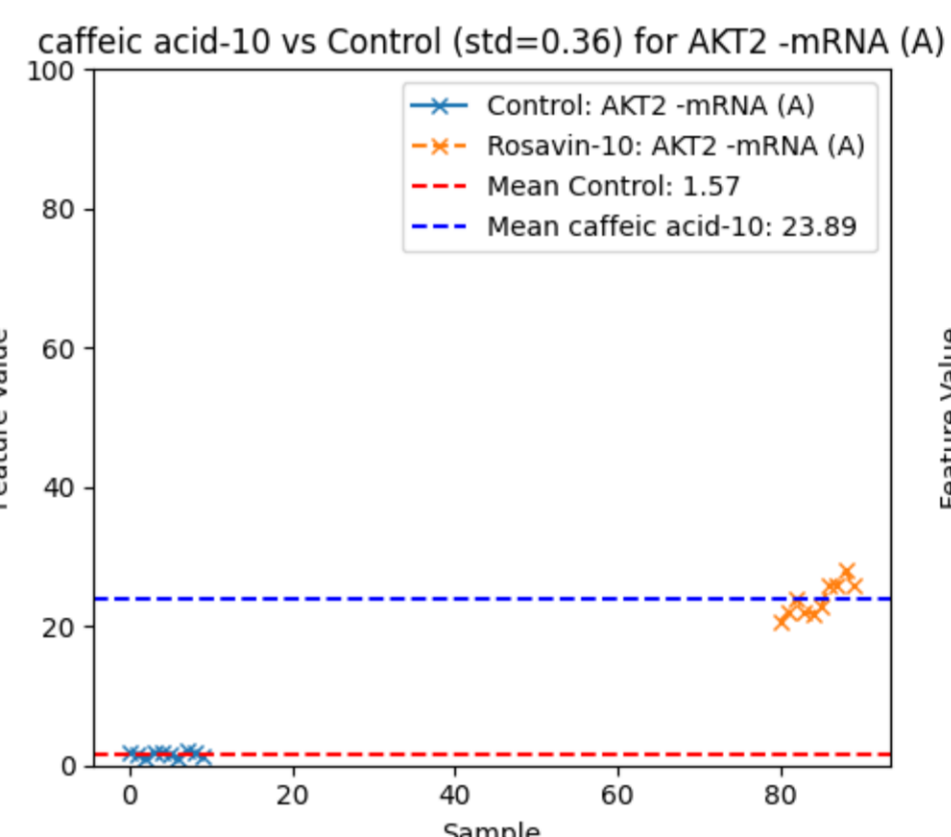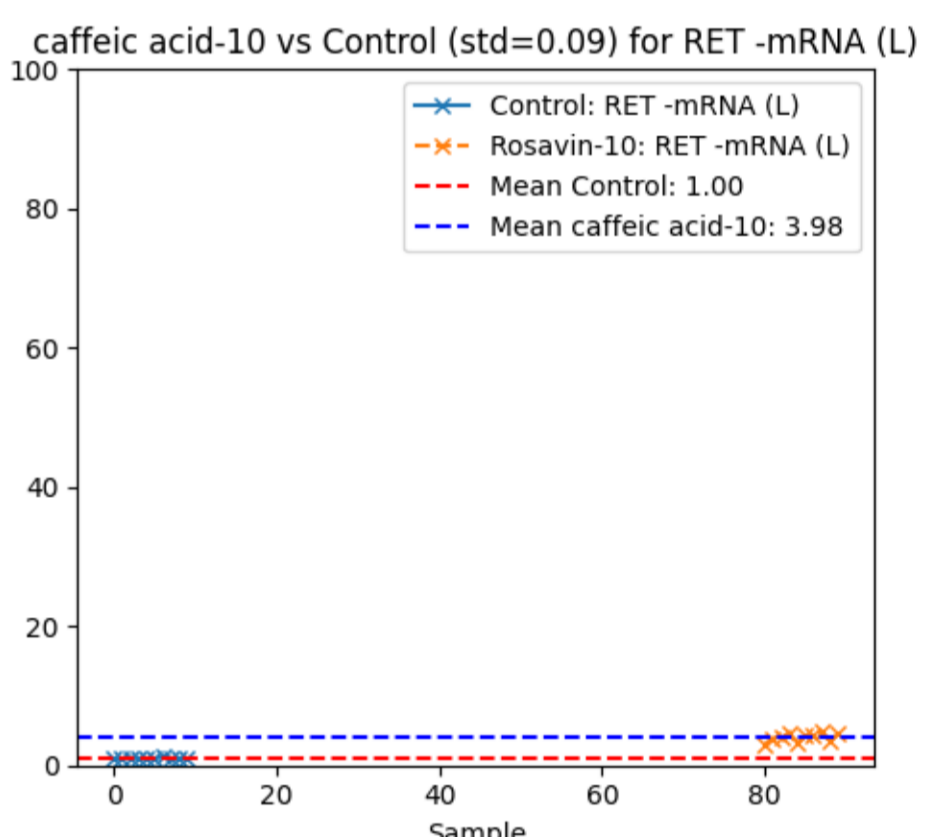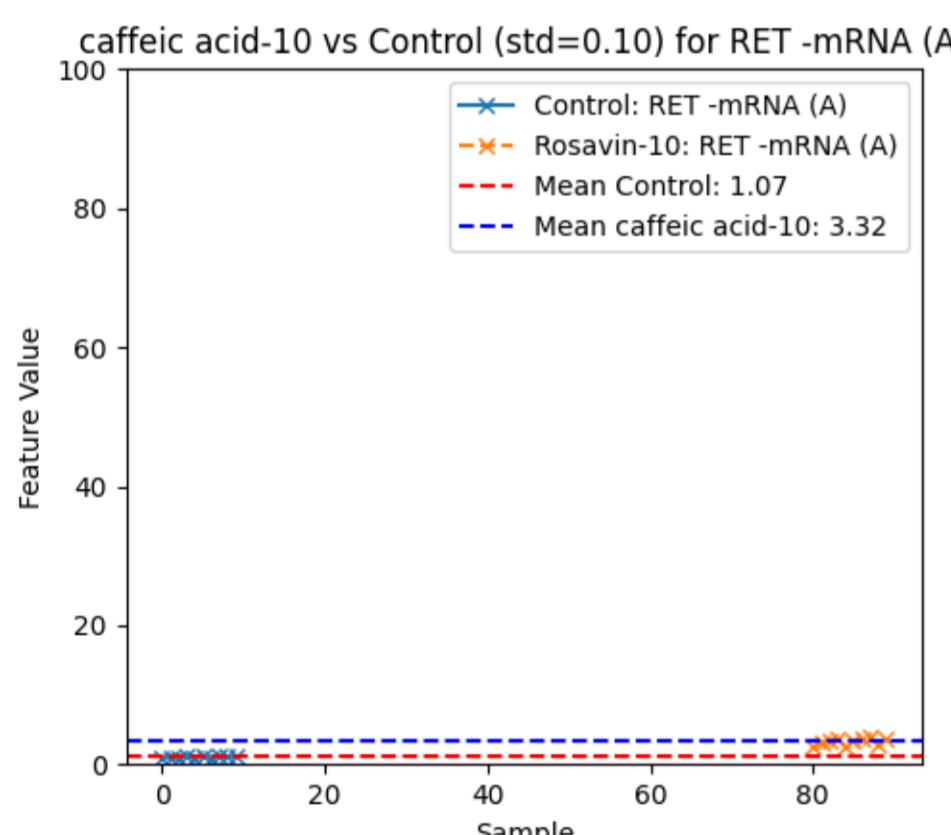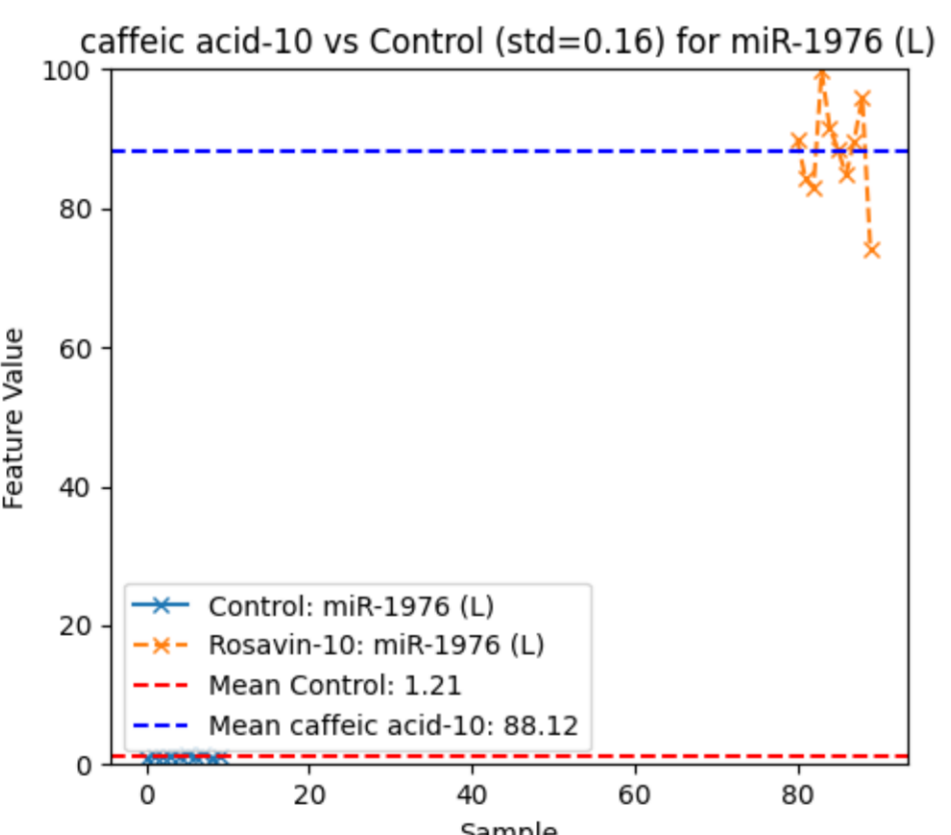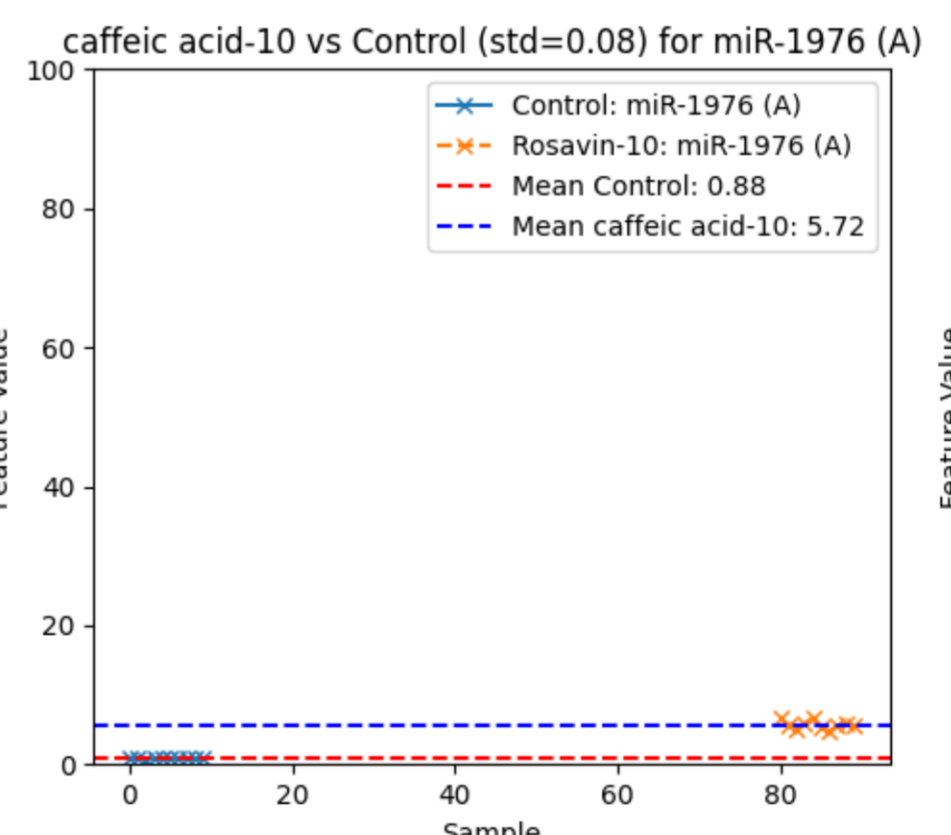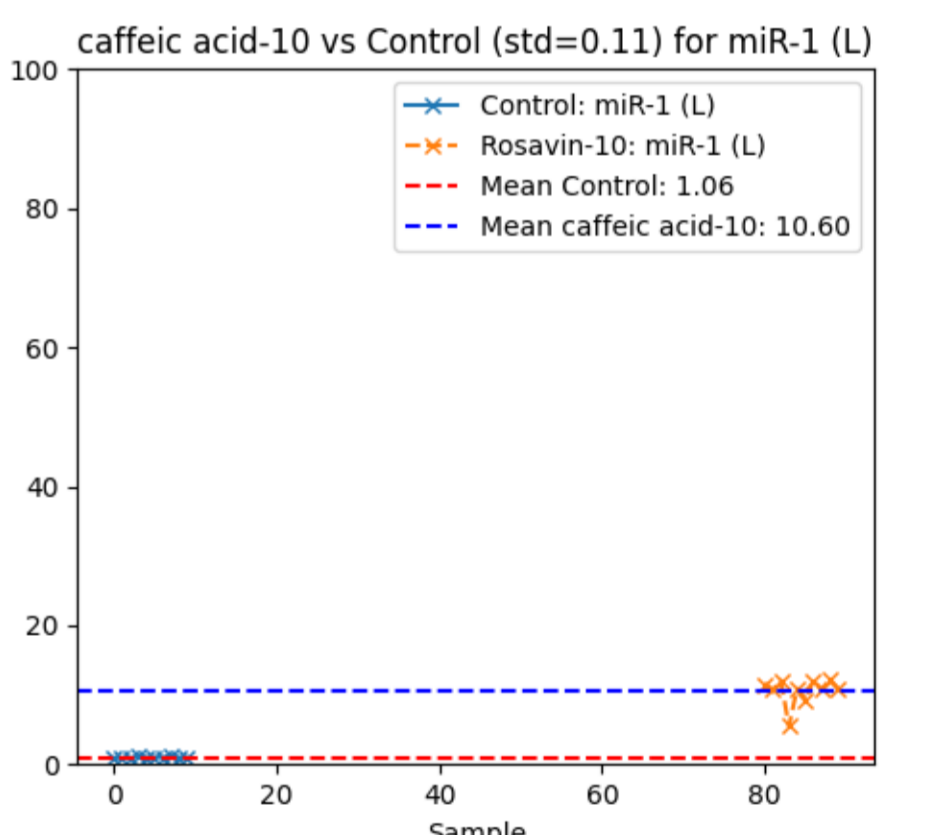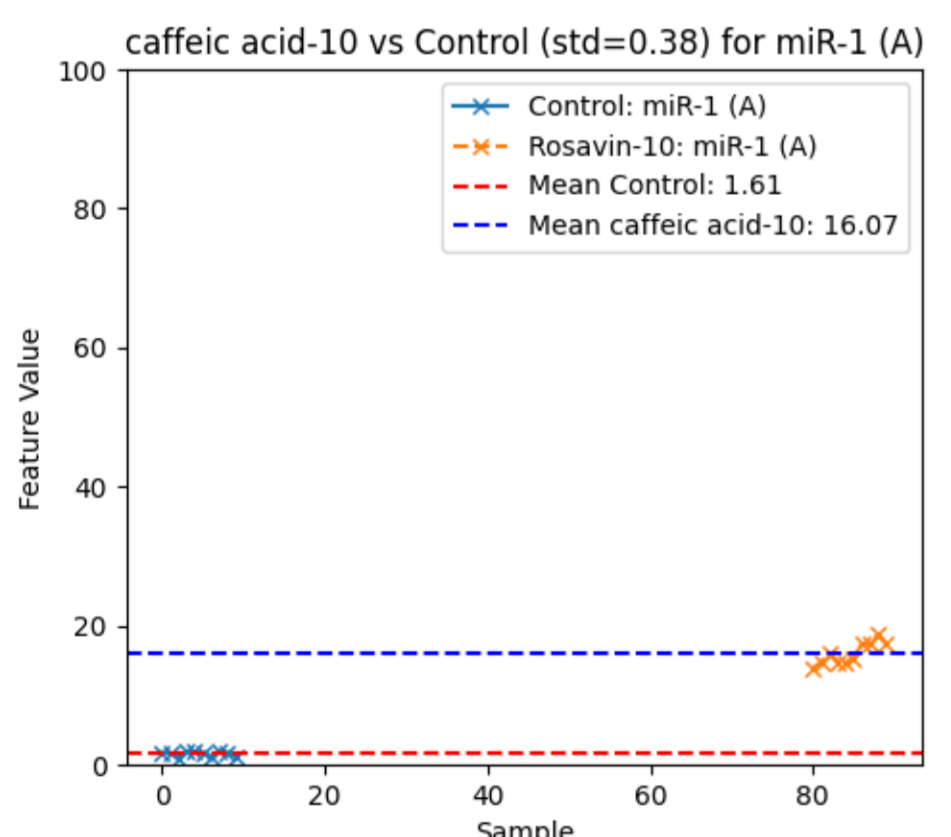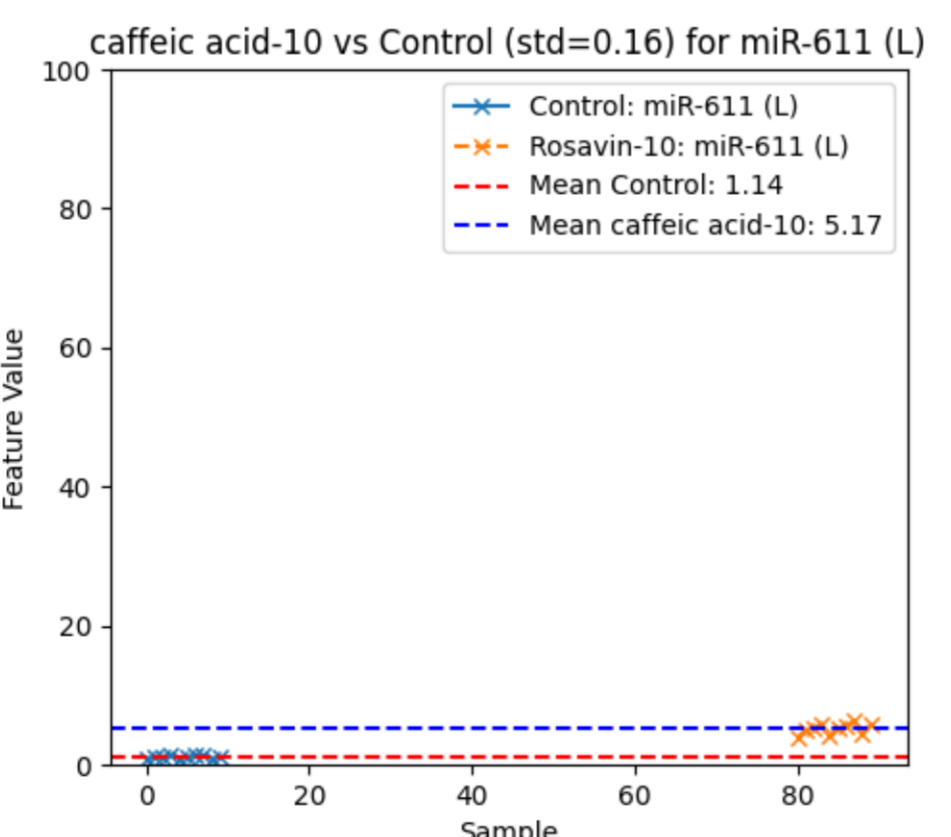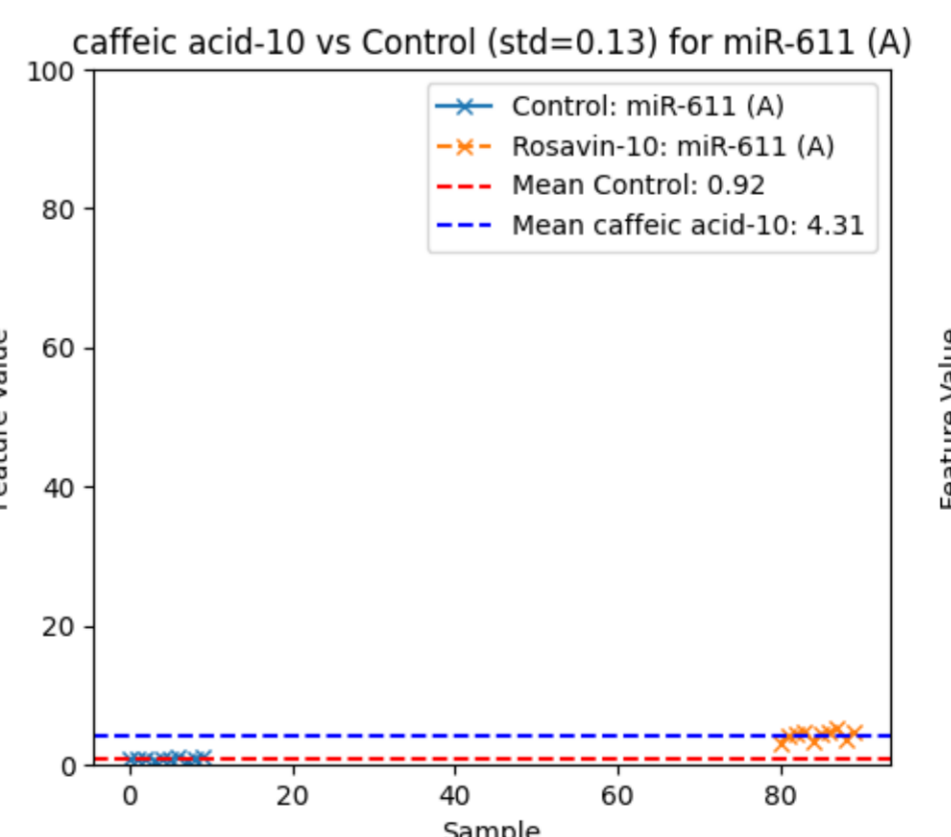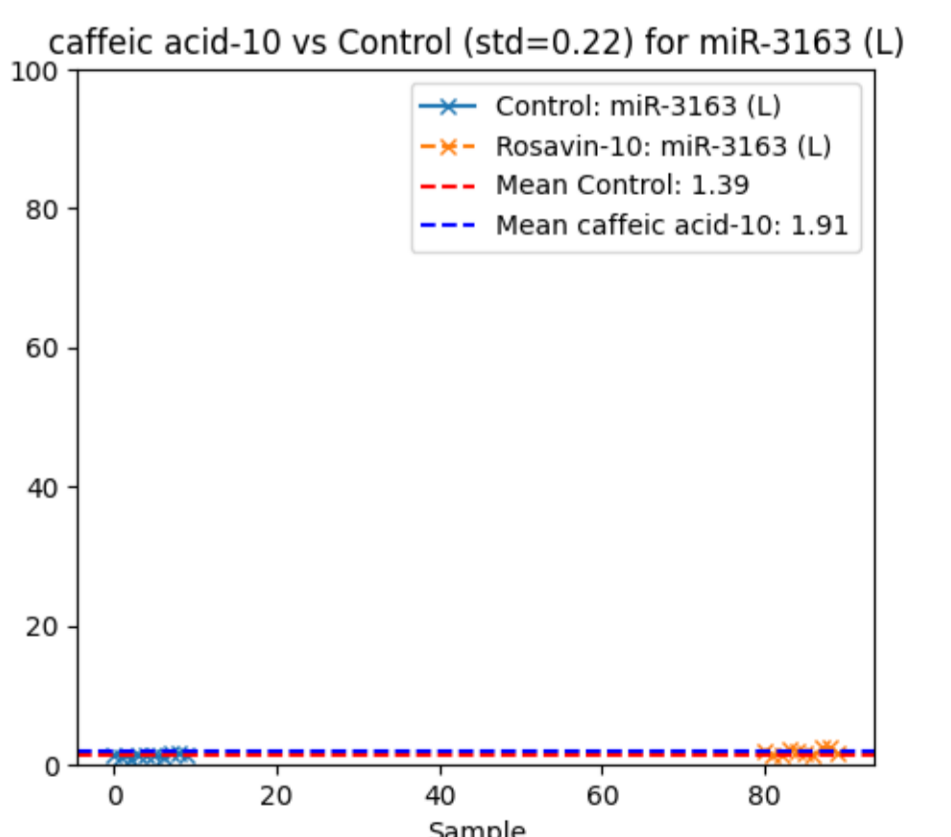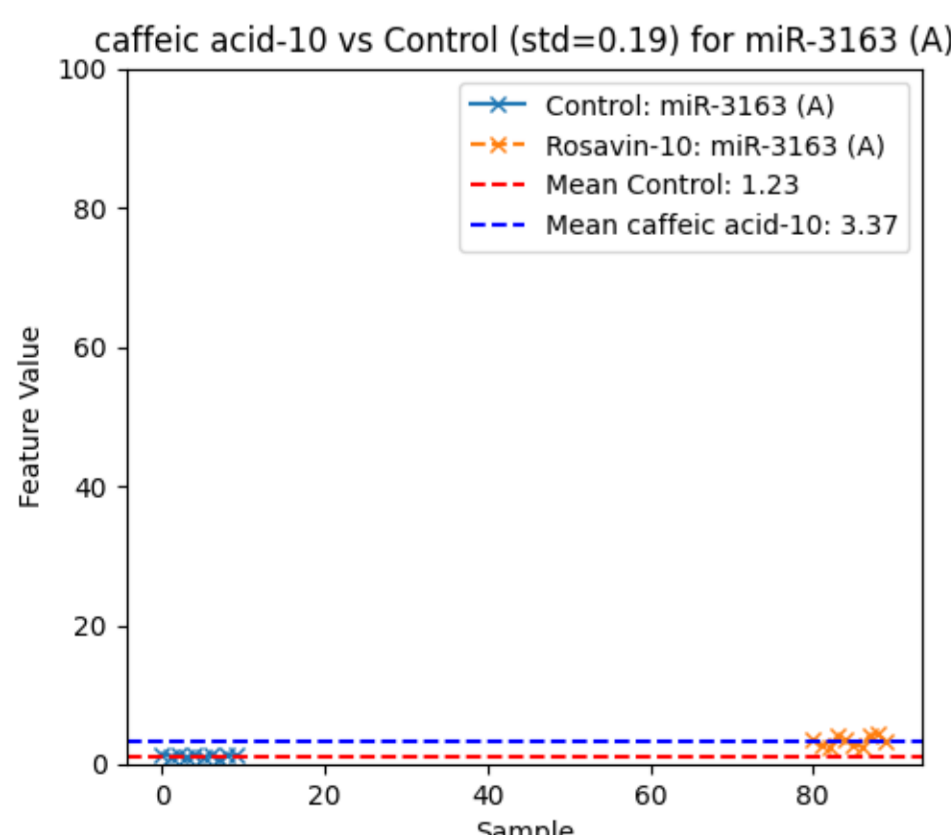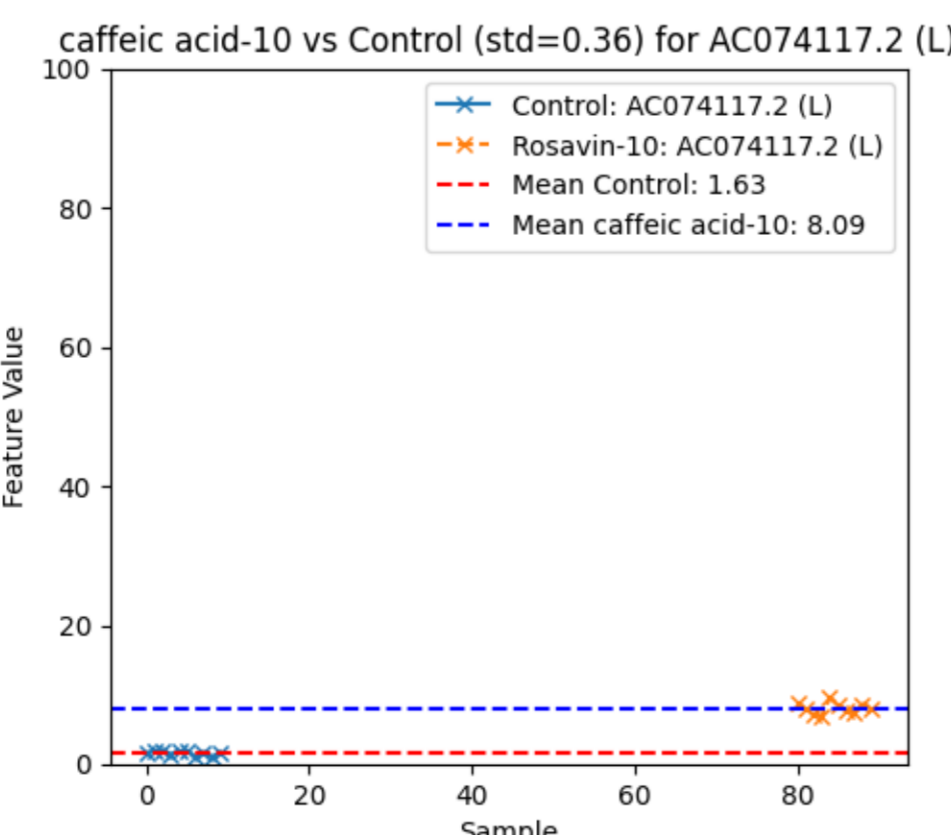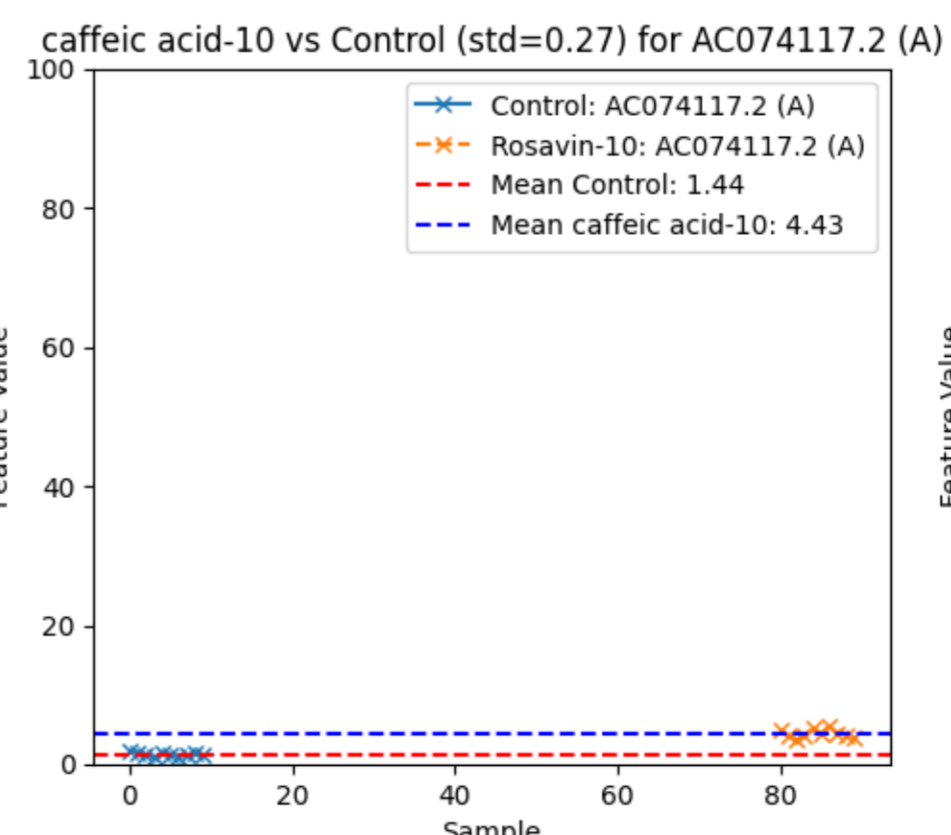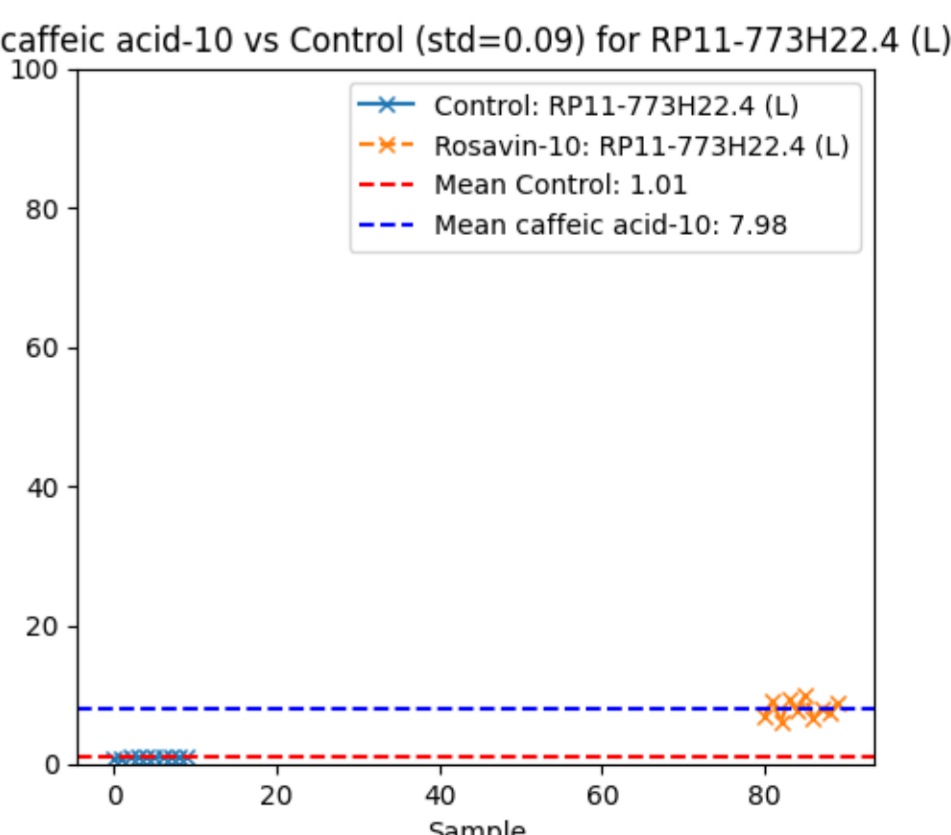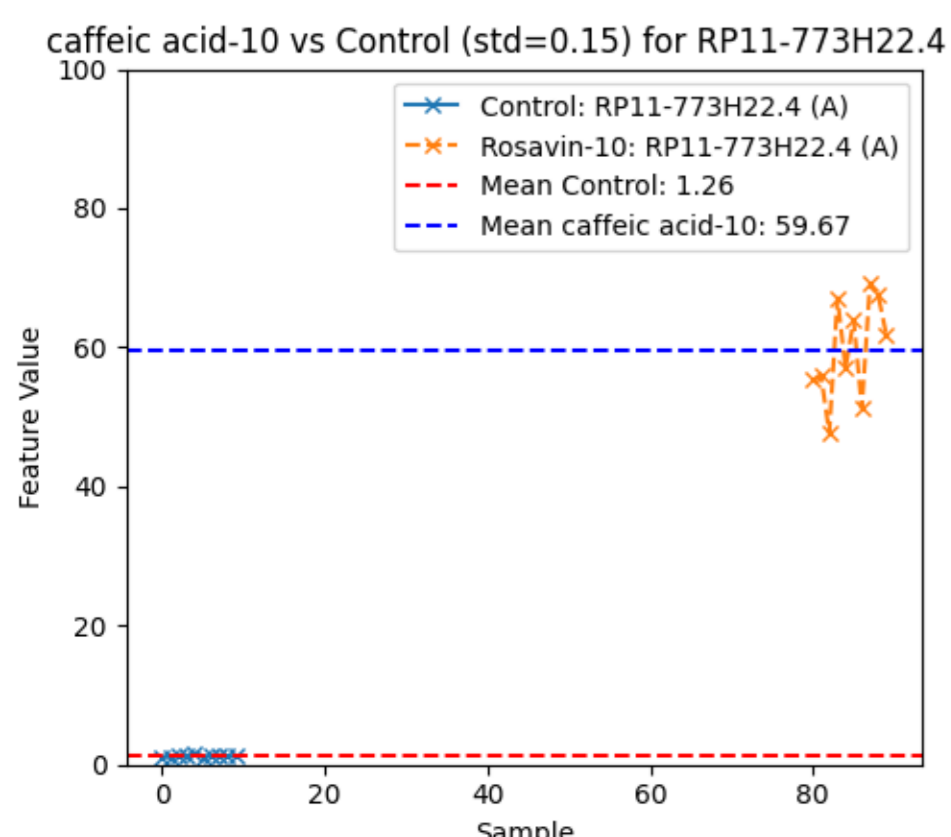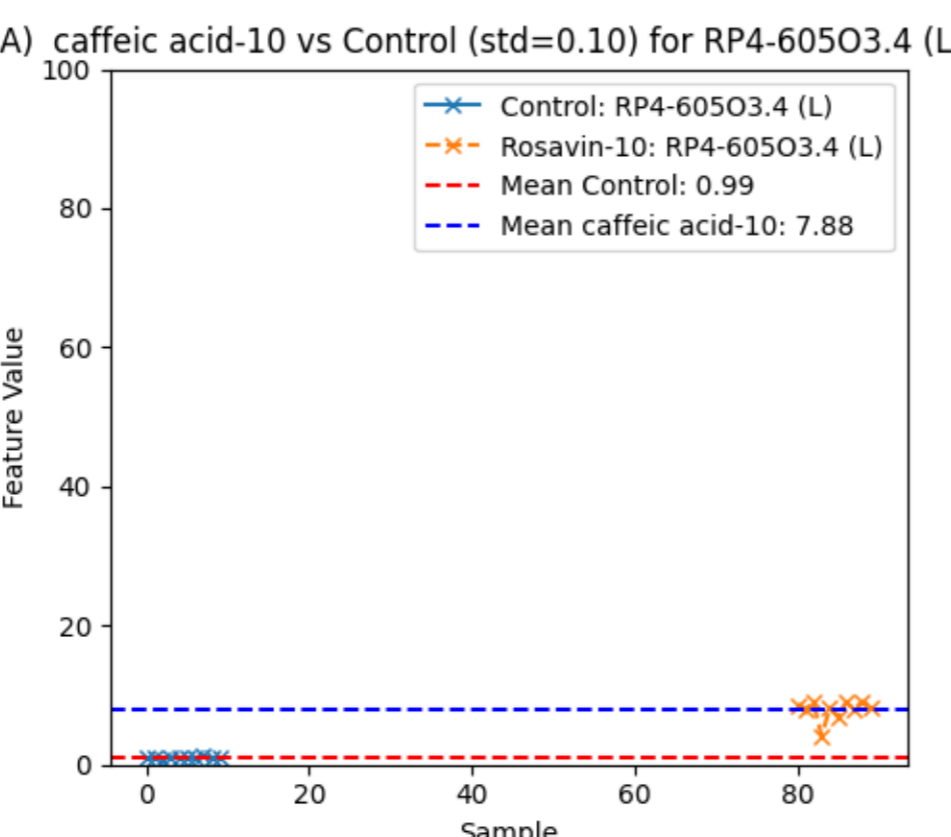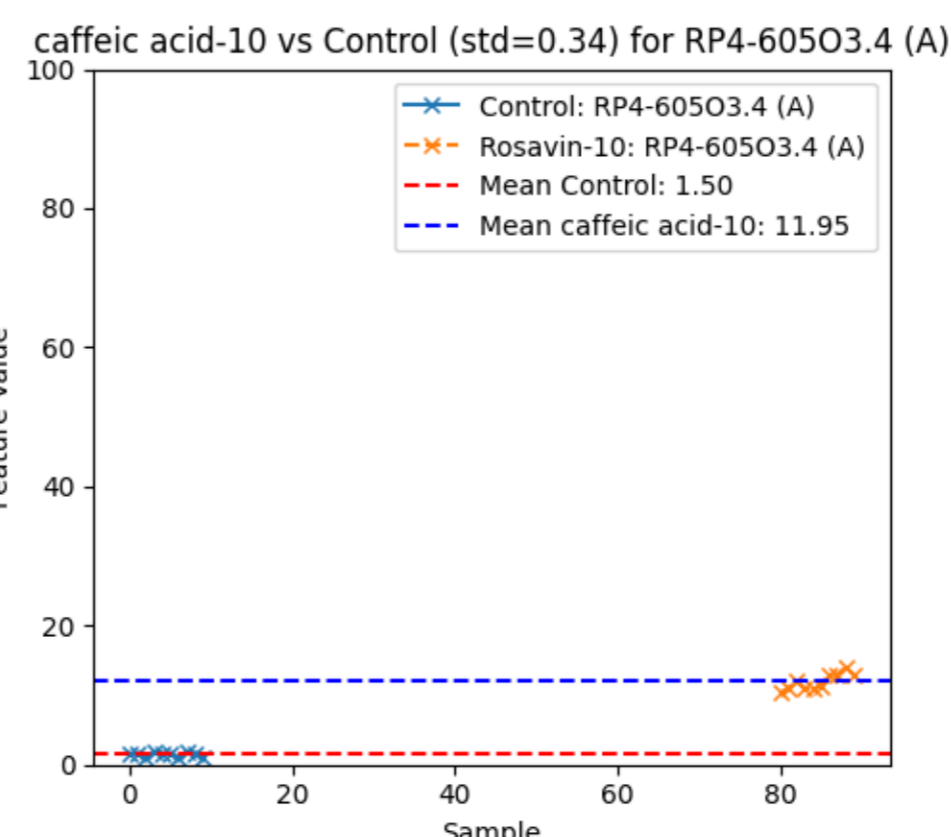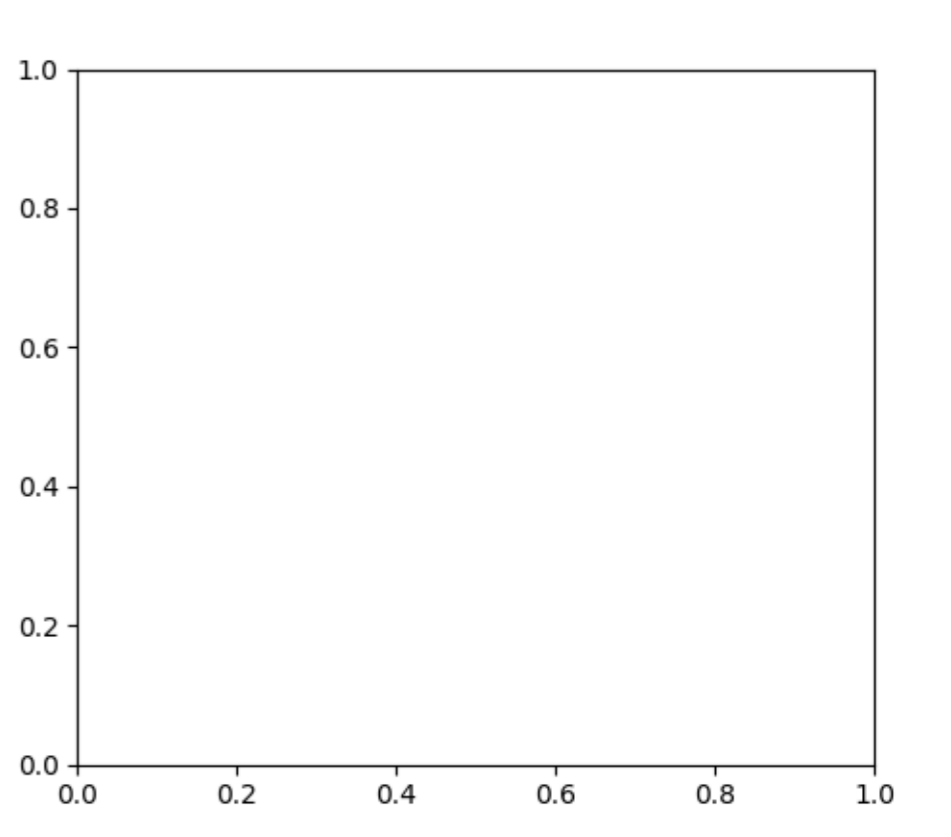

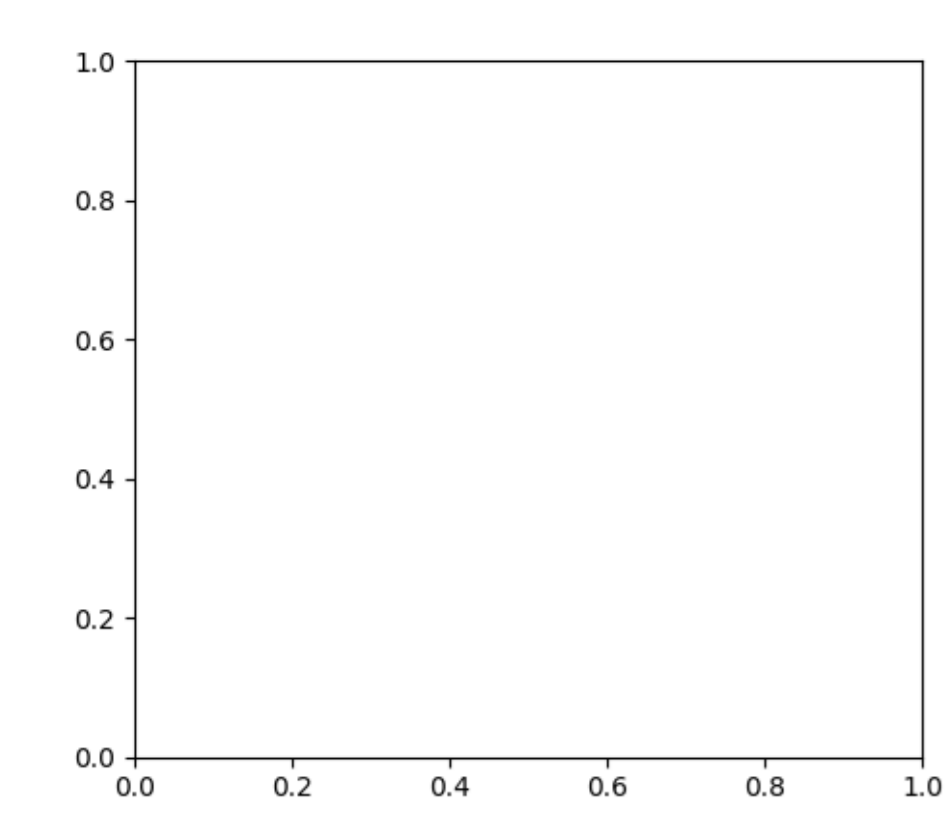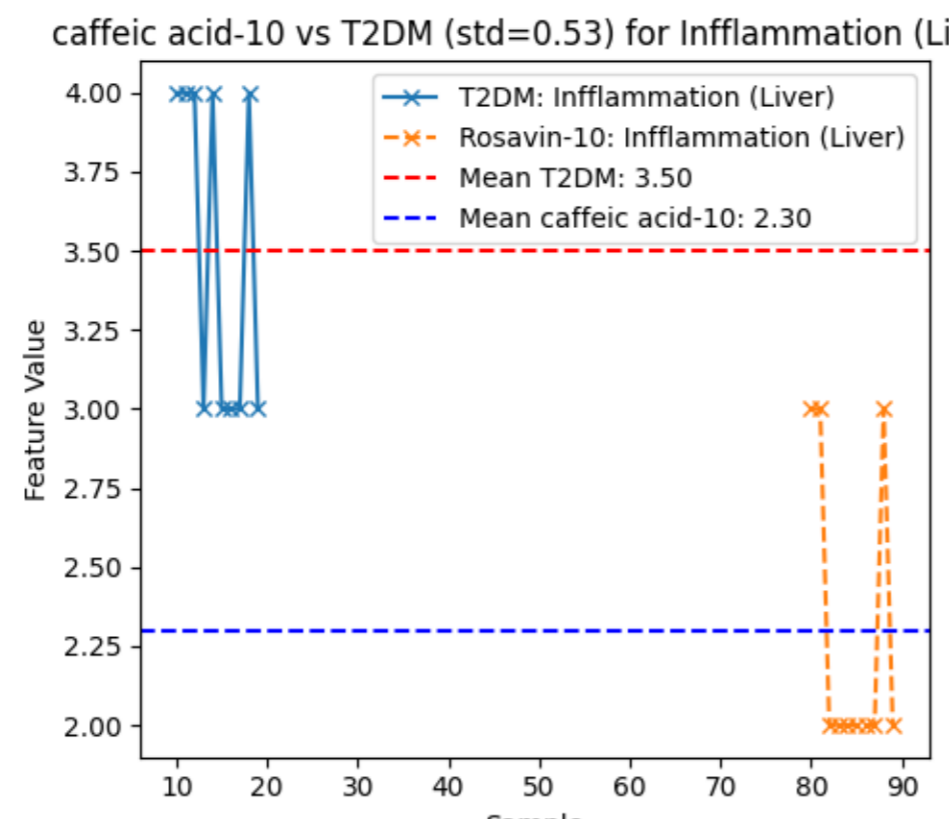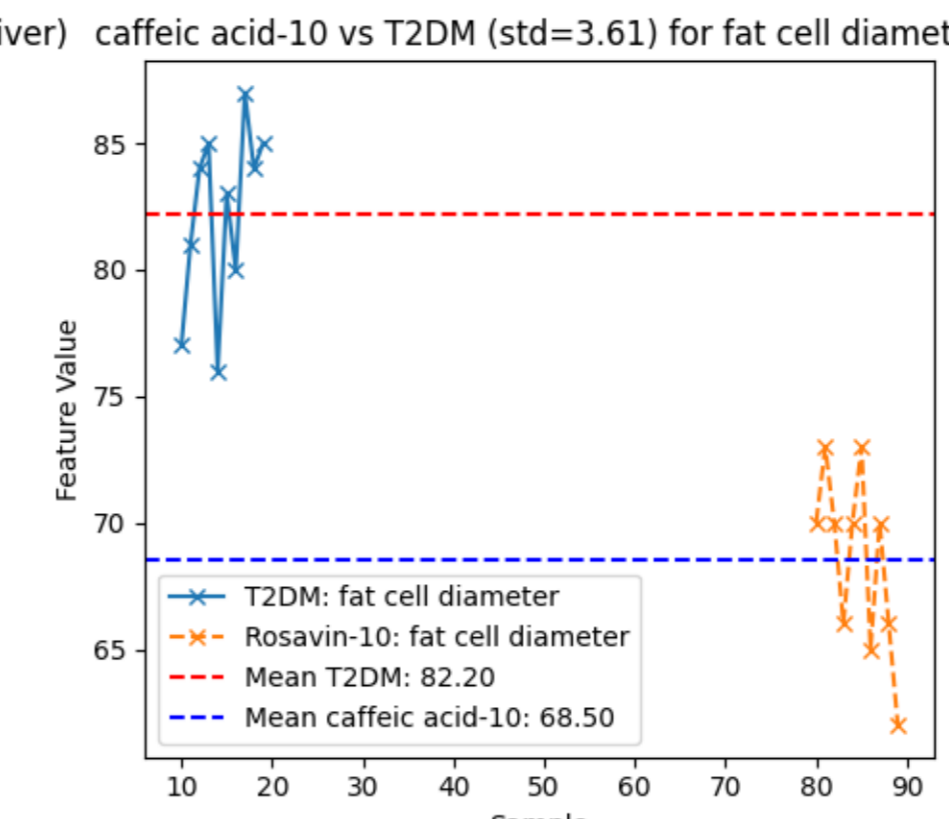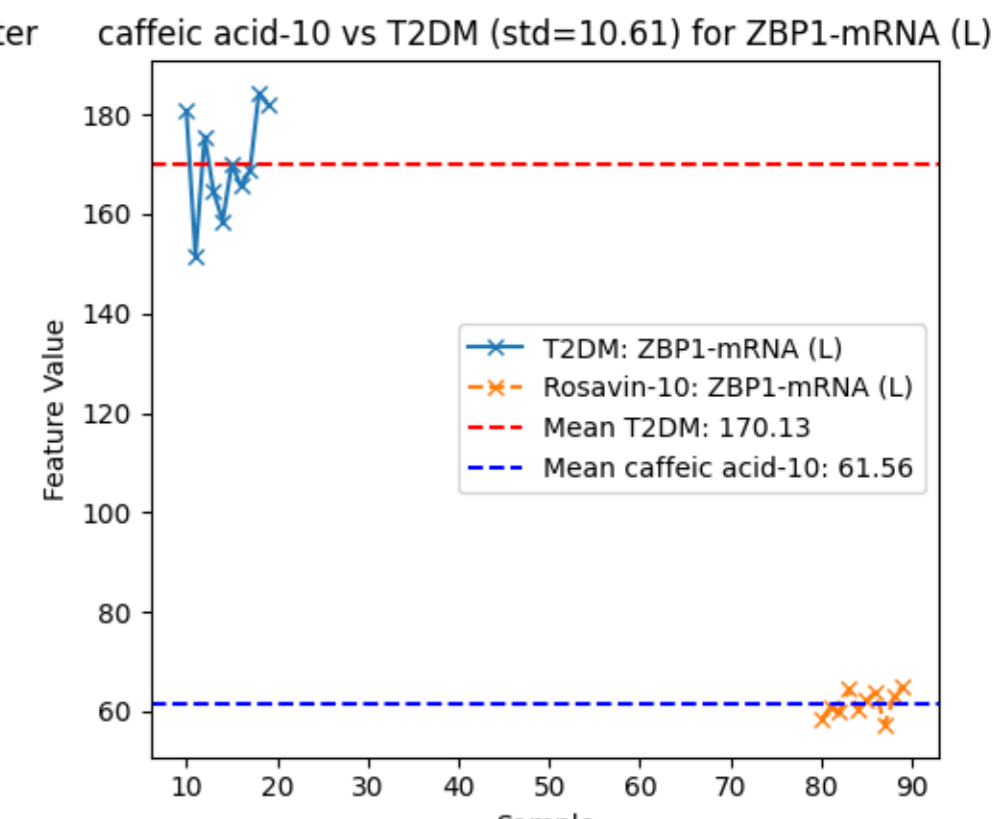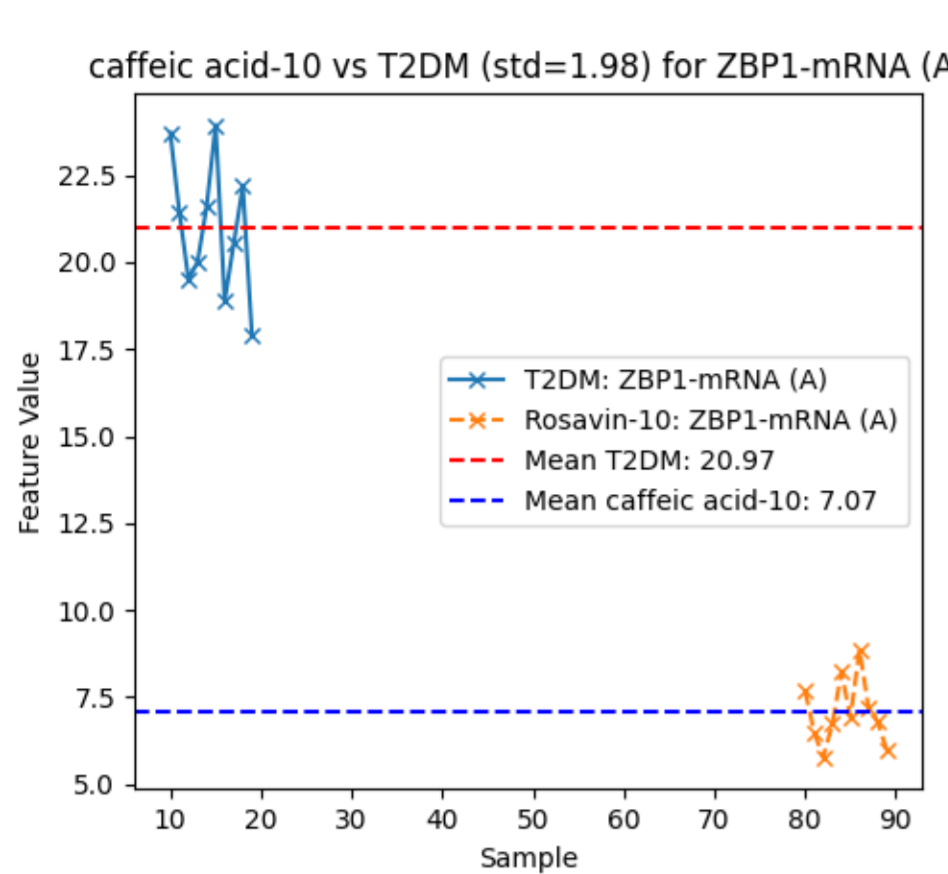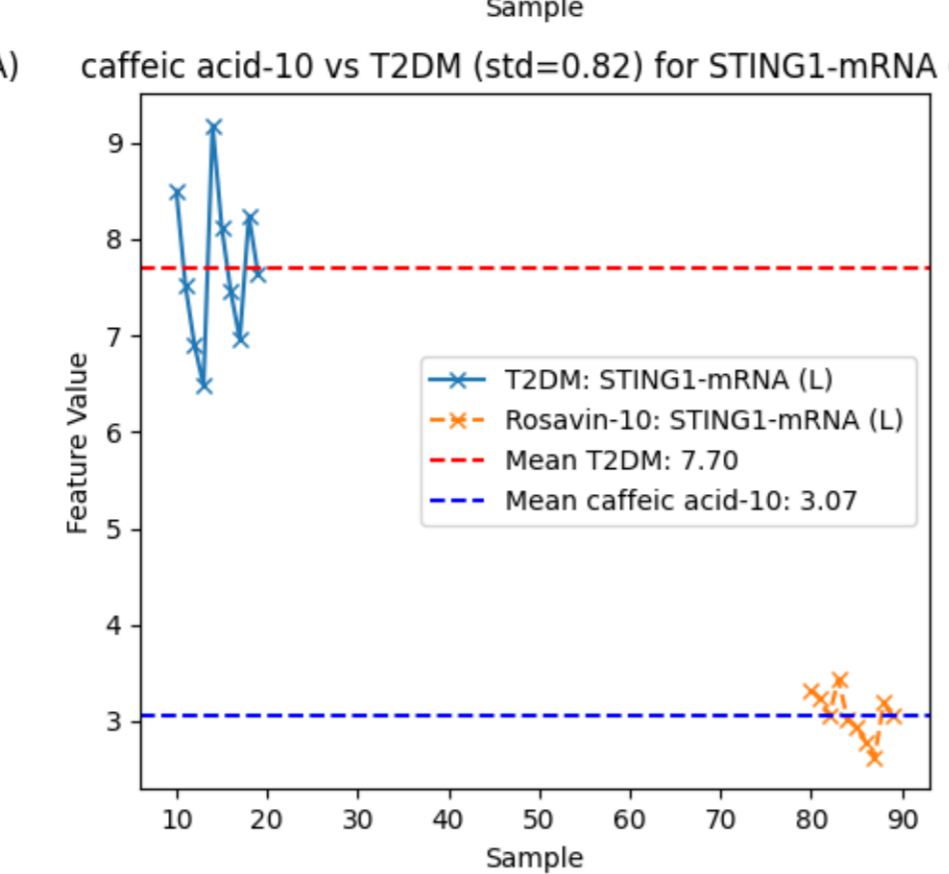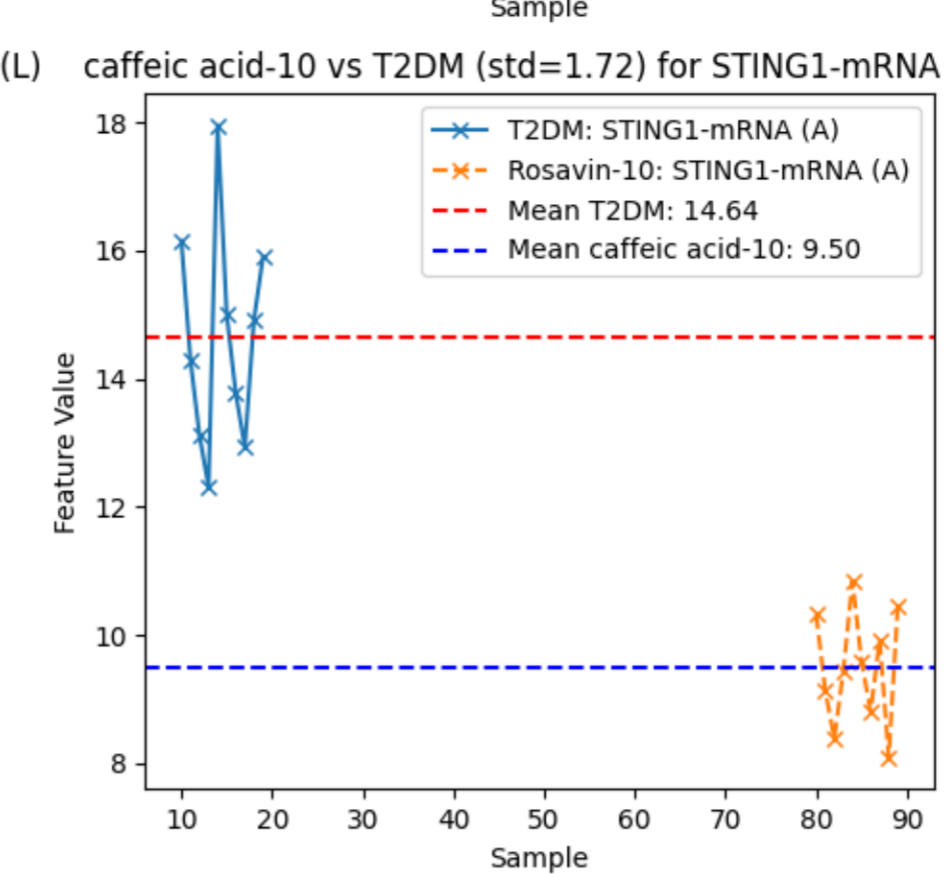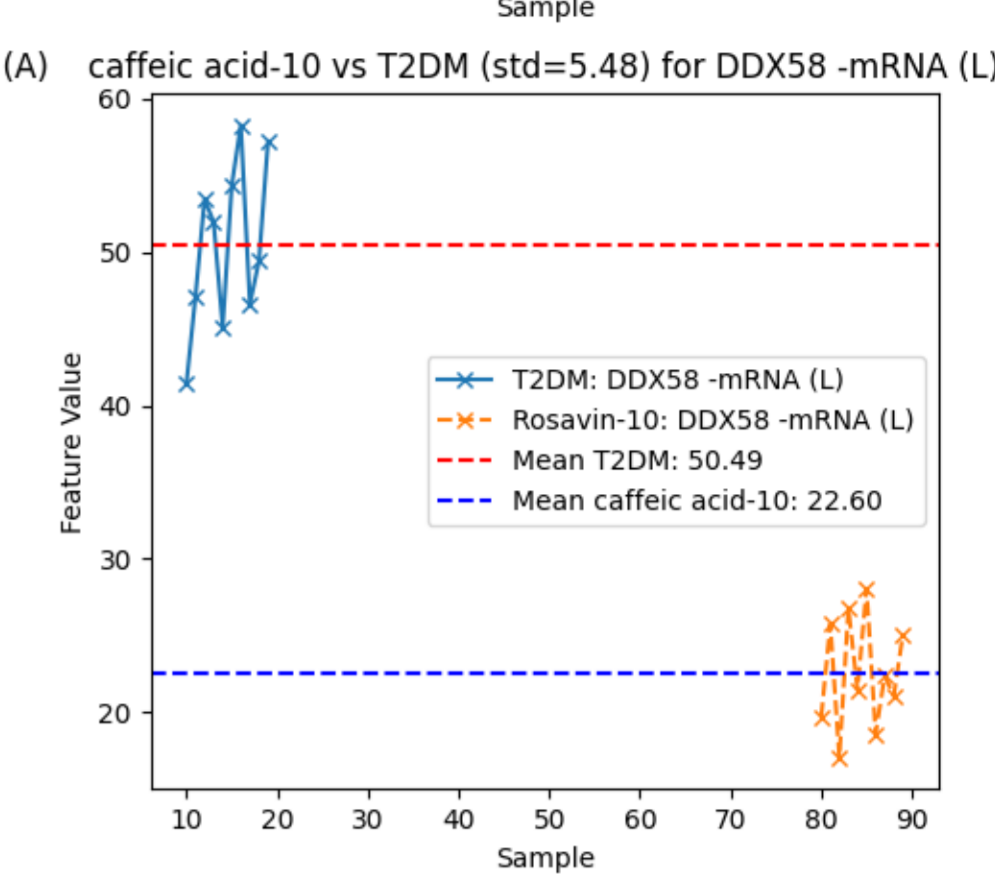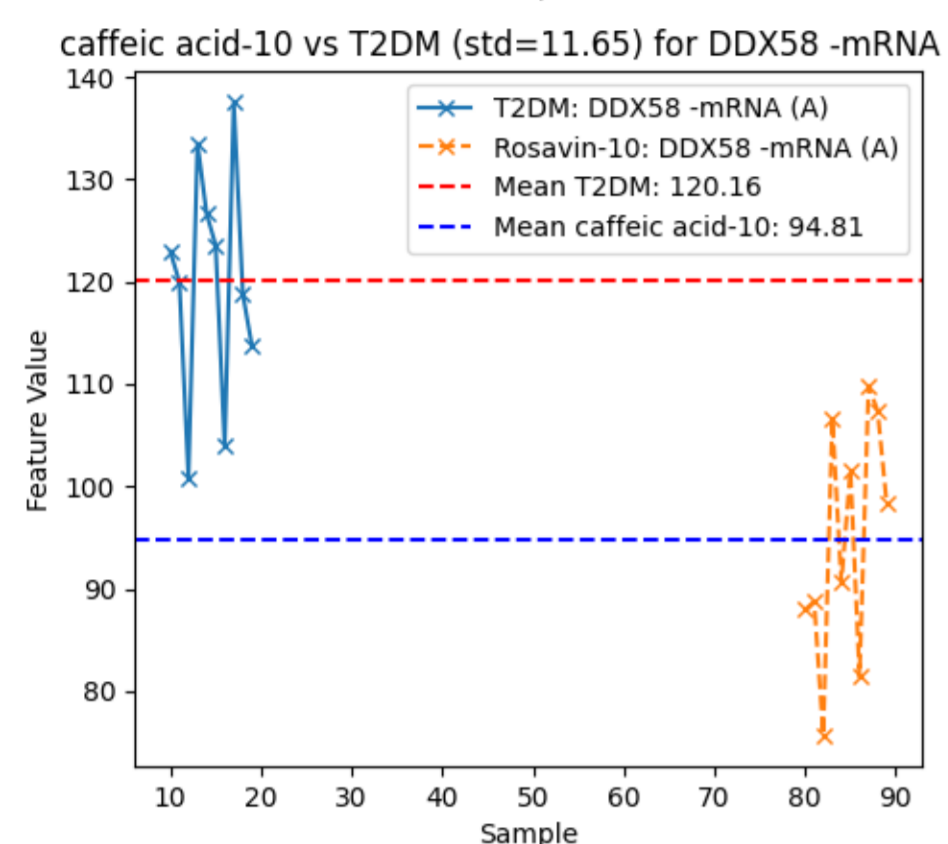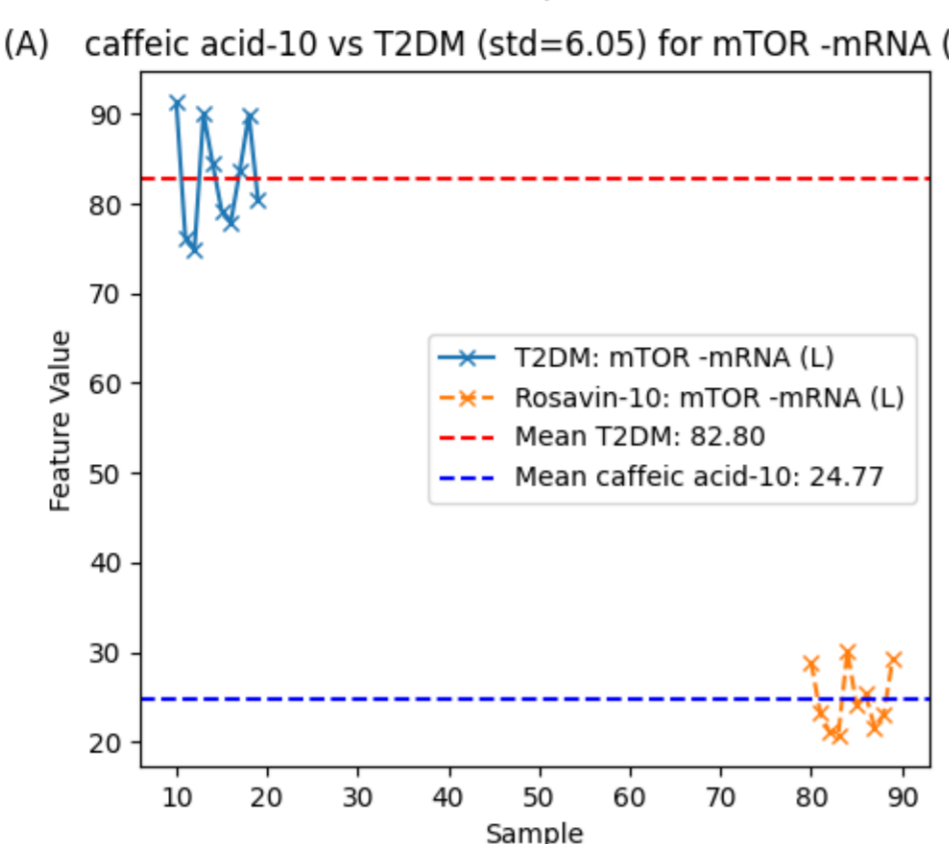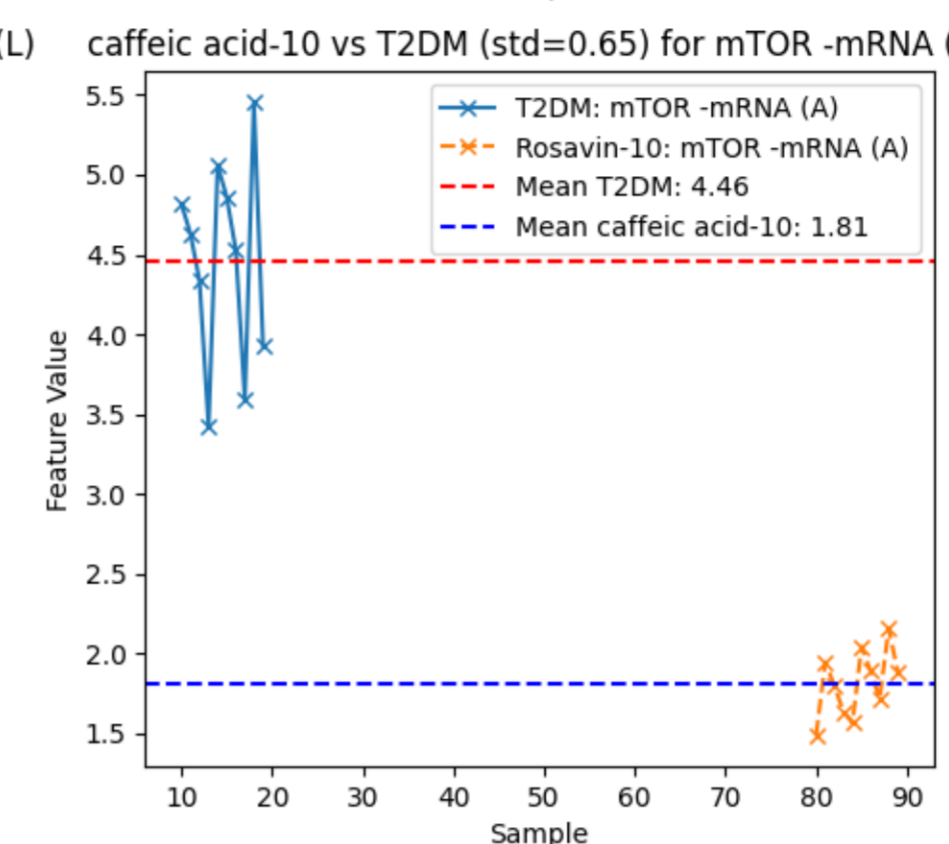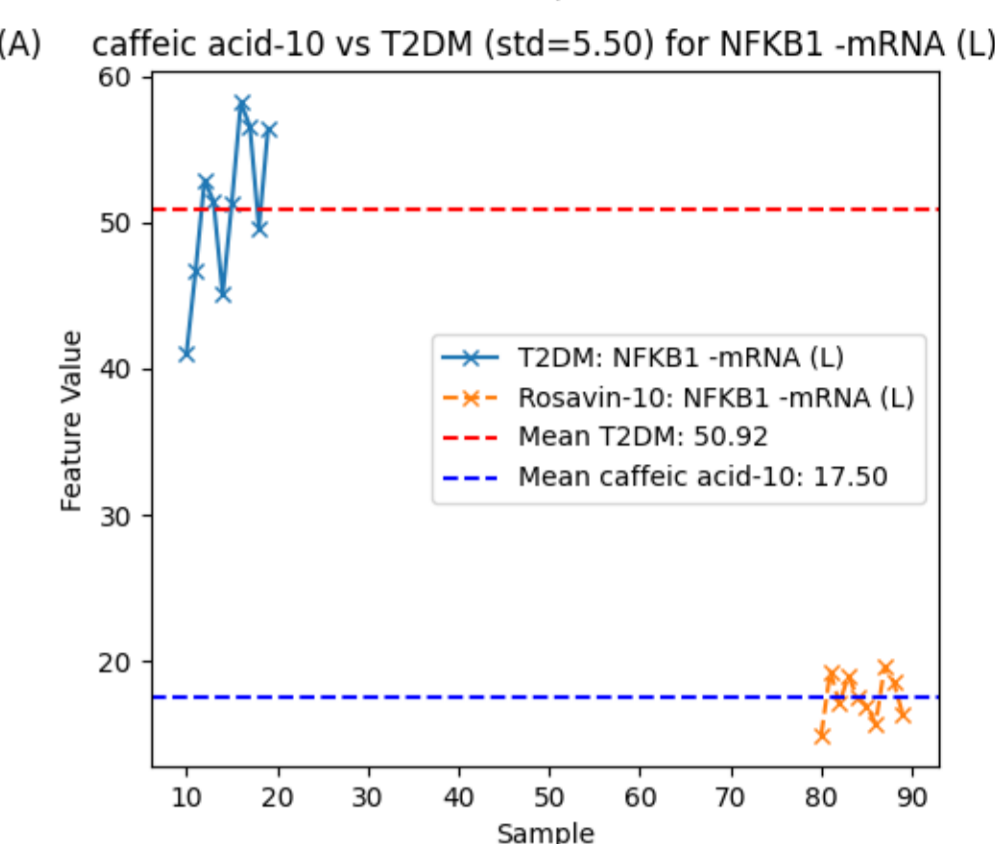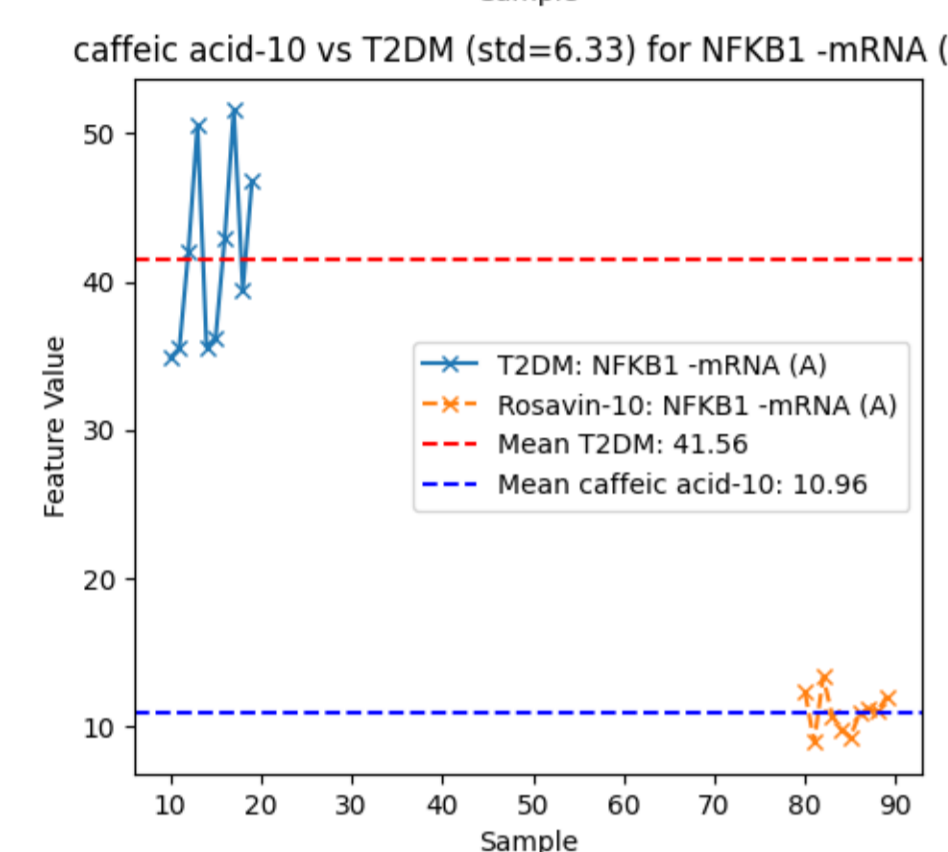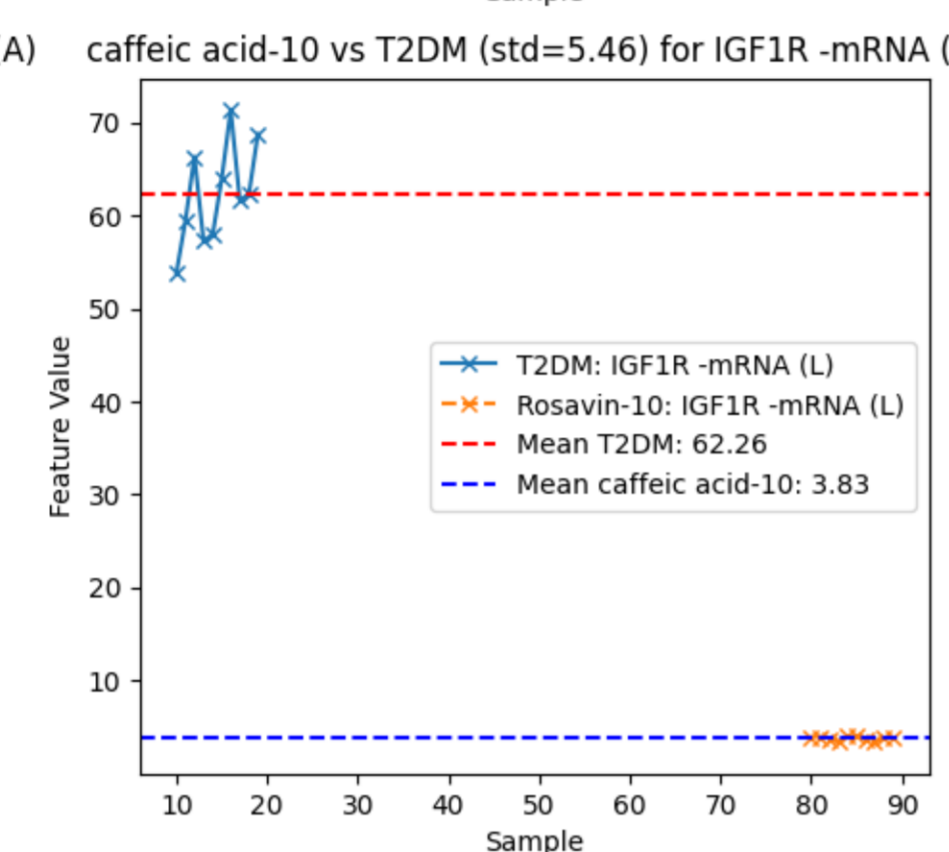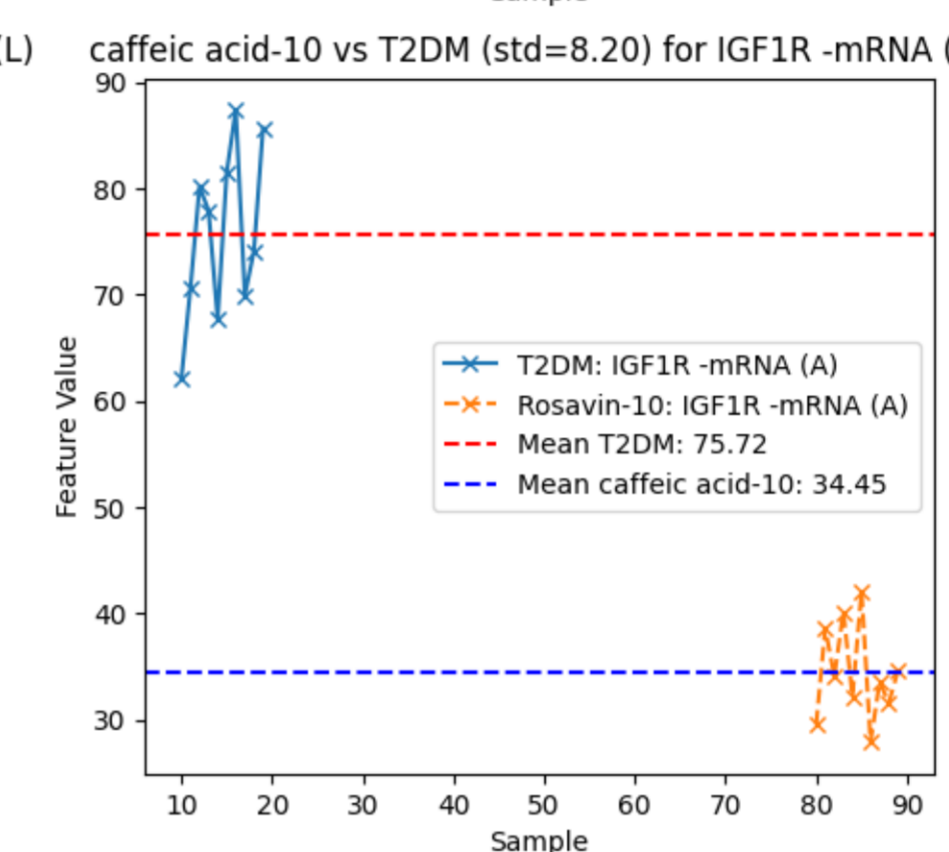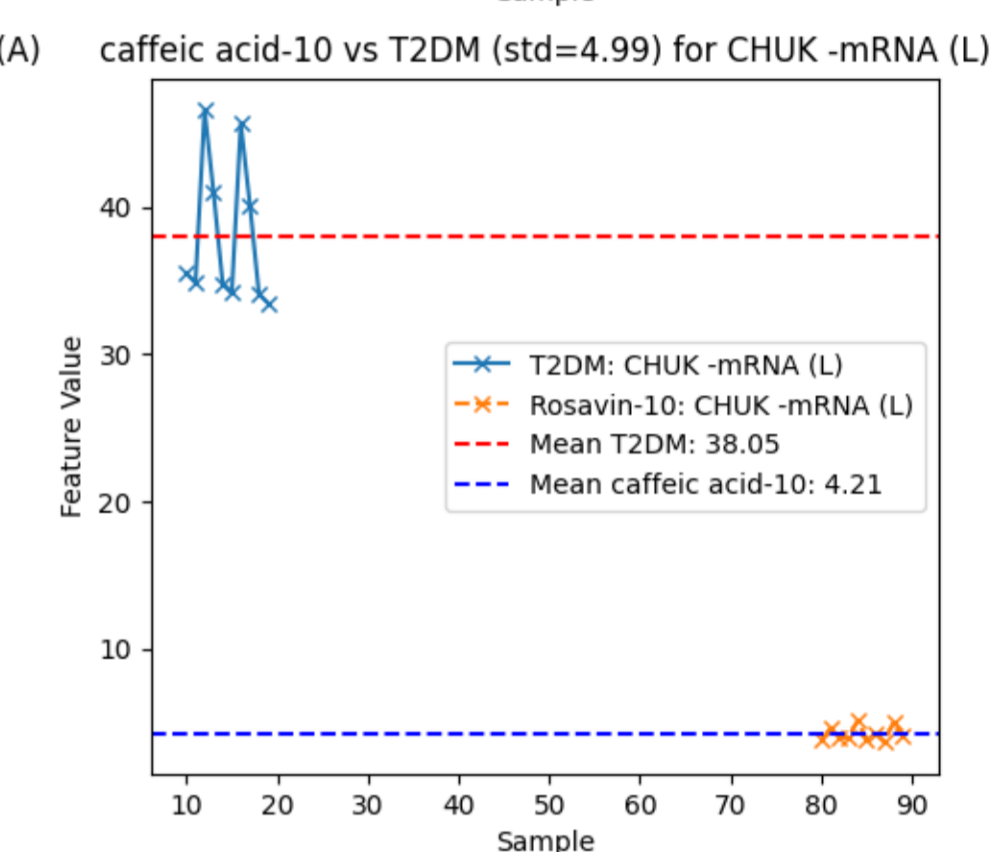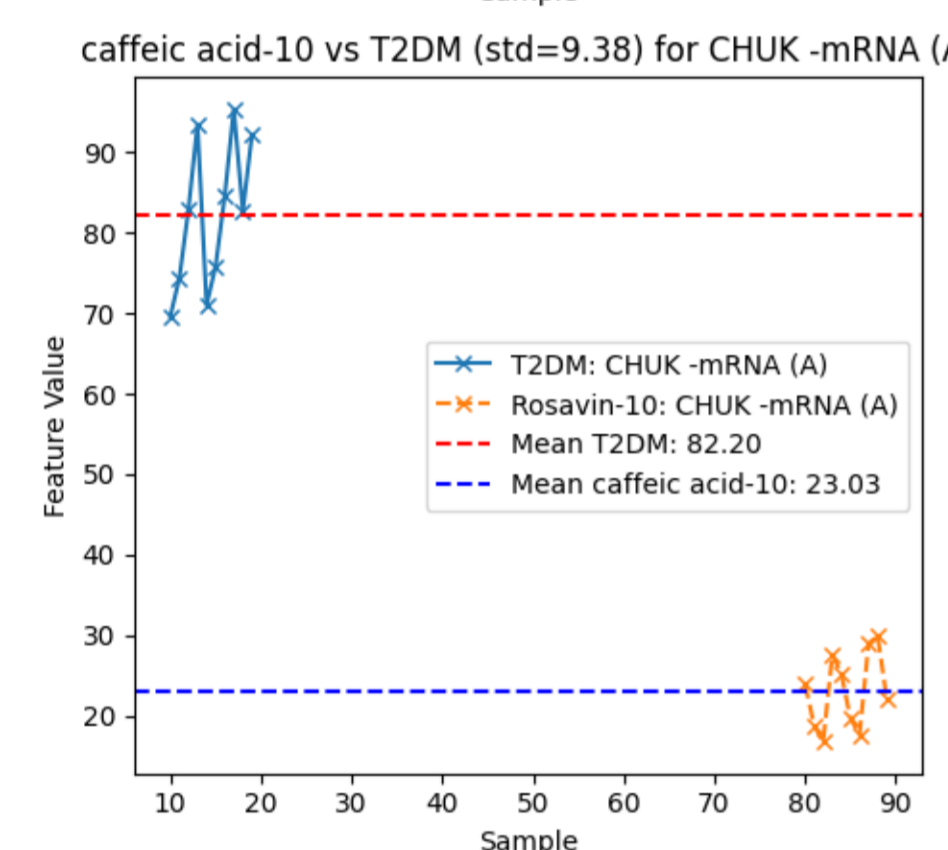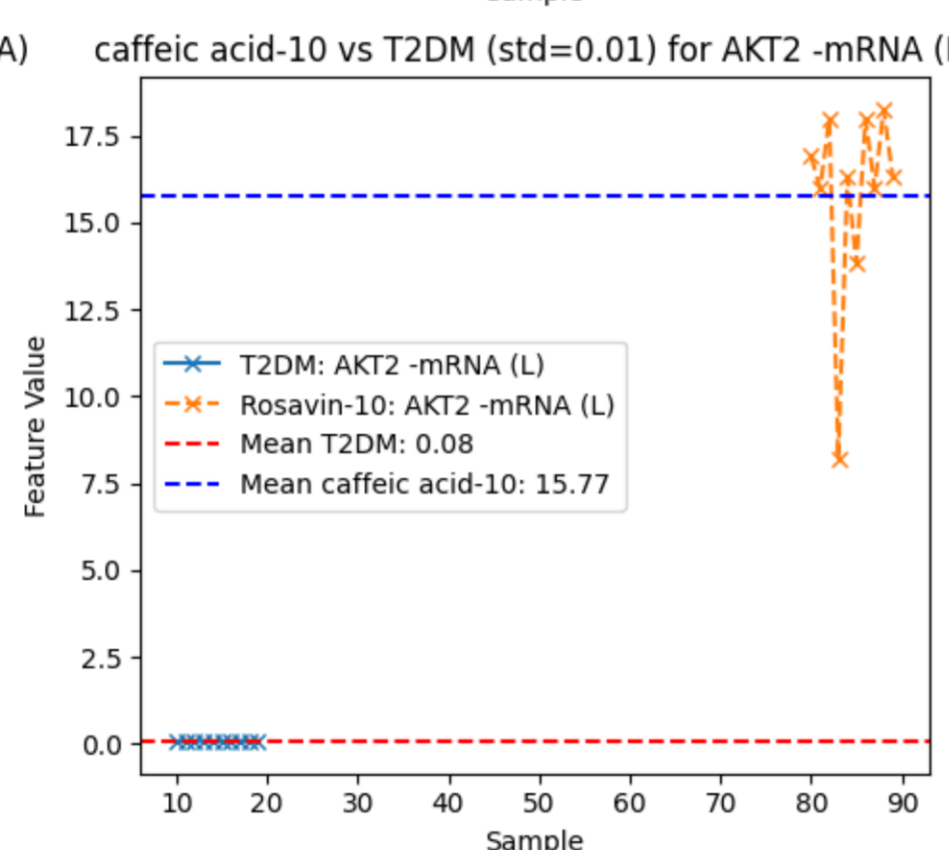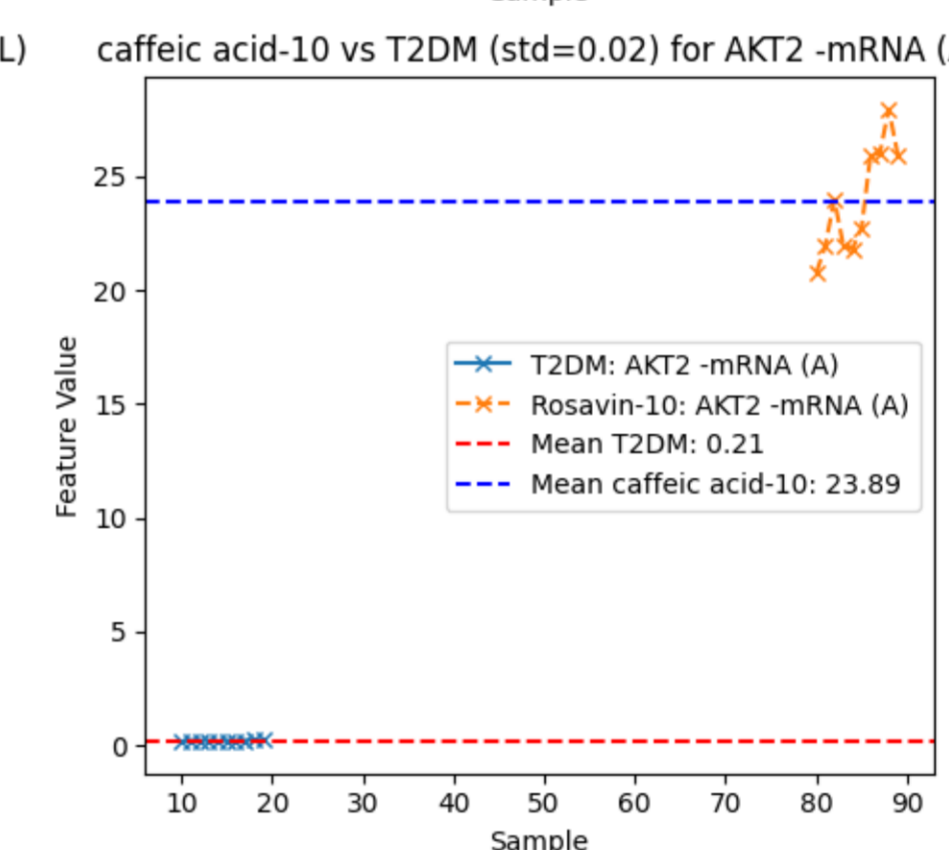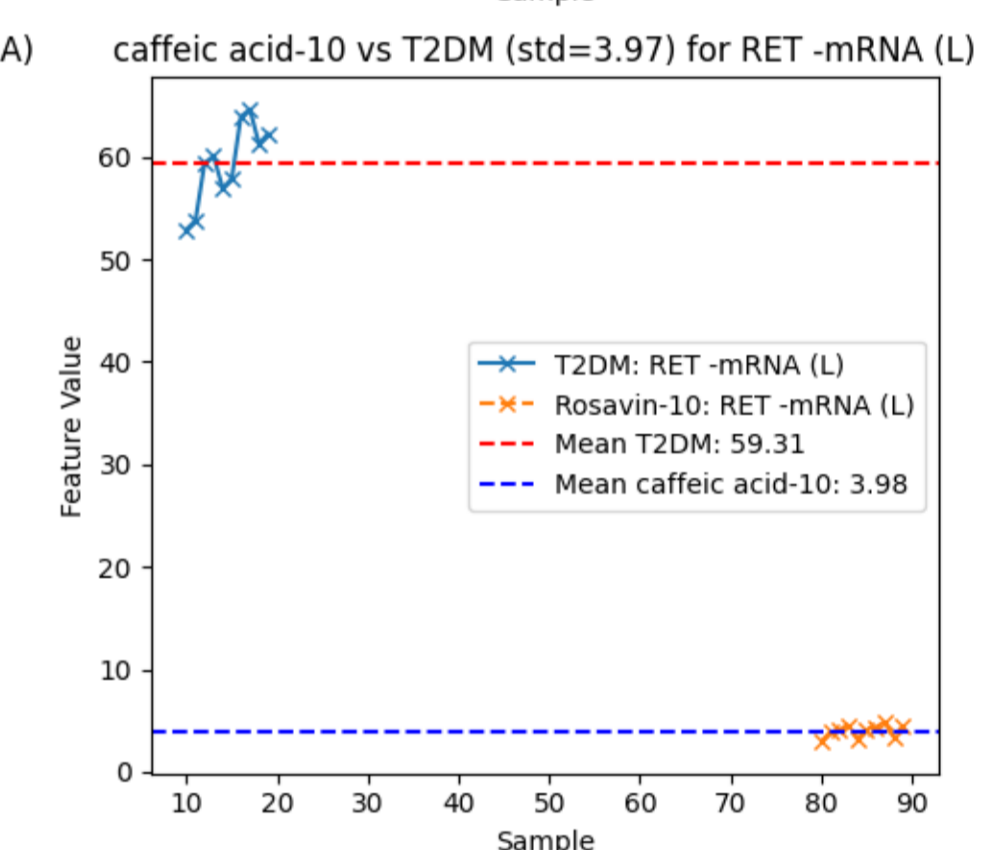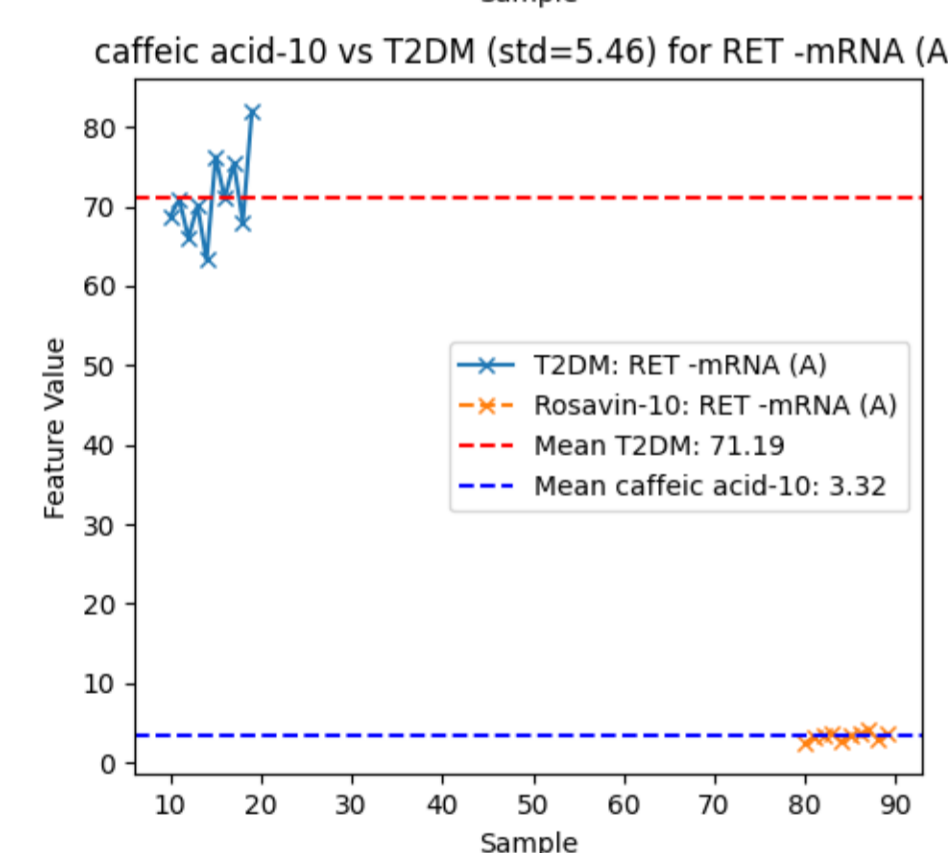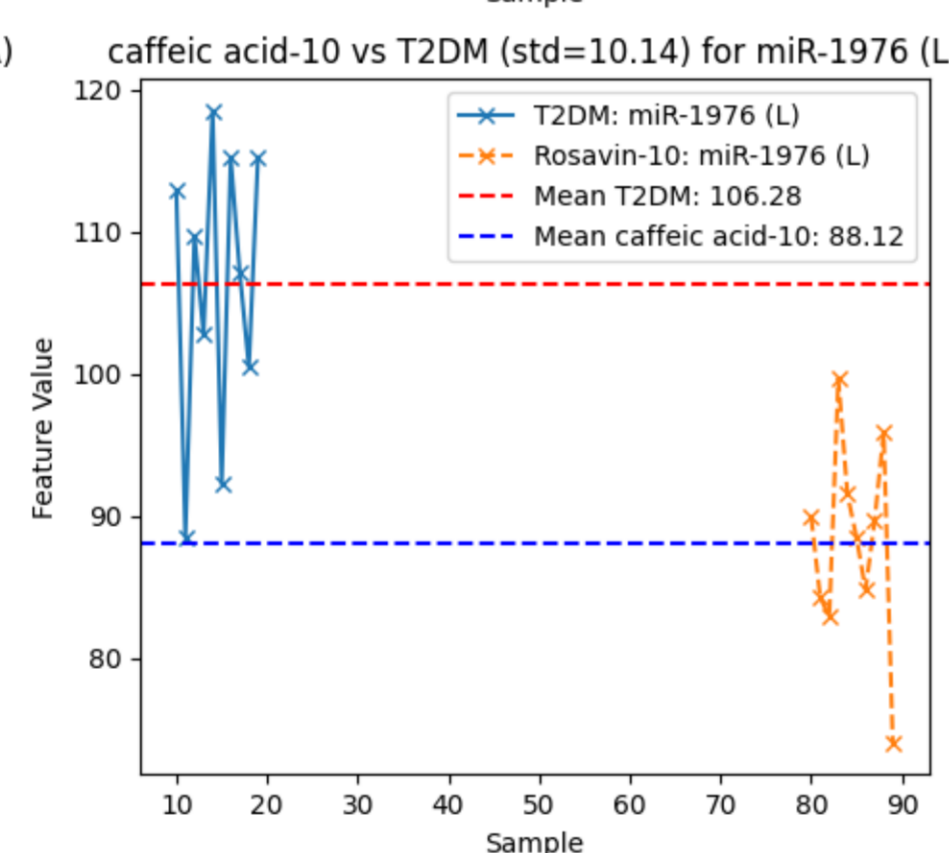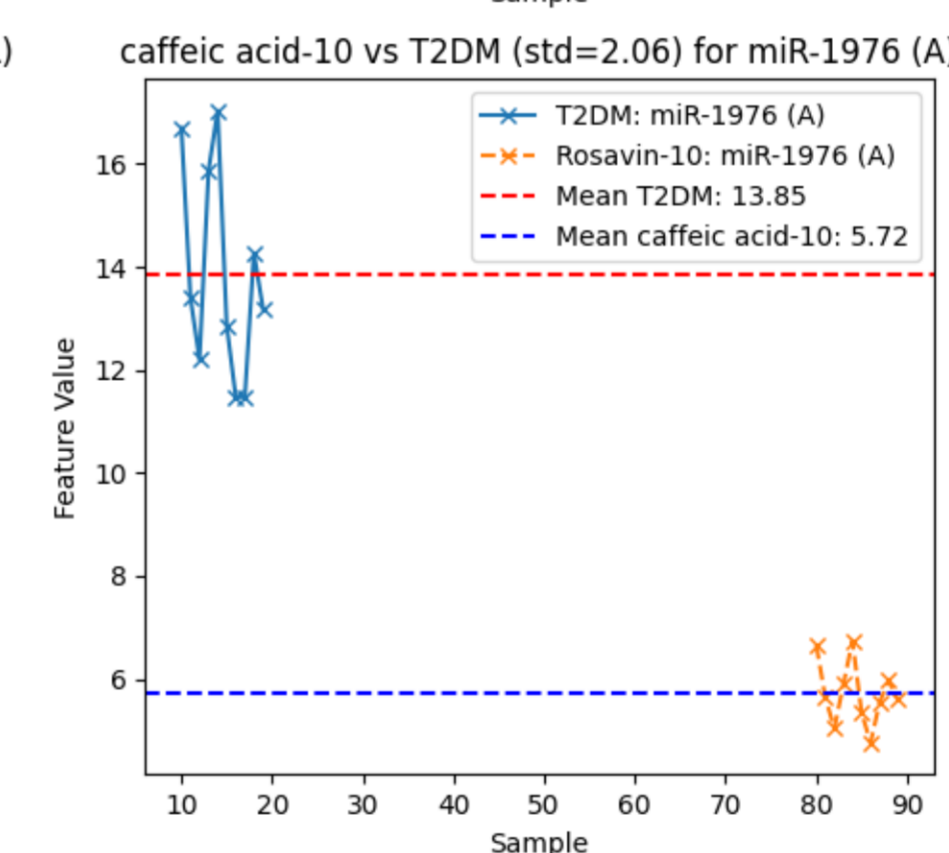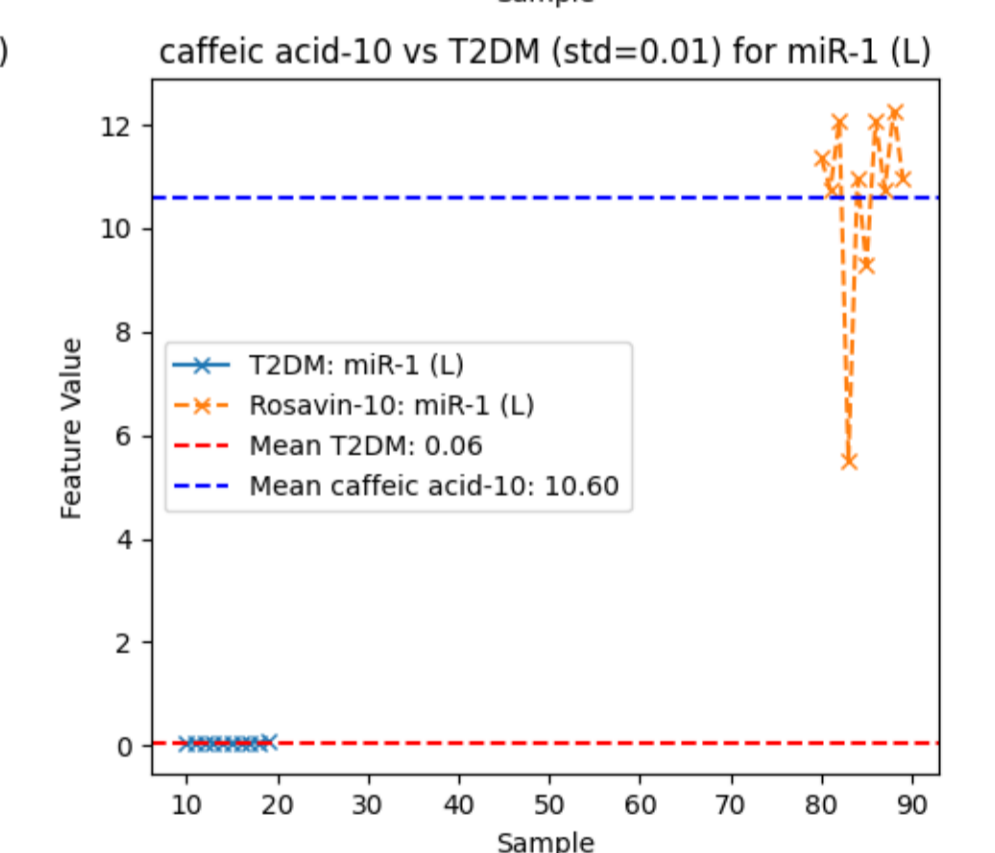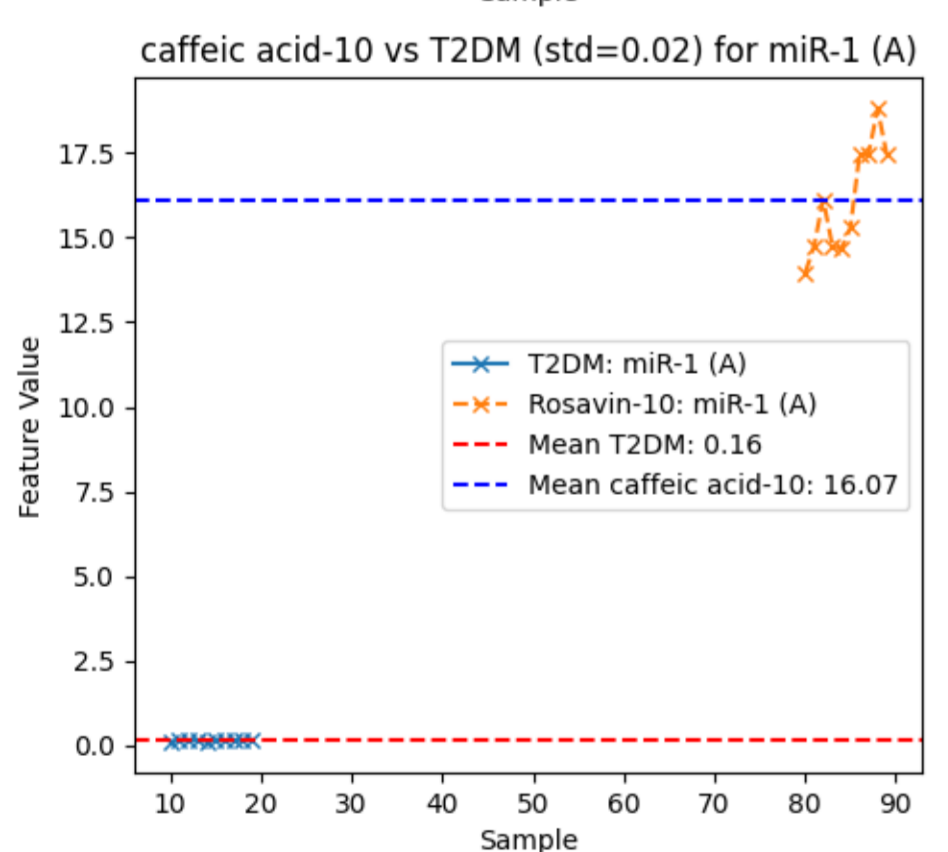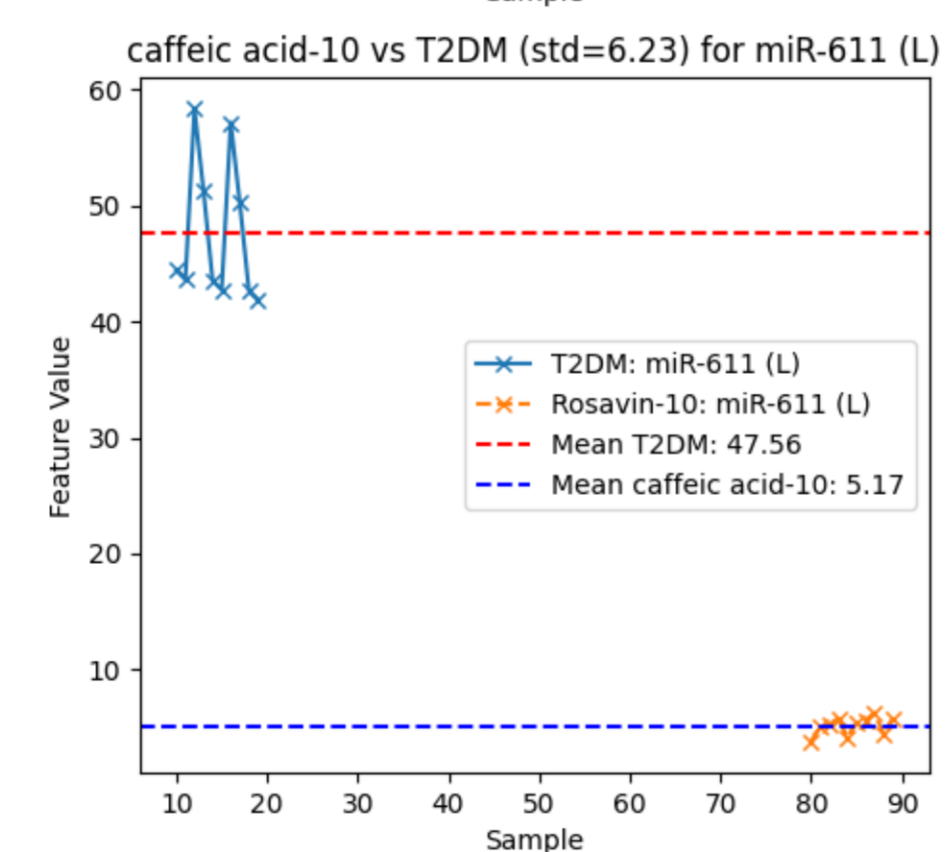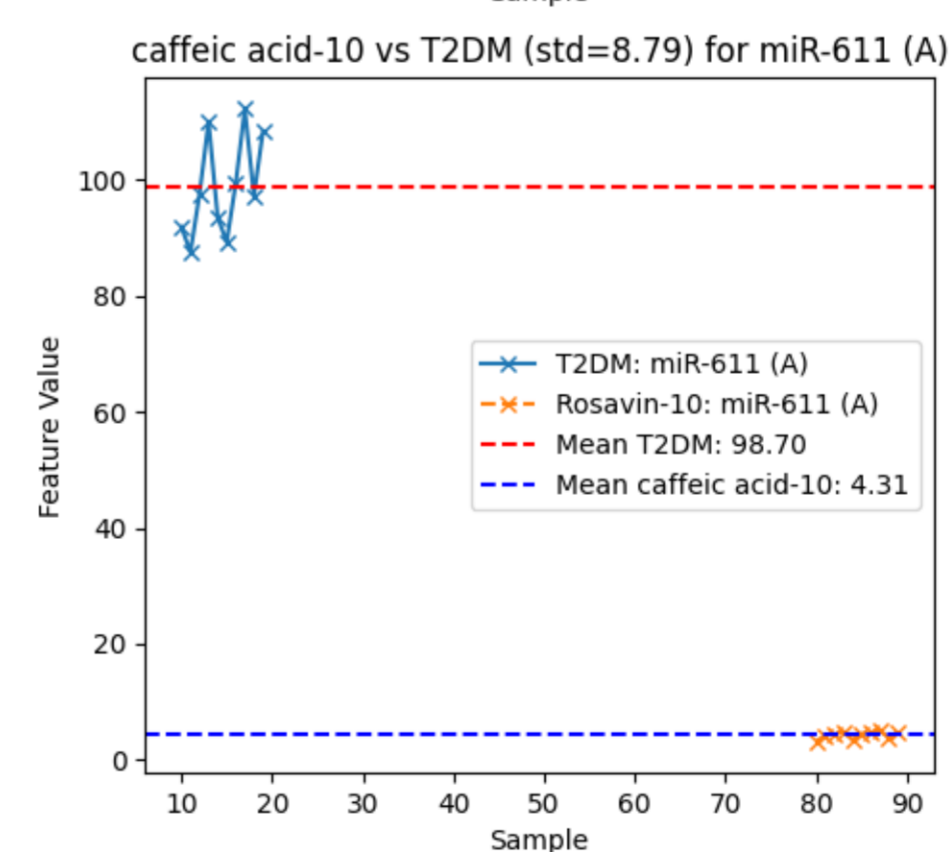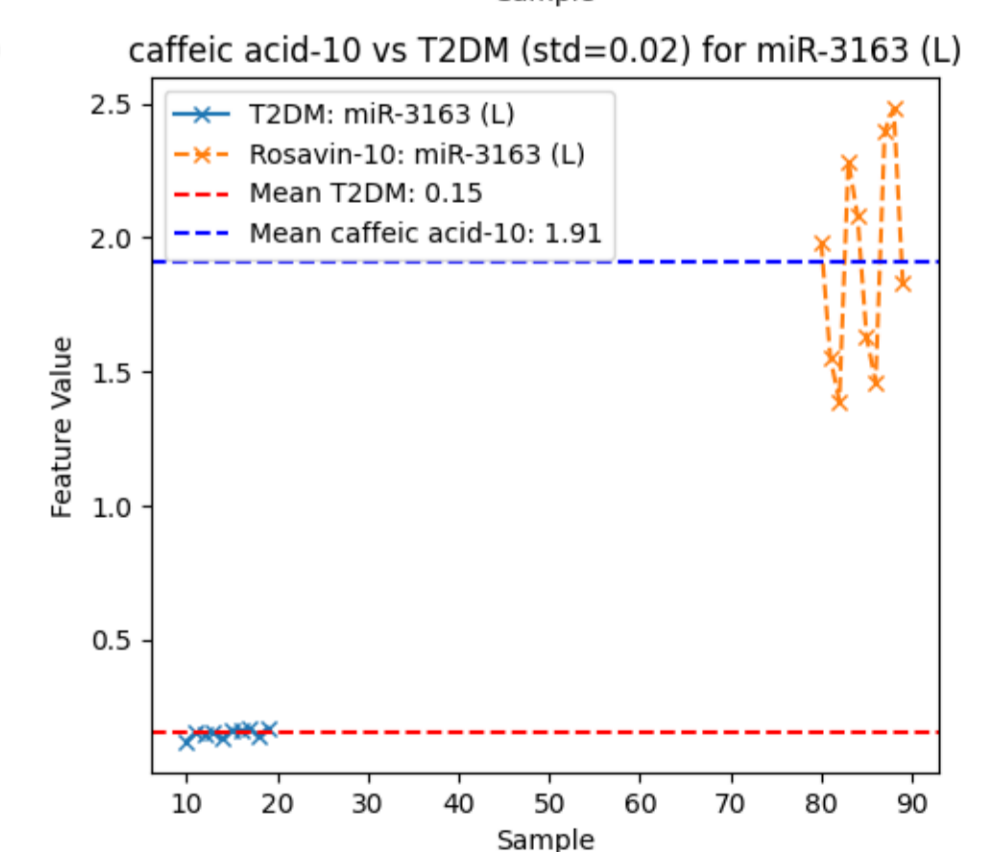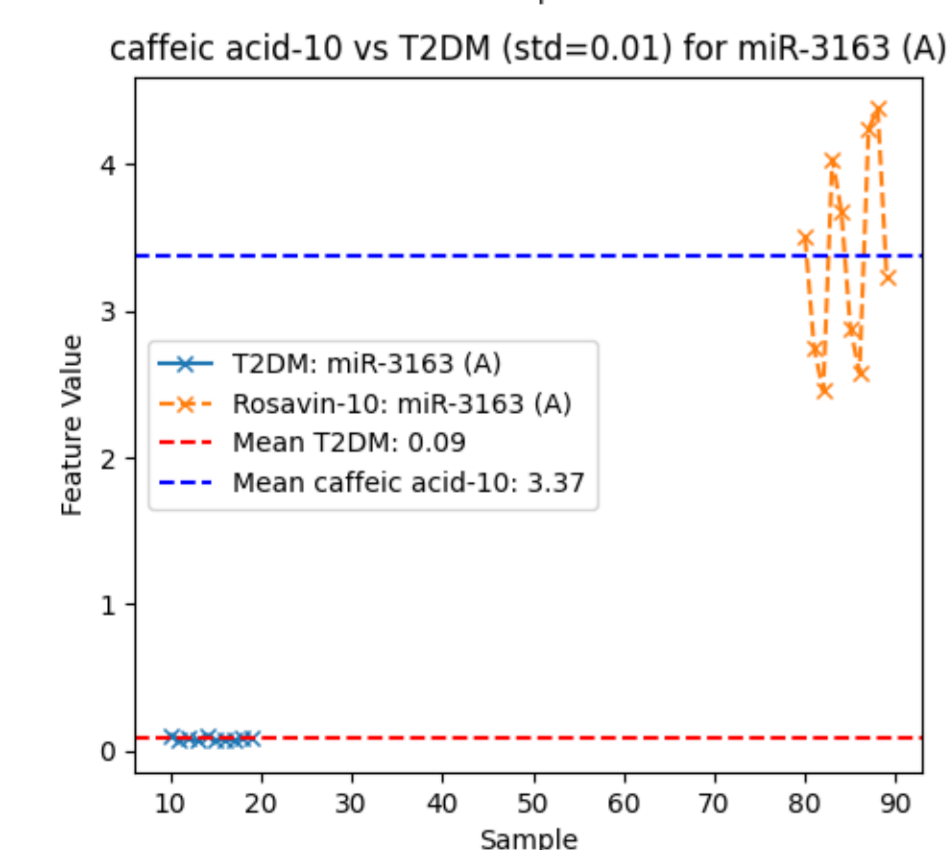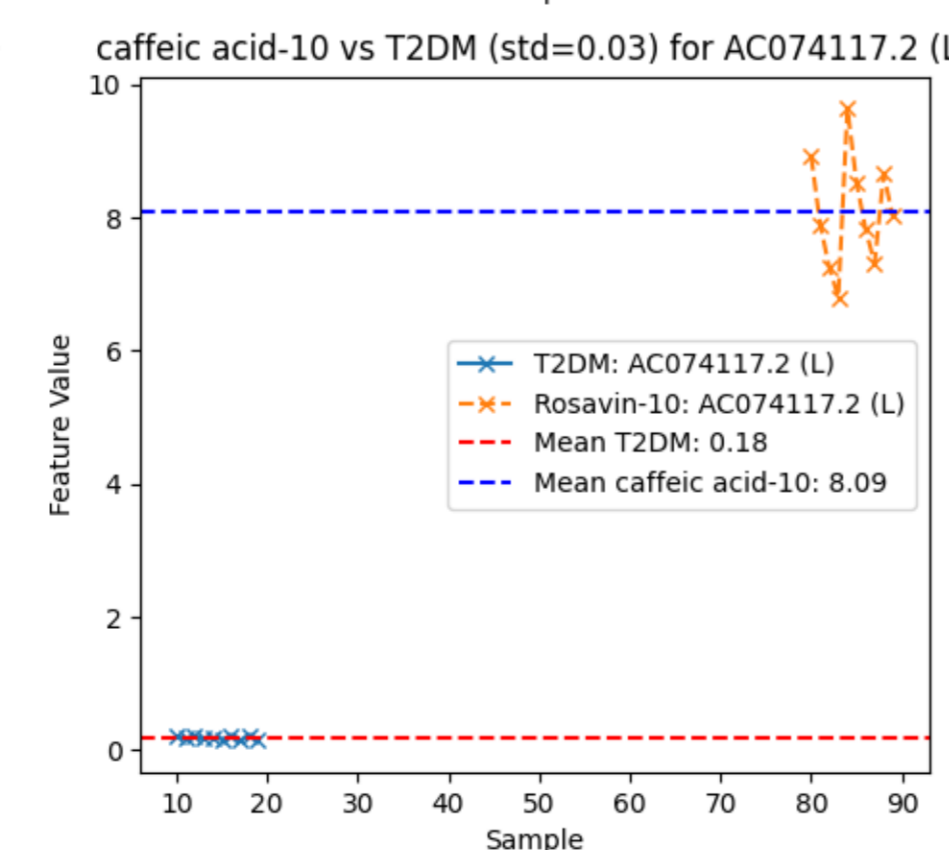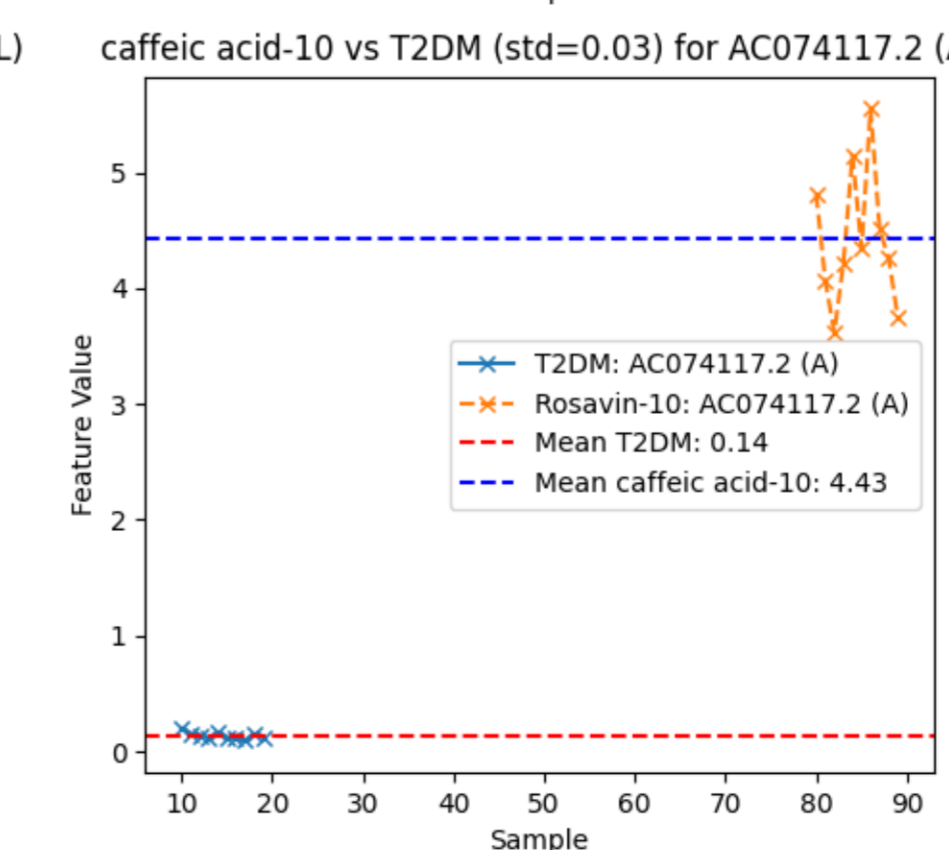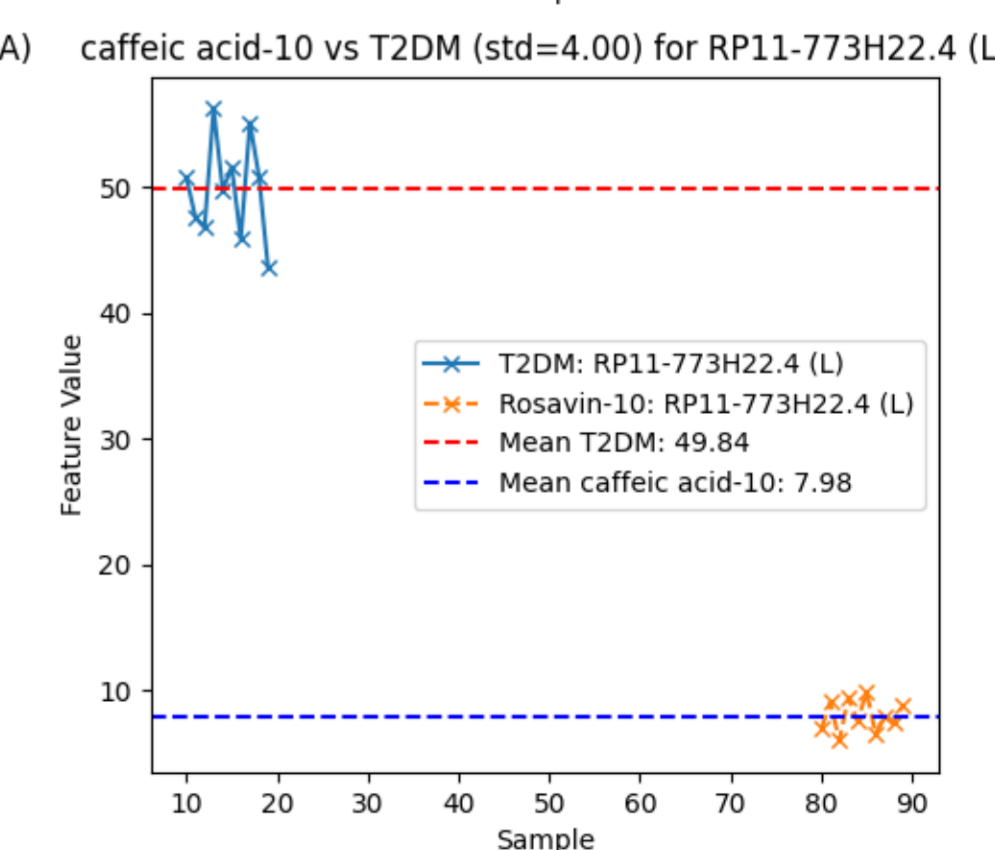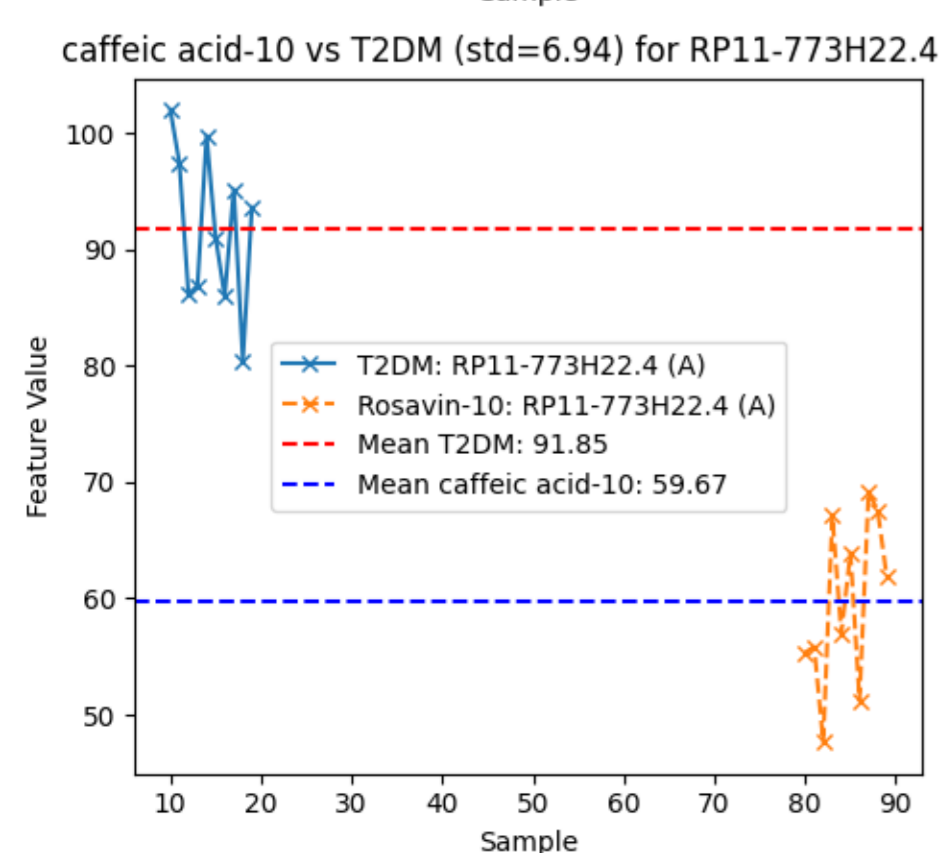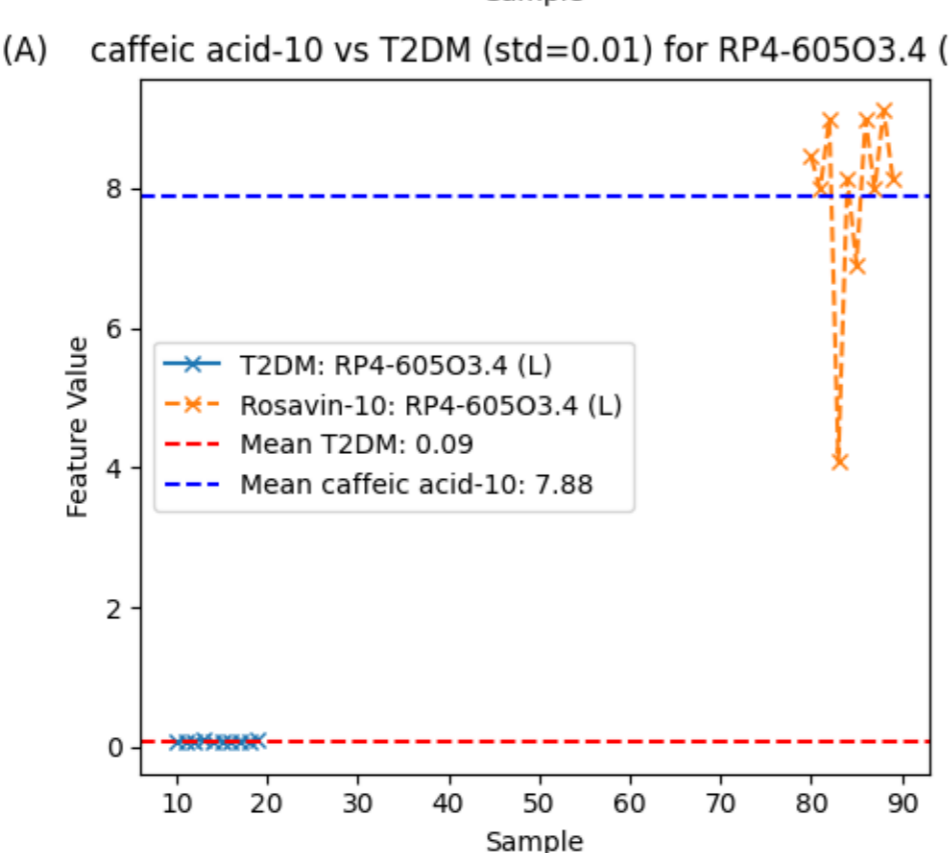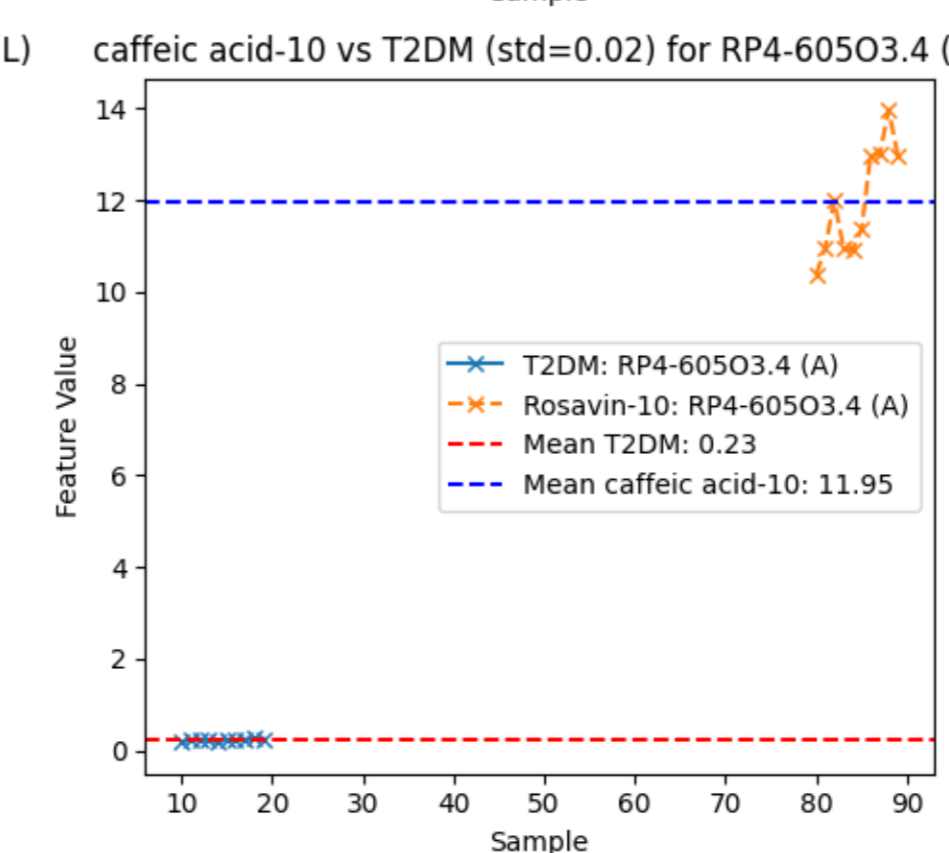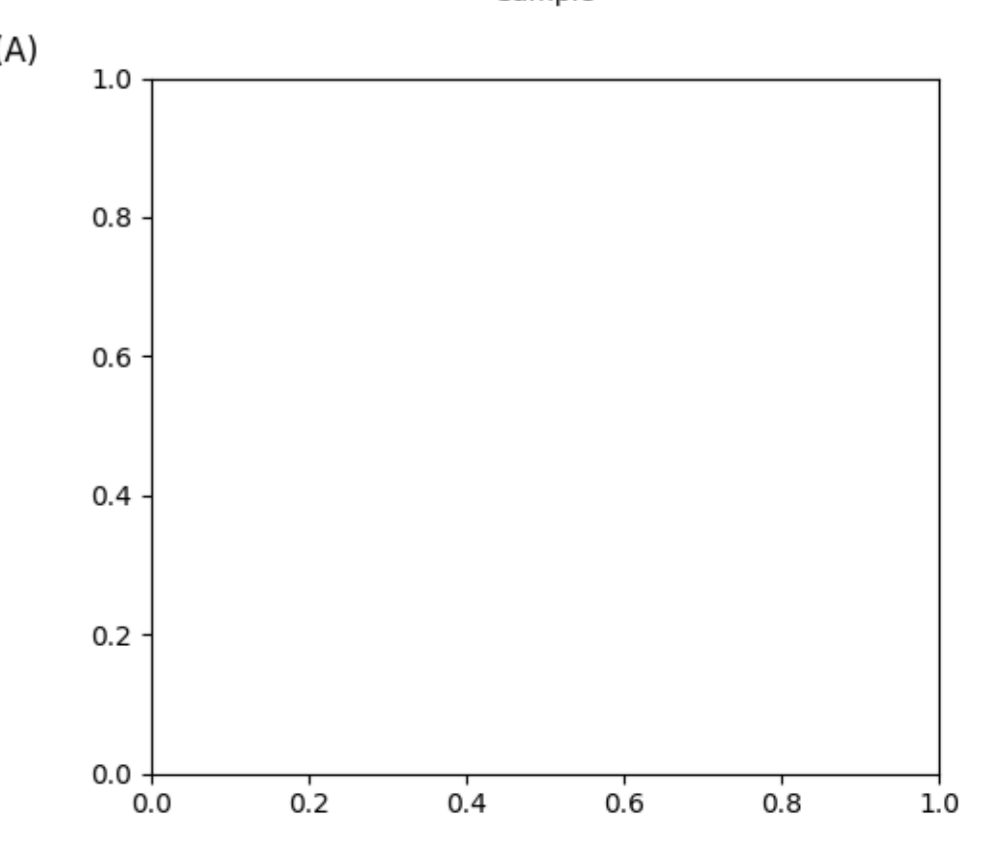

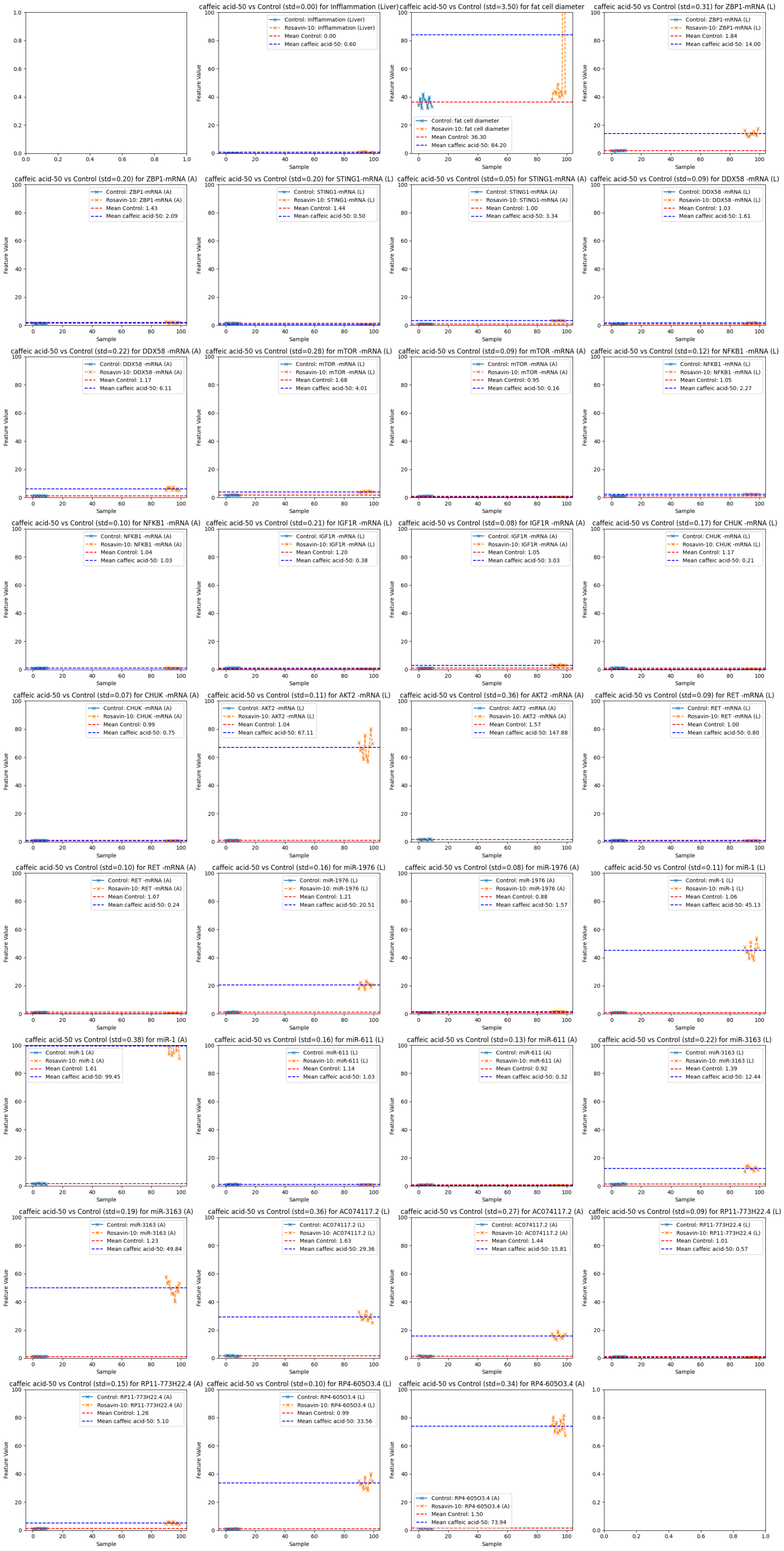

Supplement: Supplementary file 2 [file DataSheet_2.pdf]
