# Supplementary material for: Comprehensive machine learning models for predicting therapeutic targets in type 2 diabetes utilizing molecular and biochemical features in rats
Source: Front Endocrinol (Lausanne). 2024 May 24;15:1384984. doi: 10.3389/fendo.2024.1384984 (PMC11157016; doi:10.3389/fendo.2024.1384984)

Figure S7: Show treated samples vs control vs T2DM

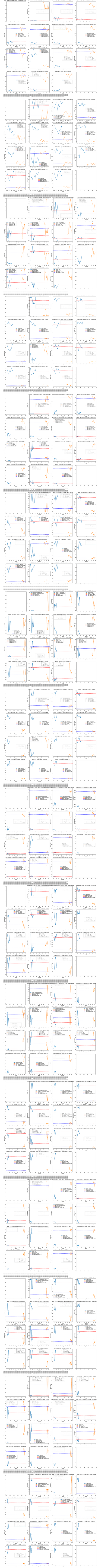

Supplement: Supplementary file 3 [file DataSheet_3.pdf]
